# Construction of trisubstituted chromone skeletons carrying electron-withdrawing groups via PhIO-mediated dehydrogenation and its application to the synthesis of frutinone A

**DOI:** 10.3762/bjoc.15.291

**Published:** 2019-12-12

**Authors:** Qiao Li, Chen Zhuang, Donghua Wang, Wei Zhang, Rongxuan Jia, Fengxia Sun, Yilin Zhang, Yunfei Du

**Affiliations:** 1 Tianjin Key Laboratory for Modern Drug Delivery & High-Efficiency, School of Pharmaceutical Science and Technology, Tianjin University, Tianjin 300072, China; 2 College of Chemical and Pharmaceutical Engineering, Hebei University of Science and Technology; Hebei Research Center of Pharmaceutical and Chemical Engineering, Shijiazhuang 050018, China; 3 C. Eugene Bennett Department of Chemistry, West Virginia University, Morgantown, WV 26506-6045, United States

**Keywords:** chromanone, chromone, dehydrogenation, frutinone A, PhIO

## Abstract

The construction of the biologically interesting chromone skeleton was realized by PhIO-mediated dehydrogenation of chromanones under mild conditions. Interestingly, this method also found application in the synthesis of the naturally occurring frutinone A.

## Introduction

**Figure 1:**
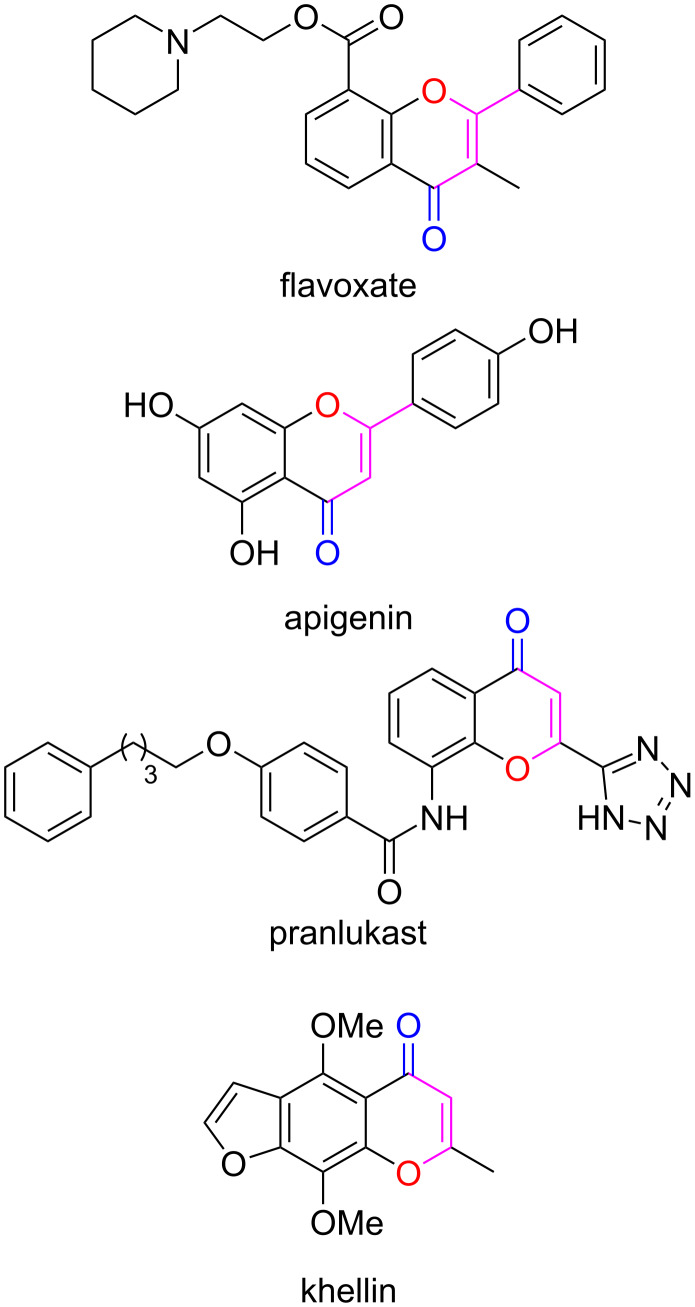
Biologically active chromone derivatives.

The chromone system and its derivatives are an important class of heterocyclic compounds, the skeleton of which widely exists in a variety of natural products and medicinal agents [1–3]. It was found that chromone derivatives exhibit a wide range of pharmacological effects, including antibacterical [4], antifungal [5–6], anticancer [7], antioxidant [8], anti-HIV [9], antiulcer, immunostimulator [10], anti-inflammatory [11], as well as biocidal [12], wound-healing [13], and immune-stimulatory activities [14]. For instance, flavoxate [15–16] is a chromone derivative that was employed as an anticholinergic agent for its antimuscarinic effects [3,17]; apigenin can function as an antiviral drug for the treatment of HIV [18–19], cancer [20–22], and other viral infections [23]; pranlukast [24] can be used in the treatment of allergic rhinitis [25] and asthma [26]; and khellin [27] has been proved to possess antiviral and antispasmodic effects (Figure 1) [28]. All of these pharmaceutical agents bear a chromone framework in their respective chemical structure.

**Scheme 1:**
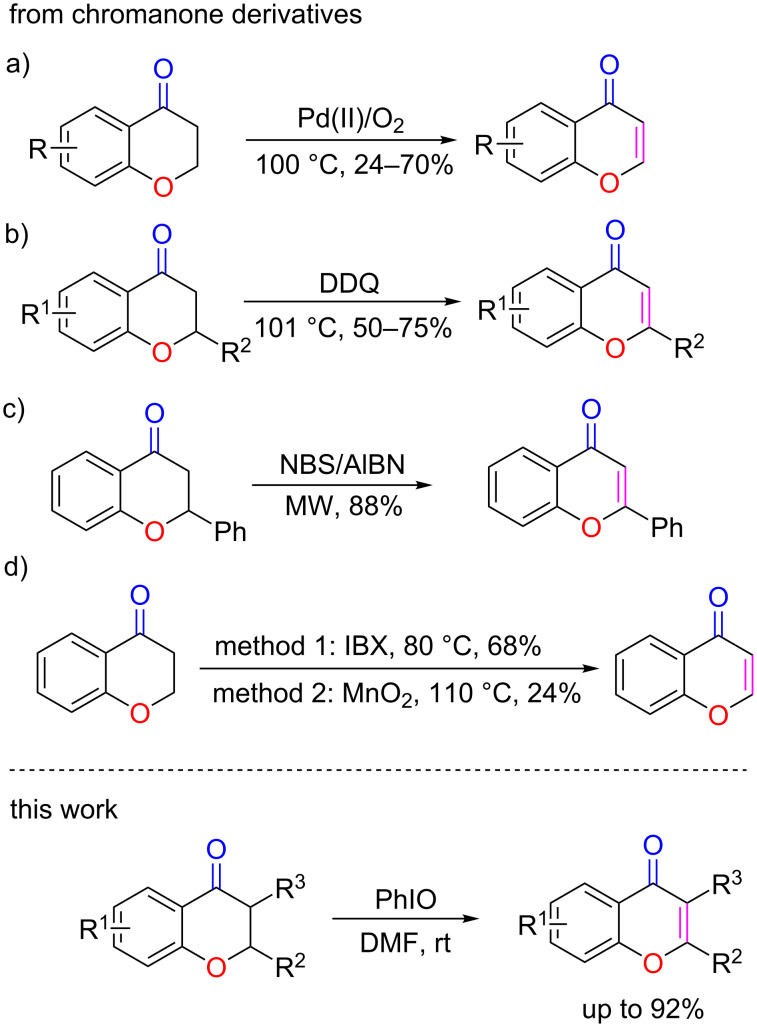
Methods for the synthesis of chromones via dehydrogenative oxidation of chromanones.

Since the chromone derivatives have a variety of biological activities, developing synthetic methods for efficient construction of the chromone skeleton has been a research field of great interest and long history [2]. Among the numerous synthetic approaches [29–54], the dehydrogenative oxidation of the readily available chromanones is a highly efficient method for the synthesis of this privileged class of heterocycles. For example, Kim and co-workers reported that chromones could be prepared via a Pd(II)-catalyzed [55–57] dehydrogenation of chromanone derivatives at 100 °C in DMSO (Scheme 1a) [58]. The synthesis of chromones could also be realized by DDQ-mediated dehydrogenation of chromanones under heating in dioxane (Scheme 1b) [3,59–60]. In 2005, Yang and co-workers reported that chromones could be formed by microwave irradiation of the corresponding chromanone reactants and *N*-bromosuccinimide (NBS) in the presence of a catalytic amount of azobis(isobutyronitrile) (AIBN) in CCl_4_ (Scheme 1c) [61–62]. In 2002, Nicolaou and co-workers found that *ortho*-iodoxybenzoic acid (IBX) could also effectively dehydrogenate chromanones to chromones (Scheme 1d, method 1) [63]. Moreover, active MnO_2_ was also found useful in the oxidative dehydrogenation of chromanones at a relatively high temperature of 110 °C (Scheme 1d, method 2) [64–65]. Although all of the above methods have their respective merits in the preparation of the corresponding chromone derivatives, it is obvious that some of them suffer from drawbacks, such as the requirement of a high reaction temperature, extended reaction time, involvement of transition metal catalysts, and low yield. In these regards, the development of alternative approaches that can realize an efficient synthesis of chromones under mild conditions is desirable.

In recent decades, hypervalent iodine reagents have emerged as a class of efficient and environmentally benign nonmetal “green” oxidants [66–73]. For instance, iodosobenzene (PhIO) [74] has been widely used in many synthetic transformations. It was found that PhIO is efficient in realizing epoxidation of olefins [75–77], converting alkynes and alkenes to ketones [78], oxidizing alcohols to aldehydes [79–80], as well as in the direct α-hydroxylation of ketones [81]. Furthermore, it could also be used to realize oxidative C–C [82], C–N [83], and C–O [84] bond formations. However, to the best of our knowledge, PhIO has never been utilized for the dehydrogenative oxidation reaction. In this letter, we report a facile PhIO-mediated dehydrogenation of chromanones, resulting in the efficient synthesis of biologically interesting chromone compounds under metal-free conditions.

## Results and Discussion

We initially studied the feasibility of converting chromanone ethyl 4-oxo-2-phenylchromane-3-carboxylate (**1a**) to chromone **2a** via PhIO-mediated dehydrogenation. To our delight, when **1a** was treated with PhIO in DCE at room temperature, the desired product **2a** could be obtained in 66% yield (Table 1, entry 1), with the generation of some unidentified byproducts. A solvent screening identified DMF to be the most appropriate solvent for this transformation (Table 1, entries 1–9). Other commonly employed oxidants, including phenyliodine(III) diacetate (PIDA), phenyliodine(III) bis(trifluoroacetate) (PIFA), and iodylbenzene (PhIO_2_), were found to be less efficient for this transformation, as the desired product **2a** was generated in significantly lower yield in each case (Table 1, entries 10–12). Further studies indicated that the reaction gave the best result if performed at room temperature, while reduced or elevated temperature (0 or 50 °C) was not beneficial for the reaction (Table 1, entries 13 and 14). In addition, the amount of PhIO was proved to be critical for this transformation, as a decreased yield of the desired product, accompanied with more byproducts, was observed when increasing the PhIO amount from 2.0 to 3.0 mmol. When the amount of PhIO was reduced to 1.0 mmol, the yield also decreased due to the fact that the starting material could not be completely consumed (Table 1, entries 15 and 16). Based on the above screening results, the optimal reaction conditions were concluded to be 1.0 mmol of substrate, 2.0 mmol of PhIO, DMF as solvent, and 10 minutes reaction time at room temperature.

**Table 1:** Optimization of reaction conditions.^a^

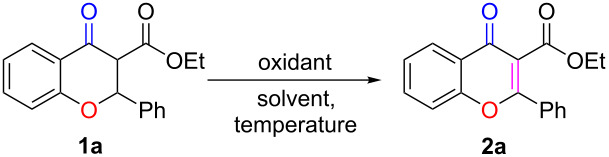				

entry	oxidant	solvent	*T* (°C)	yield (%)^b^

1	PhIO	DCE	rt	66
2	PhIO	THF	rt	57
3	PhIO	toluene	rt	42
4	PhIO	1,4-dioxane	rt	33
5	PhIO	DMSO	rt	53
6	PhIO	DMF	rt	89
7	PhIO	MeOH	rt	33
8	PhIO	MeCN	rt	37
9	PhIO	EtOAc	rt	35
10	PIDA	DMF	rt	62
11	PIFA	DMF	rt	21
12	PhIO_2_	DMF	rt	46
13	PhIO	DMF	0	38
14	PhIO	DMF	50	65
15^c^	PhIO	DMF	rt	64
16^d^	PhIO	DMF	rt	78

^a^Reaction conditions: **1a** (1.0 mmol), oxidant (2.0 mmol), solvent (6 mL). ^b^Isolated yield. ^c^**1a** (1.0 mmol), PhIO (1.0 mmol), DMF (6 mL). ^d^**1a** (1.0 mmol), PhIO (3.0 mmol), DMF (6 mL), 10 min.

**Scheme 2:**
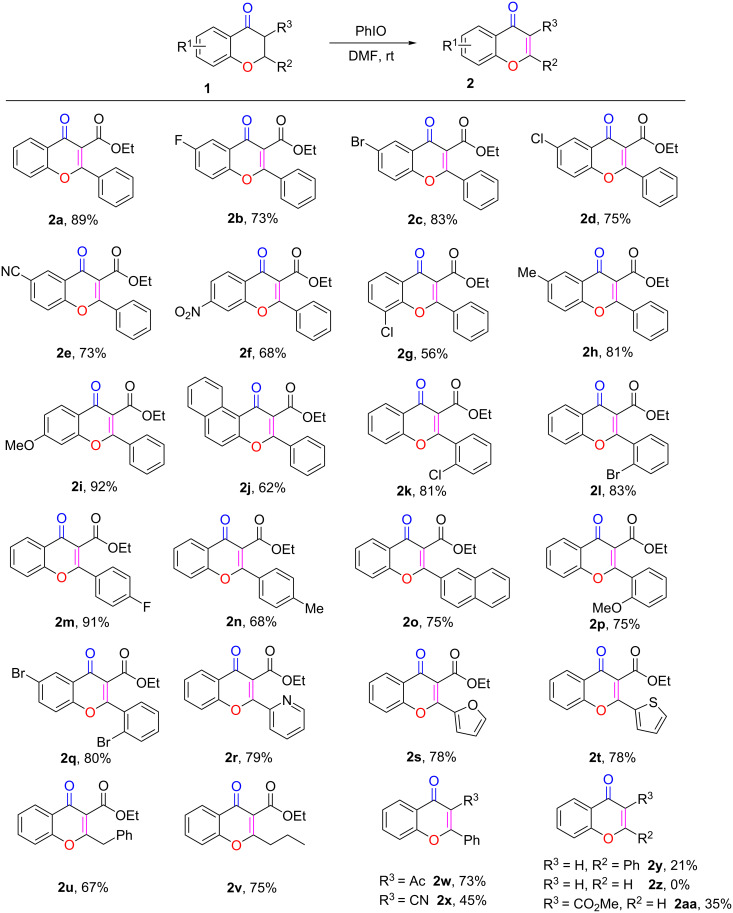
Substrate scope studies. Reaction conditions: **1** (1.0 mmol), PhIO (2.0 mmol), DMF (6 mL), rt. Isolated yields are given.

With the optimized conditions in hand, we explored the scope and generality of the newly established method. As shown in Scheme 2, a wide range of chromanones could be well tolerated under the standard conditions, affording the expected products **2a**–**x** in satisfactory to good yields. Substrates bearing various R^1^ substituents (F, Cl, Br, CN, NO_2_, Me, OMe) were all efficiently converted to the corresponding products **2a**–**i**, with the substrates carrying electron-donating groups affording the desired products **2h** and **2i** in noticeably higher yields than those with electron-withdrawing groups, **2b**–**g**. The product **2g**, with an electron-withdrawing group in *ortho* position, was formed in a much lower yield, most likely due to steric hindrance. When the phenyl group was replaced by a naphthyl group, less of the corresponding substrate was converted to the desired product **2j**. The substrates bearing various R^2^ substituents, including electron-withdrawing, electron-donating, sterically hindered, and heterocyclic groups, all reacted smoothly and afforded the desired chromone derivatives **2k**–**v** in acceptable to good yields. Furthermore, when the ethoxycarbonyl group R^3^ was replaced by an acetyl substituent, the resulting substrate was successfully converted to **2w** with a high yield. However, when the substrate bore a strong electron-withdrawing cyano group, the reaction was less efficient. and the product **2x** was produced in a much lower yield. When the electron-withdrawing group (R^3^) in substrate **1** was replaced by hydrogen, the corresponding product **2y** was obtained in a relatively low yield. To our disappointment, the method was not applicable to the synthesis of 4-chromones, as the reaction of 4-chromanones did not occur under the standard conditions (**2z**). But when R^3^ was a carbonyl group and R^2^ was a hydrogen atom, the corresponding compound **1aa** could also be converted to the desired product with 35% yield. On the basis of these results, we tentatively proposed that the failure of **1z** formation might have been caused by the absence of a carbonyl group in its chemical structure [85].

**Scheme 3:**
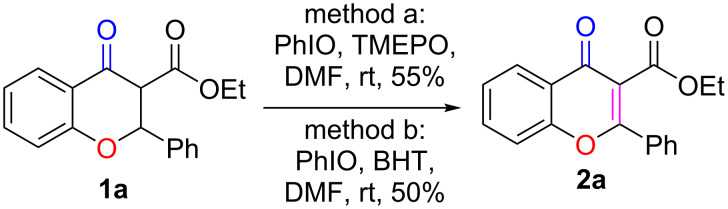
Control experiments for mechanistic studies.

Control experiments were designed and conducted to elucidate the reaction mechanism of this transformation. When 3 equiv of TEMPO, a radical-trapping reagent, were added to the reaction mixture, product **2a** was obtained in 55% yield (Scheme 3, method a). When another radical-trapping reagent, BHT, was used, the reaction gave 50% yield of the desired product **2a** (Scheme 3, method b). Since the reaction was not greatly suppressed in both cases, we tentatively propose that this dehydrogenative oxidation reaction may undergo both radical and nonradical pathways.

**Scheme 4:**
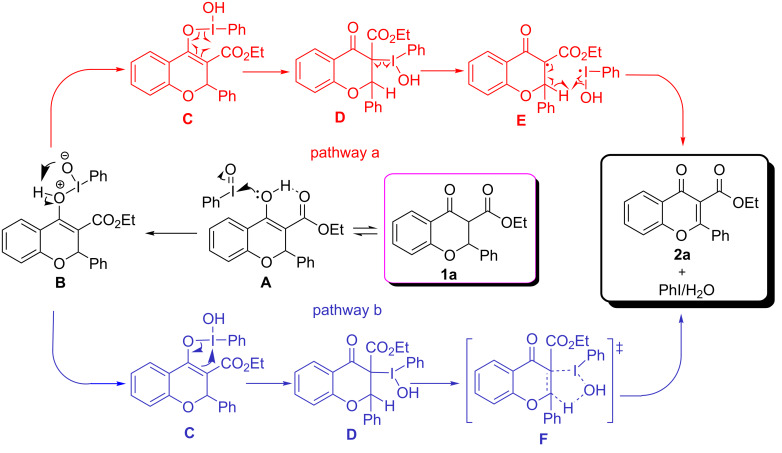
Proposed reaction mechanism.

Based on previous reports [63,86] and our own experimental results, two plausible mechanisms for this dehydrogenation reaction are proposed (Scheme 4): In pathway a, substrate **1a** is first tautomerized to its enol form **A**, which would be stabilized by an internal H-bond. It is highly likely that the enolization is a necessary process for the reaction to occur, as compound **2z** could not undergo the transformation under the standard conditions. Then, nucleophilic attack on the iodine center of PhIO [87] by the enol moiety of intermediate **A** affords the O–I enol form **C** (via intermediate **B**), which is subsequently converted to I–C intermediate **D** via radical migration [88]. The homogeneous cleavage of the C–I bond in **D** leads to the stable carbon radical **E** and an iodine radical. Finally, the reaction between **E** and the iodine radical produces product **2a**, with the concomitant release of water and iodobenzene. In pathway b, the O–I intermediate **C** is converted to a C–I intermediate **D** via 1,3-migration [89]. Then, intermediate **D** carries through a five-membered ring transition state **F** to afford the title product **2a**, accompanied by the release of iodobenzene and water.

**Scheme 5:**
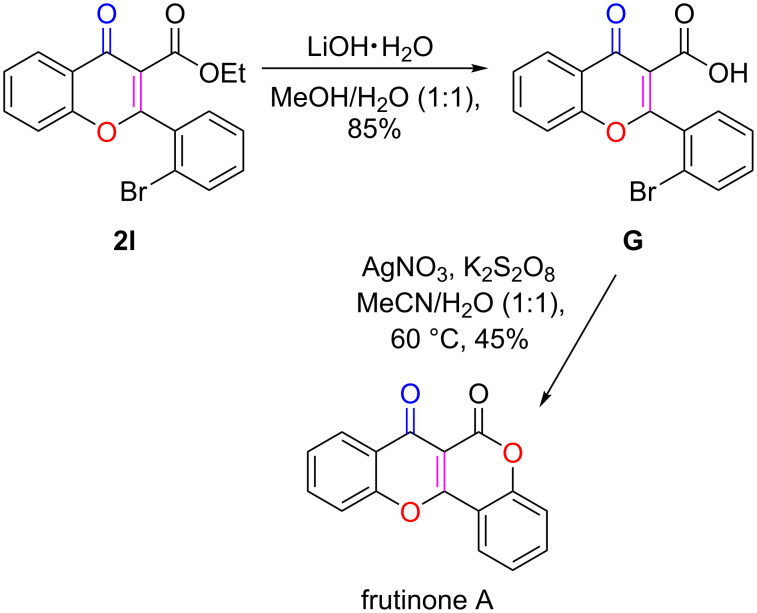
Application of the reported method to the synthesis of frutinone A.

One practical application of the obtained chromone derivatives was their conversion to chromone-derived natural products. Frutinone A, isolated from the leaves and root bark of *Polygala fruticosa*, shows various biological activities, including antibacterial, antioxidant, and potent cytochrome P450 1A2 inhibition (CYP1A2, IC_50_ = 5.3 nM) properties [90–92]. Treating the obtained chromene-3-carboxylate **2l** with LiOH [93] led to the formation of the chromene-3-carboxylic acid **G**. Heating compound **G** overnight in the presence of AgNO_3_ and K_2_S_2_O_8_ afforded frutinone A in an isolated yield of 45% (Scheme 5).

## Conclusion

In summary, we have developed an efficient metal-free approach for the synthesis of chromone derivatives via PhIO-mediated dehydrogenative oxidation of chromanones. Compared with the existing methods, the new reaction features mild conditions, high efficiency, and is metal-free. Moreover, the product **2l** could be further applied to the synthesis of the naturally occurring frutinone A.

## Supporting Information

File 1 Synthetic details and compound characterization data.
