# Supplementary material for: Construction of trisubstituted chromone skeletons carrying electron-withdrawing groups via PhIO-mediated dehydrogenation and its application to the synthesis of frutinone A
Source: Beilstein J Org Chem. 2019 Dec 12;15:2958–65. doi: 10.3762/bjoc.15.291 (PMC6941426; doi:10.3762/bjoc.15.291)
Supplement: File 1 — Synthetic details and compound characterization data. [file Beilstein_J_Org_Chem-15-2958-s001.pdf]

BEILSTEIN JOURNAL OF ORGANIC CHEMISTRY

Supporting Information

for

Construction of trisubstituted chromone skeletons carrying electron-withdrawing groups via PhIO-mediated dehydrogenation and its application to the synthesis of frutinone A

Qiao Li, Chen Zhuang, Donghua Wang, Wei Zhang, Rongxuan Jia, Fengxia Sun, Yilin Zhang and Yunfei Du

Beilstein J. Org. Chem. **2019,** *15,* 2958–2965. doi:10.3762/bjoc.15.291

Synthetic details and compound characterization data

License and Terms: This is a supporting information file under the terms of the Creative Commons Attribution License (https://creativecommons.org/licenses/by/4.0). Please note that the reuse, redistribution and reproduction in particular requires that the authors and source are credited.
The license is subject to the *Beilstein Journal of Organic Chemistry* terms and conditions: (https://www.beilstein-journals.org/bjoc)

CONTENTS:

1. General information S2

2. Experimental section S2

   2.1 Preparation of substrates **1** S2

   2.2 Preparation of compounds **2** S3

   2.3 Preparation of frutinone A S4

   2.4 Preparation of iodosobenzene S4

3. Spectral data of substrates and products S5

   3.1 Spectral data of substrates S5

   3.2 Spectral data of products S18

4. The HRMS analysis of the PhIO/DMF reaction system S30

5. References S31

6. ^{1}H NMR and ^{13}C NMR spectra S32

1. General information

All reactions were carried out at room temperature under air unless otherwise stated. 1H and 13C NMR spectra were recorded on a 400 MHz or 600 MHz spectrometer at 25 °C. Chemical shifts values are given in ppm and referred as the internal standard to TMS: 0.00 ppm. The peak patterns are indicated as follows: s, singlet; d, doublet; t, triplet; q, quartet; qui, quintet; m, multiplet and dd, doublet of doublets, brs, broad singlet. The coupling constants *J* are reported in Hertz (Hz). High resolution mass spectrometry (HRMS) was obtained on a Q-TOF micro spectrometer. Melting points were determined with a Micromelting point apparatus without corrections. Organic solutions were concentrated by rotary evaporation below 40 °C in vacuum. TLC plates were visualized by exposure to ultraviolet light.

All the chemicals and solvents were purchased from commercial sources and used without further purification, unless otherwise noticed. All reactions were carried out in over-dried glassware. Flash column chromatography was performed over silica gel (200–300 mesh) using a mixture of ethyl acetate (EtOAc), petroleum ether (PE) and dichloromethane (DCM). Before use, iodosobenzene was triturated in 75 mL. of chloroform in a beaker, then it was separated by filtration and air-dried. Iodometric titration shows the product to be more than 99% pure [1].

2. Experimental section

2.1 Preparation of substrates 1

General procedure

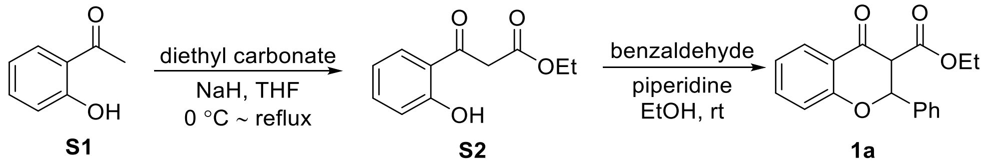

Substrates **1** were prepared adapted from a previously reported procedure [2,3].

To a suspension of ketone (5.0 mmol, 1.0 equiv) in THF (10 mL) was added the ester (10.0 mmol, 2.0 equiv). After the reaction mixture was stirred at 0 °C for about

10 min, NaH (15.0 mmol, 3.0 equiv, 60%) was added at the same temperature. Then the mixture was heated to reflux until TLC indicated the total consumption of the ketone. Removed the solvent in vacuum and the residue was mixed with ice-water (100 mL), acidified with aqueous HCl (3 M) to pH 2–3 and extracted with EtOAc (100 mL × 3). The combined organic layer was dried over sodium sulfate and evaporated under reduced pressure. The crude product was purified by flash column chromatography (EtOAc/PE = 5/95).

Ester compound thus obtained was dissolved in EtOH (6 mL) followed by addition of benzaldehyde (2.0 mmol, 1.0 equiv). After that, piperidine (0.1 mmol, 20 mmol %) was added to the above ethanol solution. The resulting mixture was stirred at ambient temperature for about 12 h. The solvent was removed in vacuo and the crude product was purified by flash column chromatography (EtOAc/PE = 5/95) to give the desired compounds **1**.

2.2 Preparation of compounds 2

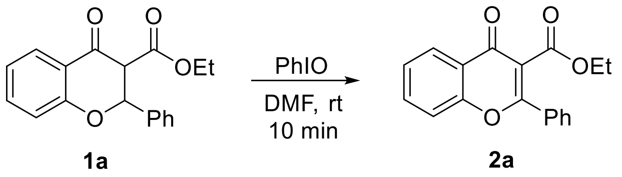

Compound **1a** (1.0 mmol, 1.0 equiv) was dissolved in DMF (4 mL) with stirring at ambient temperature. PhIO (2.0 mmol) was added to the above solution. The resulting mixture was stirred at ambient temperature for about 10 min until TLC indicated that the total consumption of substrate **1a**. Then the mixture was poured into H_2O (30 mL) and the reaction mixture was extracted with EtOAc (30 mL × 2). The organic layer was separated and dried over anhydrous sodium sulfate. The solvent was evaporated under reduced pressure, and the crude product was purified by flash column chromatography (EtOAc/PE = 15/85) to give the desired compounds **2a**.

2.3 Preparation of frutinone A

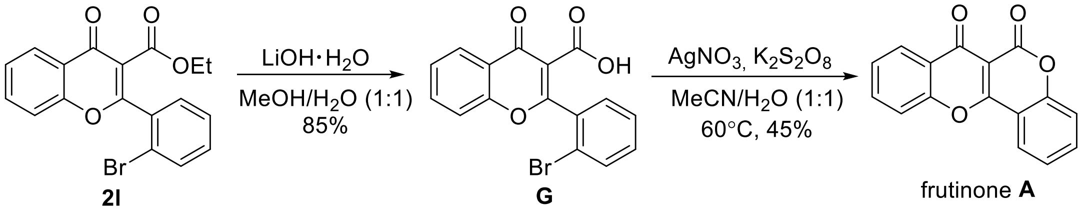

Frutinone **A** was prepared with reference to a previously reported procedure[4].

To a solution containing **2l** (744 mg, 2.0 mmol) in MeOH/H_2O (6 mL, V:V = 1:1) at rt was added LiOH·H_2O (840.0 mg, 20.0 mmol). The resulting mixture was stirred for 4 h and organic solvent was removed under reduced pressure. The crude residue was then diluted with H_2O (20 mL) and partitioned with EtOAc (20 mL). The aqueous layer was acidified with aq. HCl (1 N) to pH ≈3 and extracted with EtOAc (15 × 3 mL). The combined organic layer was then washed with a saturated aqueous NaCl solution, dried with Na_2SO_4, and concentrated under reduced pressure to give 586 mg (85% yield) of the acid **G** as a yellow solid. Then, to a solution containing acid **G** (345 mg, 1.0 mmol) MeCN/H_2O (3 mL, V:V = 1:1) solution, were added $K_2S_2O_8$ (714 mg, 3 mmol) and $AgNO_3$ (17 mg, 0.01 mmol). The solution mixture was heated at 60 °C for 15 h, cooled to rt, and quenched with a saturated aqueous $NaHCO_3$ solution (10 mL). After stirring for additional 15 mins, the mixture was extracted with a iPrOH/CH_2Cl_2 (10 × 3 mL, V:V = 1:3) solution. The combined organic layer was then washed with a saturated aqueous NaCl solution, dried with Na_2SO_4, and concentrated under reduced pressure. The crude residue was pre-absorbed on silica gel and purified by flash silica gel column chromatography using (EtOAc/PE = 20/80) as an eluent to give 119 mg (45%) of frutinone **A**.

2.4 Preparation of iodosobenzene

Finely ground iodosobenzene diacetate (32.2 g, 0.10 mol) is placed in a 250 mL beaker, and aq. NaOH (3 N, 150 mL) is added over a 5-min period with vigorous stirring. The lumps of solid that formed are triturated with a stirring rod or spatula for

15 min, and the reaction mixture stands for an additional 45 min. Then H_2O (100 mL) is added, and the mixture is stirred vigorously. The crude solid iodosobenzene is collected on a Büchner funnel. The wet solid is returned to the beaker and triturated in H_2O (200 mL). The solid is again collected on the Büchner funnel, washed there with H_2O (200 mL), and dried by maintaining suction. Final purification is effected by triturating the dried solid in $CHCl_3$ (75 mL) in a flask. The resulting iodosobenzene is separated by filtration and air-dried as a pure yellow solid, 20.2 g (92%); m.p. 210–212 °C.

3. Spectral data of substrates and products

3.1 Spectral data of substrates

Ethyl 4-oxo-2-phenylchromane-3-carboxylate (1a)

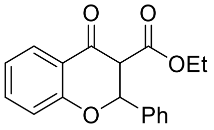

Following the general procedure, **1a** was purified by silica gel chromatography (5% EtOAc/PE). Yield: 523 mg, 82%, a white solid, mp. 93 – 94 °C. ^{1}H NMR (600 MHz, $CDCl_3$) δ 7.96 (dd, *J* = 7.8, 1.6 Hz, 1H), 7.57 – 7.52 (m, 1H), 7.51 (dd, *J* = 7.8, 1.6 Hz, 2H), 7.45 – 7.39 (m, 3H), 7.09 (t, *J* = 7.6 Hz, 1H), 7.04 (d, *J* = 8.4 Hz, 1H), 5.69 (d, *J* = 12.4 Hz, 1H), 4.13 – 4.08 (m, 2H), 1.10 (t, *J* = 7.2 Hz, 3H). ^{13}C NMR (151 MHz, $CDCl_3$) δ 187.8, 167.0, 161.1, 136.7, 136.5, 129.5, 128.8, 127.5, 127.4, 122.1, 120.0, 118.2, 81.4, 61.5, 59.8, 14.0. HRMS (ESI) calcd for $C_{18}H_{16}NaO_4^+$ [M + Na$^+$] 319.0941, found 319.0941.

Ethyl 6-fluoro-4-oxo-2-phenylchromane-3-carboxylate (1b)

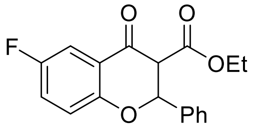

Following the general procedure, **1b** was purified by silica gel chromatography (5% EtOAc/PE). Yield: 459 mg, 68%, a white solid, mp. 100 – 102 °C. ^{1}H NMR (600 MHz, $CDCl_3$) δ 12.29 (s, 1H), 7.39 –7.31 (m, 3H), 7.31 – 7.25 (m, 3H), 6.95 (td, *J* = 8.5, 3.2 Hz, 1H), 6.75 (dd, *J* = 8.8, 4.4 Hz, 1H), 6.23 (s, 1H), 4.82 – 3.52 (m, 2H), 1.22 (t, *J* = 7.2 Hz, 3H). ^{13}C NMR (150 MHz, $CDCl_3$) δ 170.2, 161.8, 158.0, 156.5, 151.7, 139.9, 128.5, 128.4, 127.4, 127.1, 120.0($^{2}J_{C\text{-}F}$ = 23.61), 118.4($^{3}J_{C\text{-}F}$ = 7.665), 110.7, 110.5, 95.4, 74.9, 61.1, 14.1. HRMS (ESI) calcd for $C_{18}H_{15}FNaO_4^+$ [M + Na$^+$] 337.0847, found 337.0848.

Ethyl 6-bromo-4-oxo-2-phenylchromane-3-carboxylate (1c)

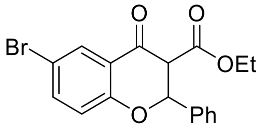

Following the general procedure, **1c** was purified by silica gel chromatography (5% EtOAc/PE). Yield: 579 mg, 73%, a white solid, mp. 84 – 86 °C. ^{1}H NMR (600 MHz, $CDCl_3$) δ 12.29 (s, 1H), 7.79 (d, *J* = 2.6 Hz, 1H), 7.38 – 7.31 (m, 3H), 7.30 – 7.26 (m, 3H), 6.68 (d, *J* = 8.7 Hz, 1H), 6.24 (s, 1H), 4.87 – 3.90 (m, 2H), 1.21 (t, *J* = 7.2 Hz, 3H). ^{13}C NMR (150 MHz, $CDCl_3$) δ 170.0, 161.4, 154.7, 139.8, 135.8, 128.6, 128.4, 127.1, 127.1, 119.5, 119.0, 113.5, 95.3, 75.2, 61.1, 14.1. HRMS (ESI) calcd for $C_{18}H_{15}{}^{79}BrNaO_4^+$ [M + Na$^+$] 397.0046, found 397.0047.

Ethyl 6-chloro-4-oxo-2-phenylchromane-3-carboxylate (1d)

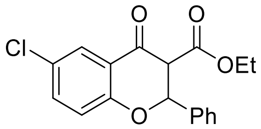

Following the general procedure, **1d** was purified by silica gel chromatography (5% EtOAc/PE). Yield: 530 mg, 75%, a white solid, mp. 90 – 92 °C. ^{1}H NMR (600 MHz, $CDCl_3$) δ 12.29 (s, 1H), 7.64 (d, *J* = 2.6 Hz, 1H), 7.35-7.31 (m, 2H), 7.31 – 7.26 (m, 3H), 7.20 (dd, *J* = 8.8, 2.6 Hz, 1H), 6.74 (d, *J* = 8.8 Hz, 1H), 6.24 (s, 1H), 4.96 – 3.26

(m, 2H), 1.21 (t, *J* = 7.2 Hz, 3H). ^{13}C NMR (150 MHz, $CDCl_3$) δ 170.1, 161.5, 154.3, 139.9, 133.0, 128.6, 128.5, 127.1, 126.4, 124.1, 119.0, 118.6, 95.4, 75.2, 61.1, 14.2. HRMS (ESI) calcd for $C_{18}H_{15}{}^{35}ClNaO_4^+$ [M + Na$^+$] 353.0551, found 353.0551.

Ethyl 6-cyano-4-oxo-2-phenylchromane-3-carboxylate (1e)

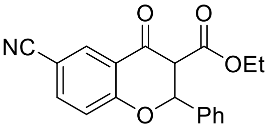

Following the general procedure, **1e** was purified by silica gel chromatography (5% EtOAc/PE). Yield: 536 mg, 78%, a light yellow solid, mp. 156 – 158 °C. ^{1}H NMR (600 MHz, $CDCl_3$) δ 8.25 (s, 1H), 7.75 (dd, *J* = 8.8, 2.0 Hz, 1H), 7.48 (m, 2H), 7.44 (m, 3H), 7.15 (d, *J* = 8.6 Hz, 1H), 5.77 (d, *J* = 12.0 Hz, 1H), 4.13-4.11 (m, 1H), 4.11 – 4.08 (m, 2H), 1.10 (t, *J* = 7.0 Hz, 3H). ^{13}C NMR (150 MHz, $CDCl_3$) δ 185.9, 166.1, 163.4, 138.9, 135.4, 132.6, 129.9, 129.0, 127.4, 120.3, 119.8, 117.7, 106.1, 81.8, 61.9, 59.0, 13.9. HRMS (ESI) calcd for $C_{19}H_{15}NNaO_4^+$ [M + Na$^+$] 344.0893, found 344.0894.

Ethyl 7-nitro-4-oxo-2-phenylchromane-3-carboxylate (1f)

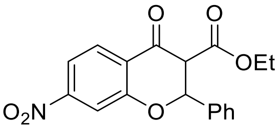

Following the general procedure, **1f** was purified by silica gel chromatography (5% EtOAc/PE). Yield: 604 mg, 83%, a yellow solid, mp. 100 – 102 °C. ^{1}H NMR (600 MHz, $CDCl_3$) δ 12.33 (s, 1H), 8.59 (d, *J* = 3.0 Hz, 1H), 8.14 (dd, *J* = 9.2, 3.0 Hz, 1H), 7.79 – 7.09 (m, 5H), 6.87 (d, *J* = 9.0 Hz, 1H), 6.36 (s, 1H), 4.27 – 4.19 (m, 2H), 1.21 (t, *J* = 7.2 Hz, 3H). ^{13}C NMR (150 MHz, $CDCl_3$) δ 169.6, 160.6, 160.1, 141.9, 139.3, 129.08, 128.7, 128.4, 127.1, 120.8, 117.7, 117.6, 95.8, 76.6, 61.4, 14.1. HRMS (ESI) calcd for $C_{18}H_{15}NNaO_6^+$ [M + Na$^+$] 364.0792, found 364.0792.

Ethyl 8-chloro-4-oxo-2-phenylchromane-3-carboxylate (1g)

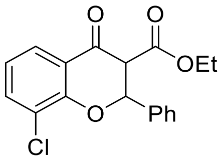

Following the general procedure, **1g** was purified by silica gel chromatography (5% EtOAc/PE). Yield: 607 mg, 86%, a white solid, mp. 106 – 108 °C. ^{1}H NMR (600 MHz, $CDCl_3$) δ 12.35 (s, 1H), 7.57 (dd, *J* = 7.8, 1.8 Hz, 1H), 7.40 (d, *J* = 7.2 Hz, 1H), 7.33 (dd, *J* = 8.0, 1.4 Hz, 2H), 7.31 – 7.24 (m, 3H), 6.88 (t, *J* = 7.8 Hz, 1H), 6.40 (s, 1H), 4.38 – 4.20 (m, 2H), 1.25 (t, *J* = 7.2 Hz, 3H). ^{13}C NMR (150 MHz, $CDCl_3$) δ 170.2, 162.3, 151.5, 139.8, 133.4, 128.4, 128.4, 126.9, 123.0, 122.1, 121.6, 119.6, 94.7, 75.0, 61.1, 14.2. HRMS (ESI) calcd for $C_{18}H_{15}{}^{35}ClNaO_4^+$ [M + Na$^+$] 353.0551, found 353.0551.

Ethyl 6-methyl-4-oxo-2-phenylchromane-3-carboxylate (1h)

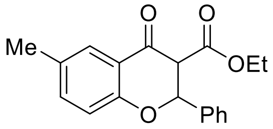

Following the general procedure, **1h** was purified by silica gel chromatography (5% EtOAc/PE). Yield: 576 mg, 87%, a white solid, mp. 76 – 78 °C. ^{1}H NMR (600 MHz, $CDCl_3$) δ 7.73 (s, 1H), 7.50 (d, *J* = 6.4 Hz, 2H), 7.44 – 7.38 (m, 3H), 7.34 (s, 1H), 6.94 (d, *J* = 8.4 Hz, 1H), 5.65 (d, *J* = 12.6 Hz, 1H), 4.10 (q, *J* = 7.2 Hz, 2H), 4.05 (d, *J* = 12.4 Hz, 1H)., 2.33 (s, 3H), 1.09 (t, *J* = 7.2 Hz, 3H). ^{13}C NMR (150 MHz, $CDCl_3$) δ 187.9, 167.1, 159.2, 137.8, 136.7, 131.6, 129.4, 128.8, 127.4, 127.0, 119.6, 118.0, 81.4, 61.4, 59.9, 20.5, 13.9. HRMS (ESI) calcd for $C_{19}H_{18}NaO_4^+$ [M + Na$^+$] 333.1097, found 333.1098.

Ethyl 7-methoxy-4-oxo-2-phenylchromane-3-carboxylate (1i)

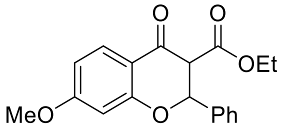

Following the general procedure, **1i** was purified by silica gel chromatography (5% EtOAc/PE). Yield: 621 mg, 89%, a white solid, mp. 102 – 104 °C.. ^{1}H NMR (600 MHz, DMSO) δ 7.75 (d, *J* = 8.8 Hz, 1H), 7.61 – 7.54 (m, 2H), 7.46 – 7.27 (m, 3H), 6.72 (dd, *J* = 8.8, 2.4 Hz, 1H), 6.66 (d, *J* = 2.4 Hz, 1H), 5.69 (d, *J* = 12.4 Hz, 1H), 4.58 (d, *J* = 12.4 Hz, 1H), 4.09 – 3.93 (m, 2H), 3.82 (s, 3H), 0.96 (t, *J* = 7.3 Hz, 1H). ^{13}C NMR (150 MHz, DMSO) δ 186.4, 167.0, 166.2, 162.8, 136.5, 129.3, 128.5, 128.4, 127.9, 113.1, 110.7, 101.0, 81.0, 60.6, 58.1, 56.0, 13.8. HRMS (ESI) calcd for $C_{19}H_{18}NaO_5^+$ [M + Na$^+$] 349.1046, found 349.1046.

Ethyl 1-oxo-3-phenyl-2,3-dihydro-1*H*-benzo[*f*]chromene-2-carboxylate (1j)

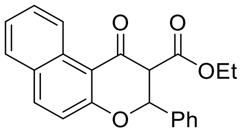

Following the general procedure, **1j** was purified by silica gel chromatography (5% EtOAc/PE). Yield: 575 mg, 78%, a yellow solid, mp. 110 – 112 °C. ^{1}H NMR (600 MHz, $CDCl_3$) δ 9.45 (dd, *J* = 8.6, 3.2 Hz, 1H), 7.96 (d, *J* = 9.0 Hz, 1H), 7.77 (d, *J* = 7.9 Hz, 1H), 7.65 (dd, *J* = 8.4, 7.4 Hz, 1H), 7.54 (d, *J* = 7.6 Hz, 2H), 7.49 – 7.38 (m, 4H), 7.13 (d, *J* = 9.0 Hz, 1H), 5.82 (d, *J* = 12.6 Hz, 1H), 4.20 (d, *J* = 12.6 Hz, 1H), 4.18 – 4.08 (m, 2H), 1.13 (td, *J* = 7.2, 2.2 Hz, 3H). ^{13}C NMR (151 MHz, $CDCl_3$) δ 188.7, 167.4, 163.4, 138.3, 136.4, 131.4, 130.1, 129.5, 129.4, 128.9, 128.6, 127.4, 125.8, 125.3, 118.6, 111.7, 81.2, 61.5, 60.5, 14.0. HRMS (ESI) calcd for $C_{22}H_{18}NaO_4^+$ [M + Na$^+$] 369.1097, found 369.1097.

Ethyl 2-(2-chlorophenyl)-4-oxochromane-3-carboxylate (1k)

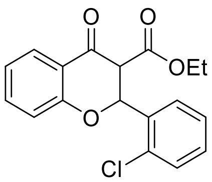

Following the general procedure, **1k** was purified by silica gel chromatography (5% EtOAc/PE). Yield: 551 mg, 78%, a white solid, mp. 72 – 74 °C. ^{1}H NMR (600 MHz,

CDCl3) δ 12.38 (s, 1H), 7.72 (d, *J* = 7.8 Hz, 1H), 7.44 – 7.39 (m, 1H), 7.29 (d, *J* = 7.7 Hz, 1H), 7.25 (dd, *J* = 10.2, 5.4 Hz, 1H), 7.21 (t, *J* = 7.8 Hz, 1H), 7.11 (t, *J* = 7.6 Hz, 1H), 6.96 (t, *J* = 7.5 Hz, 1H), 6.79 – 6.71 (m, 2H), 4.25 – 3.97 (m, 2H), 1.15 (t, *J* = 7.2 Hz, 3H). ^{13}C NMR (150 MHz, CDCl3) δ 163.1, 155.4, 136.8, 133.9, 133.4, 130.0, 129.9, 128.8, 126.8, 124.3, 121.3, 117.4, 117.2, 93.6, 71.5, 60.9, 14.0. HRMS (ESI) calcd for $C_{18}H_{15}{}^{35}ClNaO_4^+$ [M + Na$^+$] 353.0551, found 353.0551.

Ethyl 2-(2-bromophenyl)-4-oxochromane-3-carboxylate (1l)

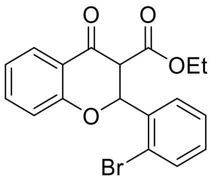

Following the general procedure, **1l** was purified by silica gel chromatography (5% EtOAc/PE). Yield: 635 mg, 80%, a white solid, mp. 78 – 80 °C. ^{1}H NMR (600 MHz, CDCl3) δ 12.38 (s, 1H), 7.71 (dd, *J* = 7.8, 1.5 Hz, 1H), 7.60 (dd, *J* = 7.6, 1.4 Hz, 1H), 7.28 (dd, *J* = 7.6, 1.8 Hz, 1H), 7.27 – 7.21 (m, 1H), 7.16 – 7.11 (m, 2H), 6.96 (t, *J* = 7.6 Hz, 1H), 6.77 – 6.68 (m, 2H), 4.31 – 4.01 (m, 2H), 1.15 (t, *J* = 7.2 Hz, 3H). ^{13}C NMR (150 MHz, CDCl3) δ 170.0, 163.1, 155.3, 138.4, 133.4, 133.3, 130.2, 129.0, 127.5, 124.3, 124.2, 121.3, 117.4, 117.3, 93.8, 74.0, 60.9, 14.0. HRMS (ESI) calcd for $C_{18}H_{15}{}^{79}BrNaO_4^+$ [M + Na$^+$] 397.0046, found 397.0046.

Ethyl 2-(4-fluorophenyl)-4-oxochromane-3-carboxylate (1m)

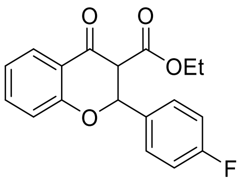

Following the general procedure, **1m** was purified by silica gel chromatography (5% EtOAc/PE). Yield: 472 mg, 70%, a white solid, mp. 66 – 68 °C. Major isomer: ^{1}H NMR (600 MHz, CDCl3) δ 7.96 (dd, *J* = 7.9, 1.7 Hz, 1H), 7.55 (dt, *J* = 8.6, 7.4 Hz, 1H), 7.53 – 7.47 (m, 2H), 7.31 – 7.26 (m, 1H), 7.15 – 7.08 (m, 2H), 7.03 (d, *J* = 8.0 Hz, 1H), 6.79

(dd, *J* = 8.2, 0.8 Hz, 1H), 5.67 (d, *J* = 12.6 Hz, 1H), 4.16 – 4.08 (m, 2H), 1.12 (t, *J* = 7.2 Hz, 3H). ^{13}C NMR (150 MHz, $CDCl_3$) δ 187.6, 164.0, 162.4, 161.0, 136.8, 133.4, 129.4($^3J_{C\text{-}F}$ = 8.385), 129.0, 127.6, 122.2, 118.1, 117.2, 115.2($^2J_{C\text{-}F}$ = 31.845), 80.6, 61.6, 59.9, 14.0. Minor isomer: ^{1}H NMR (600 MHz, $CDCl_3$) δ12.35 (s, 1H), 7.68 (dd, *J* = 7.8, 1.6 Hz, 1H), 7.36 – 7.31 (m, 2H), 7.11 – 7.08 (m, 2H), 7.00 – 6.93 (m, 3H), 6.22 (s, 1H), 4.28 – 4.18 (m, 2H), 1.22 (t, *J* = 7.2 Hz, 3H). ^{13}C NMR (150 MHz, $CDCl_3$) δ 166.9, 164.0, 163.5, 161.8, 161.0, 155.5, 136.2, 132.5, 129.3($^3J_{C\text{-}F}$ = 11.295), 128.9, 124.5, 121.4, 119.9, 117.7, 115.8($^2J_{C\text{-}F}$ = 21.54), 115.3, 74.2, 60.9, 14.2. HRMS (ESI) calcd for $C_{18}H_{15}FNaO_4^+$ [M + Na$^+$] 337.0847, found 337.0846.

Ethyl 4-oxo-2-(*p*-tolyl)chromane-3-carboxylate (1n)

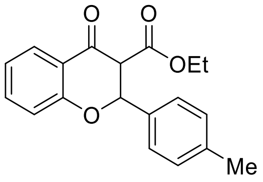

Following the general procedure, **1n** was purified by silica gel chromatography (5% EtOAc/PE). Yield: 506 mg, 76%, a white solid, mp. 126 – 128 °C. ^{1}H NMR (600 MHz, $CDCl_3$) δ 7.95 (dd, *J* = 7.8, 1.2 Hz, 1H), 7.53 (t, *J* = 7.8 Hz, 1H), 7.39 (d, *J* = 8.0 Hz, 2H), 7.22 (d, *J* = 7.8 Hz, 2H), 7.08 (t, *J* = 7.5 Hz, 1H), 7.02 (d, *J* = 8.3 Hz, 1H), 5.65 (d, *J* = 12.4 Hz, 1H), 4.15-4.11 (m, 2H), 4.09 – 4.05 (m, 1H), 2.37 (s, 3H), 1.12 (t, *J* = 7.2 Hz, 3H). ^{13}C NMR (150 MHz, $CDCl_3$) δ 188.0, 167.0, 161.2, 139.4, 136.7, 133.6, 129.5, 127.5, 127.3, 122.0, 120.0, 118.2, 81.2, 61.5, 59.7, 21.3, 14.0. HRMS (ESI) calcd for $C_{19}H_{18}NaO_4^+$ [M + Na$^+$] 333.1097, found 333.1097.

Ethyl 2-(naphthalen-2-yl)-4-oxochromane-3-carboxylate (1o)

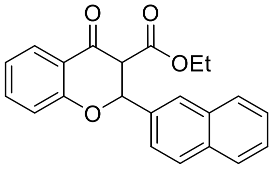

Following the general procedure, **lo** was purified by silica gel chromatography (5% EtOAc/PE). Yield: 635 mg, 86%, a white solid, mp. 118 – 120 °C. ^{1}H NMR (600 MHz, $CDCl_3$) δ 12.56 (s, 1H), 8.60 (t, *J* = 11.8 Hz, 1H), 7.86 (dd, *J* = 8.2, 3.2 Hz, 1H), 7.79 – 7.71 (m, 2H), 7.66 (dd, *J* = 14.2, 6.4 Hz, 1H), 7.56 – 7.52 (m, 1H), 7.46 – 7.34 (m, 1H), 7.29 (td, J = 7.7, 2.4 Hz, 1H), 7.12 (td, J = 8.4, 1.7 Hz, 1H), 7.10 – 7.04 (m, 1H), 6.9–6.89 (m, 1H), 6.60 (t, *J* = 7.2 Hz, 1H), 4.26 – 3.94 (m, 2H), 1.06 (td, *J* = 7.2, 1.4 Hz, 3H). ^{13}C NMR (151 MHz, $CDCl_3$) δ 170.4, 163.9, 155.6, 134.5, 134.1, 133.1, 131.8, 129.5, 128.7, 126.5, 125.8, 125.6, 124.9, 124.5, 124.4, 121.3, 118.0, 117.3, 93.4, 71.4, 60.8, 14.1. HRMS (ESI) calcd for $C_{22}H_{18}NaO_4^+$ [M + Na$^+$] 369.1097, found 369.1097.

Ethyl 2-(2-methoxyphenyl)-4-oxochromane-3-carboxylate (1p)

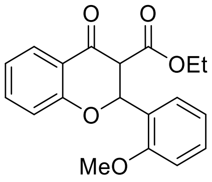

Following the general procedure, **1p** was purified by silica gel chromatography (5% EtOAc/PE). Yield: 558 mg, 80%, a white solid, mp. 132 – 134 °C. ^{1}H NMR (600 MHz, $CDCl_3$) δ7.75 (d, *J* = 8.8 Hz, 1H), 7.62 – 7.54 (m, 2H), 7.48 – 7.38 (m, 3H), 6.72 (dd, *J* = 8.8, 2.3 Hz, 1H), 6.66 (d, *J* = 2.3 Hz, 1H), 5.69 (d, *J* = 12.4 Hz, 1H), 4.58 (d, *J* = 12.4 Hz, 1H), 4.00 – 3.94 (m, 2H), 3.82 (s, 3H), 0.96 (t, *J* = 7.2 Hz, 3H). ^{13}C NMR (150 MHz, DMSO) δ 186.4, 167.0, 166.2, 162.8, 136.5, 129.3, 128.5, 128.5, 127.9, 113.1, 110.7, 101.0, 81.0, 60.6, 58.1, 56.0, 13.8. HRMS (ESI) calcd for $C_{19}H_{18}NaO_5^+$ [M + Na$^+$] 349.1046, found 349.1046.

Ethyl 6-bromo-2-(2-bromophenyl)-4-oxochromane-3-carboxylate (1q)

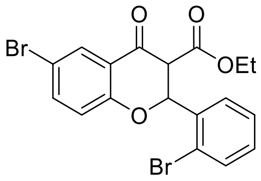

Following the general procedure, **1q** was purified by silica gel chromatography (5% EtOAc/PE). Yield: 678 mg, 75%, a white solid, mp. 108 – 110 ºC. ^{1}H NMR (600 MHz, $CDCl_3$) δ 12.31 (s, 1H), 7.82 (d, *J* = 2.4 Hz, 1H), 7.61 (dd, *J* = 7.8, 1.6 Hz, 1H), 7.32 (d, *J* = 8.8 Hz, 1H), 7.28 – 7.23 (m, 1H), 7.21 – 7.12 (m, 2H), 6.71 (s, 1H), 6.62 (dd, *J* = 8.8, 0.6 Hz, 1H), 4.21-4.09 (m, 2H), 1.15 (t, *J* = 7.2 Hz, 3H). ^{13}C NMR (150 MHz, $CDCl_3$) δ 169.7, 161.6, 154.2, 137.9, 135.9, 133.4, 130.4, 128.9, 127.6, 126.9, 124.2, 119.1, 119.0, 113.6, 94.5, 74.2, 61.1, 14.0. HRMS (ESI) calcd for $C_{18}H_{14}{}^{79}Br_2NaO_4^+$ [M + Na$^+$] 474.9151, found 474.9151.

Ethyl 4-oxo-2-(pyridin-2-yl)chromane-3-carboxylate (1r)

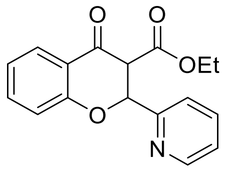

Following the general procedure, **1r** was purified by silica gel chromatography (5% EtOAc/PE). Yield: 480 mg, 75%, a white solid, mp. 86 – 88 ºC. ^{1}H NMR (600 MHz, $CDCl_3$) δ 8.60 (t, *J* = 5.8 Hz, 1H), 7.92 (d, *J* = 7.8 Hz, 1H), 7.73 (t, *J* = 7.6 Hz, 1H), 7.60 – 7.49 (m, 2H), 7.31 – 7.22 (m, 1H), 7.05 (dd, *J* = 7.8, 3.6 Hz, 2H), 5.91 (d, *J* = 10.4 Hz, 1H), 4.51 (d, *J* = 10.2 Hz, 1H), 4.24 – 4.14 (m, 2H), 1.18 (t, *J* = 7.2 Hz, 3H). ^{13}C NMR (151 MHz, $CDCl_3$) δ 187.7, 167.5, 160.4, 149.4, 137.0, 136.6, 133.2, 127.4, 124.4, 123.9, 122.9, 122.0, 118.1, 80.8, 61.5, 56.6, 14.0. HRMS (ESI) calcd for $C_{17}H_{15}NNaO_4^+$ [M + Na$^+$] 320.0893, found 320.0893.

Ethyl 2-(furan-2-yl)-4-oxochromane-3-carboxylate (1s)

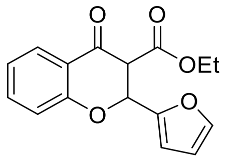

Following the general procedure, **1s** was purified by silica gel chromatography (5% EtOAc/PE). Yield: 432 mg, 70%, colorless oil. Major isomer: ^{1}H NMR (600 MHz, $CDCl_3$) δ 12.31 (s, 1H), 7.70 (dd, *J* = 7.8, 1.6 Hz, 1H), 7.36 (d, *J* = 0.8 Hz, 1H), 7.29

(td, J = 8.2, 1.6 Hz, 1H), 7.08 (t, J = 7.6 Hz, 1H), 6.98 (t, J = 7.6 Hz, 1H), 6.84 (d, J = 8.2 Hz, 1H), 6.26 – 6.21 (m, 2H), 4.24 – 4.17 (m, 2H), 1.25 (t, J = 7.2 Hz, 3H). ^{13}C NMR (150 MHz, $CDCl_3$) δ 187.2, 166.7, 155.5, 149.3, 143.2, 133.3, 124.4, 121.5, 118.1, 117.1, 110.2, 109.4, 68.1, 60.9, 56.3, 14.2. Minor isomer: 7.98 – 7.91 (m, 1H), 7.57 – 7.50 (m, 1H), 7.48 (d, J = 1.0 Hz, 1H), 7.03 (d, J = 8.4 Hz, 1H), 6.51 (dd, J = 7.4, 3.3 Hz, 1H), 6.39 (dd, J = 3.2, 1.8 Hz, 1H), 6.20 (d, J = 3.3 Hz, 1H), 5.79 (d, J = 11.1 Hz, 1H), 4.27-4.24 (m, 2H), 1.19 (t, J = 7.1 Hz, 3H). ^{13}C NMR (150 MHz, $CDCl_3$) δ 187.2, 166.7, 155.5, 149.3, 143.2, 133.3, 124.4, 121.5, 118.1, 117.1, 110.2, 109.4, 68.1, 60.9, 56.4, 14.2. HRMS (ESI) calcd for $C_{16}H_{14}NaO_5^+$ [M + Na$^+$] 309.0733, found 309.0733.

Ethyl 4-oxo-2-(thiophen-2-yl)chromane-3-carboxylate (1t)

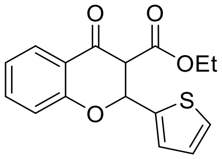

Following the general procedure, **1t** was purified by silica gel chromatography (5% EtOAc/PE). Yield: 507 mg, 78%, a white solid, mp. 70 – 72 °C. ^{1}H NMR (600 MHz, $CDCl_3$) δ 12.29 (s, 1H), 7.69 (dd, J = 7.8, 1.5 Hz, 1H), 7.35 – 7.27 (m, 1H), 7.17 (dd, J = 5.0, 1.2 Hz, 1H), 7.01 – 6.90 (m, 2H), 6.90 – 6.83 (m, 2H), 6.45 (s, 1H), 4.27 (m, 2H), 1.26 (t, J = 7.2 Hz, 3H). ^{13}C NMR (150 MHz, $CDCl_3$) δ 155.4, 144.4, 133.4, 126.5, 125.9, 125.8, 124.5, 121.7, 117.8, 117.5, 100.0, 95.5, 70.5, 60.9, 14.2. HRMS (ESI) calcd for $C_{16}H_{14}NaO_4S^+$ [M + Na$^+$] 325.0505, found 325.0505.

Ethyl 2-benzyl-4-oxochromane-3-carboxylate (1u)

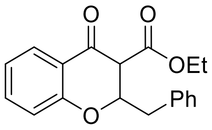

Following the general procedure, **1u** was purified by silica gel chromatography (5% EtOAc/PE). Yield: 526 mg, 79%, colorless oil. ^{1}H NMR (600 MHz, $CDCl_3$) δ 12.2 (s,

1H), 7.68 (d, *J* = 7.7 Hz, 1H), 7.36 -7.32 (m, 1H), 7.28 (t, *J* = 7.6 Hz, 2H), 7.21 (t, *J* = 7.2 Hz, 1H), 7.14 (d, *J* = 7.6 Hz, 2H), 6.99 (t, *J* = 7.4 Hz, 1H), 6.87 (d, *J* = 8.2 Hz, 1H), 5.36 – 5.34 (m, 1H), 4.16 (m, 2H), 3.02 (m, 1H), 2.81 (dd, *J* = 13.8, 3.8 Hz, 1H), 1.26 (t, *J* = 7.2 Hz, 3H). ^{13}C NMR (150 MHz, $CDCl_3$) δ 170.1, 162.2, 155.5, 137.6, 133.3, 129.5, 128.3, 126.5, 124.5, 121.3, 117.9, 117.5, 95.7, 75.1, 60.8, 41.2, 14.2. HRMS (ESI) calcd for $C_{19}H_{18}NaO_4^+$ [M + Na$^+$] 333.1097, found 333.1098.

Ethyl 4-oxo-2-propylchromane-3-carboxylate (1v)

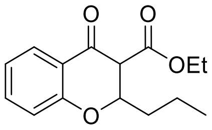

Following the general procedure, **1v** was purified by silica gel chromatography (5% EtOAc/PE). Yield: 445 mg, 78%, colorless oil. ^{1}H NMR (600 MHz, $CDCl_3$) δ 12.11 (s, 1H), 7.89 (d, *J* = 7.8 Hz, 1H), 7.64 (d, *J* = 7.8 Hz, 1H), 7.49 (t, *J* = 7.4 Hz, 1H), 7.35 – 7.28 (m, 1H), 7.09 – 7.00 (m, 1H), 6.99 – 6.93 (m, 2H), 6.86 (d, *J* = 8.2 Hz, 1H), 5.20 (dd, *J* = 9.3, 3.3 Hz, 1H), 4.80 – 4.67 (m, 1H), 4.35 – 4.22 (m, 4H), 3.63 (d, *J* = 11.2 Hz, 1H), 1.88 – 1.76 (m, 2H), 1.70 – 1.60 (m, 2H), 1.55 – 1.41 (m, 4H), 1.37 – 1.28 (m, 6H), 0.98 (t, *J* = 7.2 Hz, 3H), 0.91 (t, *J* = 7.2 Hz, 3H). ^{13}C NMR (150 MHz, $CDCl_3$) δ 188.2, 167.8, 161.0, 155.7, 136.4, 133.0, 127.6, 127.3, 124.4, 121.6, 121.0, 120.0, 117.9, 117.2, 96.3, 78.7, 73.7, 61.6, 60.6, 58.3, 37.1, 35.4, 18.6, 18.1, 14.3, 14.2, 13.8, 13.7. HRMS (ESI) calcd for $C_{15}H_{18}NaO_4^+$ [M + Na$^+$] 285.1097, found 285.1097.

3-Acetyl-2-phenylchroman-4-one (1w)

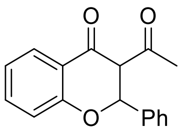

Following the general procedure, **1w** was purified by silica gel chromatography (5% EtOAc/PE). Yield: 376 mg, 65%, a yellow solid, mp. 102 – 104 ^{o}C. ^{1}H NMR (600 MHz, $CDCl_3$) δ 16.41 (s, 1H), 7.91 – 7.78 (m, 1H), 7.45 (d, *J* = 7.4 Hz, 2H), 7.40 – 7.27 (m,

4H), 6.98 (t, *J* = 7.6 Hz, 1H), 6.87 (d, *J* = 8.4 Hz, 1H), 6.29 (s, 1H), 2.08 (s, 3H). ^{13}C NMR (150 MHz, $CDCl_3$) δ 190.3, 175.5, 157.1, 139.5, 135.0, 128.8, 128.7, 127.5, 125.9, 121.7, 119.6, 117.8, 104.3, 76.4, 22.8. HRMS (ESI) calcd for $C_{17}H_{14}NaO_3^+$ [M + Na^+] 289.0835, found 289.0835.

4-Oxo-2-phenylchromane-3-carbonitrile (1x)

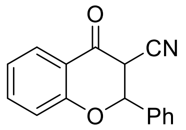

Following the general procedure, **1x** was purified by silica gel chromatography (5% EtOAc/PE). Yield: 353 mg, 65%, a yellow solid, mp. 114 – 116 °C. ^1H NMR (600 MHz, $CDCl_3$) δ 7.95 (d, *J* = 7.9 Hz, 1H), 7.64 – 7.53 (m, 3H), 7.51 – 7.42 (m, 3H), 7.14 (t, *J* = 7.6 Hz, 1H), 7.06 (d, *J* = 8.4 Hz, 1H), 5.46 (d, *J* = 12.0 Hz, 1H), 4.16 (d, *J* = 12.0 Hz, 1H). ^{13}C NMR (150 MHz, $CDCl_3$) δ 182.7, 160.8, 137.6, 135.2, 130.2, 129.2, 128.0, 127.0, 122.9, 118.8, 118.4, 113.2, 80.9, 47.3. HRMS (ESI) calcd for $C_{16}H_{11}NNaO_2^+$ [M + Na^+] 272.0682, found 272.0672.

Flavanone (1y)

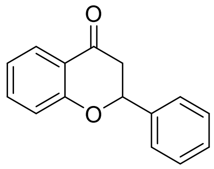

Following the general procedure, **1y** was purified by silica gel chromatography (5% EtOAc/PE). Yield: 304 mg, 68%, a white solid, mp. 76 – 78 °C. ^1H NMR (600 MHz, $CDCl_3$) δ 7.92 (dd, *J* = 8.0, 1.4 Hz, 1H), 7.52 – 7.44 (m, 3H), 7.41 (t, *J* = 7.4 Hz, 2H), 7.35 (t, *J* = 4.8 Hz, 1H), 7.02 (dd, *J* = 11.8, 4.4 Hz, 2H), 5.44 (d, *J* = 13.2 Hz, 1H), 3.04 (dd, *J* = 16.6, 13.4, Hz, 1H), 2.90 – 2.81 (m, 1H). 13C NMR (150 MHz, CDCl3) δ 191.8, 161.6, 138.9, 136.2, 128.9, 128.8, 127.1, 126.2, 121.7, 121.0, 118.2, 79.6, 44.7. HRMS (ESI) calcd for $C_{15}H_{12}NaO_2^+$ [M + Na^+] 247.0730, found 247.0732.

4-Chromanone (1z)

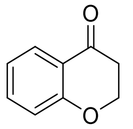

Following the general procedure, **1z** was purified by silica gel chromatography (5% EtOAc/PE). Yield: 230 mg, 78%, a white solid, mp. 37 – 39 °C. 1H NMR (600 MHz, $CDCl_3$) δ 7.85 (dd, *J* = 8.0, 1.8 Hz, 1H), 7.58 – 7.22 (m, 1H), 6.98 – 6.95 (m, 1H), 6.93 (d, *J* = 8.4 Hz, 1H), 4.71 – 4.34 (m, 1H), 3.08 – 2.59 (m, 1H). ^{13}C NMR (150 MHz, CDCl3) δ 191.5, 161.8, 135.8, 127.0, 121.2, 117.8, 66.9, 37.7. HRMS (ESI) calcd for $C_9H_8NaO_2^+$ [M + Na+] 171.0417, found 171.0417.

Methyl 4-oxochromane-3-carboxylate (1aa)[5]

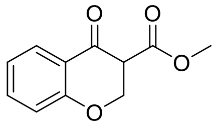

To a solution of 4-chromanone (5 mmol, 1.0 equiv) in THF (10 mL) stirred at −78 °C was added a solution of LiHMDS (1 M in THF, 7.5 mL, 7.5 mmol, 1.5 equiv) dropwise and the mixture was stirred for 30 minutes. Methyl cyanoformate (10 mmol, 2.0 equiv) in THF (10 mL) was added dropwise and the reaction was stirred at −78 °C for 1 hour. The mixture was allowed to warm to room temperature and was quenched by the addition of aq. NH_4Cl (25 mL) and H_2O (25 mL). The mixture was extracted with EtOAc (3 × 25 mL). The combined organic phases were washed with brine (30 mL), dried ($MgSO_4$) and concentrated in vacuo. Flash column chromatography (ethyl acetate/ petroleum ether = 1:15) afforded the product as a colorness oil.

Following the procedure, **1aa** was purified by silica gel chromatography (10% EtOAc/PE). Yield: 350 mg, 85%, a colorless oil. Major: ^{1}H NMR (600 MHz, $CDCl_3$) δ 7.95 – 7.85 (m, 1H), 7.53 – 7.43 (m, 1H), 7.07 – 6.92 (m, 2H), 4.97 – 4.90 (m, 1H), 4.82 – 4.74 (m, 1H), 4.62 – 4.58 (m, 1H), 3.79 – 3.72 (m, 3H). ^{13}C NMR (150 MHz, CDCl3) δ 186.8, 167.6, 161.4, 136.4, 127.6, 121.8, 117.9, 116.4, 68.1, 52.5, 51.6. Minor: ^{1}H NMR (600 MHz, $CDCl_3$) δ 11.93 (s, 1H), 7.64 (d, *J* = 7.2 Hz, 1H), 7.34 – 7.29 (m, 1H), 6.98 (d, *J* = 10.8, 1H), 6.85 (d, *J* = 8.0 Hz, 1H), 4.97 – 4.90 (m, 2H), 3.83

– 3.78 (m, 3H). ^{13}C NMR (150 MHz, $CDCl_3$) δ 170.0, 162.8, 157.7, 133.1, 124.5, 121.4, 120.5, 118.2, 91.7, 63.7, 52.7. HRMS (ESI) calcd for $C_{11}H_{10}NaO_4^+$ [M + Na$^+$] 229.0471, found 229.0473.

3.2 Spectral data of products

Ethyl 4-oxo-2-phenyl-4*H*-chromene-3-carboxylate (2a)

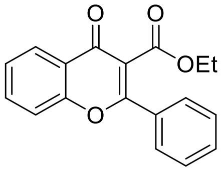

Following the general procedure, **2a** was purified by silica gel chromatography (15% EtOAc/PE). Yield: 282 mg, 89%, a white solid, mp. 81 – 82 °C. ^{1}H NMR (600 MHz, $CDCl_3$) δ 8.27 (dd, *J* = 8.0, 1.6 Hz, 1H), 7.76 (d, *J*= 6.6 Hz, 2H), 7.73 (td, *J* = 8.4, 1.8 Hz, 1H). 7.56 (t, *J* = 7.2 Hz, 1H), 7.54-7.50 (m, 3H), 7.45 (t, *J* = 7.2 Hz, 1H), 4.28 (q, *J* = 7.2 Hz, 2H) 1.17 (t, *J* = 7.2 Hz, 3H). ^{13}C NMR (150 MHz, $CDCl_3$) δ 175.1, 165.1, 163.1, 155.9, 134.3, 132.0, 131.6, 128.8, 128.1, 126.2, 125.7, 123.2, 118.4, 118.1, 61.9, 13.9. HRMS (ESI) calcd for $C_{18}H_{14}NaO_4^+$ [M + Na$^+$] 317.0784, found 317.0784.

Ethyl 6-fluoro-4-oxo-2-phenyl-4*H*-chromene-3-carboxylate (2b)

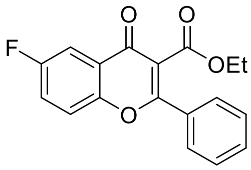

Following the general procedure, **2b** was purified by silica gel chromatography (3% EtOAc/PE). Yield: 244 mg, 73%, a white solid, mp. 100 – 102 °C. ^{1}H NMR (600 MHz, $CDCl_3$) δ 7.90 (dd, *J* = 8.0, 3.2 Hz, 1H), 7.74 (dd, *J* = 5.4, 3.4 Hz, 2H), 7.60 – 7.54 (m, 2H), 7.52 (t, *J* = 7.6 Hz, 2H), 7.45 (m, 3.1 Hz, 1H), 4.27 (q, *J* = 7.2 Hz, 2H), 1.17 (t, *J* = 7.2 Hz, 3H). ^{13}C NMR (150 MHz, $CDCl_3$) δ 174.3, 164.1 ($^1J_{C\text{-}F}$ = 211.83), 160.6, 159.0, 152.1, 131.8, 131.8, 128.8, 128.1, 124.5($^3J_{C\text{-}F}$ = 7.635), 122.7($^2J_{C\text{-}F}$ = 25.335),

120.3($^{3}J_{C-F}$ = 7.995), 117.7, 111.1($^{2}J_{C-F}$ = 23.73), 62.0, 13.8. HRMS (ESI) calcd for $C_{18}H_{13}FNaO_4^+$ [M + Na$^+$] 335.0690, found 335.0690.

Ethyl 6-bromo-4-oxo-2-phenyl-4*H*-chromene-3-carboxylate (2c)

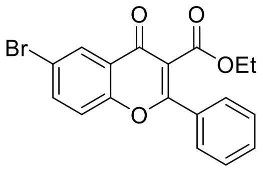

Following the general procedure, **2c** was purified by silica gel chromatography (20% EtOAc/PE). Yield: 328 mg, 83%, a white solid, mp. 114 – 116 °C. ^{1}H NMR (600 MHz, $CDCl_3$) δ 8.38 (s, 1H), 7.80 (d, *J* = 8.8 Hz, 1H), 7.74 (d, *J* = 7.5 Hz, 2H), 7.57 (t, *J* = 7.4 Hz, 1H), 7.51 (t, *J* = 7.4 Hz, 2H), 7.43 (d, *J* = 8.8 Hz, 1H), 4.27 (q, *J* = 7.0 Hz, 2H), 1.16 (t, *J* = 7.2 Hz, 3H). ^{13}C NMR (150 MHz, $CDCl_3$) δ 173.7, 164.7, 163.4, 154.6, 137.3, 131.9, 131.7, 128.8, 128.7, 128.1, 124.5, 120.1, 119.2, 118.4, 62.1, 13.8. HRMS (ESI) calcd for $C_{18}H_{13}{}^{79}BrNaO_4^+$ [M + Na$^+$] 394.9889, found 394.9889.

Ethyl 6-chloro-4-oxo-2-phenyl-4H-chromene-3-carboxylate (2d)

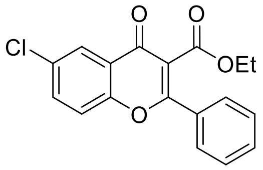

Following the general procedure, **2d** was purified by silica gel chromatography (40% DCM/PE). Yield: 263 mg, 75%, a white solid, mp. 106 – 108 °C. ^{1}H NMR (600 MHz, $CDCl_3$) δ 8.38 (s, 1H), 7.80 (d, *J* = 8.8 Hz, 1H), 7.74 (d, *J* = 7.6 Hz, 2H), 7.57 (t, *J* = 7.4 Hz, 1H), 7.51 (t, *J* = 7.4 Hz, 2H), 7.43 (d, *J* = 8.8 Hz, 1H), 4.27 (q, *J* = 7.2 Hz, 2H), 1.16 (t, *J* = 7.2 Hz, 3H). ^{13}C NMR (150 MHz, $CDCl_3$) δ 173.7, 164.7, 163.4, 154.6, 137.3, 131.9, 131.7, 128.8, 128.7, 128.1, 124.5, 120.1, 119.2, 118.4, 62.1, 13.8. HRMS (ESI) calcd for $C_{18}H_{13}{}^{35}ClNaO_4^+$ [M + Na$^+$] 351.0395, found 351.0395.

Ethyl 6-cyano-4-oxo-2-phenyl-4*H*-chromene-3-carboxylate (2e)

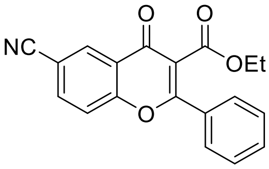

Following the general procedure, **2e** was purified by silica gel chromatography (20% EtOAc/PE). Yield: 250 mg, 73%, a white solid, mp. 148 – 150 °C. ^{1}H NMR (600 MHz, $CDCl_3$) δ 8.59 (s, 1H), 7.95 (dd, *J* = 8.8, 1.8 Hz, 1H), 7.75 (d, *J* = 7.7 Hz, 2H), 7.66 (d, *J* = 8.8 Hz, 1H), 7.60 (t, *J* = 7.4 Hz, 1H), 7.53 (t, *J* = 7.8 Hz, 2H), 4.28 (q, *J* = 7.2 Hz, 2H), 1.17 (t, *J* = 7.2 Hz, 3H). ^{13}C NMR (150 MHz, $CDCl_3$) δ 173.2, 164.1, 163.7, 157.5, 136.6, 132.2, 131.7, 131.1, 129.0, 128.1, 123.7, 119.9, 119.0, 117.3, 110.0, 62.3, 13.9. HRMS (ESI) calcd for $C_{19}H_{13}NNaO_4^+$ [M + Na$^+$] 342.0737, found 342.0738.

Ethyl 7-nitro-4-oxo-2-phenyl-4*H*-chromene-3-carboxylate (2f)

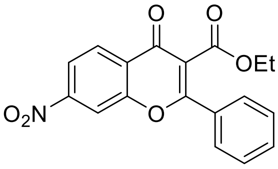

Following the general procedure, **2f** was purified by silica gel chromatography (5% DCM/PE). Yield: 246 mg, 68%, a white solid, mp. 186 – 188 °C. ^{1}H NMR (600 MHz, $CDCl_3$) δ 9.13 (d, *J* = 3.0 Hz, 1H), 8.56 (dd, *J* = 9.2, 2.8 Hz, 1H), 7.77 (d, *J* = 7.2 Hz, 2H), 7.71 (d, *J* = 9.2 Hz, 1H), 7.62 (t, *J* = 7.4 Hz, 1H), 7.55 (t, *J* = 7.6 Hz, 2H), 4.30 (q, *J* = 7.2 Hz, 2H), 1.18 (t, *J* = 7.2 Hz, 3H). ^{13}C NMR (151 MHz, $CDCl_3$) δ 173.6, 164.1, 163.7, 158.6, 145.1, 132.3, 131.0, 129.0, 128.7, 128.2, 123.4, 122.8, 120.0, 118.7, 62.3, 13.8. HRMS (ESI) calcd for $C_{18}H_{13}NNaO_6^+$ [M + Na$^+$] 362.0635, found 362.0635.

Ethyl 8-chloro-4-oxo-2-phenyl-4*H*-chromene-3-carboxylate (2g)

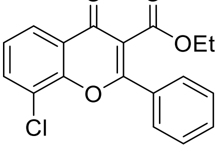

Following the general procedure, **2g** was purified by silica gel chromatography (20% EtOAc/PE). Yield: 197 mg, 56%, a white solid, mp. 108 – 110 ºC. ^{1}H NMR (600 MHz, $CDCl_3$) δ 8.22 – 8.12 (m, 1H), 7.84 (dd, *J* = 7.2, 1.8 Hz, 2H), 7.81 – 7.72 (m, 1H), 7.62 – 7.56 (m, 1H), 7.55 – 7.50 (m, 2H), 7.39 (m, 1H), 4.31 (q, *J* = 7.2, 2H), 1.20 (td, *J* = 7.2, 1.8 Hz, 3H). ^{13}C NMR (150 MHz, $CDCl_3$) δ 174.6, 164.8, 162.7, 151.6, 134.5, 132.0, 131.4, 128.9, 128.3, 125.7, 124.7, 124.6, 123.3, 118.2, 62.1, 13.9. HRMS (ESI) calcd for $C_{18}H_{13}{}^{35}ClNaO_4^+$ [M + Na$^+$] 351.0395, found 351.0397.

Ethyl 6-methyl-4-oxo-2-phenyl-4*H*-chromene-3-carboxylate (2h)

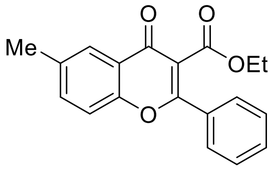

Following the general procedure, **2h** was purified by silica gel chromatography (15% EtOAc/PE). Yield: 268 mg, 81%, a white solid, mp. 114 – 116 ºC. ^{1}H NMR (600 MHz, $CDCl_3$) δ 8.03 (s, 1H), 7.74 (d, *J* = 7.8 Hz, 2H), 7.58 – 7.46 (m, 4H), 7.42 (dd, *J* = 8.6, 2.9 Hz, 1H), 4.27 (q, *J* = 7.2 Hz, 2H), 2.46 (s, 3H), 1.16 (t, *J* = 7.2 Hz, 3H). ^{13}C NMR (150 MHz, $CDCl_3$) δ 175.1, 165.2, 162.9, 154.2, 135.8, 135.5, 132.2, 131.5, 128.7, 128.1, 125.4, 122.9, 118.2, 117.8, 61.8, 21.0, 13.8. HRMS (ESI) calcd for $C_{19}H_{16}NaO_4^+$ [M + Na$^+$] 331.0941, found 331.0941.

Ethyl 7-methoxy-4-oxo-2-phenyl-4*H*-chromene-3-carboxylate (2i)

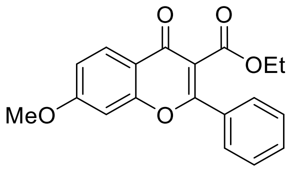

Following the general procedure, **2i** was purified by silica gel chromatography (15% EtOAc/PE). Yield: 298 mg, 92%, a white solid, mp. 100 – 102 ºC. 8.16 (d, *J* = 8.9 Hz, 1H), 7.75 – 7.72 (m, 2H), 7.59 – 7.53 (m, 1H), 7.50 (t, *J* = 7.5 Hz, 2H), 7.01 (dd, *J* = 8.9, 2.3 Hz, 1H), 6.91 (d, *J* = 2.3 Hz, 1H), 4.27 (q, *J* = 7.1 Hz, 2H), 3.92 (s, 3H), 1.17

(t, J = 7.1 Hz, 3H). ^{13}C NMR (150 MHz, $CDCl_3$) δ 174.4, 165.2, 164.6, 162.6, 157.6, 132.1, 131.5, 128.7, 128.1, 127.6, 118.3, 117.0, 115.0, 100.3, 61.9, 55.9, 13.9. HRMS (ESI) calcd for $C_{19}H_{16}NaO_5^+$ [M + Na$^+$] 347.0890, found 347.0891.

Ethyl 1-oxo-3-phenyl-1*H*-benzo[*f*]chromene-2-carboxylate (2j)

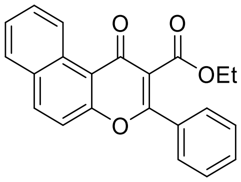

Following the general procedure, **2j** was purified by silica gel chromatography (15% EtOAc/PE). Yield: 213 mg, 62%, a white solid, mp. 136 – 138 °C. ^{1}H NMR (600 MHz, $CDCl_3$) δ 10.04 – 10.02 (m, 1H), 8.15 – 8.11 (m, 1H), 7.92 (t, J = 6.6 Hz, 1H), 7.83 – 7.80 (m, 2H), 7.78-7.74 (m, 1H), 7.68 – 7.61 (m, 1H), 7.56 -7.51 (m, 4H), 4.35 (q, J = 7.2 Hz, 2H), 1.24 (t, J = 7.2 Hz, 3H). ^{13}C NMR (150 MHz, $CDCl_3$) δ 176.8, 165.4, 160.1, 157.1, 136.2, 131.5, 131.5, 130.7, 130.3, 129.5, 128.8, 128.3, 128.0, 127.2, 126.9, 120.9, 117.3, 116.5, 62.0, 14.0. HRMS (ESI) calcd for $C_{22}H_{16}NaO_4^+$ [M + Na$^+$] 367.0941, found 367.0951.

Ethyl 2-(2-chlorophenyl)-4-oxo-4*H*-chromene-3-carboxylate (2k)

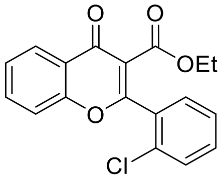

Following the general procedure, **2k** was purified by silica gel chromatography (30% DCM/PE). Yield: 284 mg, 81%, a white solid, mp. 132 – 134 °C. ^{1}H NMR (400 MHz, $CDCl_3$) δ 8.30 (dd, J = 8.0, 1.4 Hz, 1H), 7.77 – 7.68 (m, 1H), 7.57 – 7.44 (m, 5H), 7.38 (td, J = 7.4, 1.2 Hz, 1H), 4.13 (q, J = 7.2 Hz, 2H), 1.01 (t, J = 7.2 Hz, 3H). ^{13}C NMR (100 MHz, $CDCl_3$) δ 174.5, 163.6, 163.1, 155.9, 134.4, 133.3, 132.0, 131.6, 130.5, 130.0, 126.7, 126.3, 125.9, 123.7, 120.0, 118.2, 61.5, 13.7. HRMS (ESI) calcd for $C_{18}H_{13}{}^{35}ClNaO_4^+$ [M + Na$^+$] 351.0395, found 351.0395.

Ethyl 2-(2- bromophenyl)-4-oxo-4*H*-chromene-3-carboxylate (2l)

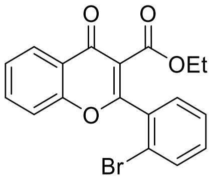

Following the general procedure, **2l** was purified by silica gel chromatography (15% EtOAc/PE). Yield: 327 mg, 83%, a white solid, mp. 134 – 136 ºC. ^{1}H NMR (600 MHz, $CDCl_3$) δ 8.29 (d, *J* = 7.9 Hz, 1H), 7.79 – 7.65 (m, 2H), 7.51 – 7.37 (m, 5H), 4.11 (q, *J* = 7.1 Hz, 2H), 0.99 (t, *J* = 7.1 Hz, 3H). ^{13}C NMR (150 MHz, $CDCl_3$) δ 174.5, 164.0, 163.5, 155.9, 134.5, 133.6, 133.2, 132.1, 130.6, 127.3, 126.2, 125.9, 123.6, 122.3, 119.7, 118.2, 61.4, 13.7. HRMS (ESI) calcd for $C_{18}H_{13}{}^{79}BrNaO_4^+$ [M + Na^+] 394.9889, found 394.9889.

Ethyl 2-(4-fluorophenyl)-4-oxo-4*H*-chromene-3-carboxylate (2m)

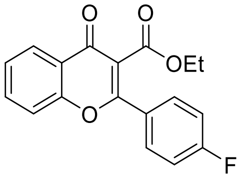

Following the general procedure, **2m** was purified by silica gel chromatography (15% EtOAc/PE). Yield: 283 mg, 91%, colorless oil. ^{1}H NMR (600 MHz, $CDCl_3$) δ 8.10 – 8.07 (m, 1H), 7.68 - 7.62 (m, 2H), 7.61 – 7.53 (m, 1H), 7.42 – 7.34 (m, 1H), 7.32 – 7.25 (m, 1H), 7.16 – 6.99 (m, 2H), 4.19 - 4.15 (m, 2H), 1.37 – 0.83 (m, 3H). ^{13}C NMR (150 MHz, $CDCl_3$) δ 174.8, 164.9, 164.5 ($^1J_{C\text{-}F}$ = 251.955), 161.7, 155.7, 134.4, 130.4 ($^3J_{C\text{-}F}$ = 8.82), 128.1, 125.9, 125.7, 123.0, 118.3, 118.0, 116.0 ($^2J_{C\text{-}F}$ = 21.96), 61.9, 13.9. HRMS (ESI) calcd for $C_{18}H_{13}FNaO_4^+$ [M + Na^+] 335.0690, found 335.0690.

Ethyl 4-oxo-2-(*p*-tolyl)-4*H*-chromene-3-carboxylate (2n)

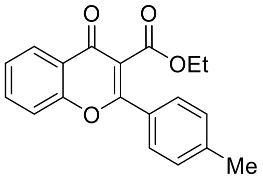

Following the general procedure, **2n** was purified by silica gel chromatography (15% EtOAc/PE). Yield: 225 mg, 68%, colorless oil. ^{1}H NMR (600 MHz, $CDCl_3$) δ 8.32 – 8.16 (m, 1H), 7.70 – 7.58 (m, 3H), 7.49 -7.46 (m, 1H), 7.43 – 7.35 (m, 1H), 7.29 – 7.27 (d, 2H), 4.29 (q, *J* = 6.6 Hz, 2H), 2.41 (s, 3H), 1.44 – 0.92 (m, 3H). ^{13}C NMR (151 MHz, $CDCl_3$) δ 175.0, 165.3, 163.0, 155.8, 142.3, 134.2, 129.5, 129.1, 128.0, 126.0, 125.6, 123.1, 118.1, 117.9, 61.8, 21.6, 13.9. HRMS (ESI) calcd for $C_{19}H_{16}NaO_4^+$ [M + Na$^+$] 331.0941, found 331.0941.

Ethyl 2-(naphthalen-2-yl)-4-oxo-4*H*-chromene-3-carboxylate (2o)

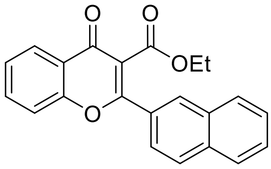

Following the general procedure, **2o** was purified by silica gel chromatography (15% EtOAc/PE). Yield: 275 mg, 75%, a white solid, mp. 112 – 114 °C. ^{1}H NMR (400 MHz, $CDCl_3$) δ 8.35 (dd, *J* = 7.8, 1.6 Hz, 1H), 8.02 (d, *J* = 8.3 Hz, 1H), 7.93 (dd, *J* = 6.4, 3.2 Hz, 1H), 7.89 – 7.83 (m, 1H), 7.76 – 7.66 (m, 2H), 7.58 – 7.45 (m, 5H), 3.95 (q, *J* = 7.2 Hz, 2H), 0.73 (t, *J* = 7.2 Hz, 3H). ^{13}C NMR (100 MHz, $CDCl_3$) δ 174.7, 164.8, 164.0, 156.1, 134.4, 133.4, 131.5, 130.6, 129.7, 128.5, 127.8, 127.5, 126.7, 126.3, 125.9, 124.9, 124.8, 123.7, 120.8, 118.3, 61.3, 13.5. HRMS (ESI) calcd for $C_{22}H_{16}NaO_4^+$ [M + Na$^+$] 367.0941, found 367.0941.

Ethyl 2-(2-methoxyphenyl)-4-oxo-4*H*-chromene-3-carboxylate (2p)

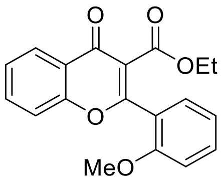

Following the general procedure, **2p** was purified by silica gel chromatography (15% EtOAc/PE). Yield: 260 mg, 75%, a white solid, mp. 120 – 122 °C. ^{1}H NMR (600 MHz, $CDCl_3$) δ 8.28 (dd, *J* = 7.8 Hz, 1.6 Hz, 1H), 7.69 (t, *J* = 9.0 Hz, 1H), 7.52-7.49 (m, 2H), 7.47 (d, *J* = 8.4 Hz, 1H), 7.43 (t, *J* = 7.8 Hz, 1H), 7.07 (t, *J* = 7.8 Hz, 1H), 6.99 (d, *J* =

7.8 Hz, 1H), 4.15 (q, *J* = 7.2 Hz, 2H), 3.83 (s, 3H), 1.06 (t, *J* = 7.2 Hz, 3H). ^{13}C NMR (150 MHz, $CDCl_3$): δ 174.7, 164.3, 163.4, 157.0, 156.0, 134.0, 132.6, 130.1, 126.2, 125.5, 123.6, 121.7, 120.5, 119.4, 118.1, 111.1, 61.2, 55.6, 13.8. HRMS (ESI) calcd for $C_{19}H_{16}NaO_5^+$ [M + Na$^+$] 347.0890, found 347.0891.

Ethyl 6-bromo-2-(2-bromophenyl)-4-oxo-4*H*-chromene-3-carboxylate (2q)

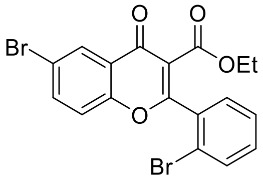

Following the general procedure, **2q** was purified by silica gel chromatography (15% EtOAc/PE). Yield: 360 mg, 80%, a light yellow solid, decomposition > 300 °C. ^{1}H NMR (600 MHz, $CDCl_3$) δ 8.41 (d, *J* = 2.4 Hz, 1H), 7.81 (dd, *J* = 8.8, 2.4 Hz, 1H), 7.72 (dd, *J* = 7.6, 1.4 Hz, 1H), 7.49 (dd, *J* = 7.4, 1.8 Hz, 1H), 7.47 – 7.37 (m, 3H), 4.11 (q, *J* = 7.2 Hz, 2H), 0.99 (t, *J* = 7.2 Hz, 3H). ^{13}C NMR (150 MHz, $CDCl_3$) δ 173.3, 164.2, 163.1, 154.6, 137.4, 133.3, 133.2, 132.2, 130.6, 128.8, 127.3, 125.0, 122.3, 120.2, 119.8, 119.5, 61.6, 13.7. HRMS (ESI) calcd for $C_{18}H_{12}{}^{79}Br_2NaO_4^+$ [M + Na$^+$] 472.8995, found 472.8996.

Ethyl 4-oxo-2-(pyridin-2-yl)-4*H*-chromene-3-carboxylate (2r)

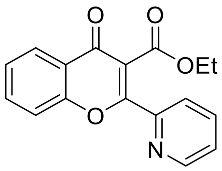

Following the general procedure, **2r** was purified by silica gel chromatography (15% EtOAc/PE). Yield: 251 mg, 79%, a white solid, mp. 142 – 144 °C. ^{1}H NMR (600 MHz, $CDCl_3$) δ 8.65 (s, 1H), 8.23 (t, *J* = 7.0 Hz, 1H), 8.11 - 8.09 (m, 1H), 7.94 – 7.81 (m, 1H), 7.81 – 7.67 (m, 1H), 7.66 – 7.53 (m, 1H), 7.51 - 7.40 (m, 2H), 4.47 (qd, *J* = 7.6, 2.8 Hz, 2H), 1.37 (td, *J* = 7.2, 2.2 Hz, 3H). ^{13}C NMR (150 MHz, $CDCl_3$) δ 175.6, 165.1, 158.1, 155.3, 149.1, 148.8, 137.1, 134.5, 126.1, 125.8, 125.7, 123.5, 122.2, 118.2,

118.0, 61.6, 14.1. HRMS (ESI) calcd for $C_{17}H_{13}NNa4^+$ [M + Na^+] 318.0737, found 318.0737.

Ethyl 2-(furan-2-yl)-4-oxo-4*H*-chromene-3-carboxylate (2s)

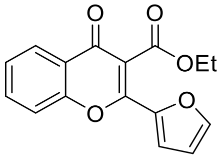

Following the general procedure, **2s** was purified by silica gel chromatography (15% EtOAc/PE). Yield: 240 mg, 78%, a white solid, mp. 88 – 90 °C. ^{1}H NMR (600 MHz, $CDCl_3$) δ 8.22 (dd, *J* = 7.8, 1.6 Hz, 1H), 7.72 – 7.67 (m, 1H), 7.63 (d, *J* = 1.0 Hz, 1H), 7.50 (d, *J* = 8.4 Hz, 1H), 7.45 – 7.38 (m, 1H), 7.23 (d, *J* = 3.4 Hz, 1H), 6.64 (dd, *J* = 3.4, 1.8 Hz, 1H), 4.48 (q, *J* = 7.2 Hz, 2H), 1.40 (t, *J* = 7.2 Hz, 3H). ^{13}C NMR (150 MHz, $CDCl_3$) δ 174.9, 164.5, 155.3, 151.6, 146.5, 145.3, 134.3, 126.0, 125.6, 123.3, 117.8, 115.6, 114.8, 112.6, 62.0, 14.2. HRMS (ESI) calcd for $C_{16}H_{12}NaO_5^+$ [M + Na^+] 307.0577, found 307.0577.

Ethyl 4-oxo-2-(thiophen-2-yl)-4*H*-chromene-3-carboxylate (2t)

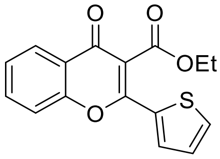

Following the general procedure, **2t** was purified by silica gel chromatography (15% EtOAc/PE). Yield: 252 mg, 78%, a white solid, mp. 88 – 90 °C. ^{1}H NMR (600 MHz, $CDCl_3$) δ 8.21 (d, *J* = 7.8 Hz, 1H), 7.75 – 7.61 (m, 3H), 7.52 (d, *J* = 7.8 Hz, 1H), 7.42 (t, *J* = 8.8 Hz, 1H), 7.18 (t, *J* = 4.8 Hz, 1H), 4.45 (q, *J* = 7.2 Hz, 2H), 1.37 (t, *J* = 7.2 Hz, 3H). ^{13}C NMR (150 MHz, $CDCl_3$) δ 174.9, 165.2, 156.0, 155.5, 134.3, 133.2, 131.6, 130.9, 128.3, 125.9, 125.6, 123.0, 117.9, 116.1, 62.3, 14.0. HRMS (ESI) calcd for $C_{16}H_{12}NaO_4S^+$ [M + Na^+] 323.0349, found 323.0348.

Ethyl 2-benzyl-4-oxo-4*H*-chromene-3-carboxylate (2u)

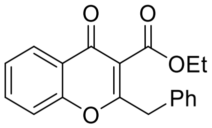

Following the general procedure, **2u** was purified by silica gel chromatography (15% EtOAc/PE). Yield: 222 mg, 67%, colorless oil. ^{1}H NMR (600 MHz, CDCl$_3$) δ 8.17 (d, J = 8.2 Hz, 1H), 7.61 (t, J = 7.8 Hz, 1H), 7.42 – 7.31 (m, 6H), 7.29 – 7.21 (m, 1H), 4.42 (d, J = 7.2 Hz, 2H), 4.08 (s, 2H), 1.38 (t, J = 7.2 Hz, 3H). ^{13}C NMR (151 MHz, CDCl$_3$) δ 174.7, 166.9, 165.1, 155.7, 134.9, 134.0, 129.1, 128.8, 127.4, 126.0, 125.6, 123.3, 118.6, 117.9, 62.0, 39.0, 14.2. HRMS (ESI) calcd for $C_{19}H_{16}NaO_4^+$ [M + Na$^+$] 331.0941, found 331.0941.

Ethyl 4-oxo-2-propyl-4*H*-chromene-3-carboxylate (2v)

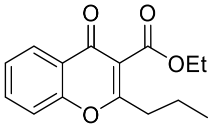

Following the general procedure, **2v** was purified by silica gel chromatography (15% EtOAc/PE). Yield: 212 mg, 75%, colorless oil. ^{1}H NMR (600 MHz, CDCl$_3$) δ 8.20 (d, J = 8.0 Hz, 1H), 7.74 – 7.62 (m, 1H), 7.43 (d, J = 8.4 Hz, 1H), 7.40 (t, J = 7.5 Hz, 1H), 4.42 (d, J = 7.2 Hz, 2H), 2.73 (t, J = 7.6 Hz, 2H), 1.84 (dd, J = 15.0, 7.6 Hz, 2H), 1.40 (t, J = 7.2 Hz, 3H), 1.04 (t, J = 7.4 Hz, 3H). ^{13}C NMR (151 MHz, CDCl$_3$) δ 174.6, 169.2, 165.1, 155.7, 133.9, 126.1, 125.4, 123.4, 118.3, 117.8, 61.7, 34.9, 20.8, 14.2, 13.7. HRMS (ESI) calcd for $C_{15}H_{16}NaO_4^+$ [M + Na$^+$] 283.0941, found 283.0941.

3-Acetyl-2-phenyl-4*H*-chromen-4-one (2w)

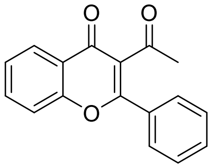

Following the general procedure, **2w** was purified by silica gel chromatography (15% EtOAc/PE). Yield: 193 mg, 73%, colorless oil. ^{1}H NMR (600 MHz, CDCl$_3$) δ 8.25 (dd, J = 8.0, 1.4 Hz, 1H), 7.76 – 7.70 (m, 1H), 7.69 – 7.64 (m, 2H), 7.57 -7.49 (m, 4H), 7.46

(t, J = 7.6 Hz, 1H), 2.46 (s, 3H). ^{13}C NMR (150 MHz, $CDCl_3$) δ 200.5, 175.9, 162.5, 155.9, 134.4, 131.9, 131.6, 128.9, 128.6, 126.0, 125.7, 124.9, 123.5, 118.1, 32.4. HRMS (ESI) calcd for $C_{17}H_{12}NaO_3^+$ [M + Na$^+$] 287.0679, found 287.0679.

4-Oxo-2-phenyl-4*H*-chromene-3-carbonitrile (2x)

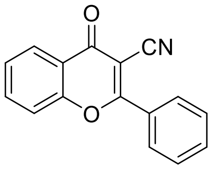

Following the general procedure, **2x** was purified by silica gel chromatography (15% EtOAc/PE). Yield: 112 mg, 45%, a white solid, mp. 150 – 152 °C. ^{1}H NMR (600 MHz, $CDCl_3$) δ 8.28 (d, J = 7.8 Hz, 1H), 8.12 (d, J = 7.8 Hz, 2H), 7.81 (t, J = 7.8 Hz, 1H), 7.68 (t, J = 7.4 Hz, 1H), 7.61 (t, J = 8.8 Hz, 3H), 7.53 (t, J = 7.6 Hz, 1H). ^{13}C NMR (150 MHz, $CDCl_3$) δ 174.2, 170.8, 155.4, 135.3, 133.4, 130.1, 129.2, 128.8, 126.9, 126.2, 122.0, 118.3, 114.0, 98.3. HRMS (ESI) calcd for $C_{16}H_9NNaO_2^+$ [M + Na$^+$] 270.0525, found 270.0526.

2-Phenyl-4*H*-chromen-4-one (2y)

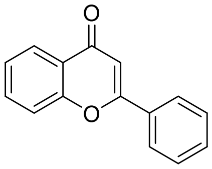

Following the general procedure, **2y** was purified by silica gel chromatography (25% EtOAc/PE). Yield: 47 mg, 21%, a white solid, mp. 98 – 100 °C. ^{1}H NMR (600 MHz, $CDCl_3$) δ 8.25 (dd, J = 8.0, 1.6 Hz, 1H), 7.94 (dd, J = 7.8, 1.8 Hz, 2H), 7.71 (dd, J = 8.6, 7.2 Hz, 1H), 7.58 (d, J = 8.4 Hz, 1H), 7.56 – 7.50 (m, 3H), 7.43 (t, J = 7.2 Hz, 1H), 6.85 (s, 1H). ^{13}C NMR (150 MHz, $CDCl_3$) δ 178.4, 163.5, 156.3, 133.8, 131.9, 131.6, 129.1, 126.3, 125.8, 125.2, 124.0, 118.1, 107.7. HRMS (ESI) calcd for $C_{15}H_{10}NaO_2^+$ [M + Na$^+$] 245.0573, found 245.0576.

Methyl 4-oxo-4*H*-chromene-3-carboxylate (2aa)

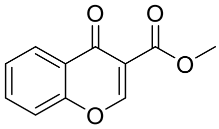

Following the general procedure, **2aa** was purified by silica gel chromatography (15% EtOAc/PE). Yield: 71 mg, 35%, a white solid, mp. 63 –65 °C.^{1}H NMR (600 MHz, $CDCl_3$) δ 7.99 (dd, *J* = 8.0, 1.6 Hz, 1H), 7.62 (t, *J* = 7.2 Hz, 1H), 7.22 (t, *J* = 7.8 Hz, 1H), 7.10 (d, *J* = 8.4 Hz, 1H), 5.73 (s, 1H), 3.93 (s, 3H). ^{13}C NMR (151 MHz, $CDCl_3$) δ 183.2, 163.5, 154.9, 136.8, 127.6, 123.9, 119.0, 118.1, 79.2, 58.6, 53.3. HRMS (ESI) calcd for $C_{11}H_8NaO_4^+$ [M + Na$^+$] 227.0315, found 227.0314.

2-(2-Bromophenyl)-4-oxo-4*H*-chromene-3-carboxylic acid (G)

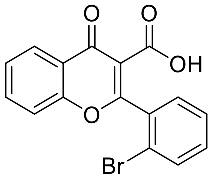

Following the general procedure, the acid **G** was obtained. Yield: 585 mg, 85%, a light yellow solid, mp. 182 – 184 °C. ^{1}H NMR (600 MHz, $CDCl_3$) δ 8.21 (d, *J* = 2.4 Hz, 1H), 7.77 (dd, *J* = 8.8, 2.4 Hz, 1H), 7.68 (d, *J* = 7.2 Hz, 2H), 7.57 (t, *J* = 7.6 Hz, 1H), 7.45 (t, *J* = 7.8 Hz, 2H), 7.20 (d, *J* = 8.8 Hz, 1H). ^{13}C NMR (151 MHz, $CDCl_3$) δ 200.7, 177.0, 158.8, 154.0, 138.9, 138.8, 137.4, 132.9, 128.5, 128.4, 128.0, 118.9, 118.9, 117.3, 116.8, 100.9. HRMS (ESI) calcd for $C_{16}H_9{}^{79}BrNaO_4^+$ [M + Na$^+$] 366.9576, found 366.9577.

6*H*,7*H*-Chromeno[4,3-*b*]chromene-6,7-dione (Frutinone A)

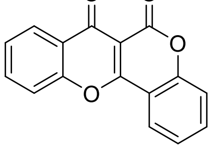

Following the general procedure, Frutinone **A** was purified by silica gel chromatography (25% EtOAc/PE). Yield: 119 mg, 45%, a white solid, mp. 234 – 236 °C. ^{1}H NMR (600 MHz, $CDCl_3$) δ 8.35 (dd, *J* = 7.8, 1.6 Hz, 1H), 8.22 (dd, *J* = 8.0, 1.4 Hz, 1H), 7.81 – 7.73 (m, 2H), 7.64 (d, *J* = 8.4 Hz, 1H), 7.51 (t, *J* = 7.6 Hz, 1H), 7.46 (dd, *J* = 11.4, 3.8 Hz, 1H), 7.41 (d, *J* = 8.4 Hz, 1H). ^{13}C NMR (150 MHz, $CDCl_3$) δ 173.0, 165.0, 156.3, 154.4, 154.3, 135.6, 134.9, 126.9, 126.7, 124.9, 124.5, 124.2, 117.9, 117.5, 113.3, 105.1. HRMS (ESI) calcd for $C_{16}H_8NaO_4^+$ [M + Na$^+$] 287.0315, found 287.0315.

4. The HRMS analysis of PhIO/DMF reaction system

To a suspension of PhIO (1.0 mmol) in MeOH (5 mL) at room temperature. Then the resulting mixture was kept at room temerature. After one hour, the HRMS analysis of the reaction mixture was carried out and the result showed that (PhIO)n would undergo depolymerization to give PhIO species in DMF.

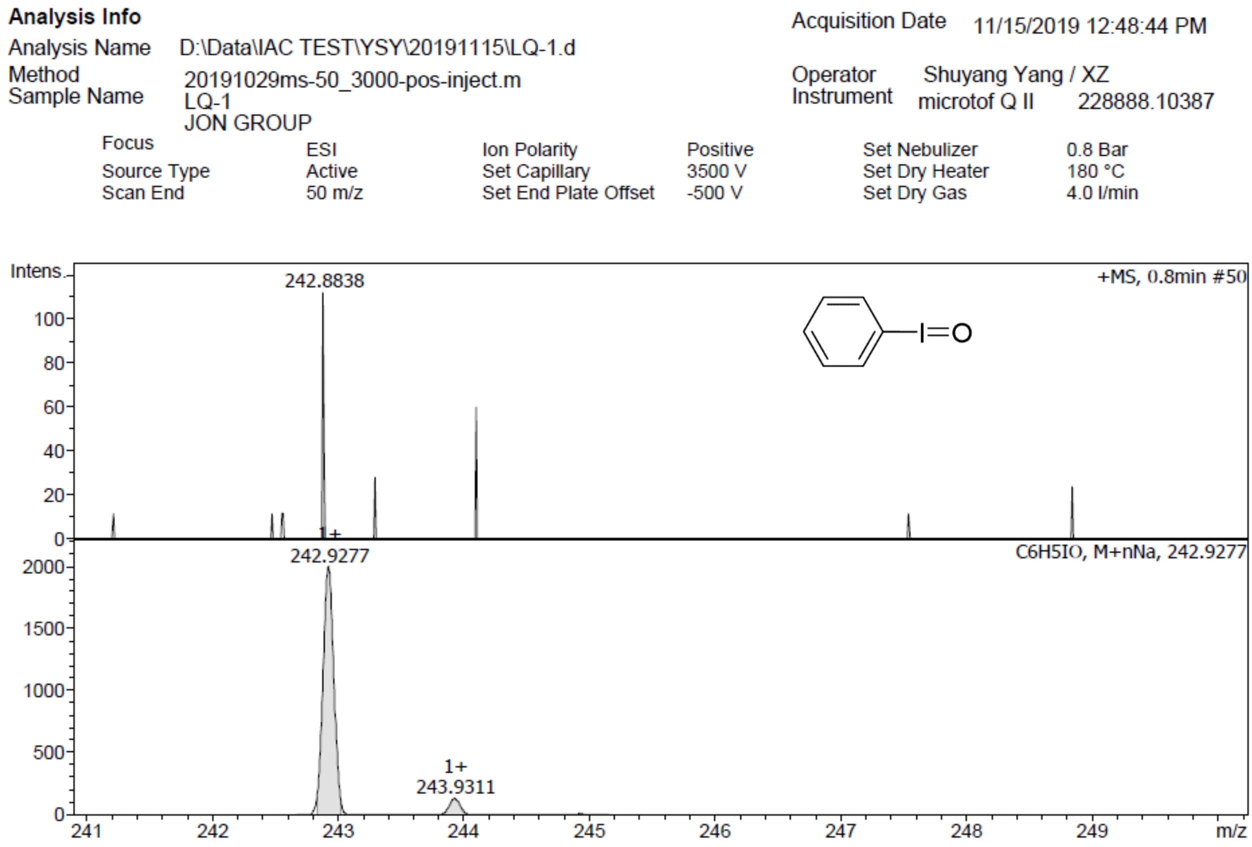

5. References

1. Saltzman, H. Sharefkin, J. G. *Org. Synth.* **1973**, *5,* 658-660.

2 Zhao, X. H.; Ma, K. P.; Zhong, Z. H.; Zhou, Z. P.; Huang, X. Dai, D. J.; Wang, D. CN107176938 2017.

3. Cui, H.; Li, P.; Chai, Z.; Zheng, C.; Zhao, G.; Zhu, S. *J. Org. Chem.* **2019**, *74,* 1400–1402.

4. Boonsombat, J.; Thongnest, S.; Ruchirawat, S. *Eur. J. Org. Chem.* **2019**, 2971-2983.

5. Ding, T. Q.; Jiang, L. Q.; Yang, J.; Xu, Y. M.; Wang, G. X.; Yi, W. B. *Org. Lett.* **2019**, *21*, 6025−6028.

6. ^{1}H-NMR and ^{13}C-NMR Spectra

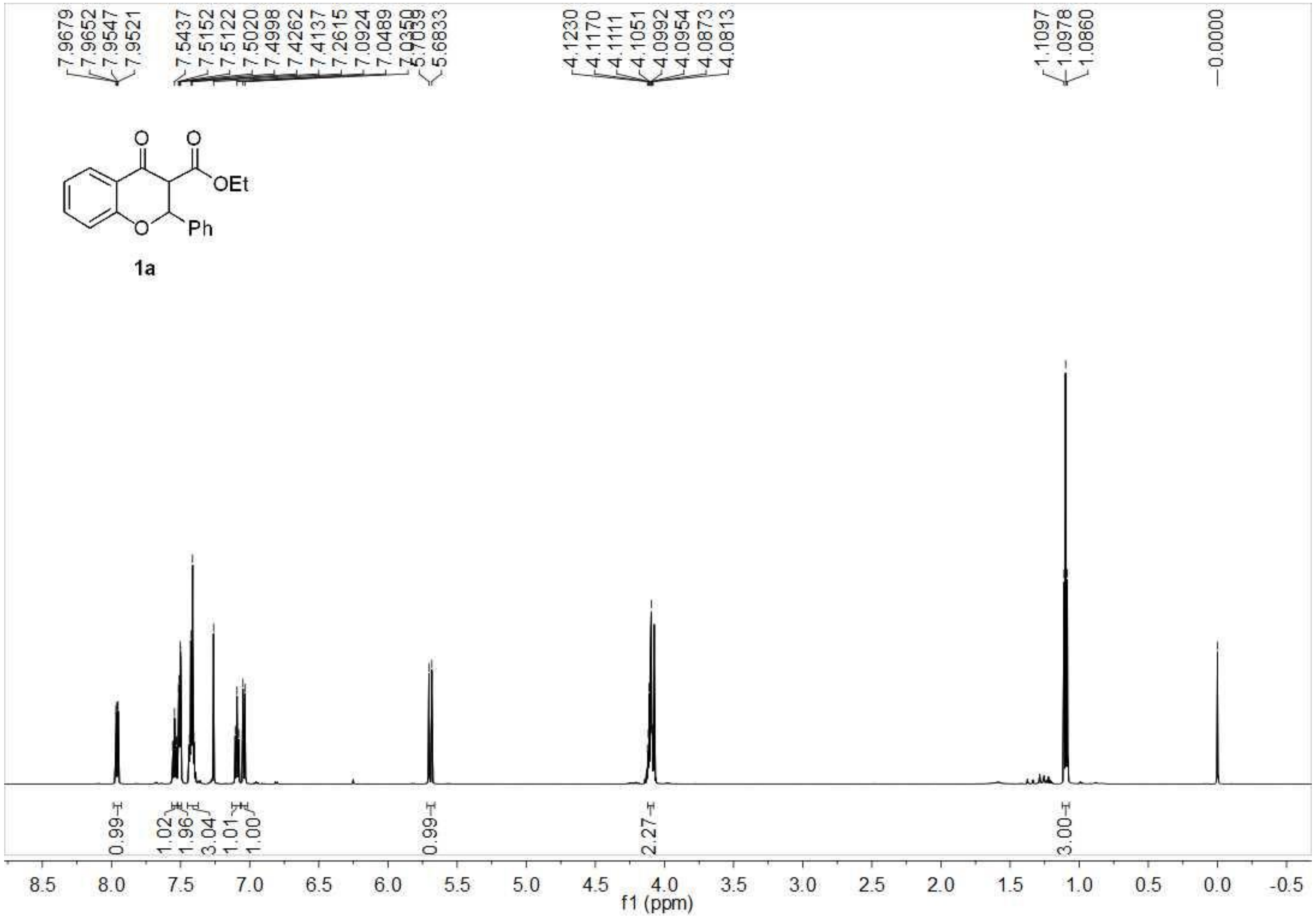

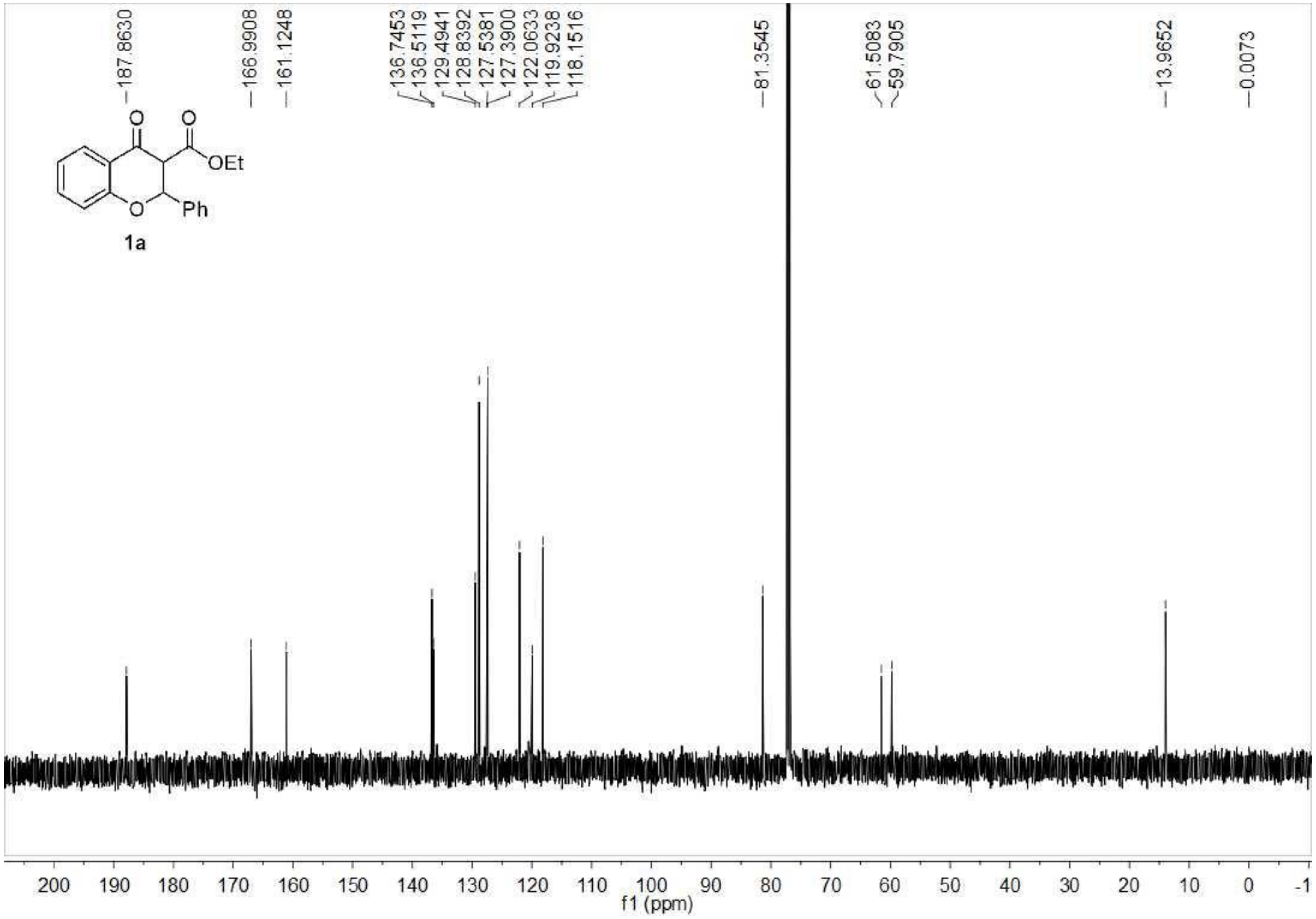
187.8630
166.9908
161.1248
136.7453
136.5119
129.4941
128.8392
127.5381
127.3900
122.0633
119.9238
118.1516
81.3545
61.5083
59.7905
13.9652
0.0073
OEt
Ph
1a
200 190 180 170 160 150 140 130 120 110 100 90 80 70 60 50 40 30 20 10 0 -1
f1 (ppm)

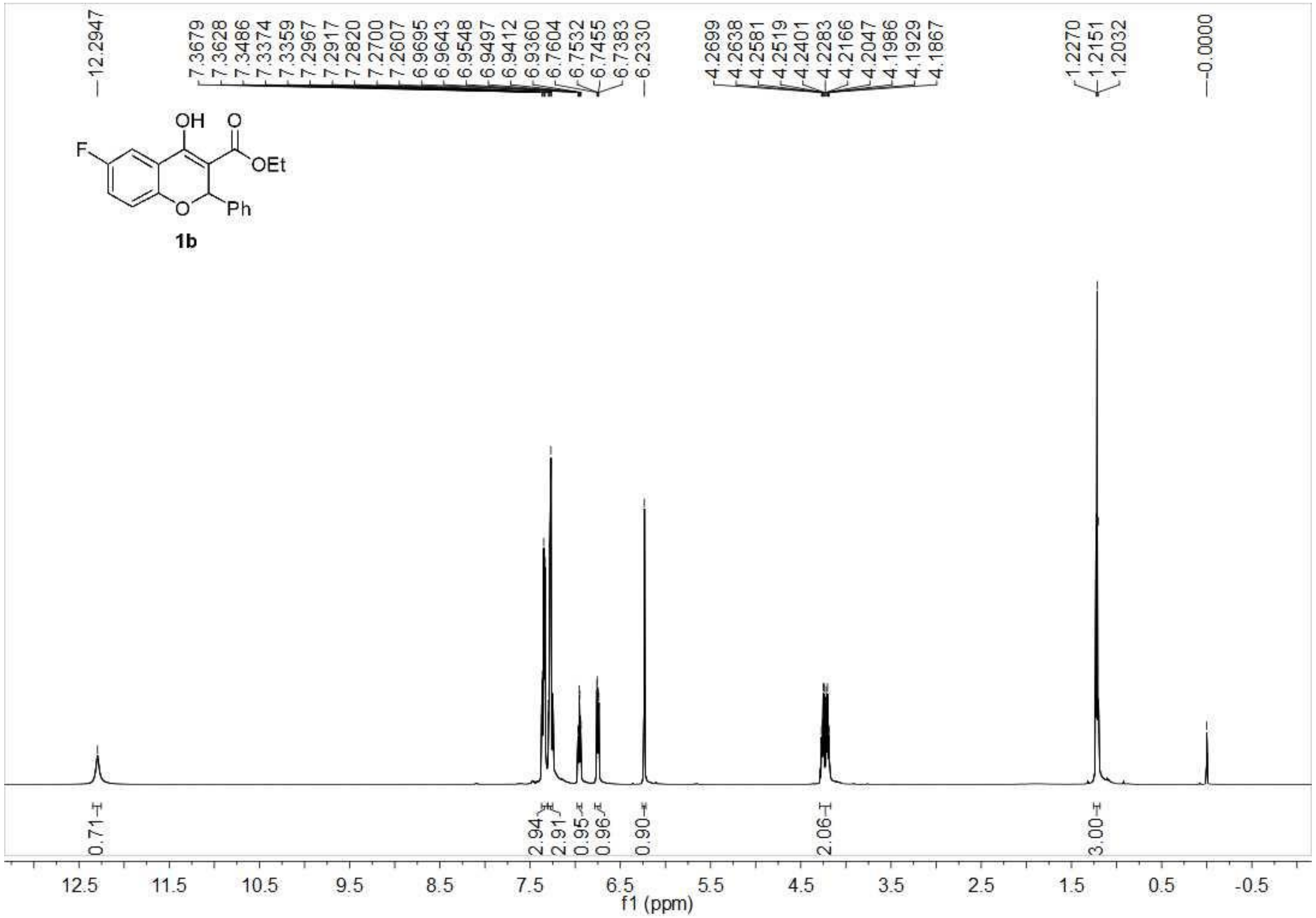
12.2947
7.3679
7.3628
7.3486
7.3374
7.3359
7.2967
7.2917
7.2820
7.2700
7.2607
6.9695
6.9643
6.9548
6.9497
6.9412
6.9360
6.7604
6.7532
6.7455
6.7383
6.2330
4.2699
4.2638
4.2581
4.2519
4.2401
4.2283
4.2166
4.2047
4.1986
4.1929
4.1867
1.2270
1.2151
1.2032
0.0000
OH
O
F
OEt
O
Ph
1b
0.71
2.94
2.91
0.95
0.96
0.90
2.06
3.00
12.5
11.5
10.5
9.5
8.5
7.5
6.5
5.5
4.5
3.5
2.5
1.5
0.5
-0.5
f1 (ppm)

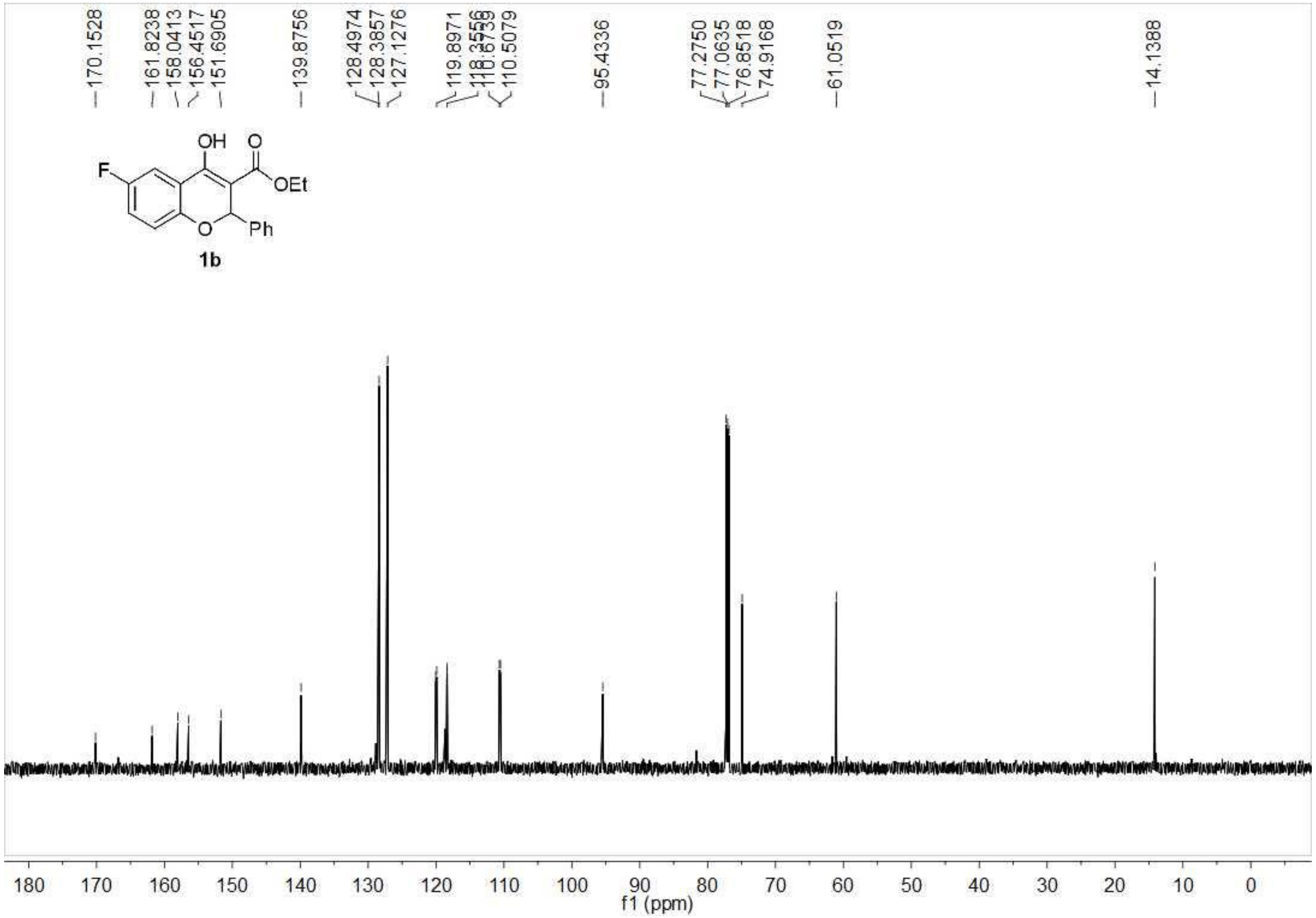
170.1528
161.8238
158.0413
156.4517
151.6905
139.8756
128.4974
128.3857
127.1276
119.8971
118.3556
110.6739
110.5079
95.4336
77.2750
77.0635
76.8518
74.9168
61.0519
14.1388
OH
O
F
OEt
O
Ph
1b
180
170
160
150
140
130
120
110
100
90
80
70
60
50
40
30
20
10
0
f1 (ppm)

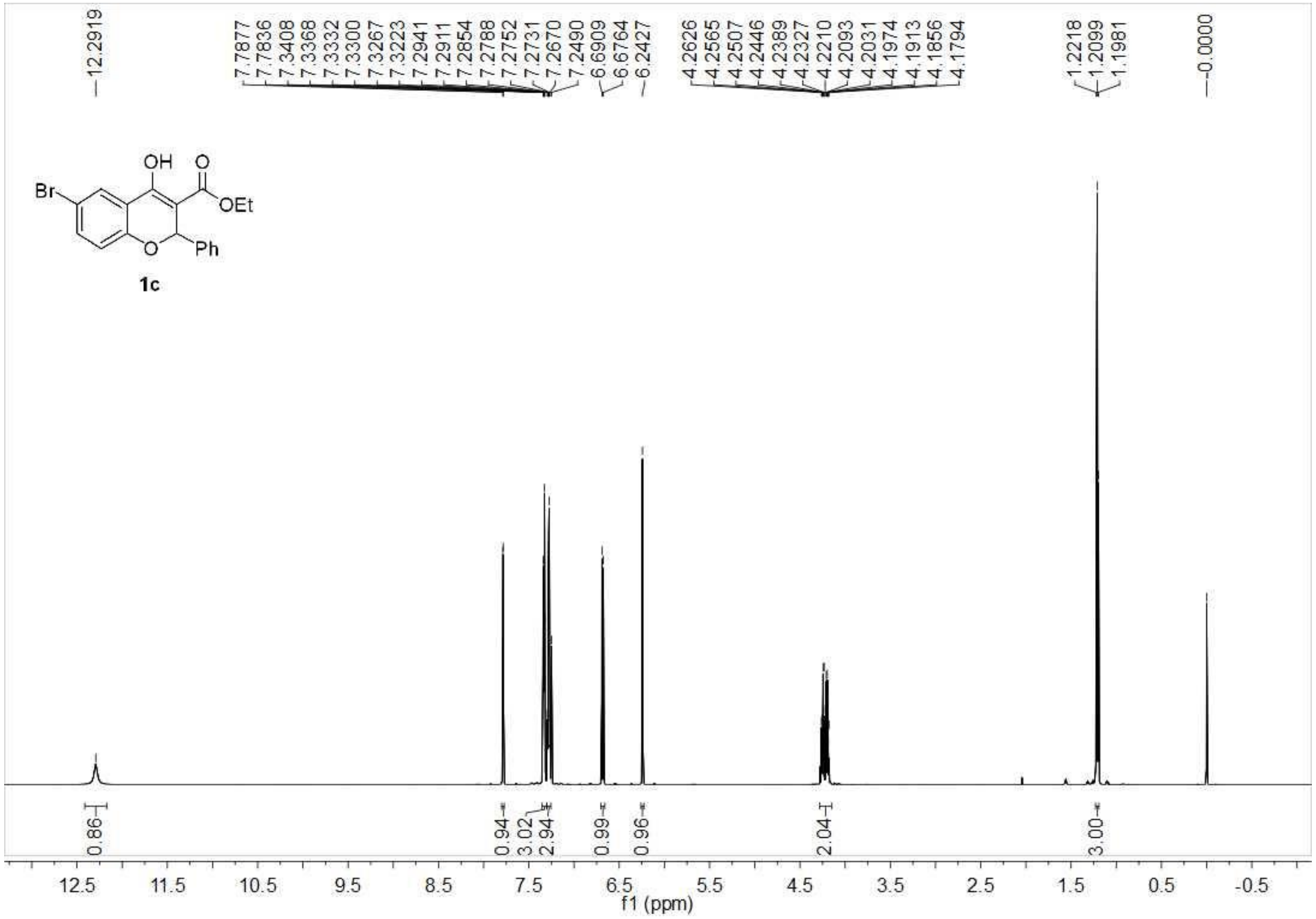

12.2919
7.7877
7.7836
7.3408
7.3368
7.3332
7.3300
7.3267
7.3223
7.2941
7.2911
7.2854
7.2788
7.2752
7.2731
7.2670
7.2490
6.6909
6.6764
6.2427
4.2626
4.2565
4.2507
4.2446
4.2389
4.2327
4.2210
4.2093
4.2031
4.1974
4.1913
4.1856
4.1794
1.2218
1.2099
1.1981
-0.0000
Br
OH
O
OEt
O
Ph
1c
0.86
0.94
3.02
2.94
0.99
0.96
2.04
3.00
12.5
11.5
10.5
9.5
8.5
7.5
6.5
5.5
4.5
3.5
2.5
1.5
0.5
-0.5
f1 (ppm)

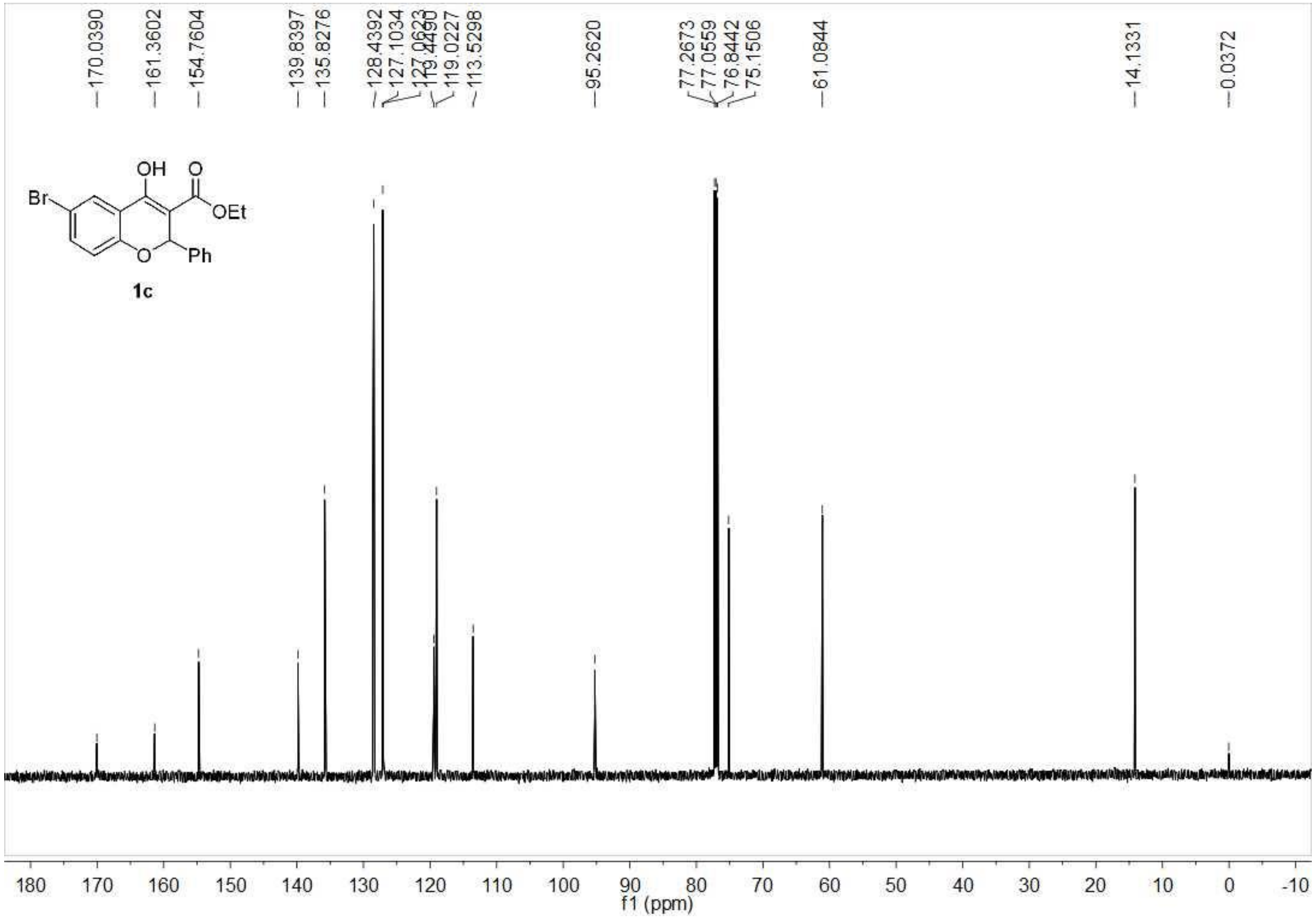
170.0390
161.3602
154.7604
139.8397
135.8276
128.4392
127.1034
127.0623
119.4490
119.0227
113.5298
95.2620
77.2673
77.0559
76.8442
75.1506
61.0844
14.1331
0.0372
OH
O
Br
OEt
O
Ph
1c
180
170
160
150
140
130
120
110
100
90
80
70
60
50
40
30
20
10
0
-10
f1 (ppm)

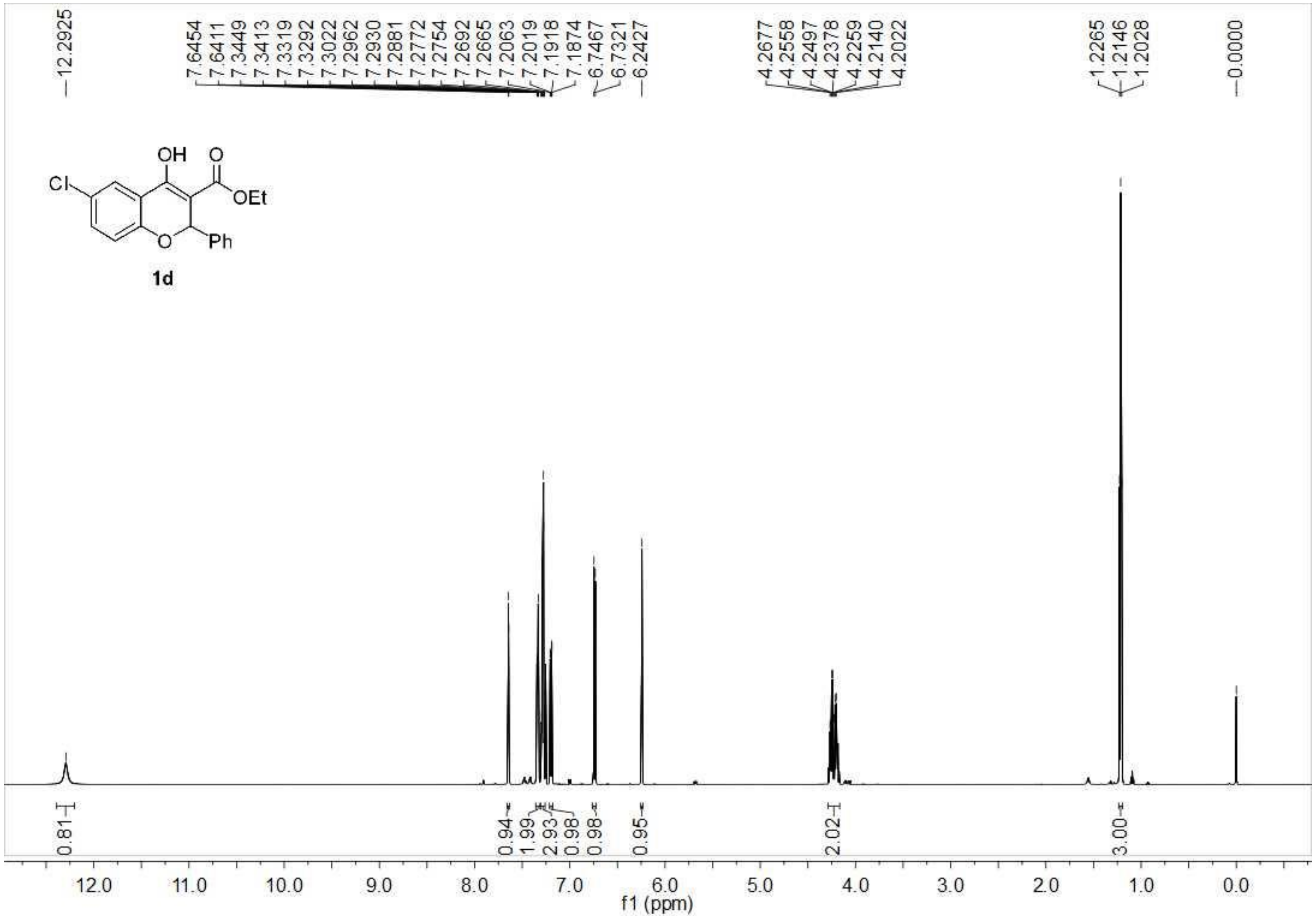
12.2925
7.6454
7.6411
7.3449
7.3413
7.3319
7.3292
7.3022
7.2962
7.2930
7.2881
7.2772
7.2754
7.2692
7.2665
7.2063
7.2019
7.1918
7.1874
6.7467
6.7321
6.2427
4.2677
4.2558
4.2497
4.2378
4.2259
4.2140
4.2022
1.2265
1.2146
1.2028
0.0000
OH
O
Cl
OEt
O
Ph
1d
0.81
0.94
1.99
2.93
0.98
0.98
0.95
2.02
3.00
12.0
11.0
10.0
9.0
8.0
7.0
6.0
5.0
4.0
3.0
2.0
1.0
0.0
f1 (ppm)

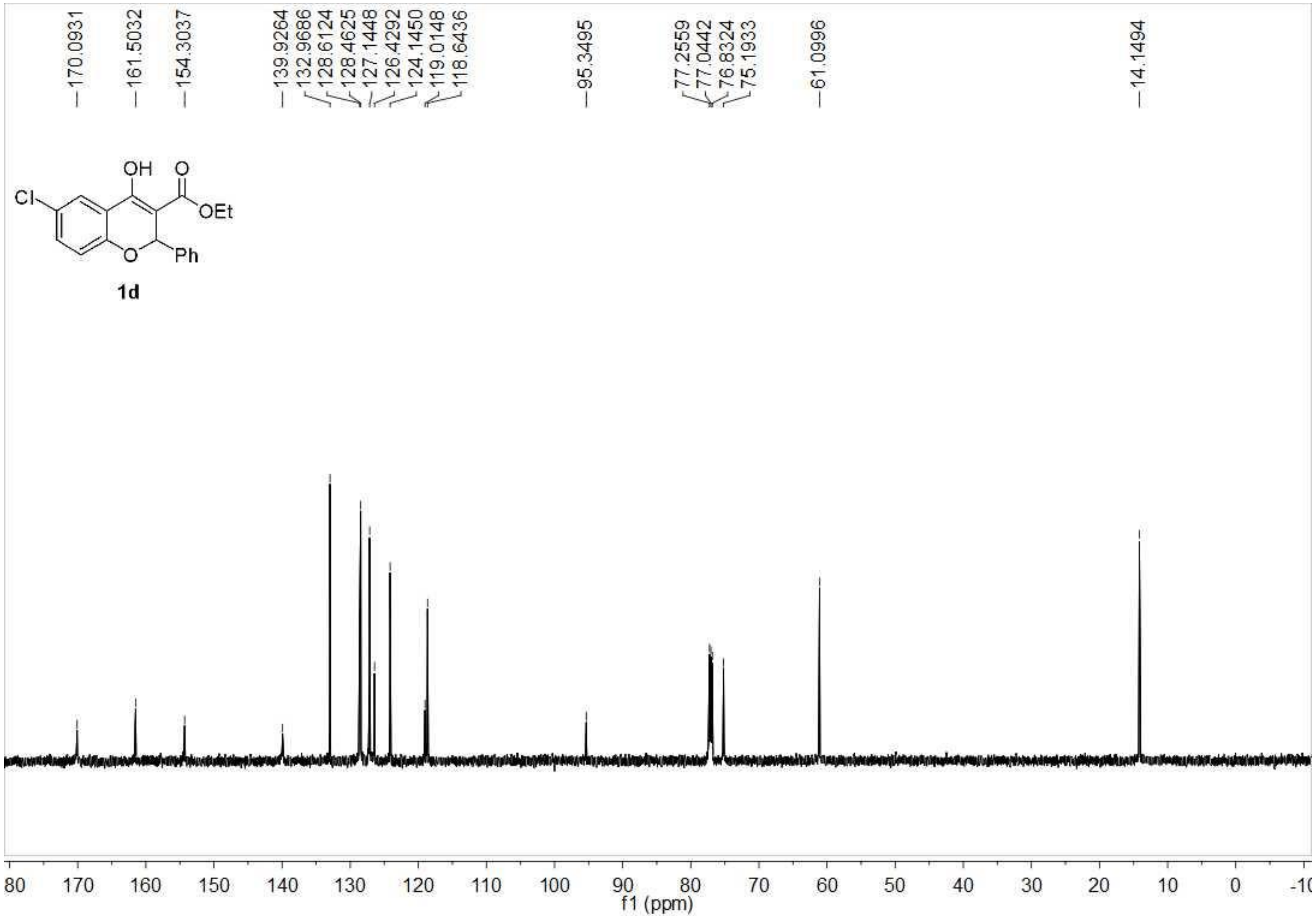
170.0931
161.5032
154.3037
139.9264
132.9686
128.6124
128.4625
127.1448
126.4292
124.1450
119.0148
118.6436
95.3495
77.2559
77.0442
76.8324
75.1933
61.0996
14.1494
OH
O
Cl
OEt
O
Ph
1d
f1 (ppm)

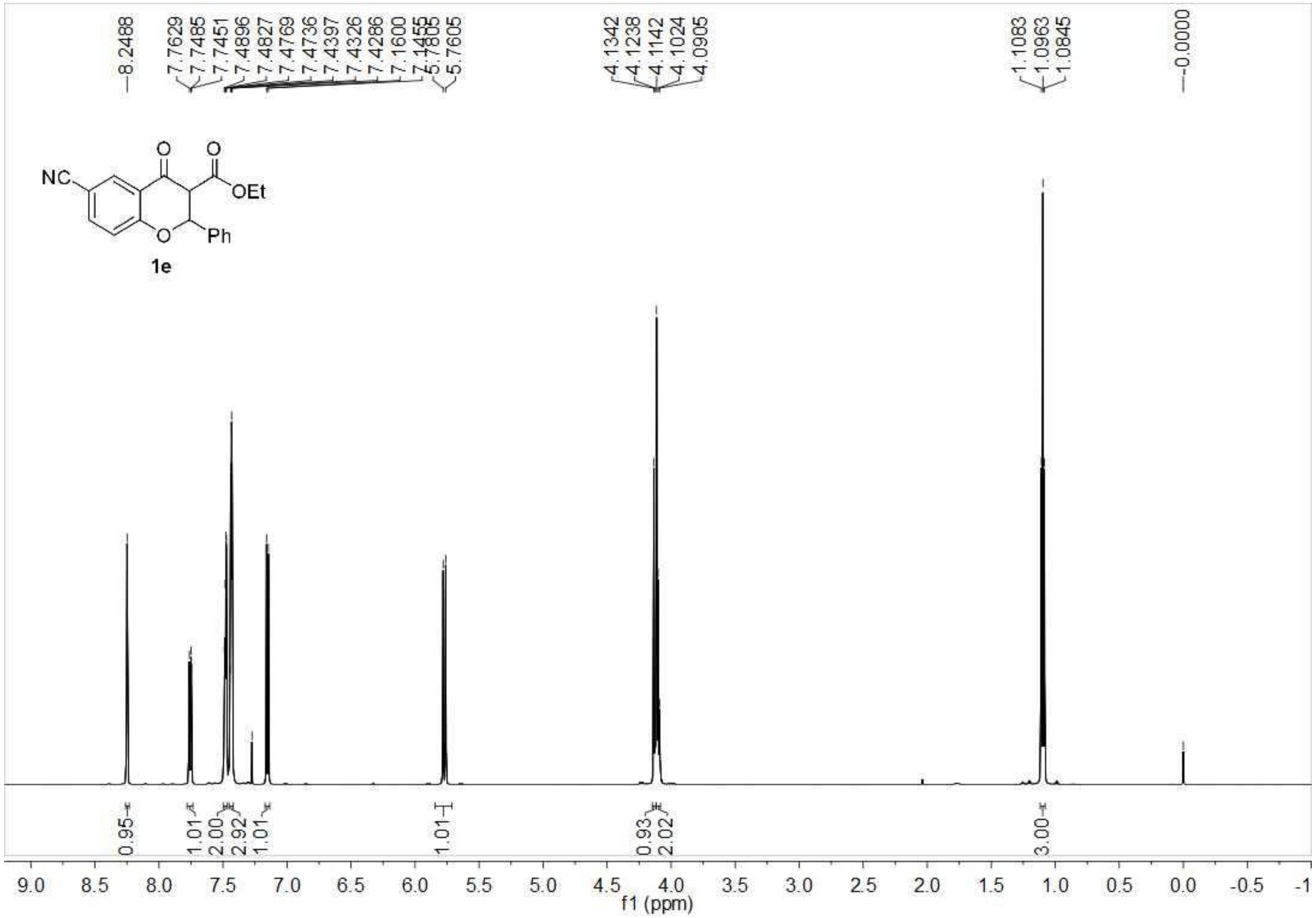

8.2488
7.7629
7.7485
7.7451
7.4896
7.4827
7.4769
7.4736
7.4397
7.4326
7.4286
7.1600
7.1455
5.7805
5.7605
4.1342
4.1238
4.1142
4.1024
4.0905
1.1083
1.0963
1.0845
0.0000
NC
O
O
OEt
O
Ph
1e
0.95
1.01
2.00
2.92
1.01
1.01
0.93
2.02
3.00
f1 (ppm)

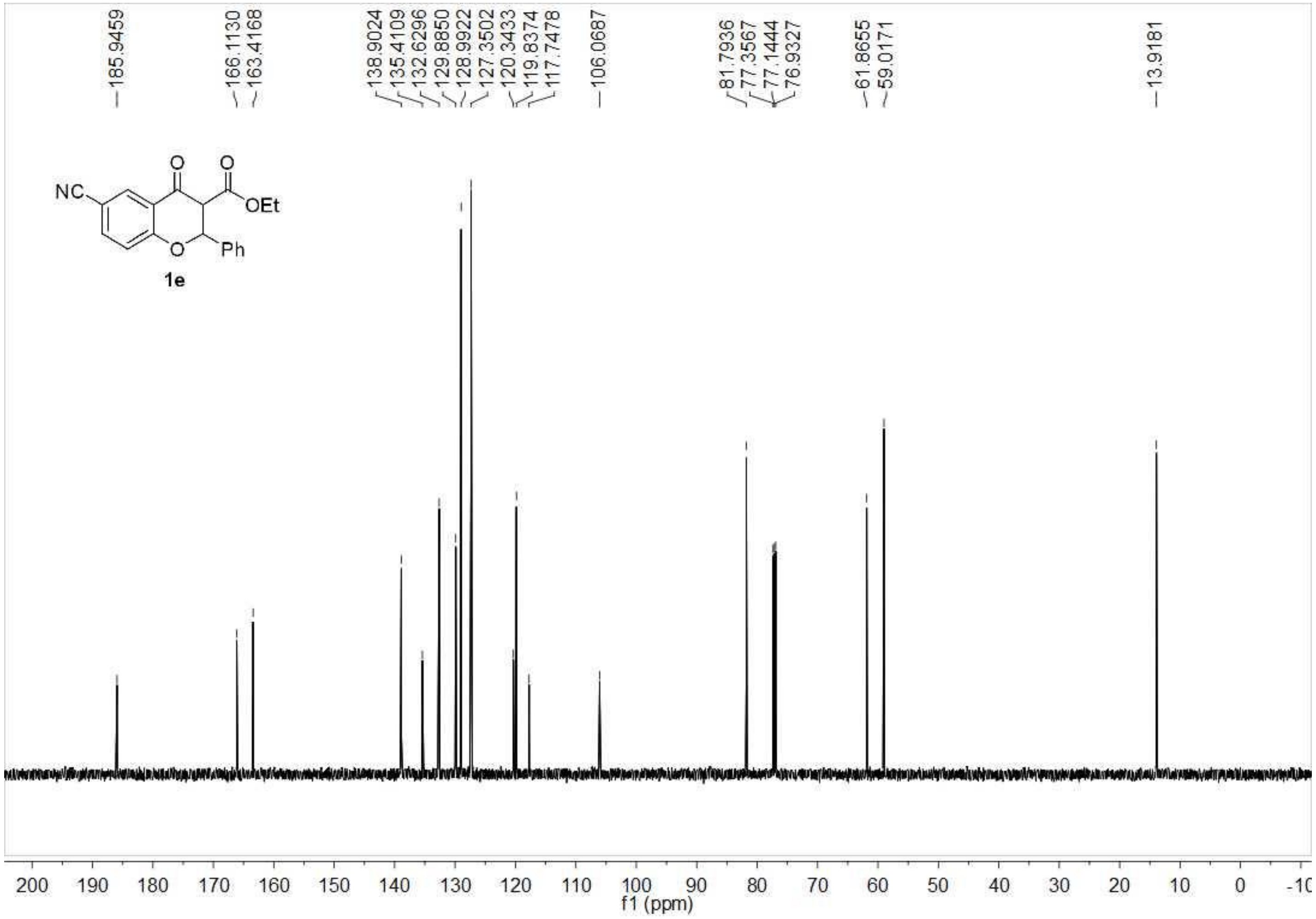
185.9459
166.1130
163.4168
138.9024
135.4109
132.6296
129.8850
128.9922
127.3502
120.3433
119.8374
117.7478
106.0687
81.7936
77.3567
77.1444
76.9327
61.8655
59.0171
13.9181
NC
O
O
OEt
O
Ph
1e
200 190 180 170 160 150 140 130 120 110 100 90 80 70 60 50 40 30 20 10 0 -10
f1 (ppm)

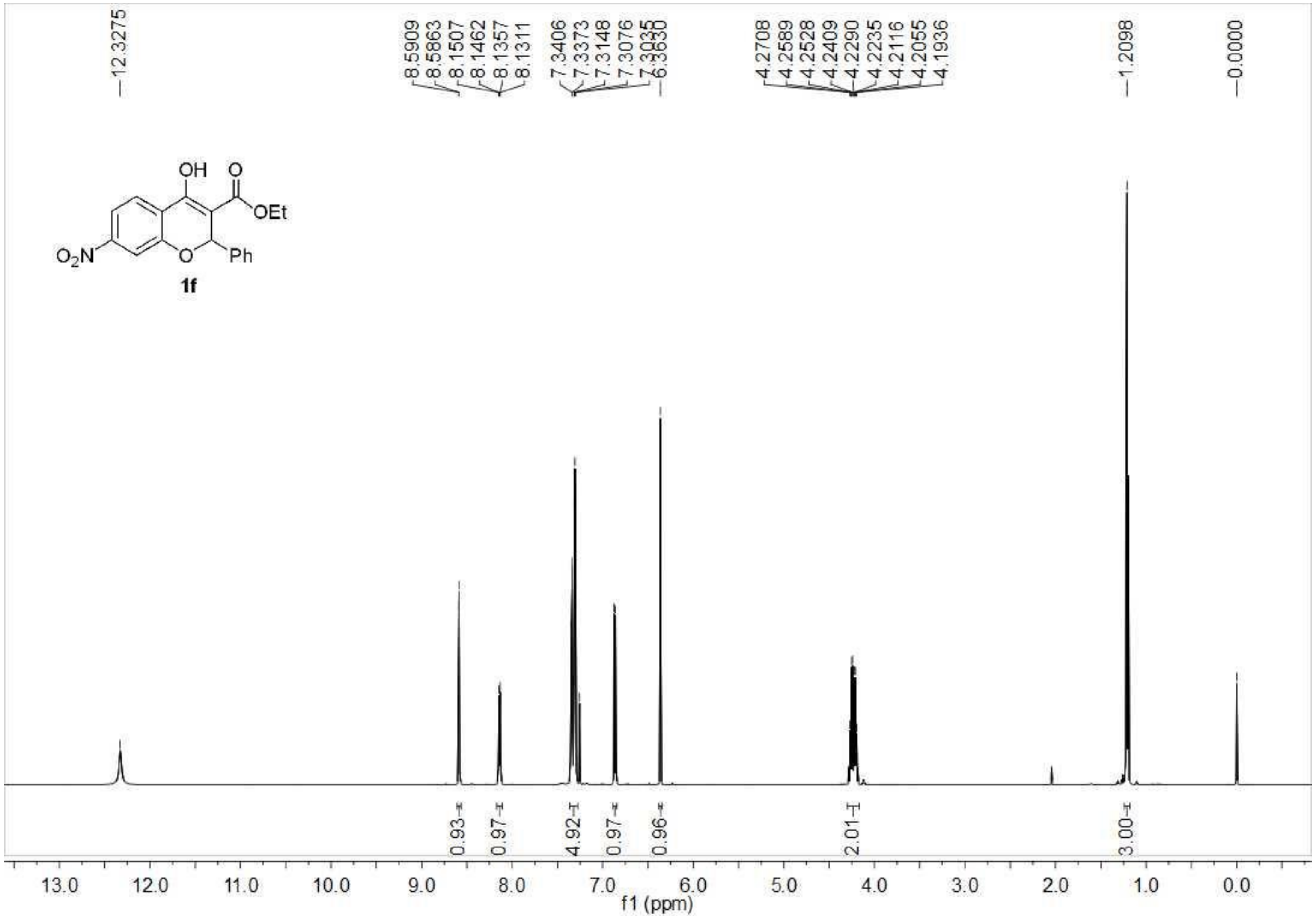

12.3275
8.5909
8.5863
8.1507
8.1462
8.1357
8.1311
7.3406
7.3373
7.3148
7.3076
7.3035
6.3630
4.2708
4.2589
4.2528
4.2409
4.2290
4.2235
4.2116
4.2055
4.1936
1.2098
0.0000
OH
O
OEt
O_2N
O
Ph
1f
0.93
0.97
4.92
0.97
0.96
2.01
3.00
13.0
12.0
11.0
10.0
9.0
8.0
7.0
6.0
5.0
4.0
3.0
2.0
1.0
0.0
f1 (ppm)

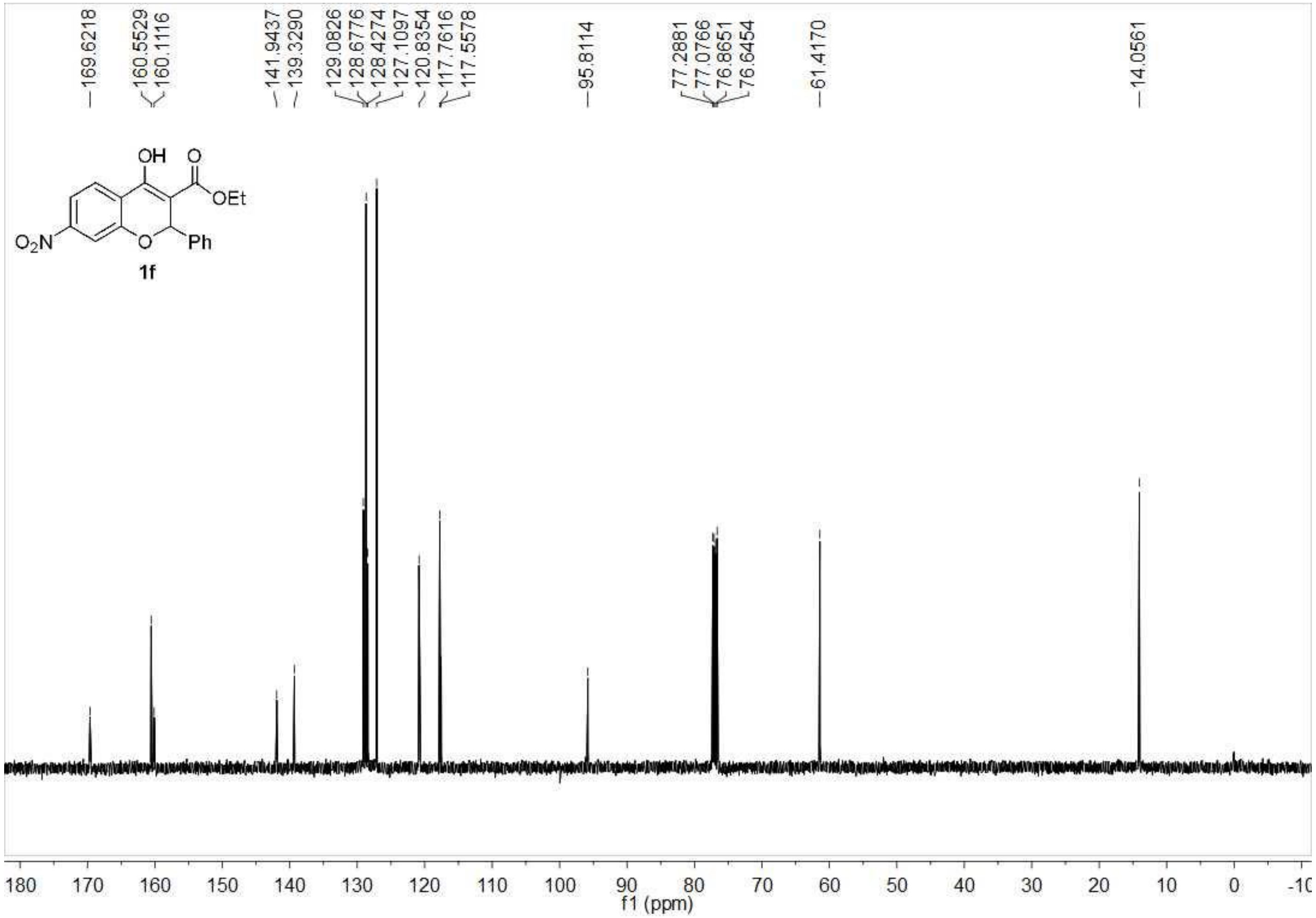
169.6218
160.5529
160.1116
141.9437
139.3290
129.0826
128.6776
128.4274
127.1097
120.8354
117.7616
117.5578
95.8114
77.2881
77.0766
76.8651
76.6454
61.4170
14.0561
OH
O
OEt
O_2N
O
Ph
1f
180 170 160 150 140 130 120 110 100 90 80 70 60 50 40 30 20 10 0 -10
f1 (ppm)

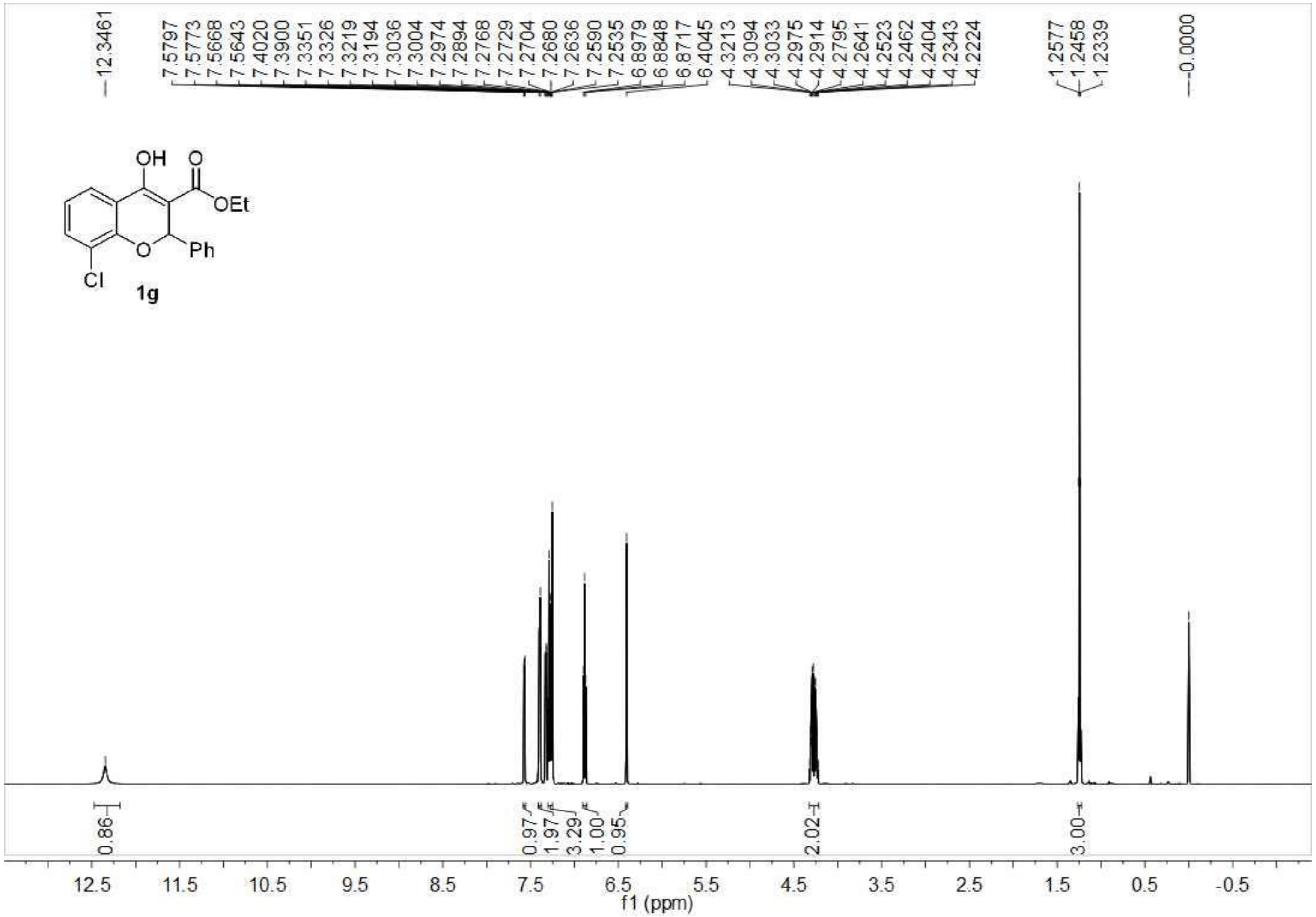
12.3461
7.5797
7.5773
7.5668
7.5643
7.4020
7.3900
7.3351
7.3326
7.3219
7.3194
7.3036
7.3004
7.2974
7.2894
7.2768
7.2729
7.2704
7.2680
7.2636
7.2590
7.2535
6.8979
6.8848
6.8717
6.4045
4.3213
4.3094
4.3033
4.2975
4.2914
4.2795
4.2641
4.2523
4.2462
4.2404
4.2343
4.2224
1.2577
1.2458
1.2339
0.0000
OH
O
OEt
O
Ph
Cl
1g
0.86
0.97
1.97
3.29
1.00
0.95
2.02
3.00
12.5
11.5
10.5
9.5
8.5
7.5
6.5
5.5
4.5
3.5
2.5
1.5
0.5
-0.5
f1 (ppm)

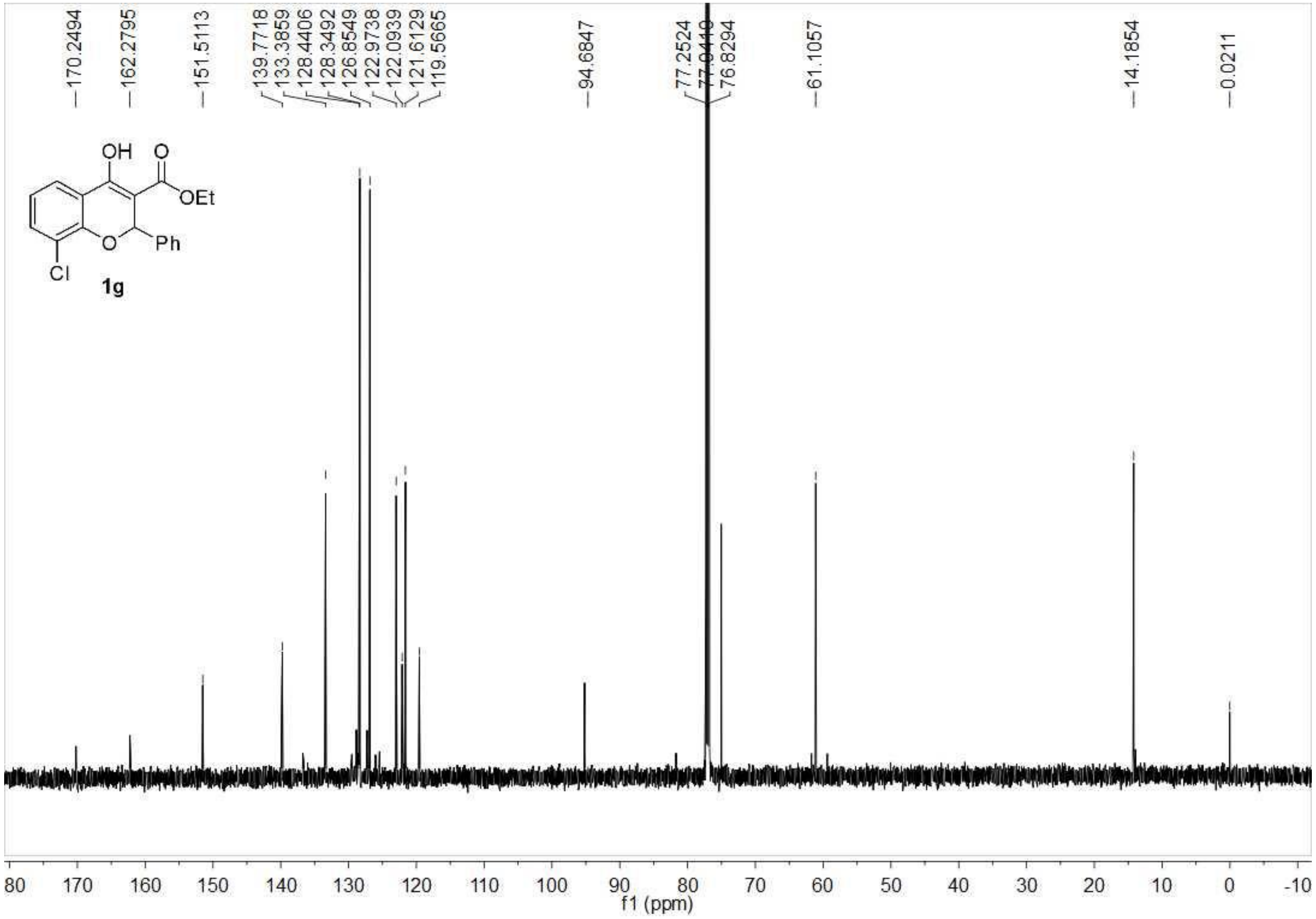
170.2494
162.2795
151.5113
139.7718
133.3859
128.4406
128.3492
126.8549
122.9738
122.0939
121.6129
119.5665
94.6847
77.2524
77.0410
76.8294
61.1057
14.1854
0.0211
OH
O
OEt
O
Ph
Cl
1g
f1 (ppm)

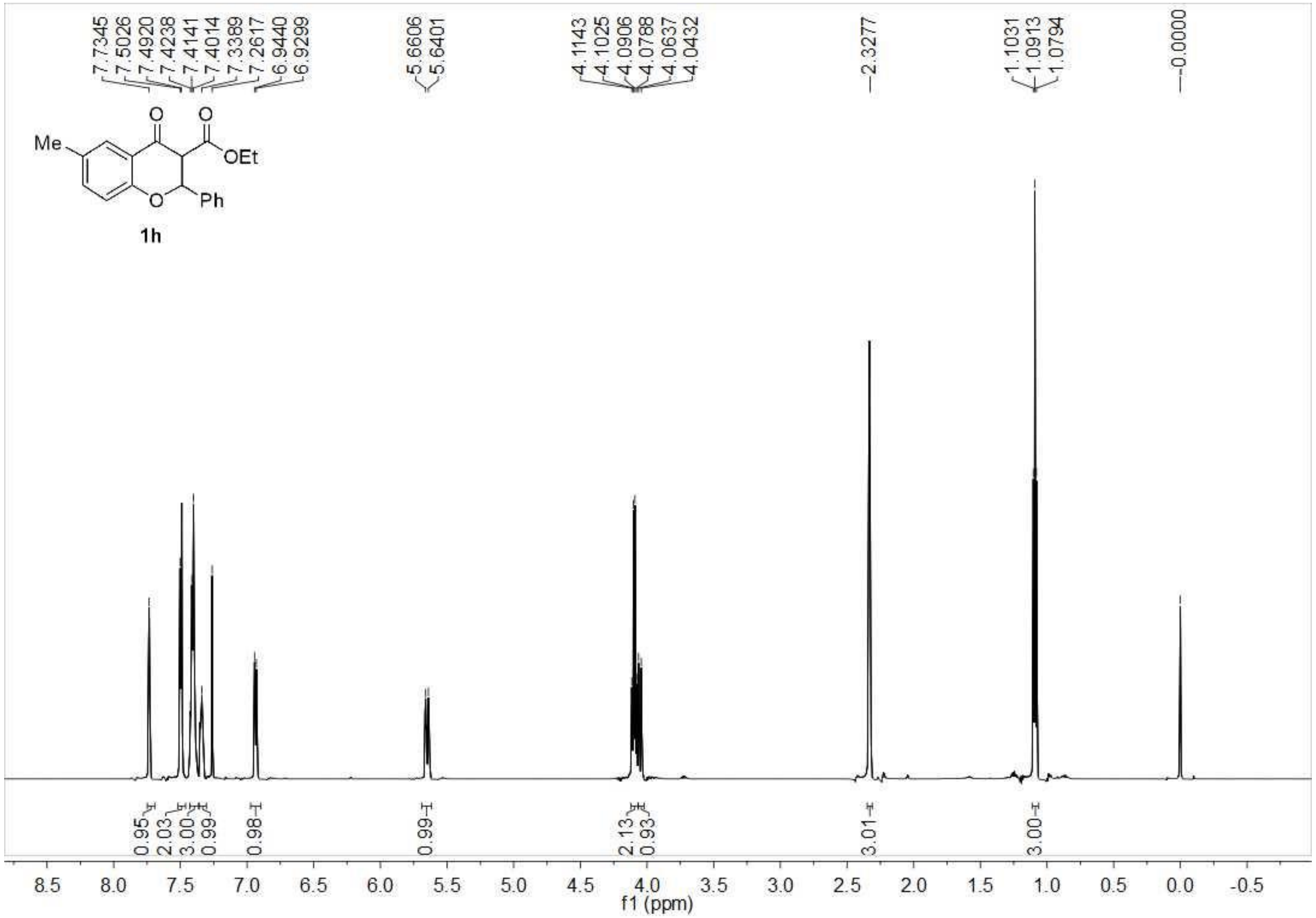

7.7345
7.5026
7.4920
7.4238
7.4141
7.4014
7.3389
7.2617
6.9440
6.9299
5.6606
5.6401
4.1143
4.1025
4.0906
4.0788
4.0637
4.0432
2.3277
1.1031
1.0913
1.0794
0.0000
Me
O
O
OEt
O
Ph
1h
0.95
2.03
3.00
0.99
0.98
0.99
2.13
0.93
3.01
3.00
8.5
8.0
7.5
7.0
6.5
6.0
5.5
5.0
4.5
4.0
3.5
3.0
2.5
2.0
1.5
1.0
0.5
0.0
-0.5
f1 (ppm)

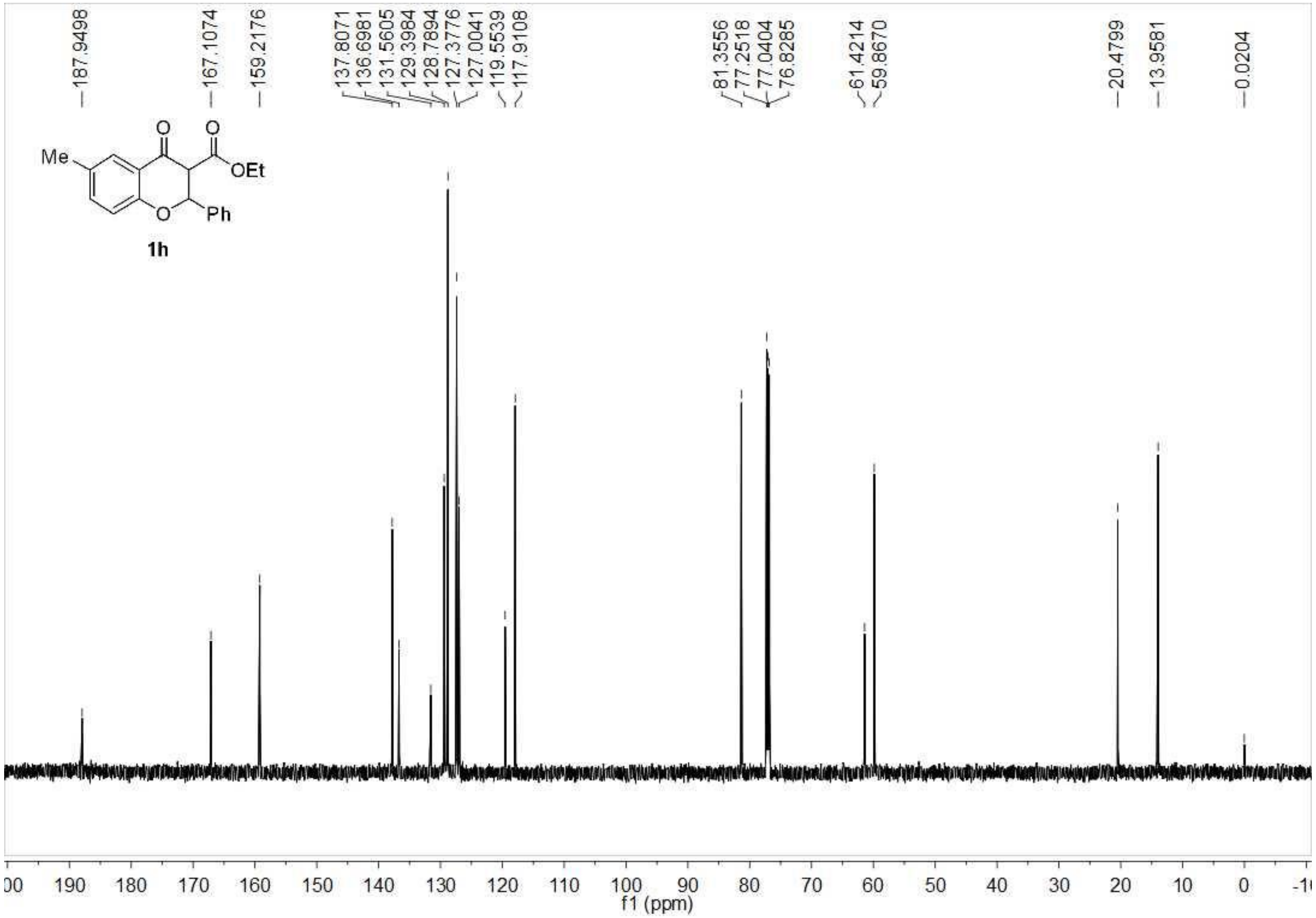

187.9498
167.1074
159.2176
137.8071
136.6981
131.5605
129.3984
128.7894
127.3776
127.0041
119.5539
117.9108
81.3556
77.2518
77.0404
76.8285
61.4214
59.8670
20.4799
13.9581
0.0204
Me
O
O
OEt
O
Ph
1h
00
190
180
170
160
150
140
130
120
110
100
90
80
70
60
50
40
30
20
10
0
-1
f1 (ppm)

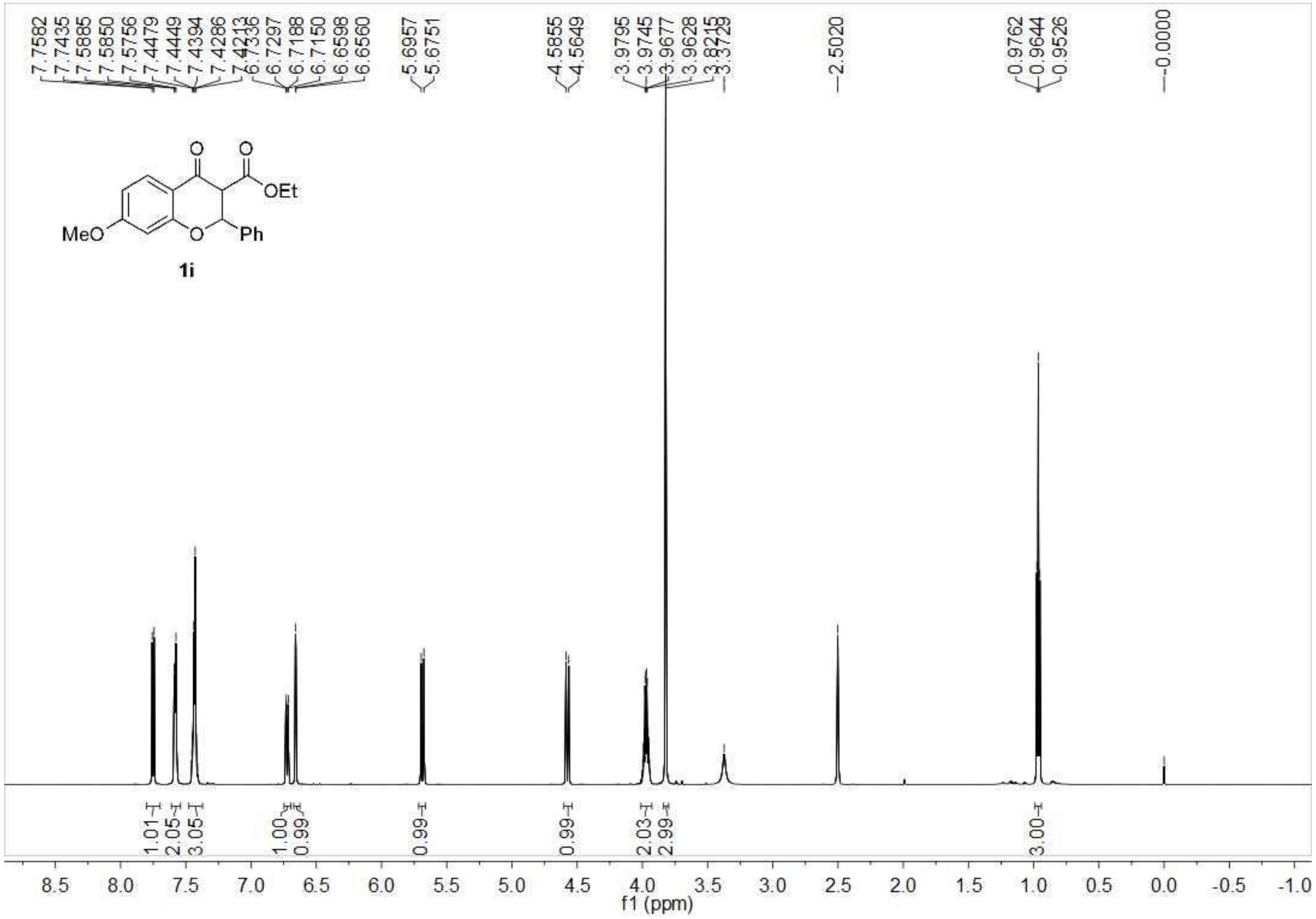
7.7582
7.7435
7.5885
7.5850
7.5756
7.4479
7.4449
7.4394
7.4286
7.4213
6.7336
6.7297
6.7188
6.7150
6.6598
6.6560
5.6957
5.6751
4.5855
4.5649
3.9795
3.9745
3.9677
3.9628
3.8215
3.3729
2.5020
0.9762
0.9644
0.9526
0.0000
MeO
O
O
O
OEt
Ph
1i
1.01
2.05
3.05
1.00
0.99
0.99
0.99
2.03
2.99
3.00
8.5
8.0
7.5
7.0
6.5
6.0
5.5
5.0
4.5
4.0
3.5
3.0
2.5
2.0
1.5
1.0
0.5
0.0
-0.5
-1.0
f1 (ppm)

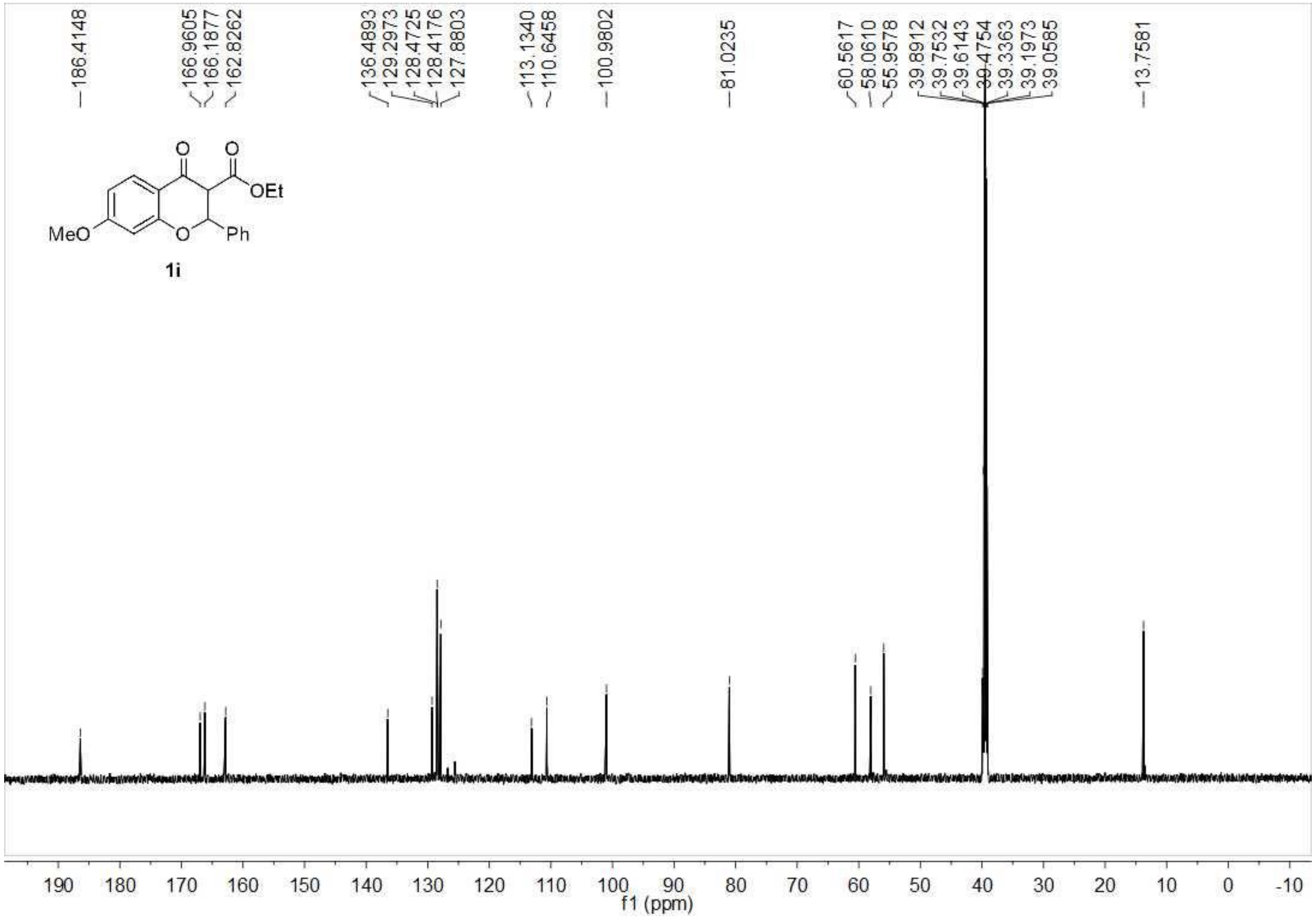
186.4148
166.9605
166.1877
162.8262
136.4893
129.2973
128.4725
128.4176
127.8803
113.1340
110.6458
100.9802
81.0235
60.5617
58.0610
55.9578
39.8912
39.7532
39.6143
39.4754
39.3363
39.1973
39.0585
13.7581
O
O
OEt
MeO
O
Ph
1i
190 180 170 160 150 140 130 120 110 100 90 80 70 60 50 40 30 20 10 0 -10
f1 (ppm)

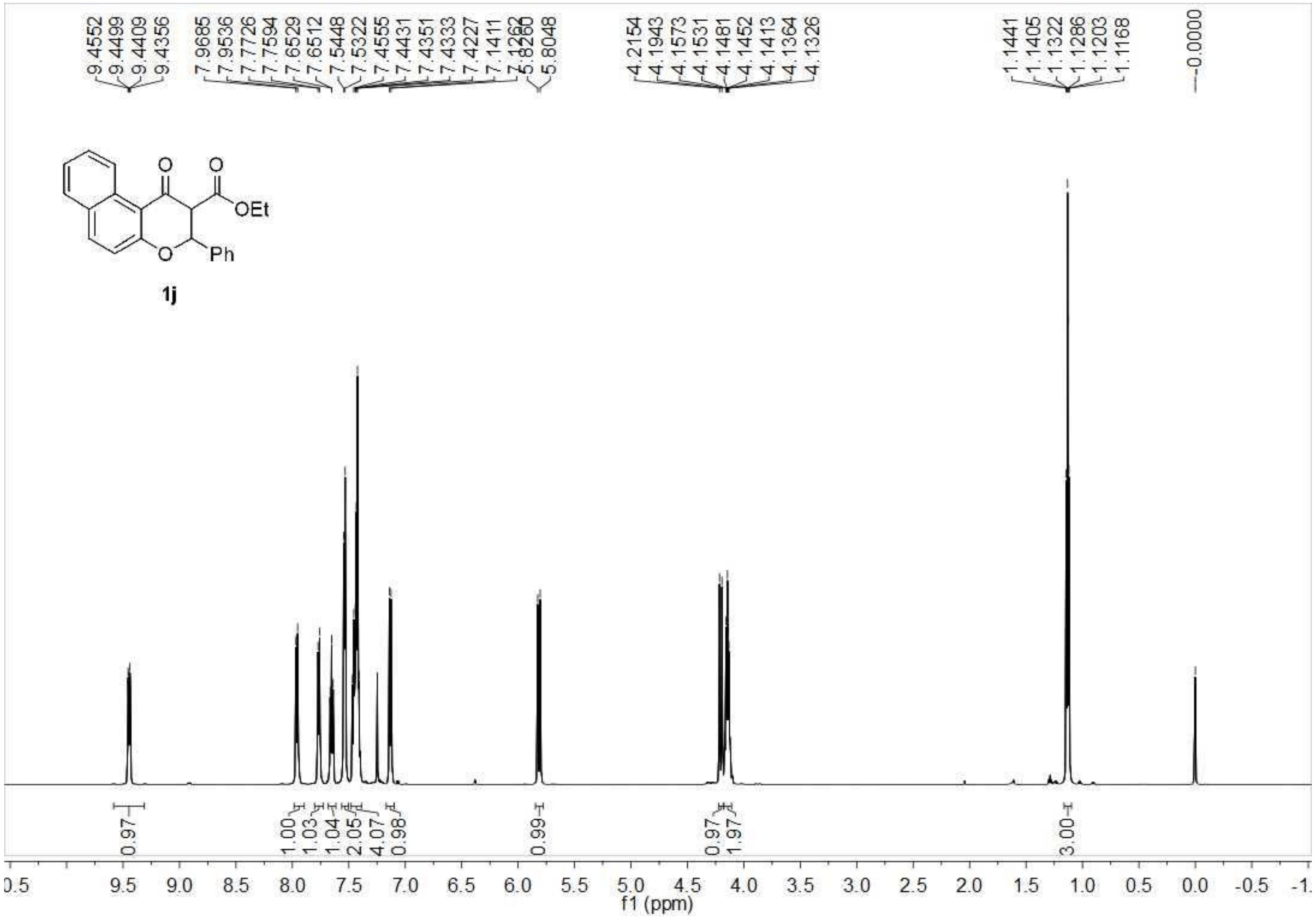
9.4552
9.4499
9.4409
9.4356
7.9685
7.9536
7.7726
7.7594
7.6529
7.6512
7.5448
7.5322
7.4555
7.4431
7.4351
7.4333
7.4227
7.1411
7.1262
5.8200
5.8048
4.2154
4.1943
4.1573
4.1531
4.1481
4.1452
4.1413
4.1364
4.1326
1.1441
1.1405
1.1322
1.1286
1.1203
1.1168
0.0000
O
O
OEt
O
Ph
1j
0.97
1.00
1.03
1.04
2.05
4.07
0.98
0.99
0.97
1.97
3.00
f1 (ppm)

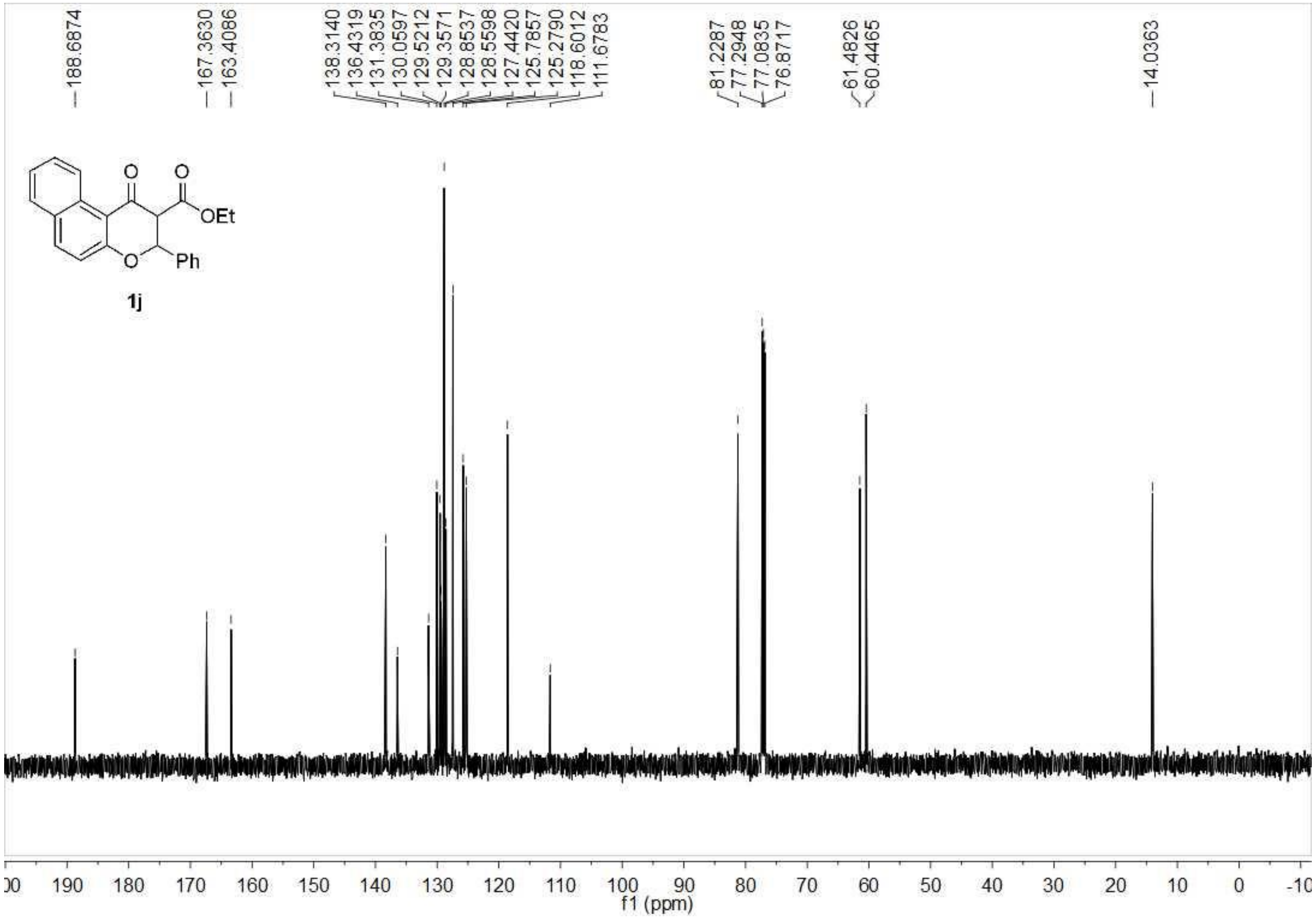

188.6874
167.3630
163.4086
138.3140
136.4319
131.3835
130.0597
129.5212
129.3571
128.8537
128.5598
127.4420
125.7857
125.2790
118.6012
111.6783
81.2287
77.2948
77.0835
76.8717
61.4826
60.4465
14.0363
OEt
Ph
O
1j
f1 (ppm)

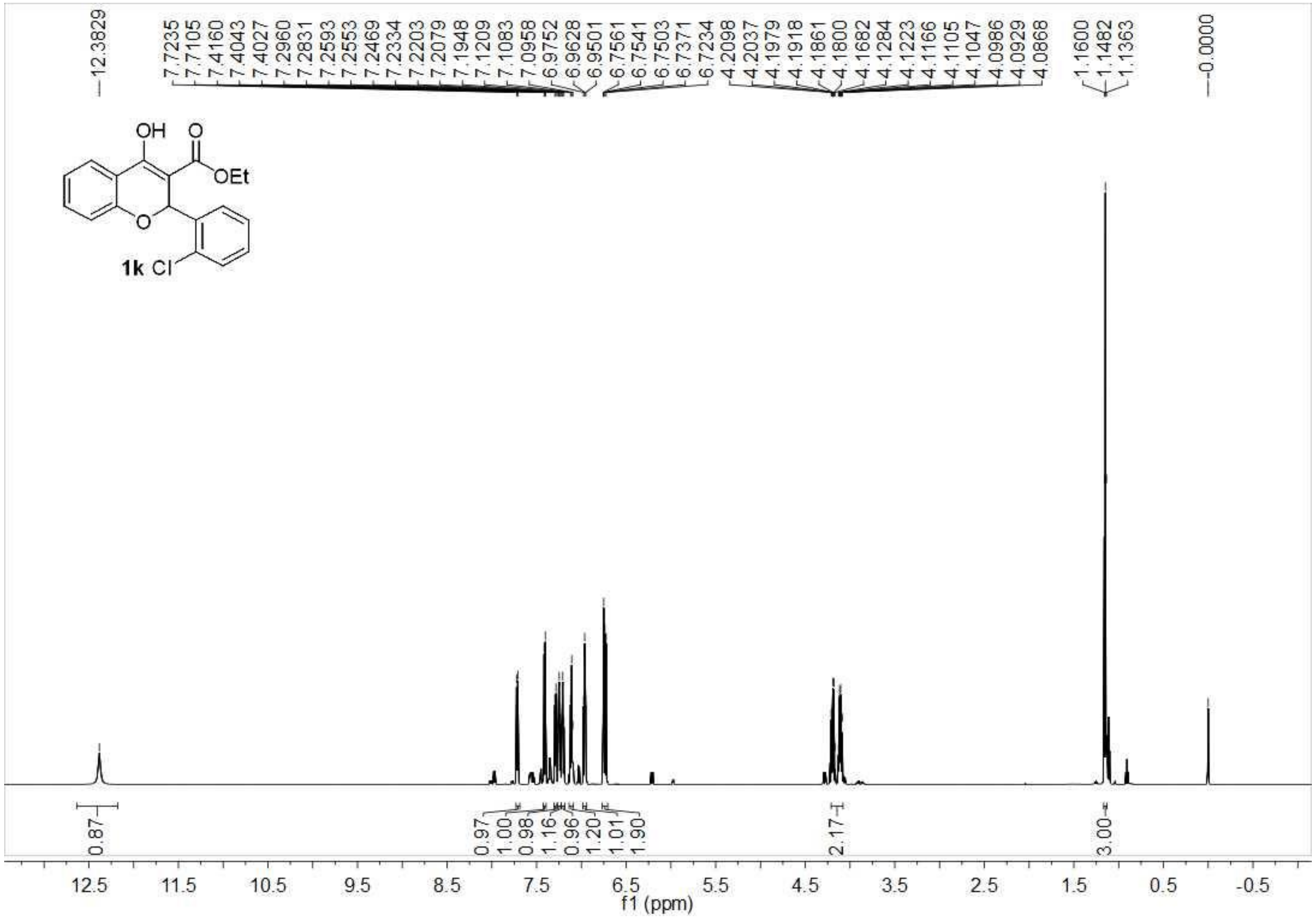

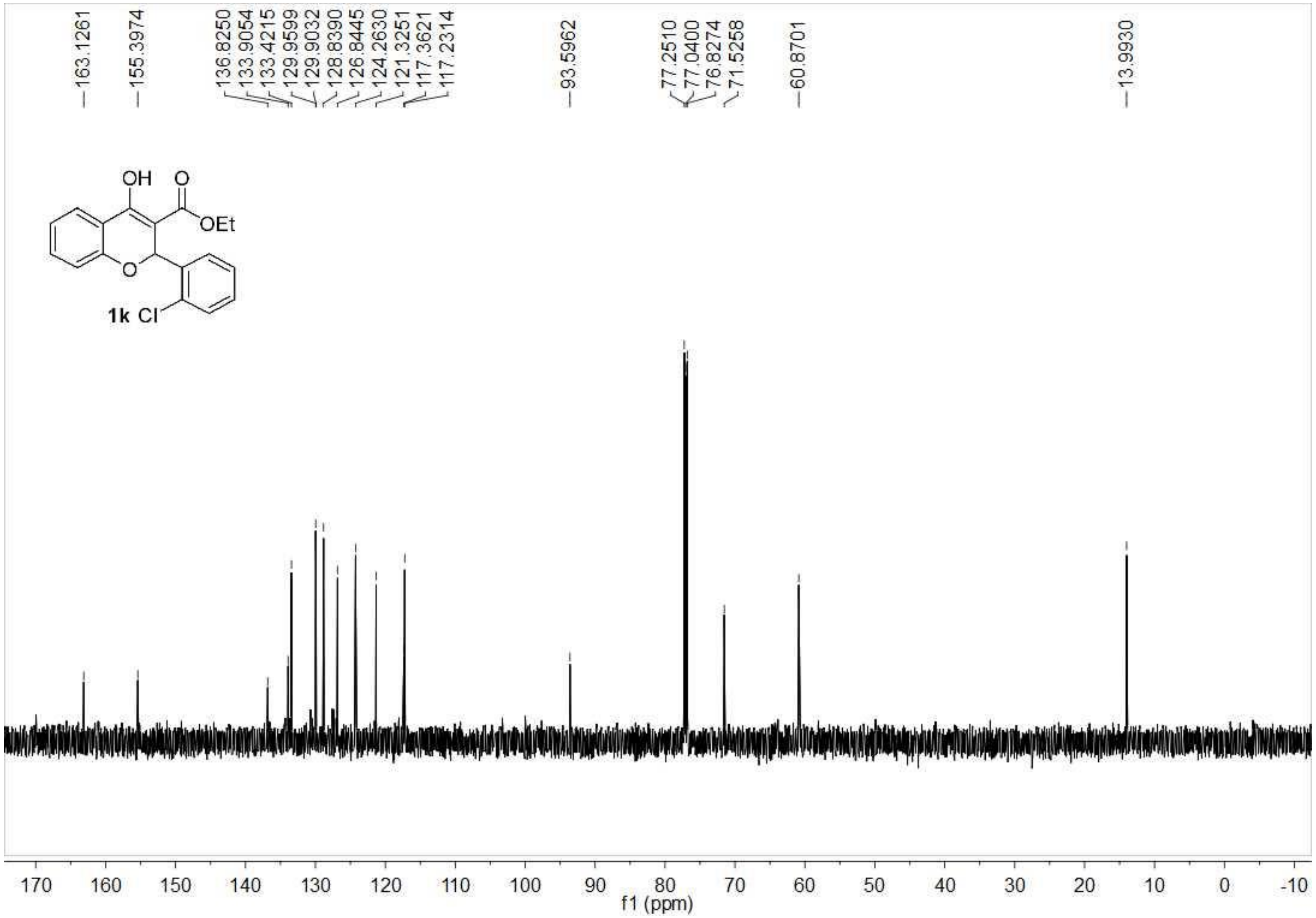

163.1261
155.3974
136.8250
133.9054
133.4215
129.9599
129.9032
128.8390
126.8445
124.2630
121.3251
117.3621
117.2314
93.5962
77.2510
77.0400
76.8274
71.5258
60.8701
13.9930
OH
O
OEt
O
1k Cl
170 160 150 140 130 120 110 100 90 80 70 60 50 40 30 20 10 0 -10
f1 (ppm)

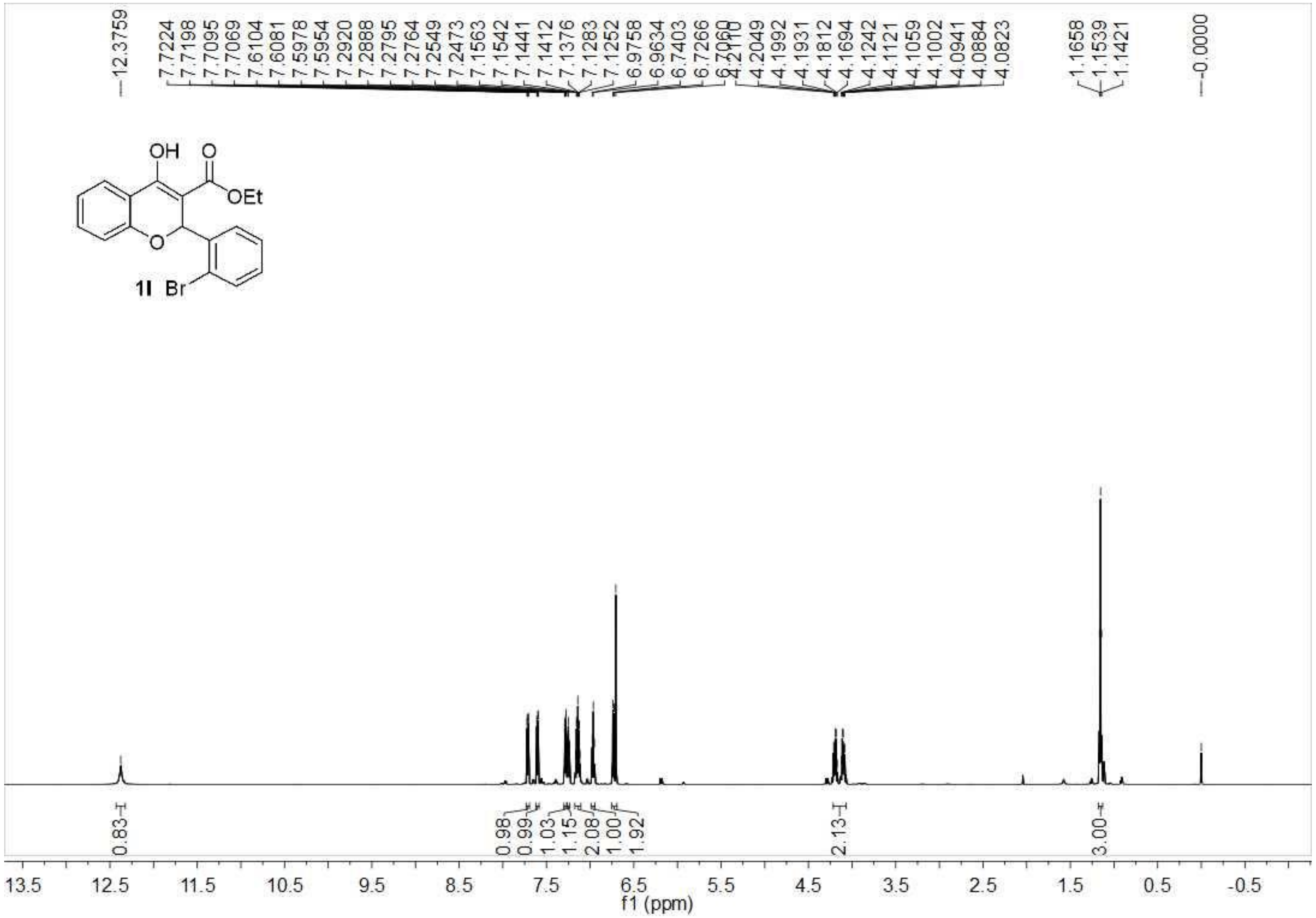
12.3759
7.7224
7.7198
7.7095
7.7069
7.6104
7.6081
7.5978
7.5954
7.2920
7.2888
7.2795
7.2764
7.2549
7.2473
7.1563
7.1542
7.1441
7.1412
7.1376
7.1283
7.1252
6.9758
6.9634
6.7403
6.7266
6.7060
4.2110
4.2049
4.1992
4.1931
4.1812
4.1694
4.1242
4.1121
4.1059
4.1002
4.0941
4.0884
4.0823
1.1658
1.1539
1.1421
-0.0000
OH
O
OEt
O
1l Br
0.83
0.98
0.99
1.03
1.15
2.08
1.00
1.92
2.13
3.00
13.5
12.5
11.5
10.5
9.5
8.5
7.5
6.5
5.5
4.5
3.5
2.5
1.5
0.5
-0.5
f1 (ppm)

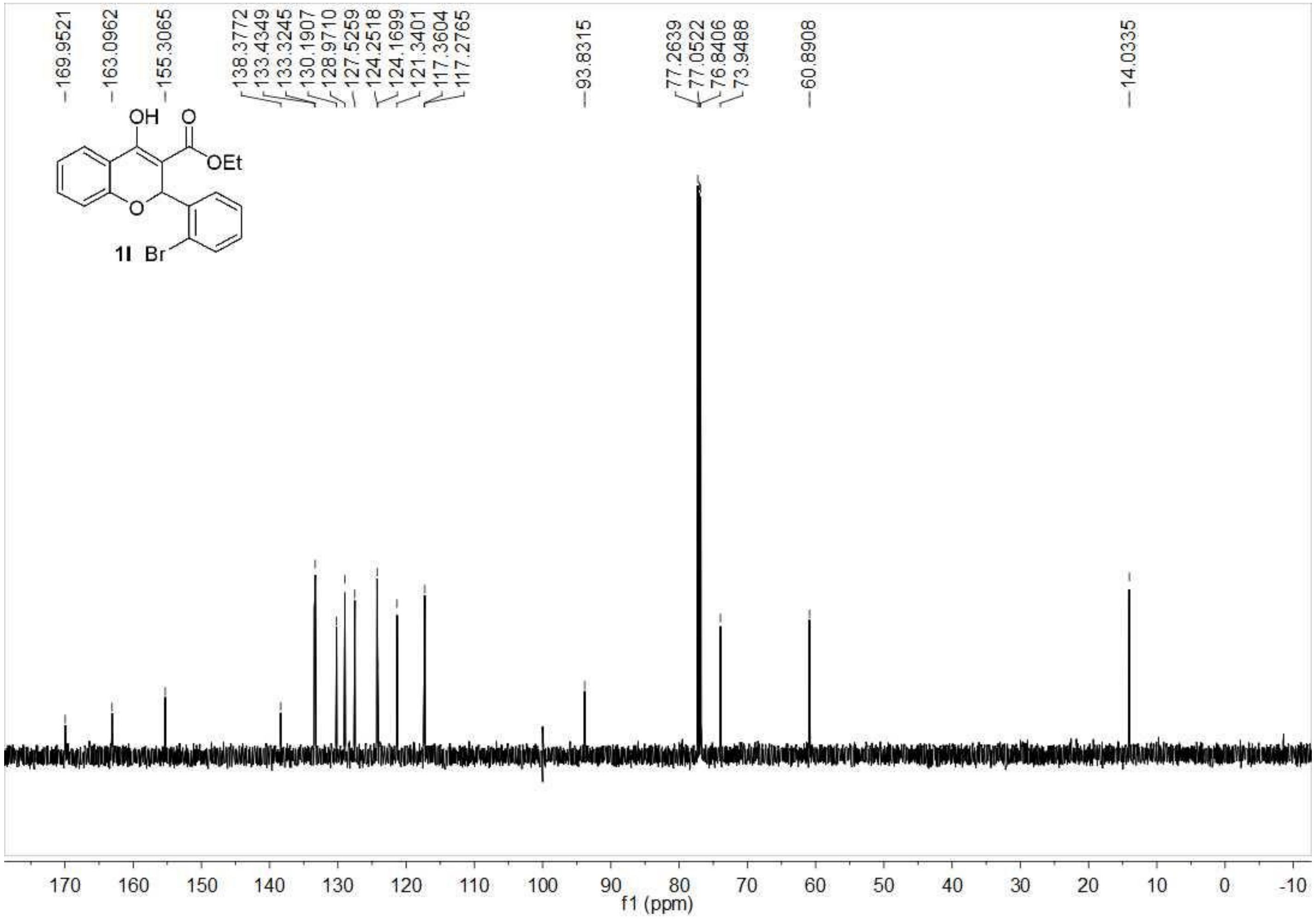

169.9521
163.0962
155.3065
138.3772
133.4349
133.3245
130.1907
128.9710
127.5259
124.2518
124.1699
121.3401
117.3604
117.2765
93.8315
77.2639
77.0522
76.8406
73.9488
60.8908
14.0335
OH
O
OEt
O
1l
Br
170 160 150 140 130 120 110 100 90 80 70 60 50 40 30 20 10 0 -10
f1 (ppm)

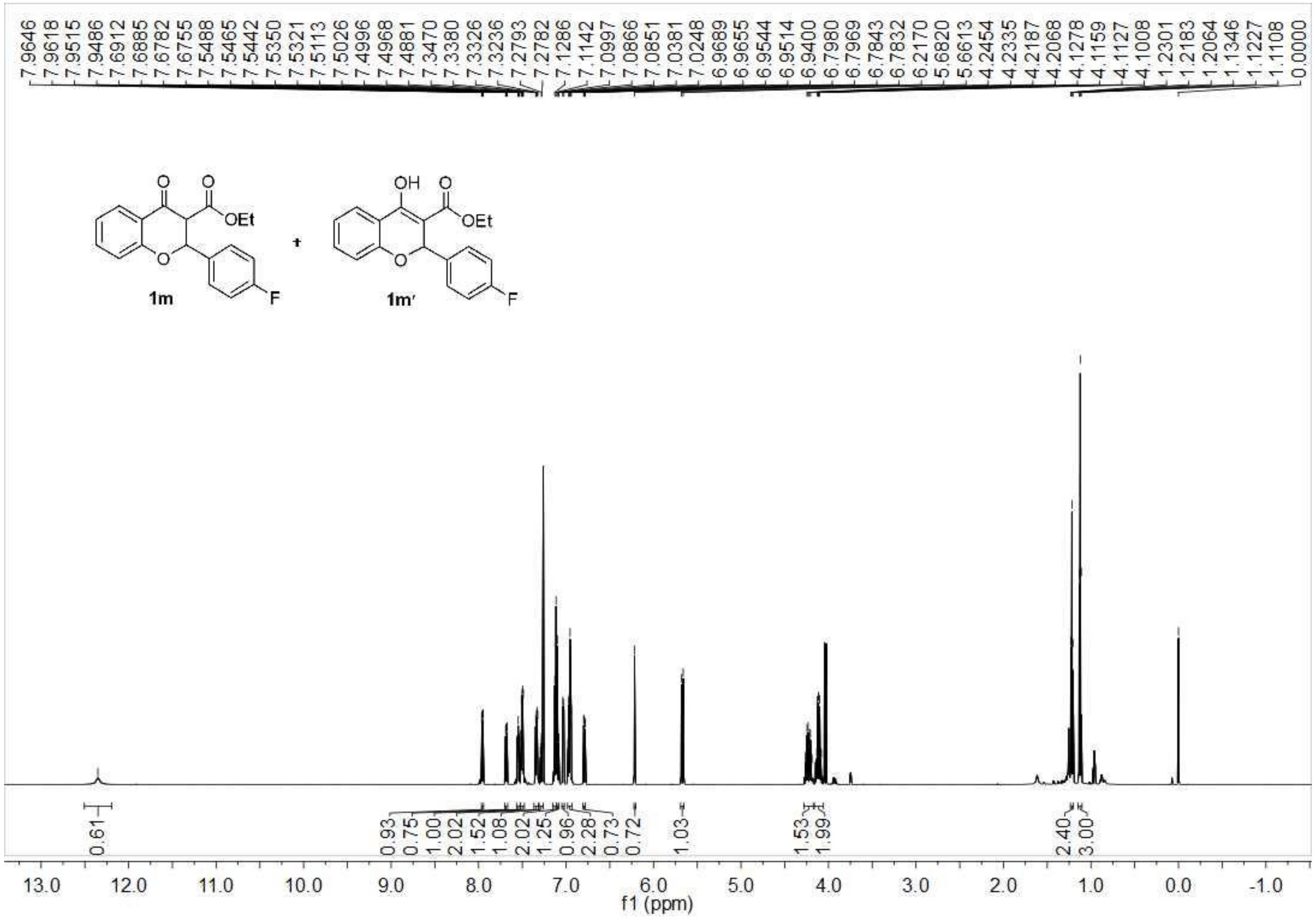

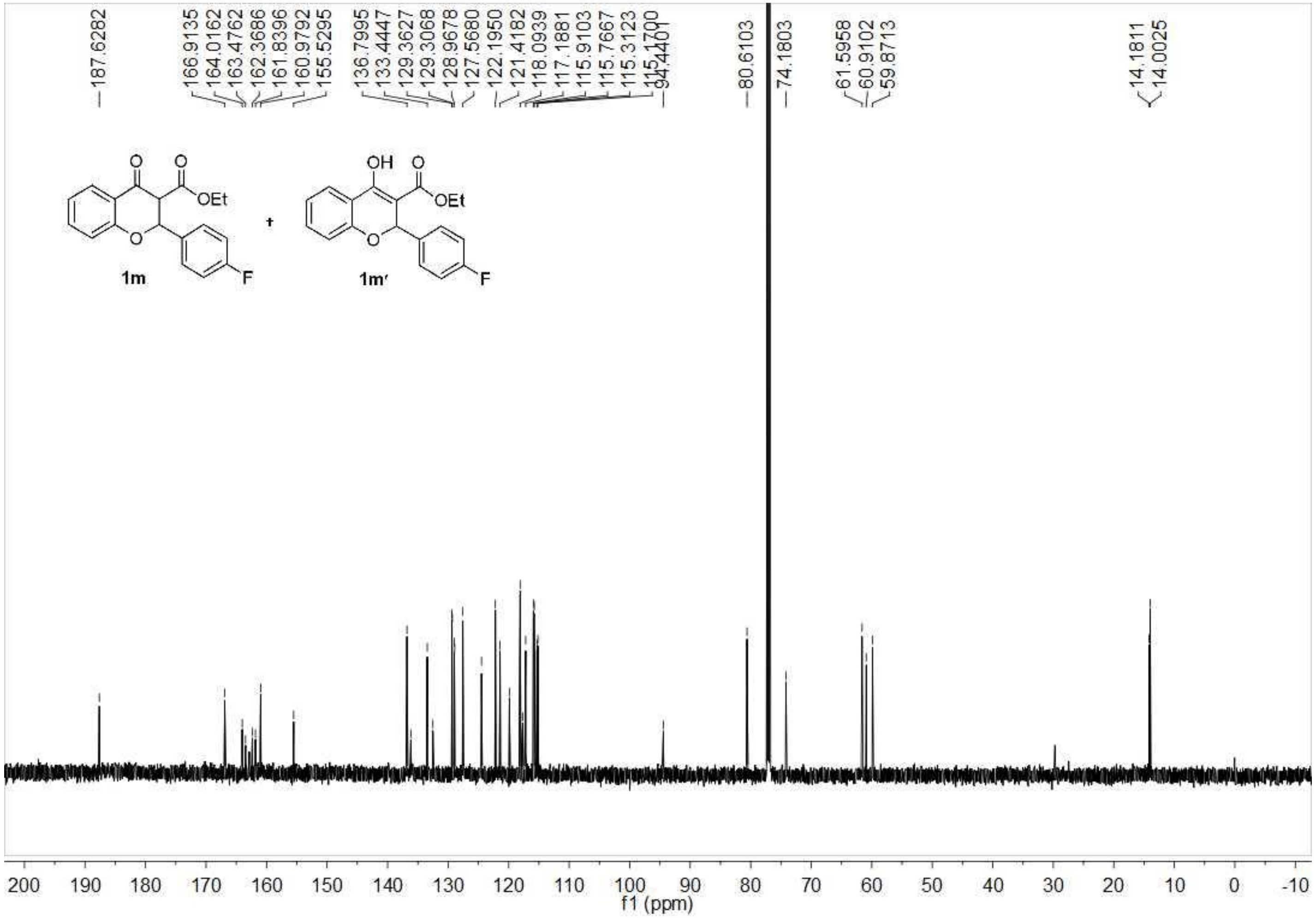

187.6282
166.9135
164.0162
163.4762
162.3686
161.8396
160.9792
155.5295
136.7995
133.4447
129.3627
129.3068
128.9678
127.5680
122.1950
121.4182
118.0939
117.1881
115.9103
115.7667
115.3123
115.1700
94.4401
80.6103
74.1803
61.5958
60.9102
59.8713
14.1811
14.0025
OH
OEt
O
F
1m
1m'
200
190
180
170
160
150
140
130
120
110
100
90
80
70
60
50
40
30
20
10
0
-10
f1 (ppm)

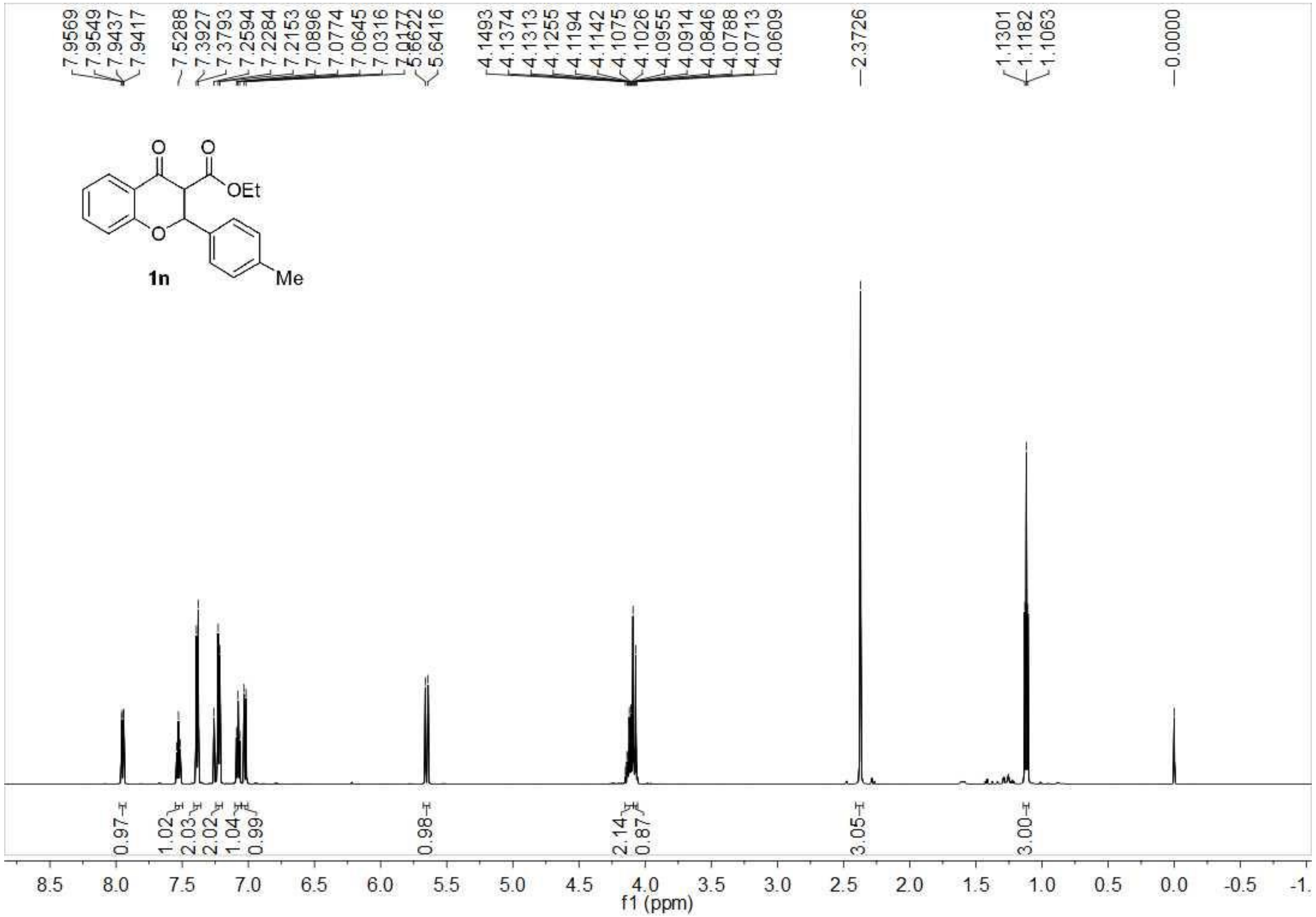

7.9569
7.9549
7.9437
7.9417
7.5288
7.3927
7.3793
7.2594
7.2284
7.2153
7.0896
7.0774
7.0645
7.0316
7.0177
5.6622
5.6416
4.1493
4.1374
4.1313
4.1255
4.1194
4.1142
4.1075
4.1026
4.0955
4.0914
4.0846
4.0788
4.0713
4.0609
2.3726
1.1301
1.1182
1.1063
0.0000
OEt
Me
1n
0.97
1.02
2.03
2.02
1.04
0.99
0.98
2.14
0.87
3.05
3.00
8.5
8.0
7.5
7.0
6.5
6.0
5.5
5.0
4.5
4.0
3.5
3.0
2.5
2.0
1.5
1.0
0.5
0.0
-0.5
-1.
f1 (ppm)

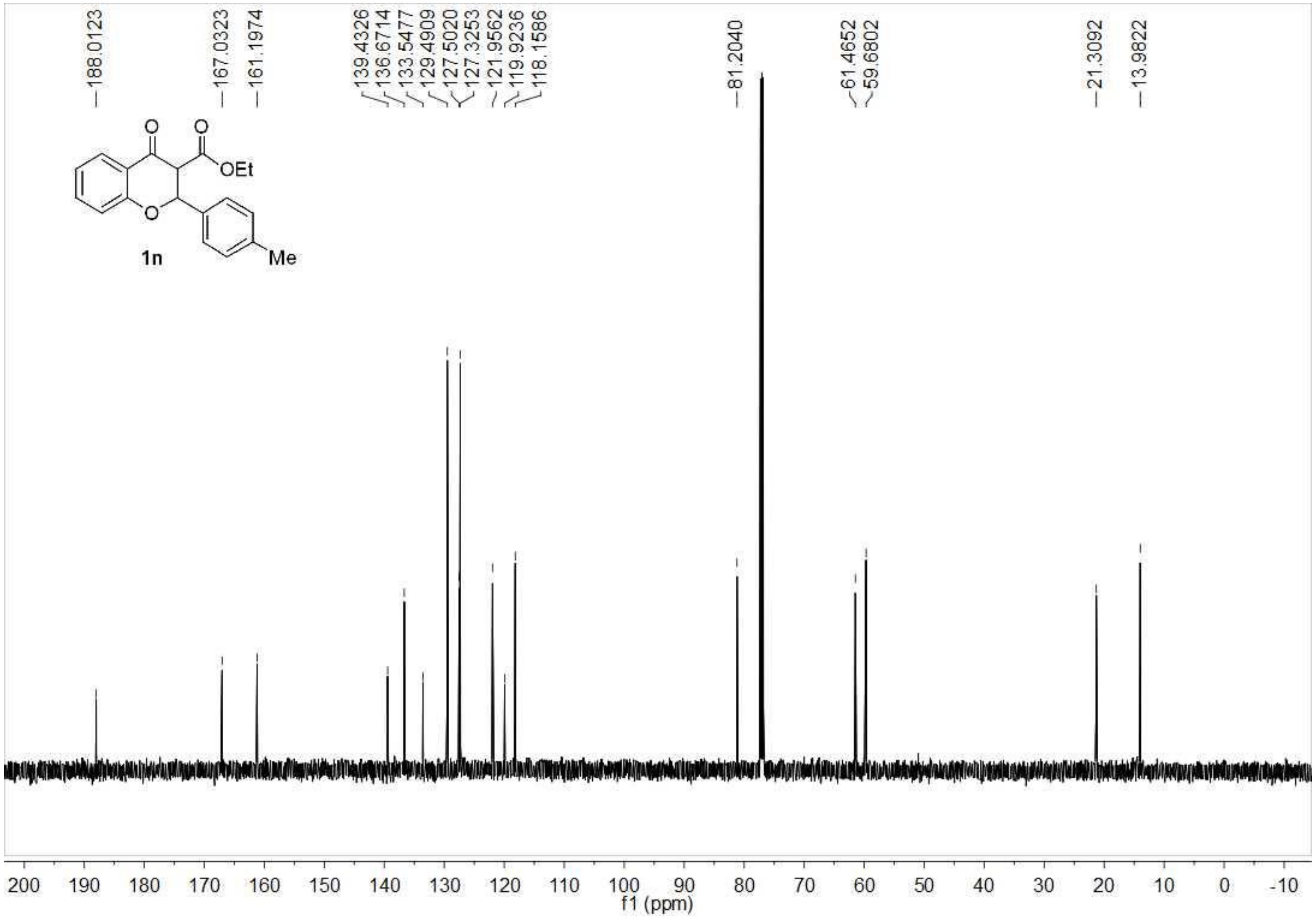
188.0123
167.0323
161.1974
139.4326
136.6714
133.5477
129.4909
127.5020
127.3253
121.9562
119.9236
118.1586
81.2040
61.4652
59.6802
21.3092
13.9822
OEt
Me
1n
200 190 180 170 160 150 140 130 120 110 100 90 80 70 60 50 40 30 20 10 0 -10
f1 (ppm)

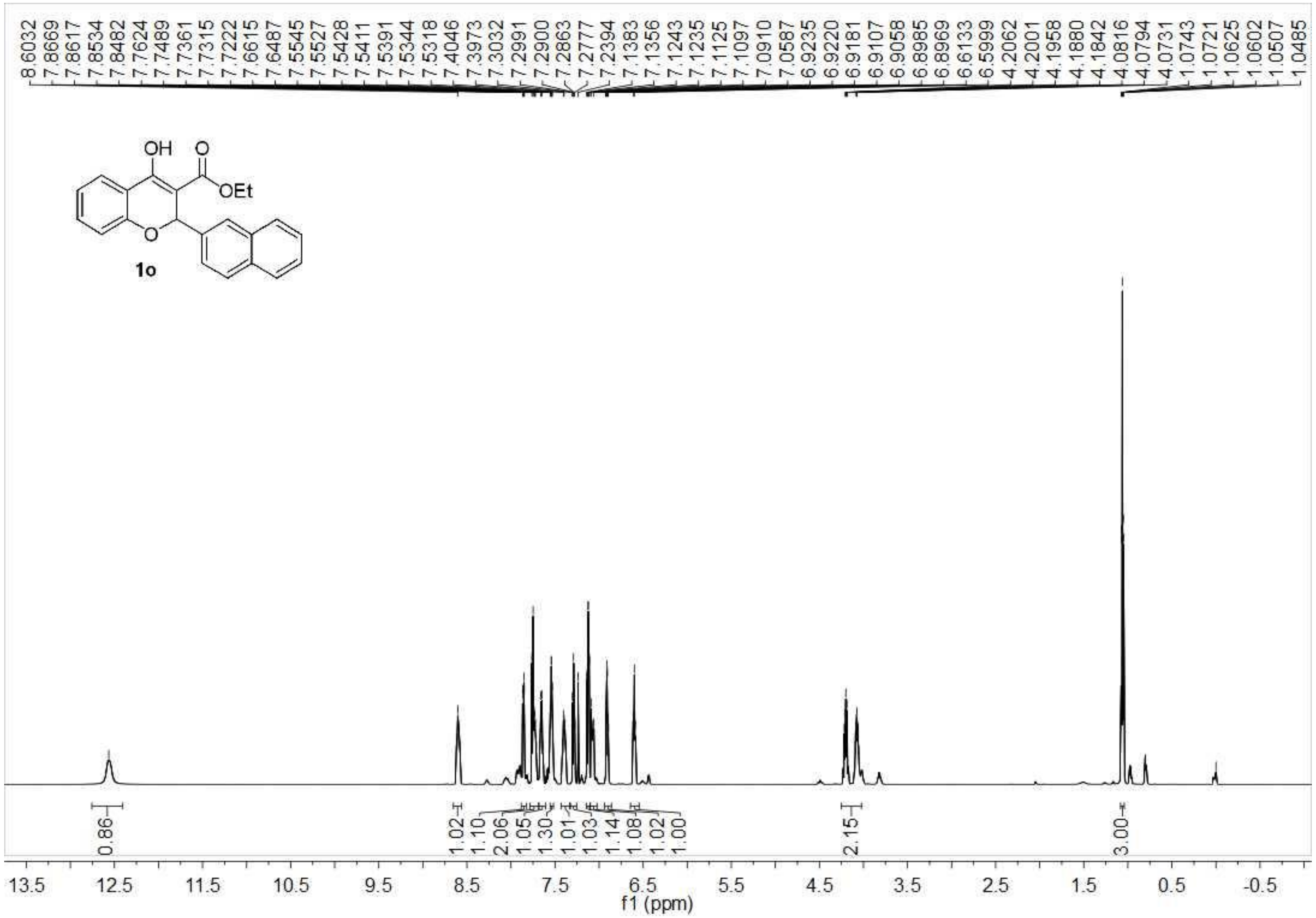
1o
OH
O
OEt
O
f1 (ppm)

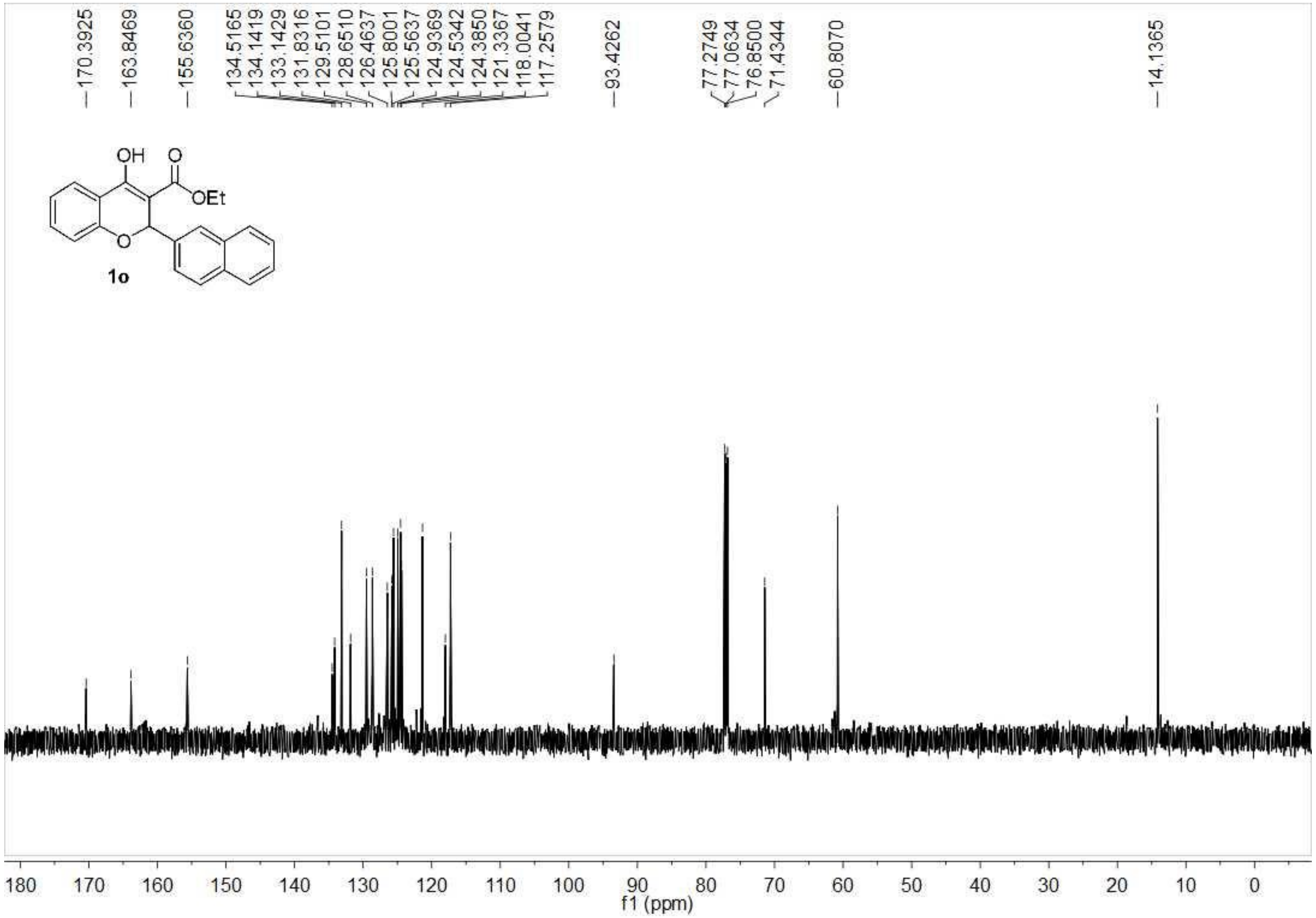
170.3925
163.8469
155.6360
134.5165
134.1419
133.1429
131.8316
129.5101
128.6510
126.4637
125.8001
125.5637
124.9369
124.5342
124.3850
121.3367
118.0041
117.2579
93.4262
77.2749
77.0634
76.8500
71.4344
60.8070
14.1365
OH
O
OEt
O
1o
180 170 160 150 140 130 120 110 100 90 80 70 60 50 40 30 20 10 0
f1 (ppm)

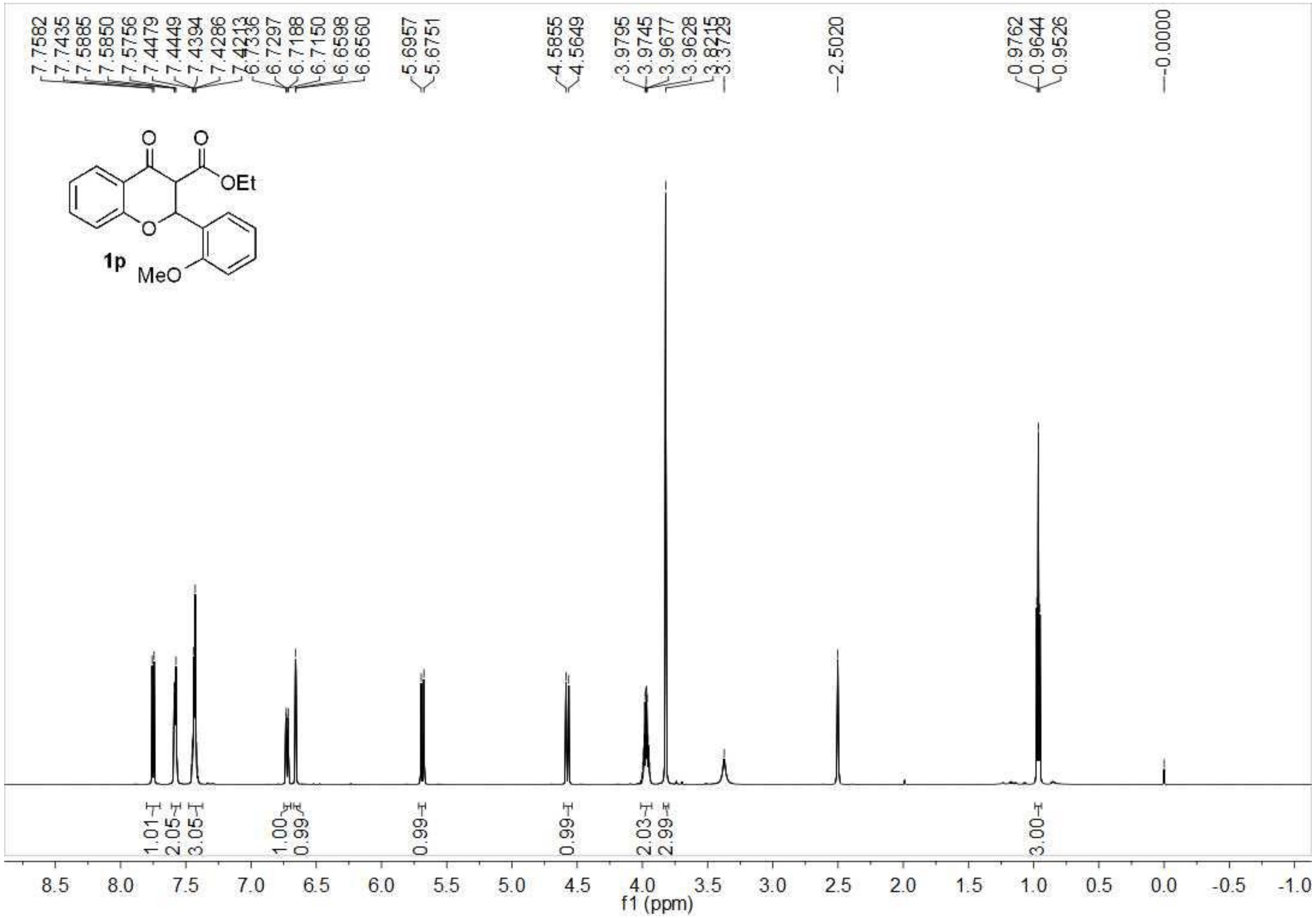
7.7582
7.7435
7.5885
7.5850
7.5756
7.4479
7.4449
7.4394
7.4286
7.4213
6.7336
6.7297
6.7188
6.7150
6.6598
6.6560
5.6957
5.6751
4.5855
4.5649
3.9795
3.9745
3.9677
3.9628
3.8215
3.3729
2.5020
0.9762
0.9644
0.9526
0.0000
OEt
1p
MeO
1.01
2.05
3.05
1.00
0.99
0.99
0.99
2.03
2.99
3.00
8.5 8.0 7.5 7.0 6.5 6.0 5.5 5.0 4.5 4.0 3.5 3.0 2.5 2.0 1.5 1.0 0.5 0.0 -0.5 -1.0
f1 (ppm)

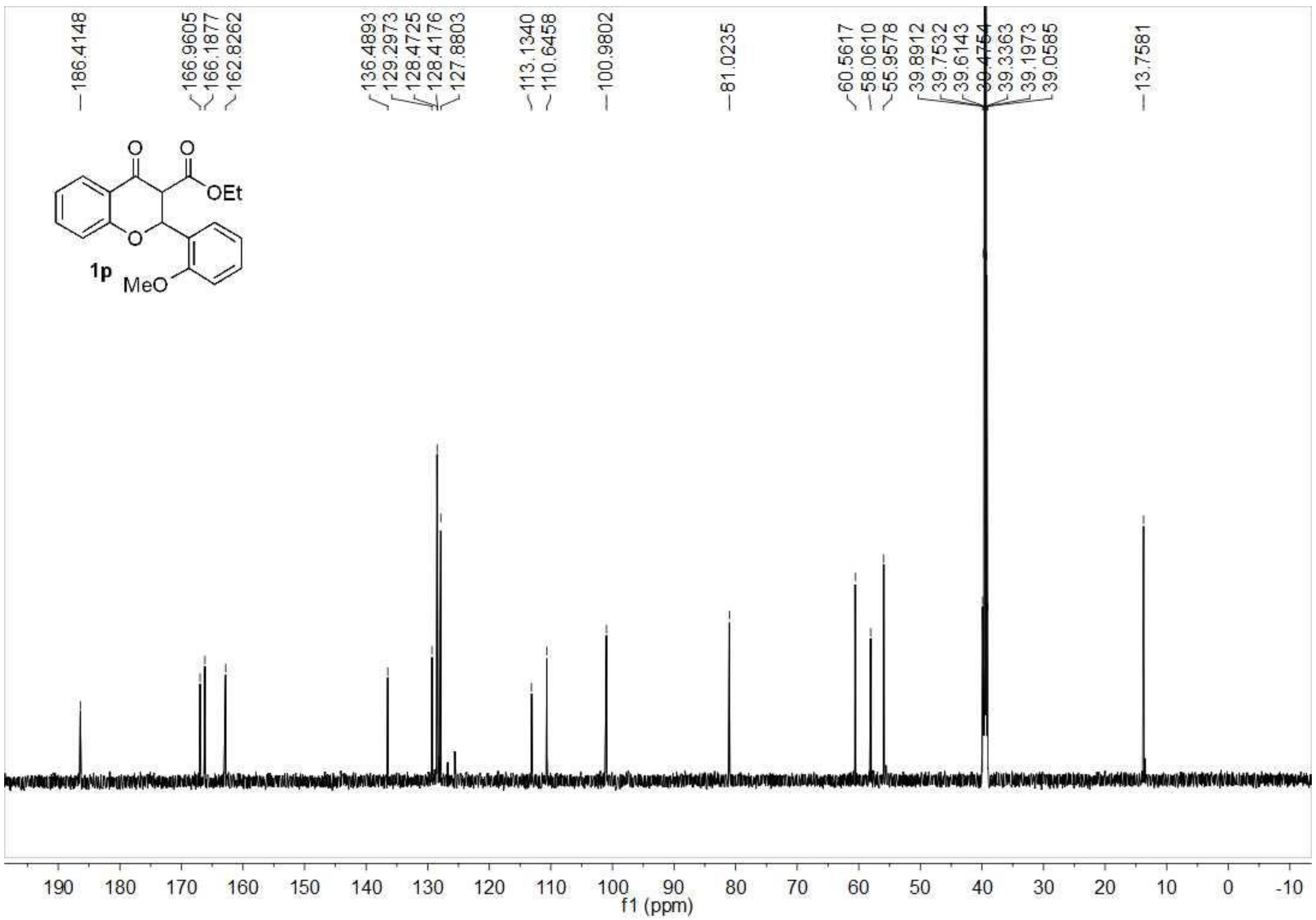
186.4148
166.9605
166.1877
162.8262
136.4893
129.2973
128.4725
128.4176
127.8803
113.1340
110.6458
100.9802
81.0235
60.5617
58.0610
55.9578
39.8912
39.7532
39.6143
39.4754
39.3363
39.1973
39.0585
13.7581
OEt
MeO
1p
190 180 170 160 150 140 130 120 110 100 90 80 70 60 50 40 30 20 10 0 -10
f1 (ppm)

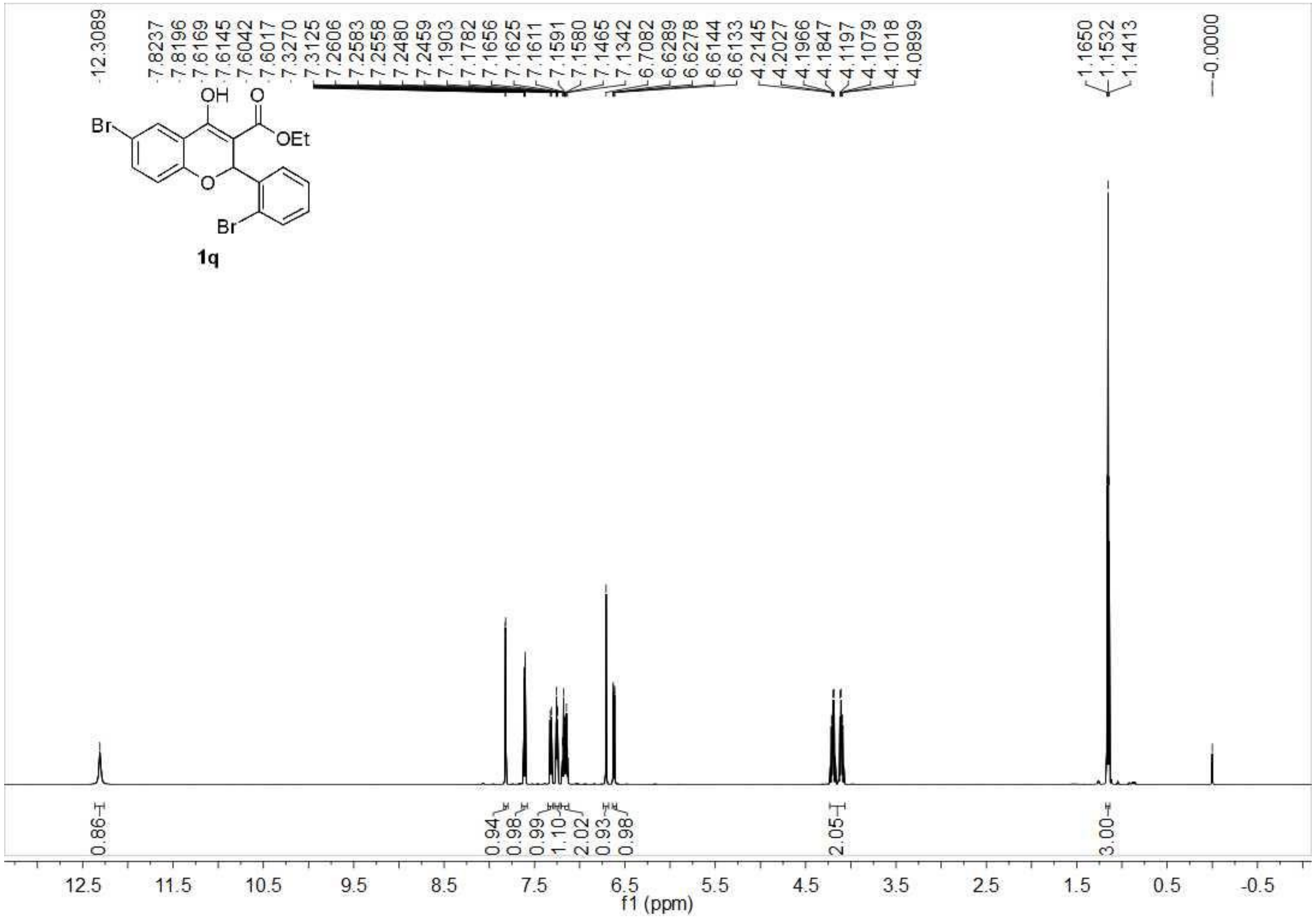

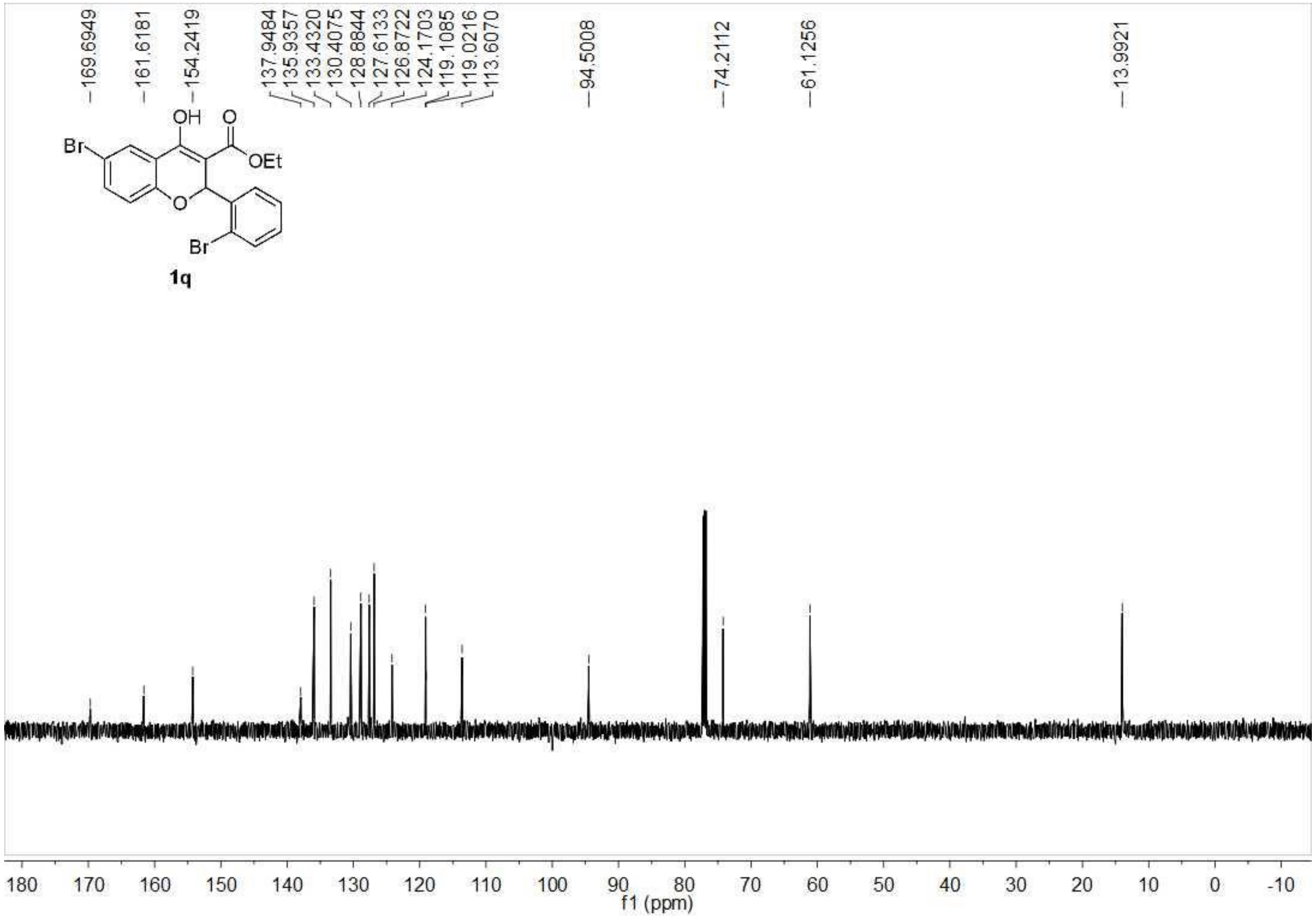
169.6949
161.6181
154.2419
137.9484
135.9357
133.4320
130.4075
128.8844
127.6133
126.8722
124.1703
119.1085
119.0216
113.6070
94.5008
74.2112
61.1256
13.9921
OH
O
OEt
Br
O
Br
1q
180
170
160
150
140
130
120
110
100
90
80
70
60
50
40
30
20
10
0
-10
f1 (ppm)

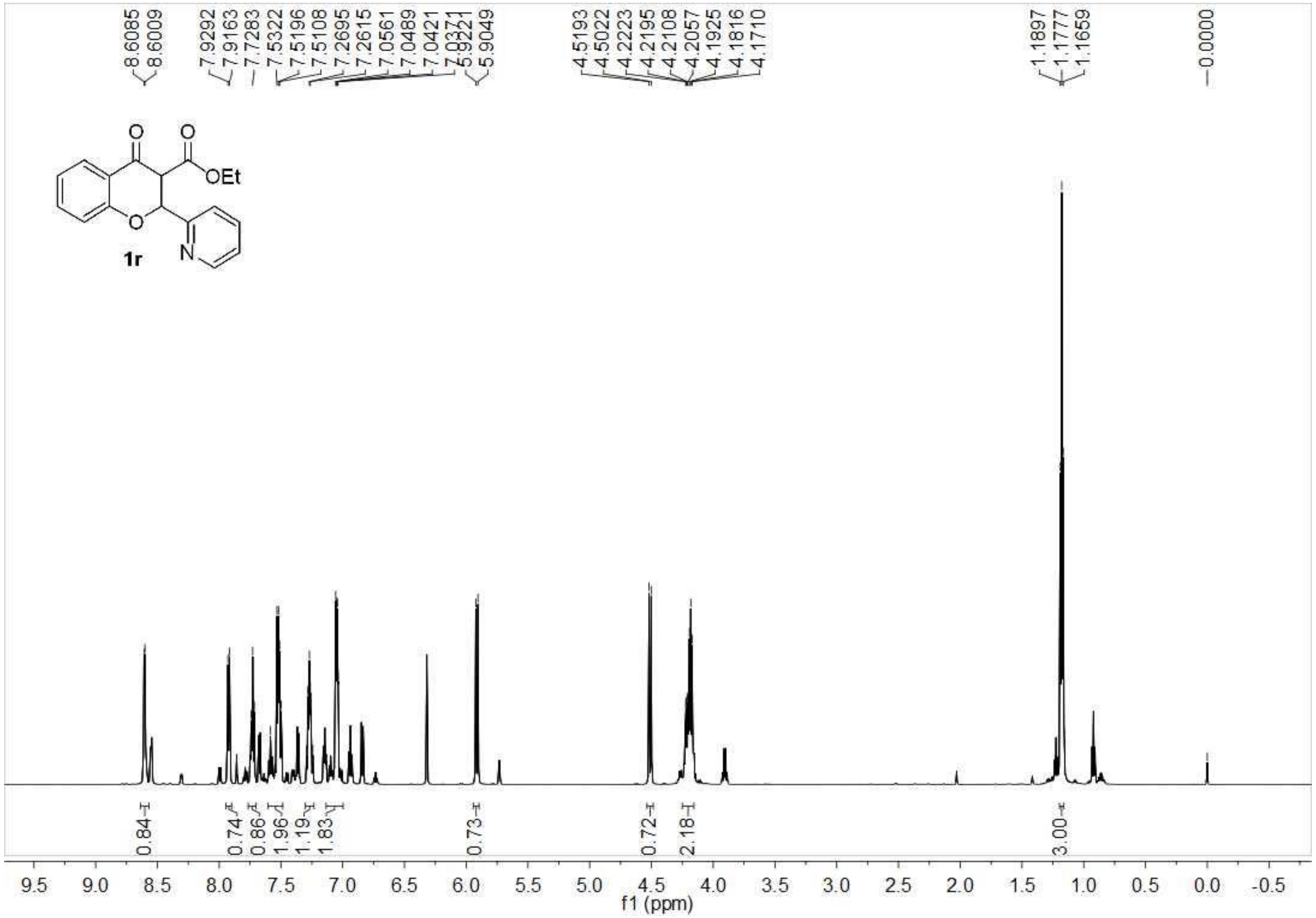

8.6085
8.6009
7.9292
7.9163
7.7283
7.5322
7.5196
7.5108
7.2695
7.2615
7.0561
7.0489
7.0421
7.0371
5.9221
5.9049
4.5193
4.5022
4.2223
4.2195
4.2108
4.2057
4.1925
4.1816
4.1710
1.1897
1.1777
1.1659
0.0000
OEt
1r
0.84
0.74
0.86
1.96
1.19
1.83
0.73
0.72
2.18
3.00
9.5 9.0 8.5 8.0 7.5 7.0 6.5 6.0 5.5 5.0 4.5 4.0 3.5 3.0 2.5 2.0 1.5 1.0 0.5 0.0 -0.5
f1 (ppm)

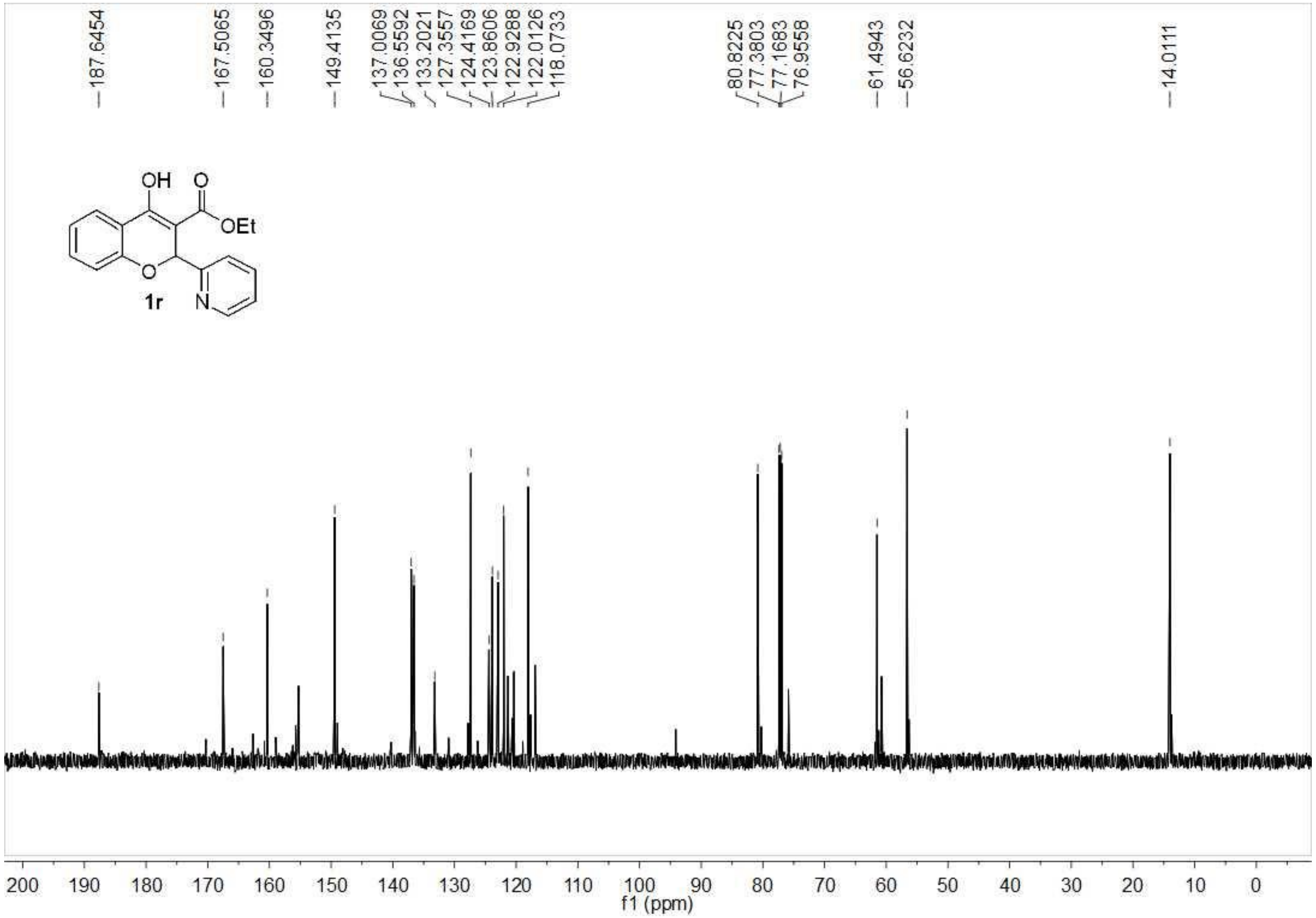
187.6454
167.5065
160.3496
149.4135
137.0069
136.5592
133.2021
127.3557
124.4169
123.8606
122.9288
122.0126
118.0733
80.8225
77.3803
77.1683
76.9558
61.4943
56.6232
14.0111
OH
O
OEt
O
N
1r
200 190 180 170 160 150 140 130 120 110 100 90 80 70 60 50 40 30 20 10 0
f1 (ppm)

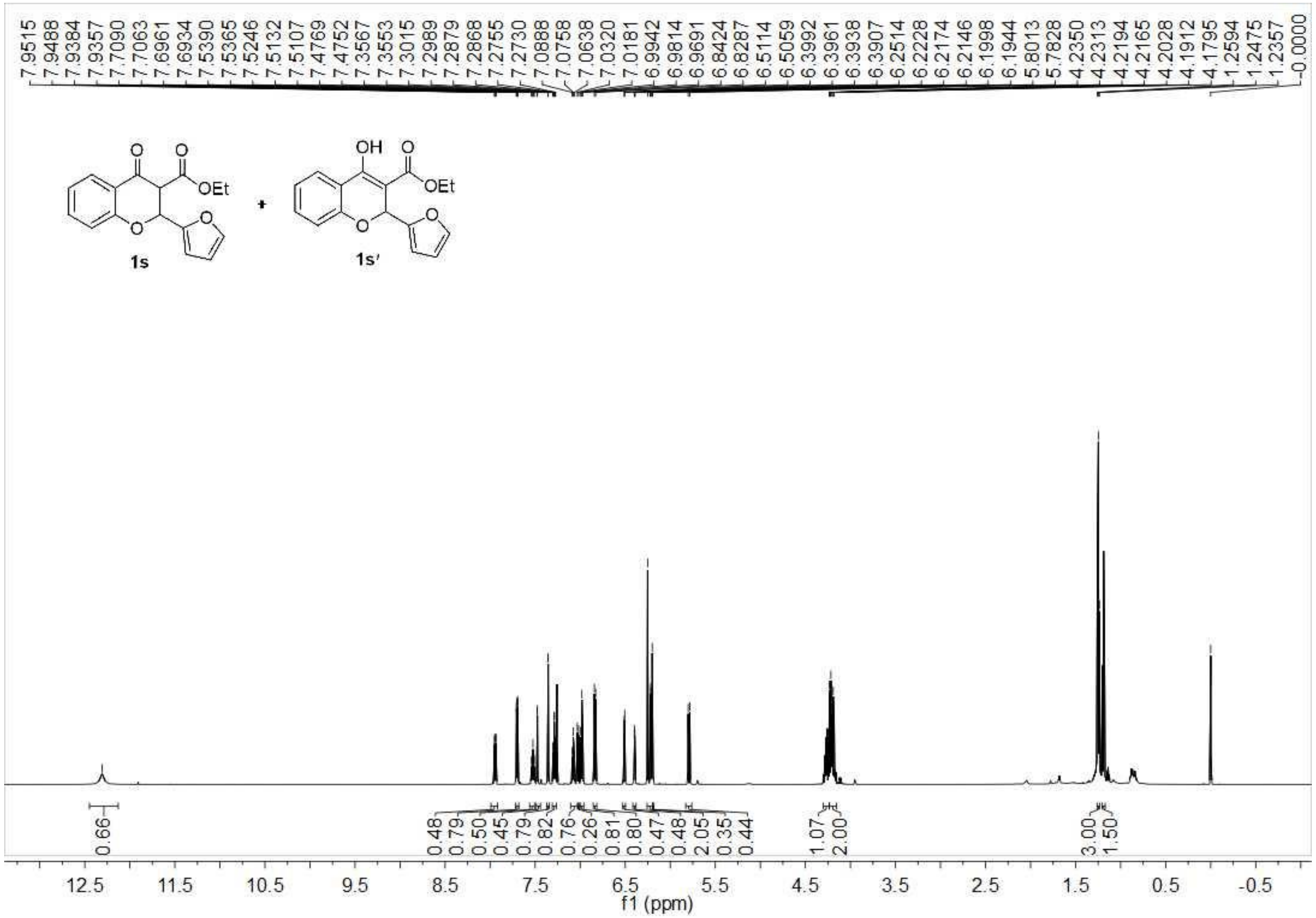
7.9515
7.9488
7.9384
7.9357
7.7090
7.7063
7.6961
7.6934
7.5390
7.5365
7.5246
7.5132
7.5107
7.4769
7.4752
7.3567
7.3553
7.3015
7.2989
7.2879
7.2868
7.2755
7.2730
7.0888
7.0758
7.0638
7.0320
7.0181
6.9942
6.9814
6.9691
6.8424
6.8287
6.5114
6.5059
6.3992
6.3961
6.3938
6.3907
6.2514
6.2228
6.2174
6.2146
6.1998
6.1944
5.8013
5.7828
4.2350
4.2313
4.2194
4.2165
4.2028
4.1912
4.1795
1.2594
1.2475
1.2357
-0.0000
O
O
OEt
O
O
1s
+
OH
O
OEt
O
O
1s′
0.66
0.48
0.79
0.50
0.45
0.79
0.82
0.76
0.26
0.81
0.80
0.47
0.48
2.05
0.35
0.44
1.07
2.00
3.00
1.50
12.5
11.5
10.5
9.5
8.5
7.5
6.5
5.5
4.5
3.5
2.5
1.5
0.5
-0.5
f1 (ppm)

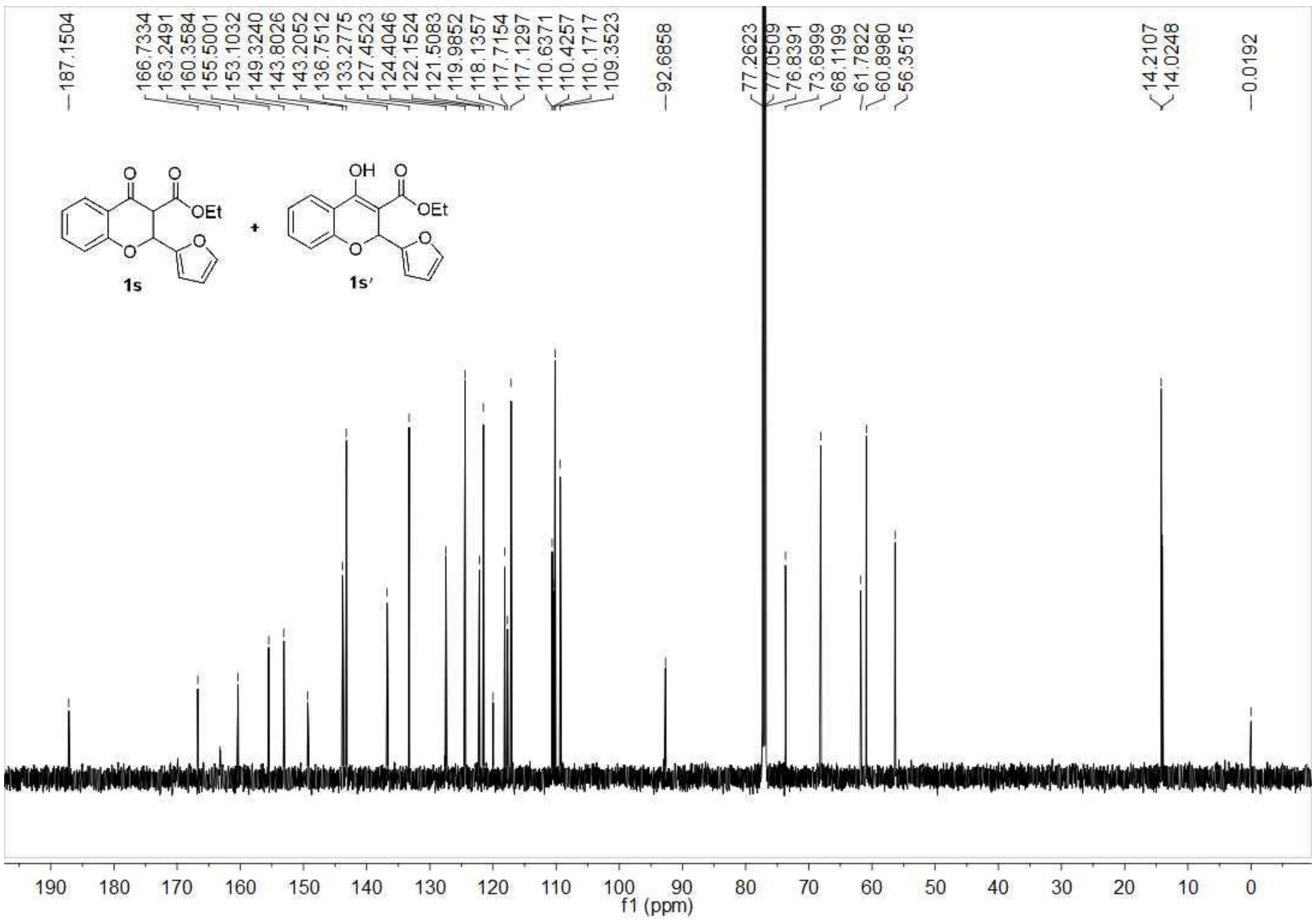
187.1504
166.7334
163.2491
160.3584
155.5001
153.1032
149.3240
143.8026
143.2052
136.7512
133.2775
127.4523
124.4046
122.1524
121.5083
119.9852
118.1357
117.7154
117.1297
110.6371
110.4257
110.1717
109.3523
92.6858
77.2623
77.0509
76.8391
73.6999
68.1199
61.7822
60.8980
56.3515
14.2107
14.0248
-0.0192
OEt
OH
1s
1s'
f1 (ppm)

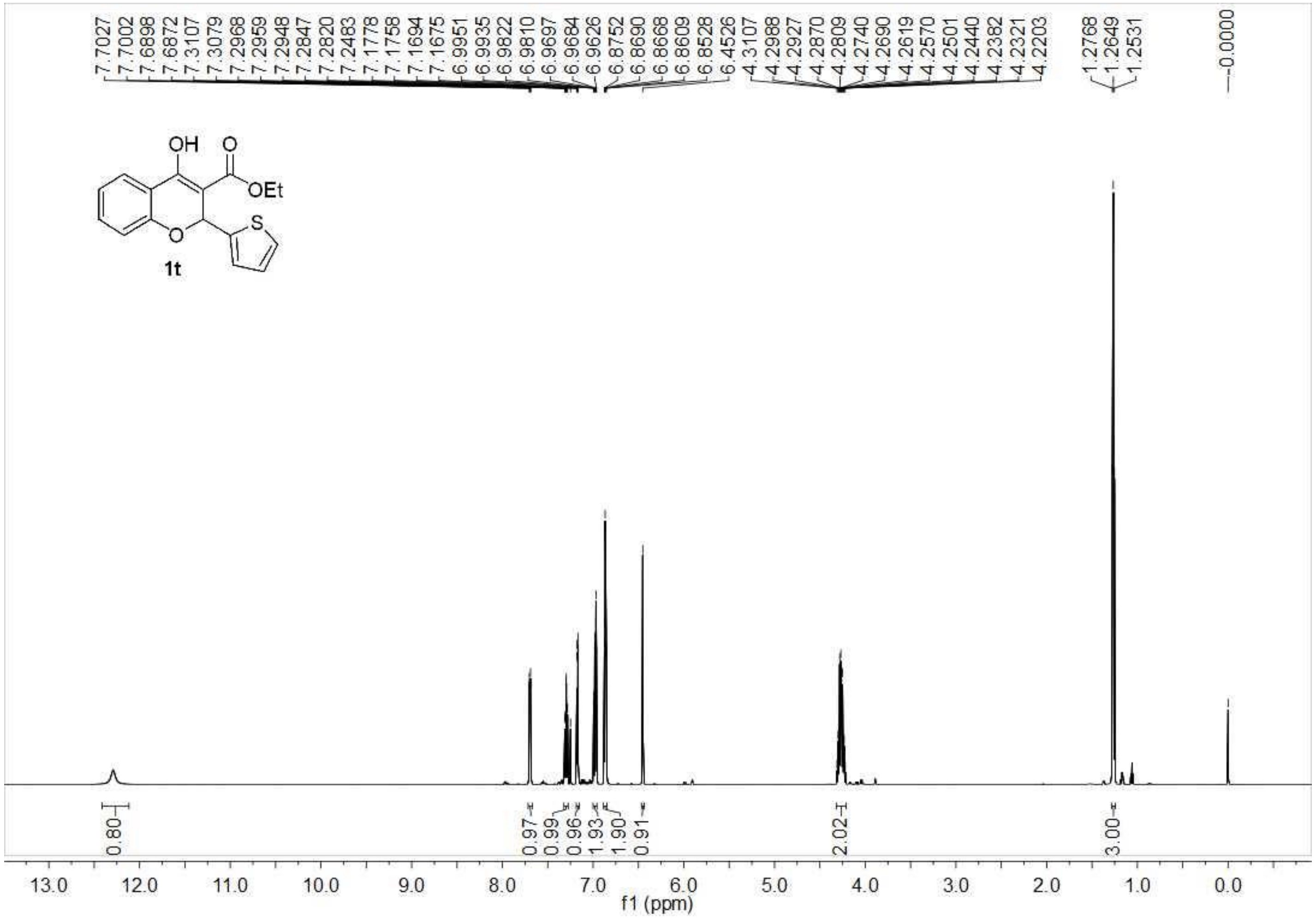
OH
O
OEt
O
S
1t
f1 (ppm)

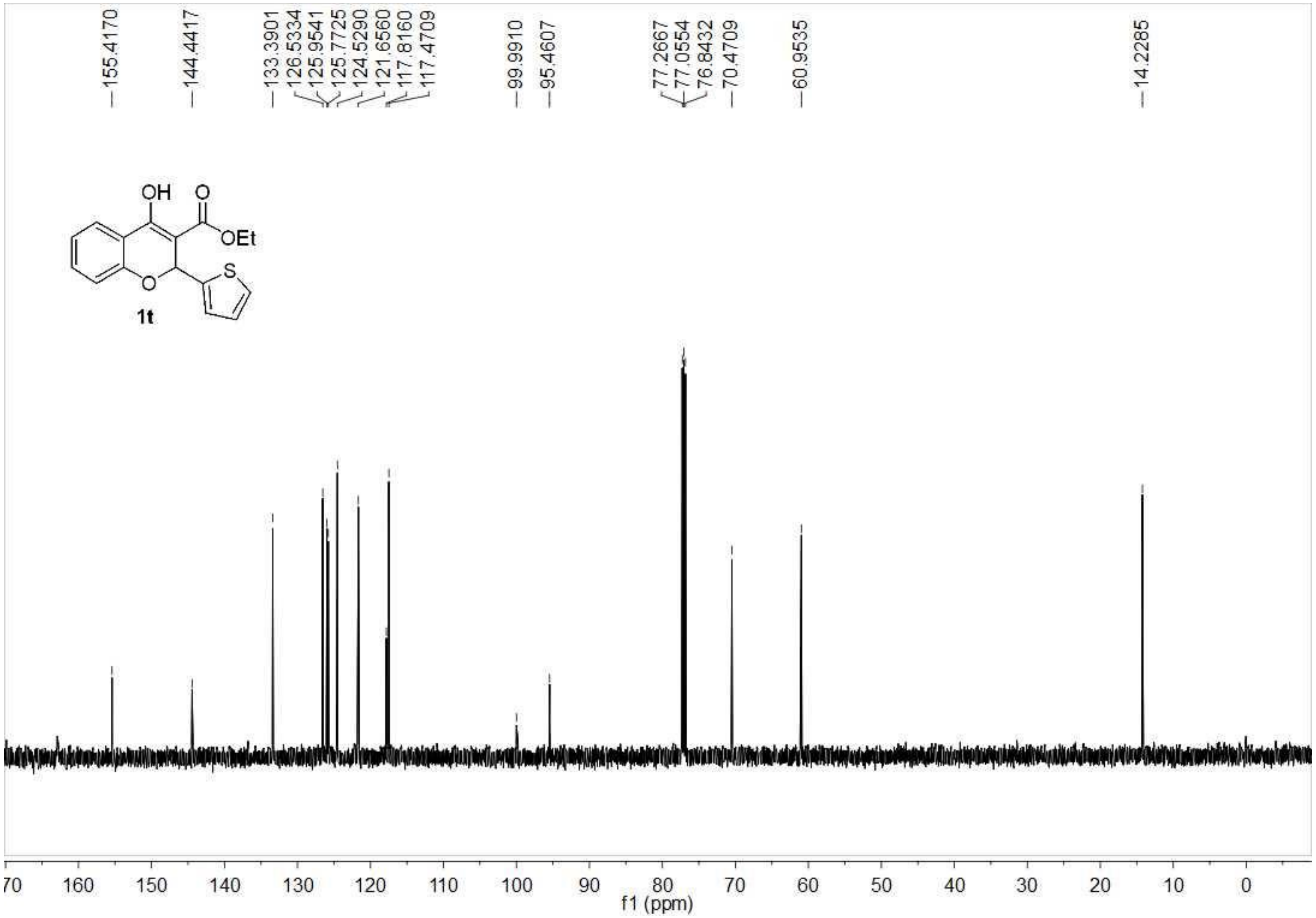
155.4170
144.4417
133.3901
126.5334
125.9541
125.7725
124.5290
121.6560
117.8160
117.4709
99.9910
95.4607
77.2667
77.0554
76.8432
70.4709
60.9535
14.2285
OH
O
OEt
O
S
1t
70
160
150
140
130
120
110
100
90
80
70
60
50
40
30
20
10
0
f1 (ppm)

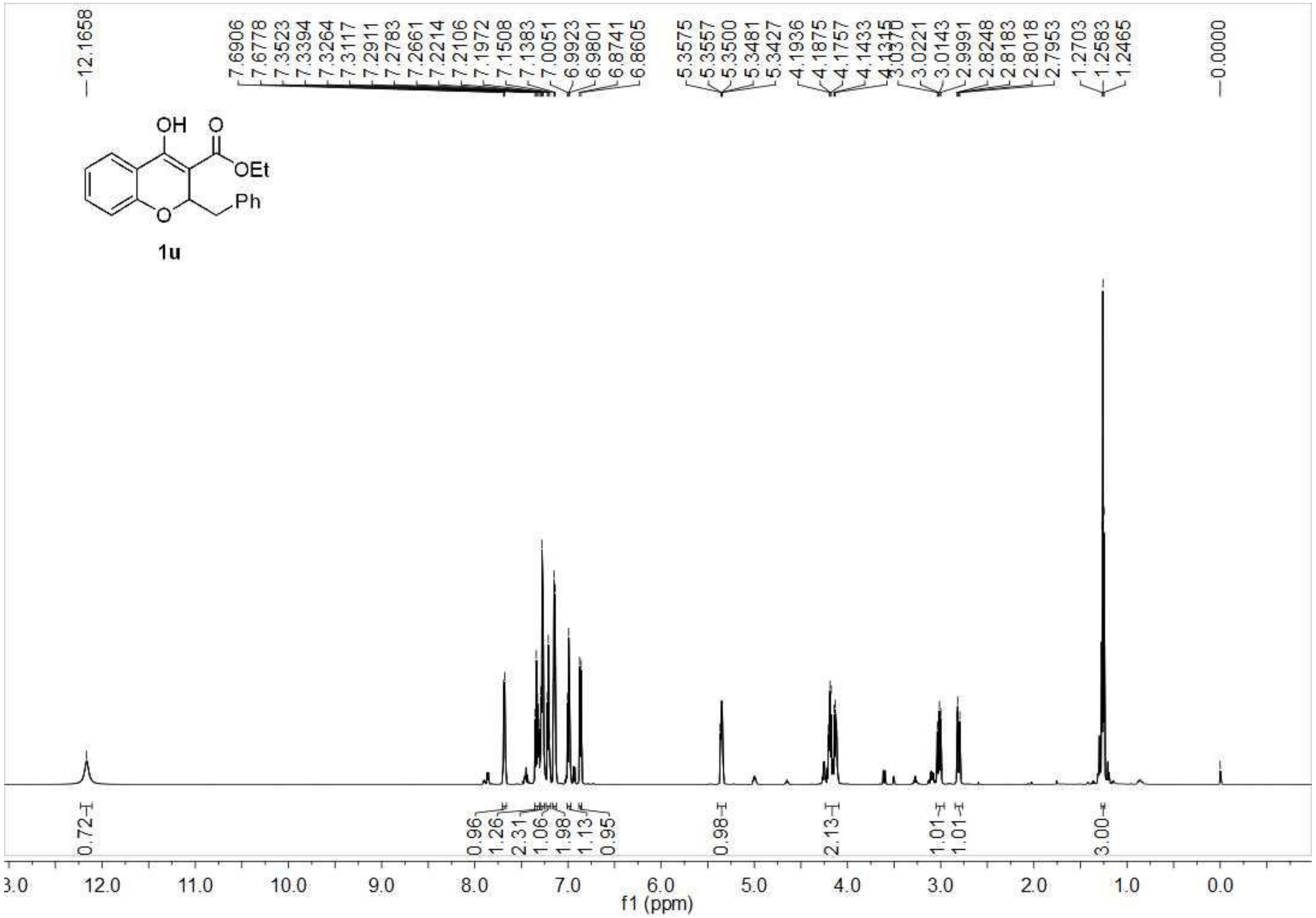

12.1658
7.6906
7.6778
7.3523
7.3394
7.3264
7.3117
7.2911
7.2783
7.2661
7.2214
7.2106
7.1972
7.1508
7.1383
7.0051
6.9923
6.9801
6.8741
6.8605
5.3575
5.3557
5.3500
5.3481
5.3427
4.1936
4.1875
4.1757
4.1433
4.1315
3.0370
3.0221
3.0143
2.9991
2.8248
2.8183
2.8018
2.7953
1.2703
1.2583
1.2465
0.0000
OH
O
OEt
O
Ph
1u
0.72
0.96
1.26
2.31
1.06
1.98
1.13
0.95
0.98
2.13
1.01
1.01
3.00
13.0
12.0
11.0
10.0
9.0
8.0
7.0
6.0
5.0
4.0
3.0
2.0
1.0
0.0
f1 (ppm)

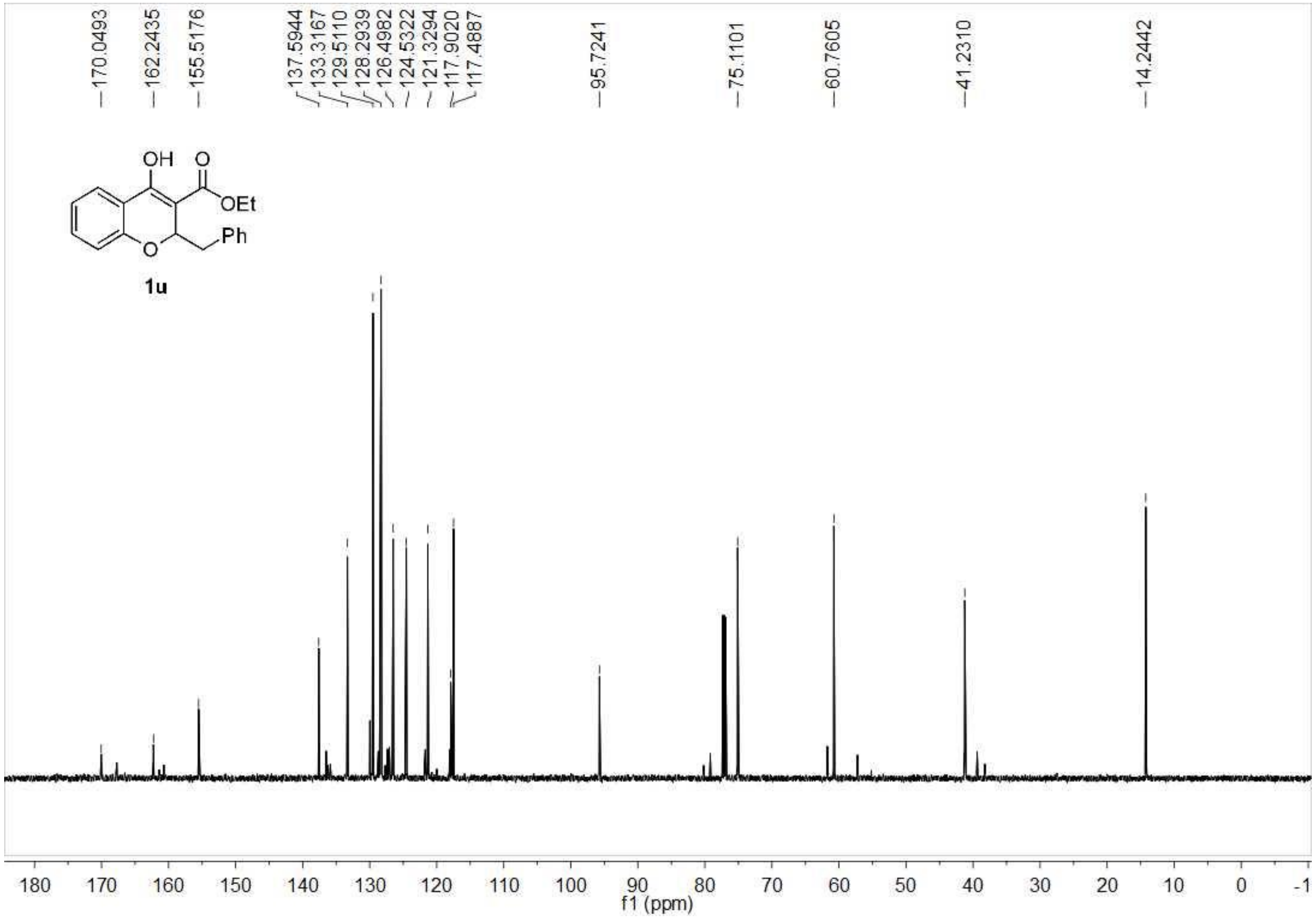
170.0493
162.2435
155.5176
137.5944
133.3167
129.5110
128.2939
126.4982
124.5322
121.3294
117.9020
117.4887
95.7241
75.1101
60.7605
41.2310
14.2442
OH
O
OEt
O
Ph
1u
180 170 160 150 140 130 120 110 100 90 80 70 60 50 40 30 20 10 0 -1
f1 (ppm)

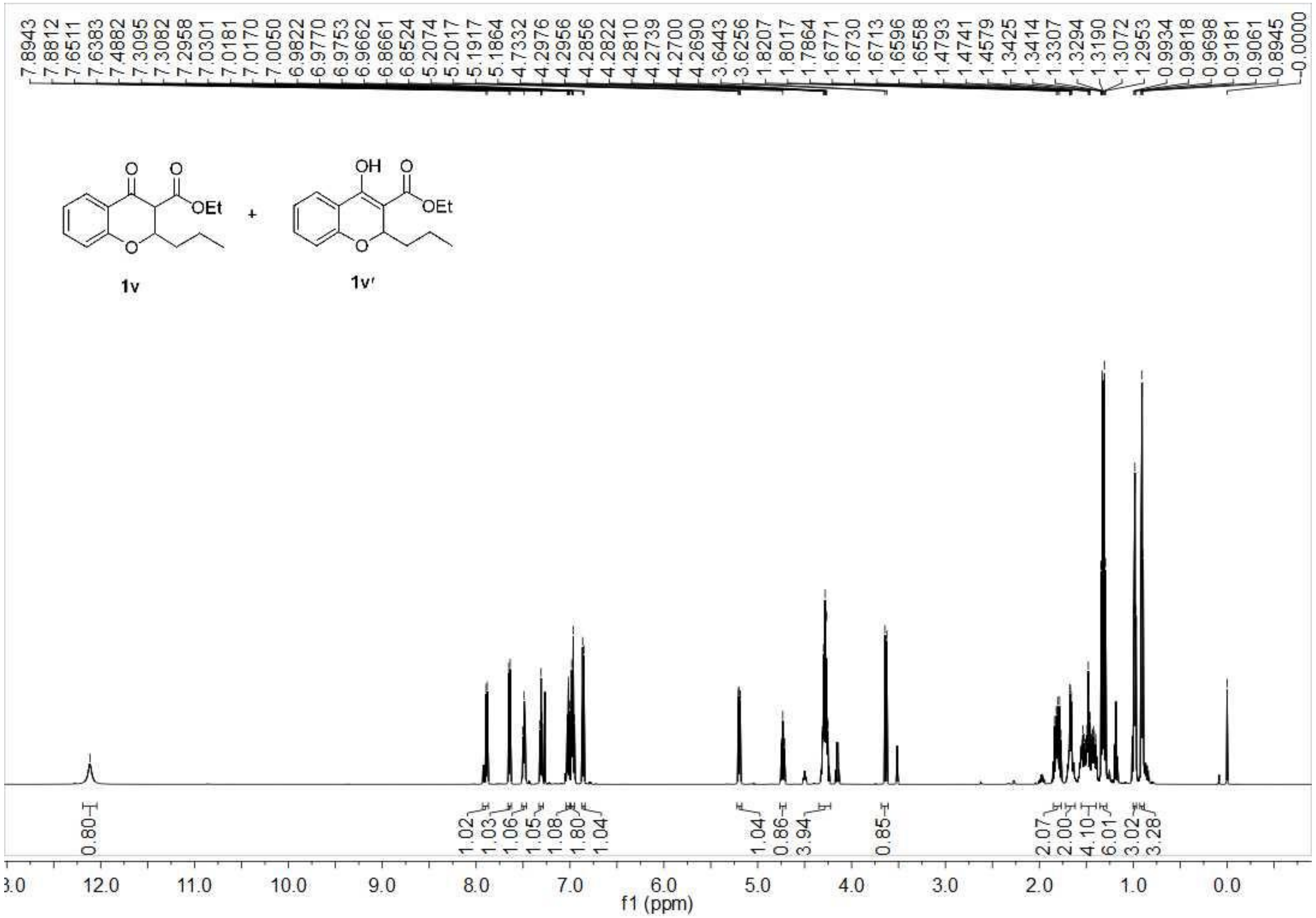
OEt
OEt
OH
1v
1v'
f1 (ppm)

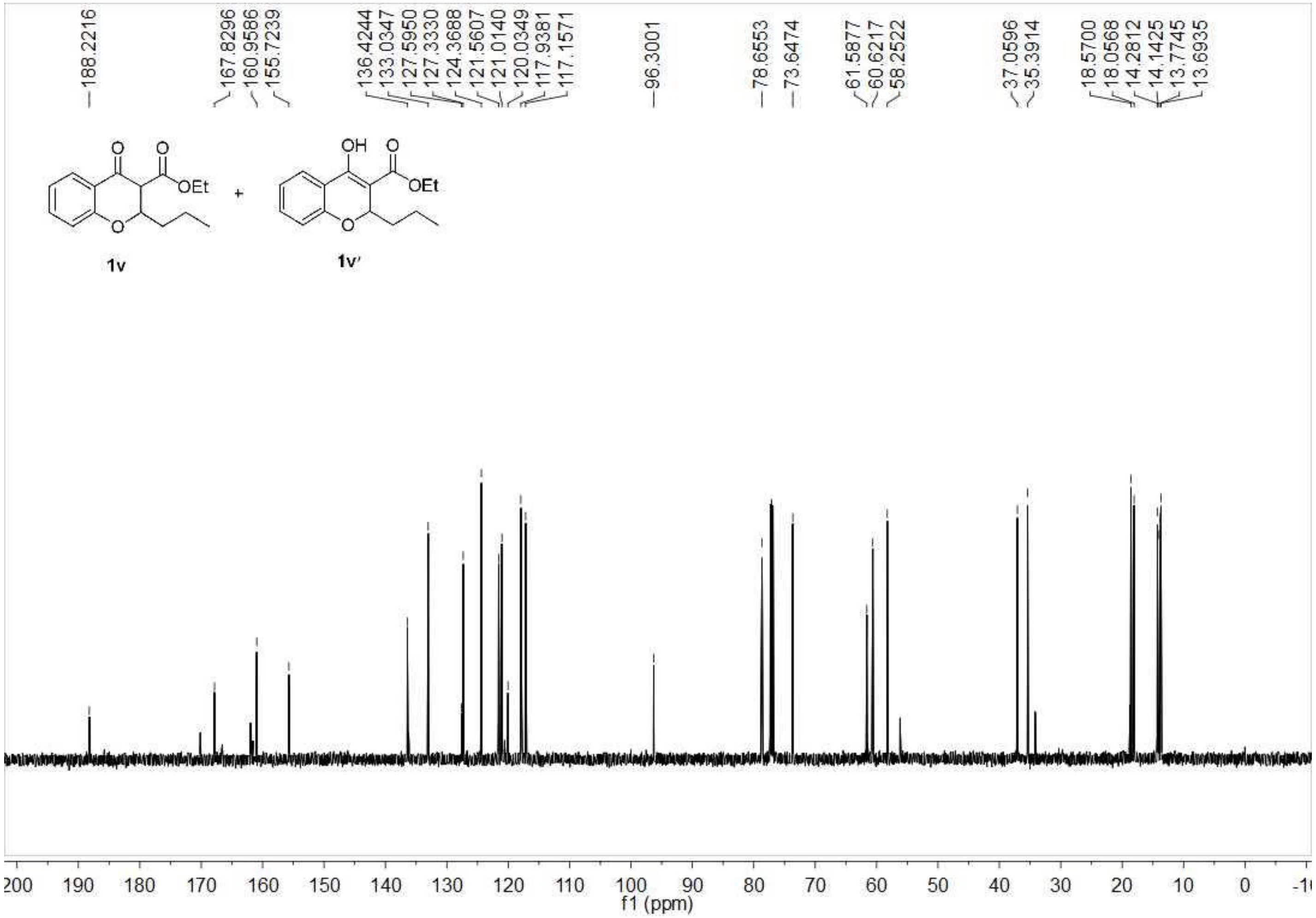
188.2216
167.8296
160.9586
155.7239
136.4244
133.0347
127.5950
127.3330
124.3688
121.5607
121.0140
120.0349
117.9381
117.1571
96.3001
78.6553
73.6474
61.5877
60.6217
58.2522
37.0596
35.3914
18.5700
18.0568
14.2812
14.1425
13.7745
13.6935
O
O
OEt
O
1v
OH
O
OEt
O
1v'
+
200
190
180
170
160
150
140
130
120
110
100
90
80
70
60
50
40
30
20
10
0
f1 (ppm)

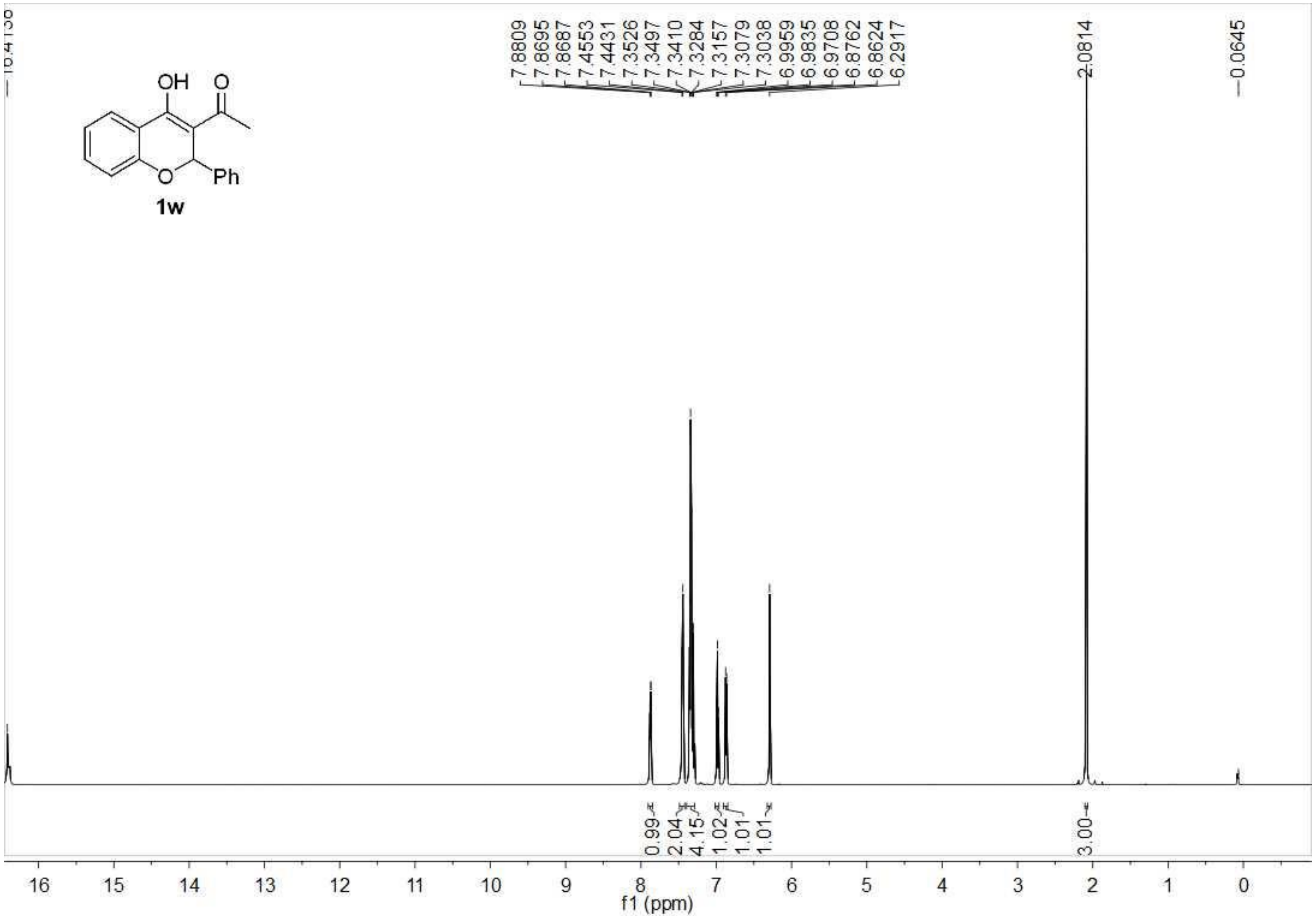

16.4158
7.8809
7.8695
7.8687
7.4553
7.4431
7.3526
7.3497
7.3410
7.3284
7.3157
7.3079
7.3038
6.9959
6.9835
6.9708
6.8762
6.8624
6.2917
2.0814
0.0645
OH
O
O
Ph
1w
0.99
2.04
4.15
1.02
1.01
1.01
3.00
16
15
14
13
12
11
10
9
8
7
6
5
4
3
2
1
0
f1 (ppm)

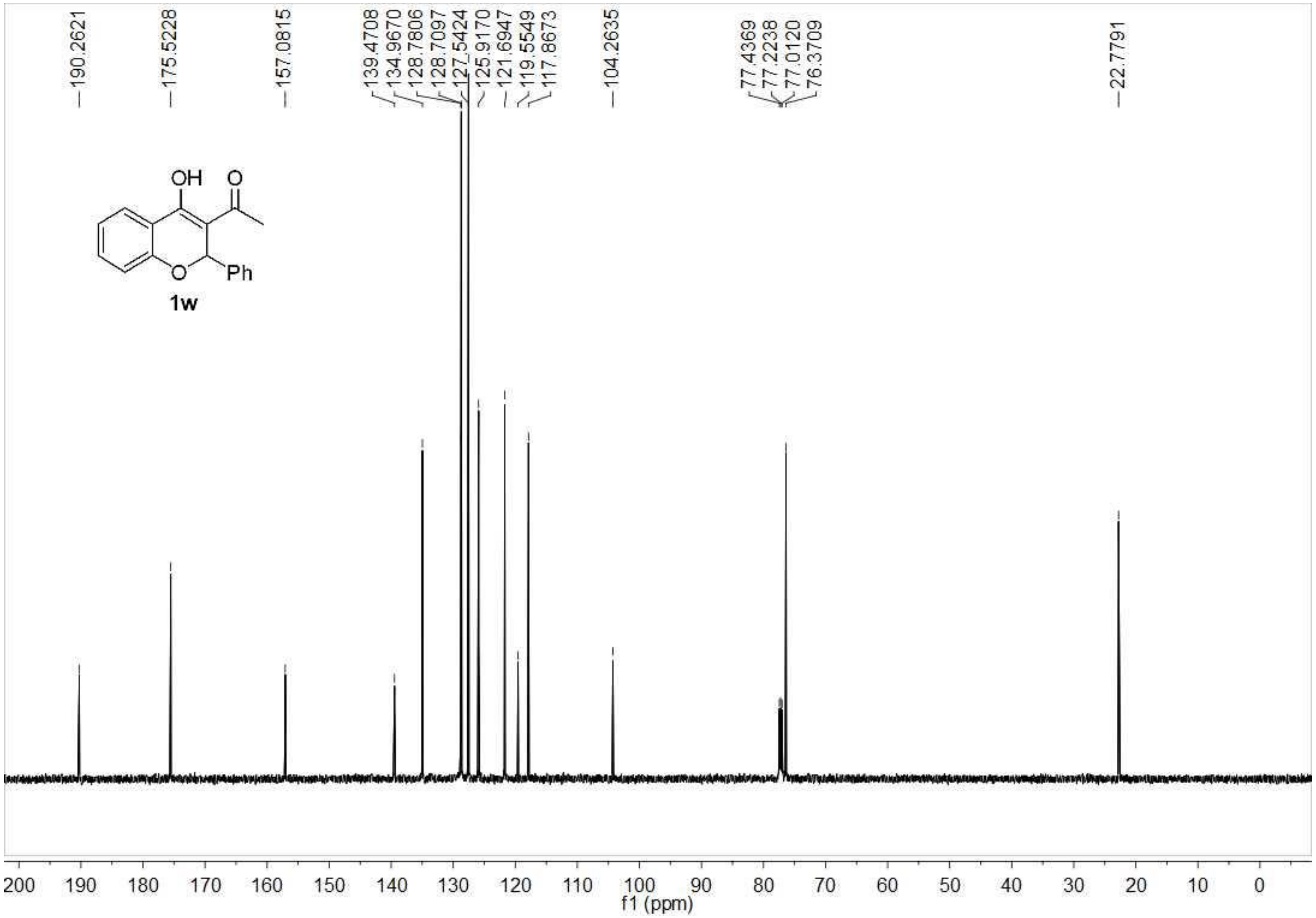
190.2621
175.5228
157.0815
139.4708
134.9670
128.7806
128.7097
127.5424
125.9170
121.6947
119.5549
117.8673
104.2635
77.4369
77.2238
77.0120
76.3709
22.7791
OH
O
Ph
1w
200 190 180 170 160 150 140 130 120 110 100 90 80 70 60 50 40 30 20 10 0
f1 (ppm)

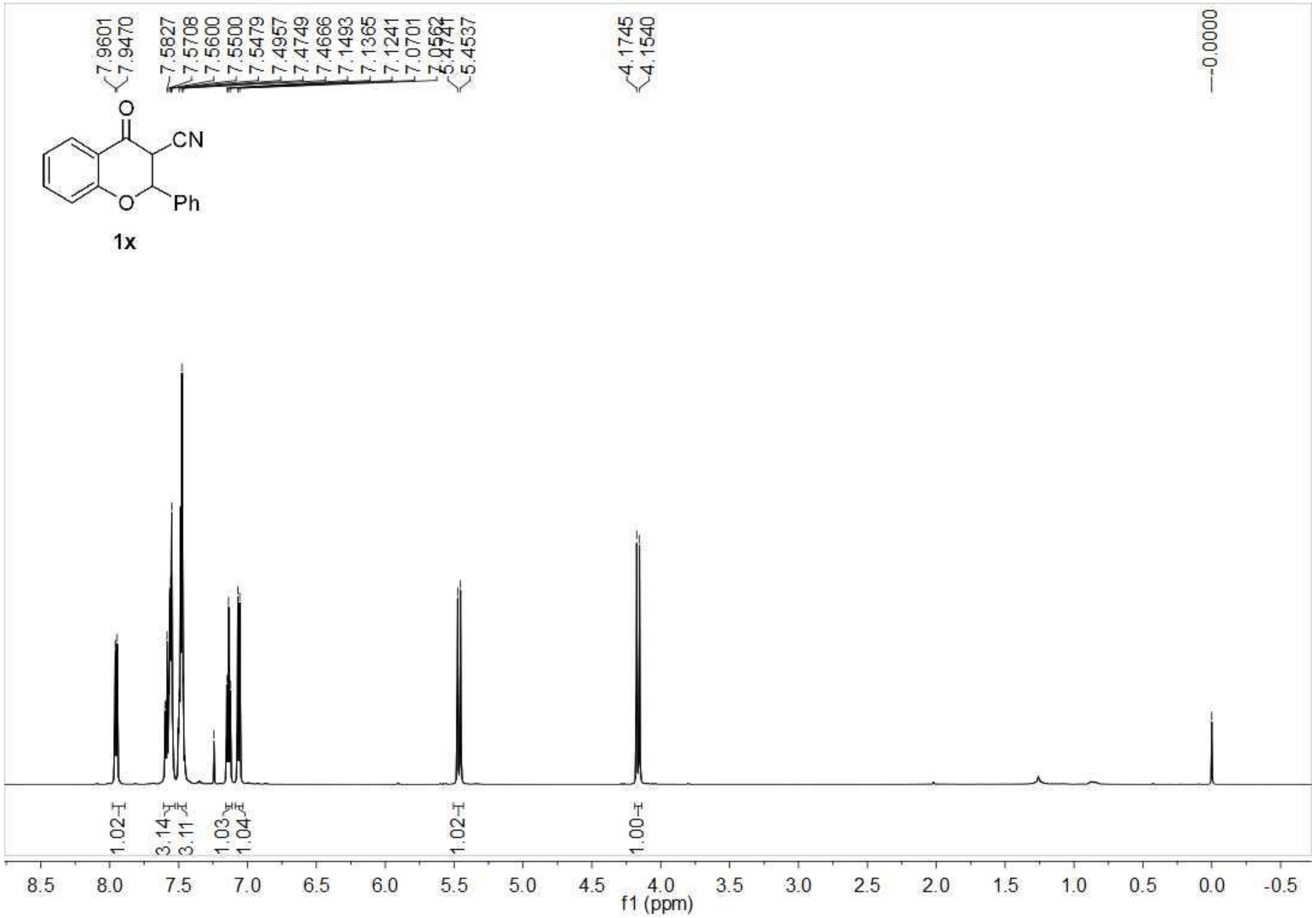
7.9601
7.9470
7.5827
7.5708
7.5600
7.5500
7.5479
7.4957
7.4749
7.4666
7.1493
7.1365
7.1241
7.0701
7.0562
5.4741
5.4537
4.1745
4.1540
0.0000
O
CN
O
Ph
1x
1.02
3.14
3.11
1.03
1.04
1.02
1.00
8.5
8.0
7.5
7.0
6.5
6.0
5.5
5.0
4.5
4.0
3.5
3.0
2.5
2.0
1.5
1.0
0.5
0.0
-0.5
f1 (ppm)

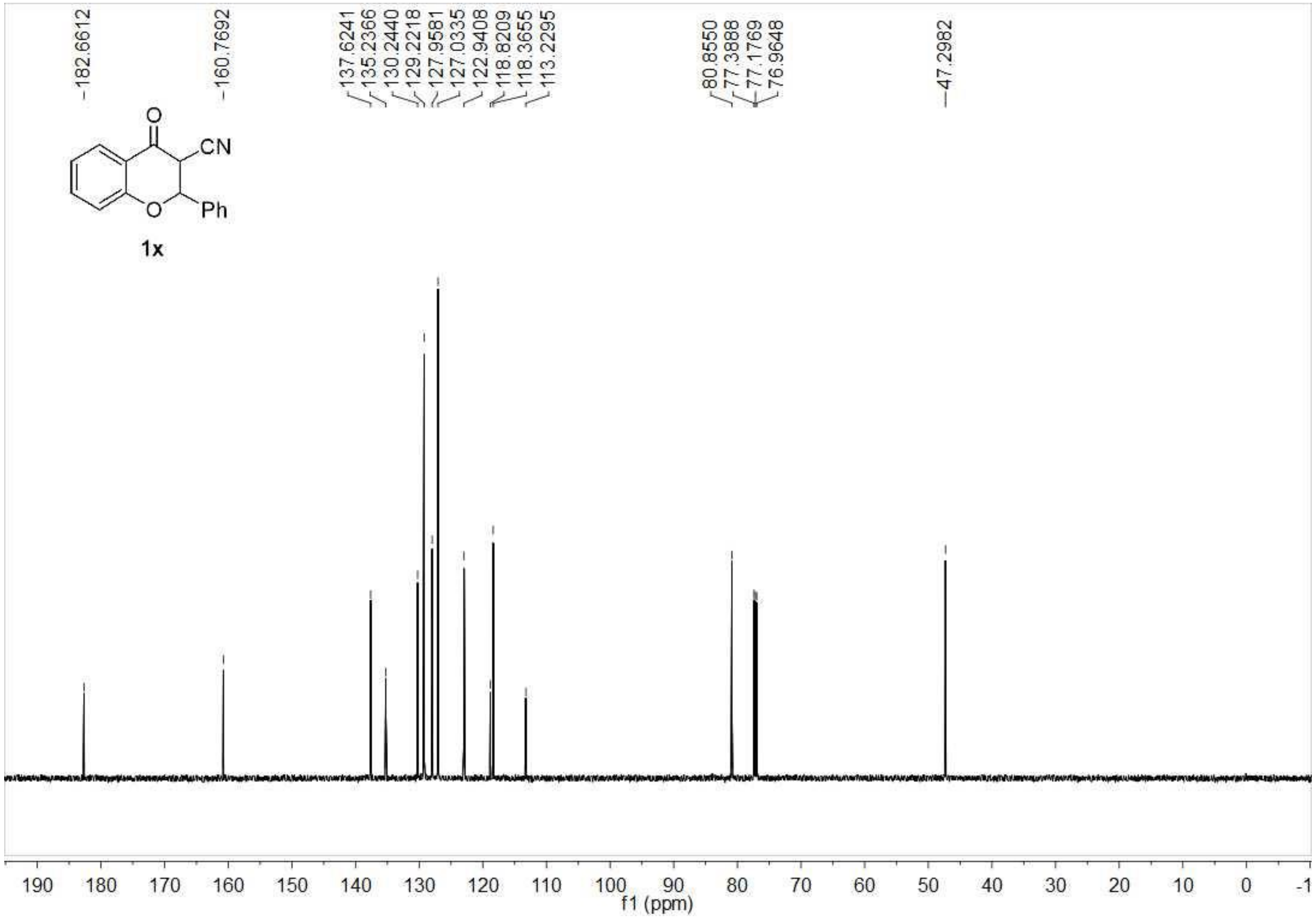

182.6612
160.7692
137.6241
135.2366
130.2440
129.2218
127.9581
127.0335
122.9408
118.8209
118.3655
113.2295
80.8550
77.3888
77.1769
76.9648
47.2982
O
CN
O
Ph
1x
190 180 170 160 150 140 130 120 110 100 90 80 70 60 50 40 30 20 10 0 -1
f1 (ppm)

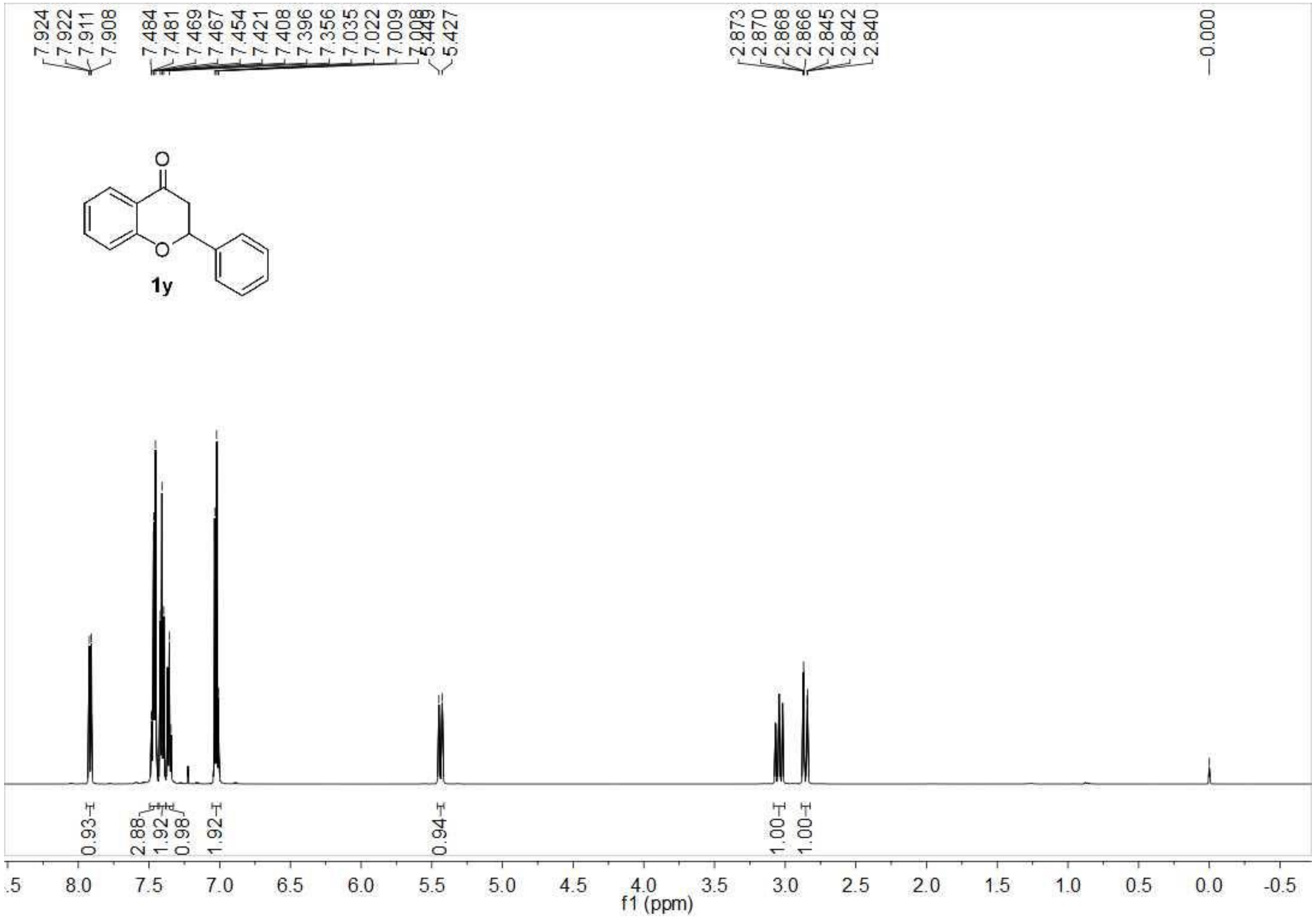
7.924
7.922
7.911
7.908
7.484
7.481
7.469
7.467
7.454
7.421
7.408
7.396
7.356
7.035
7.022
7.009
5.427
2.873
2.870
2.868
2.866
2.845
2.842
2.840
0.000
O
O
1y
0.93
2.88
1.92
0.98
1.92
0.94
1.00
1.00
8.0 7.5 7.0 6.5 6.0 5.5 5.0 4.5 4.0 3.5 3.0 2.5 2.0 1.5 1.0 0.5 0.0 -0.5
f1 (ppm)

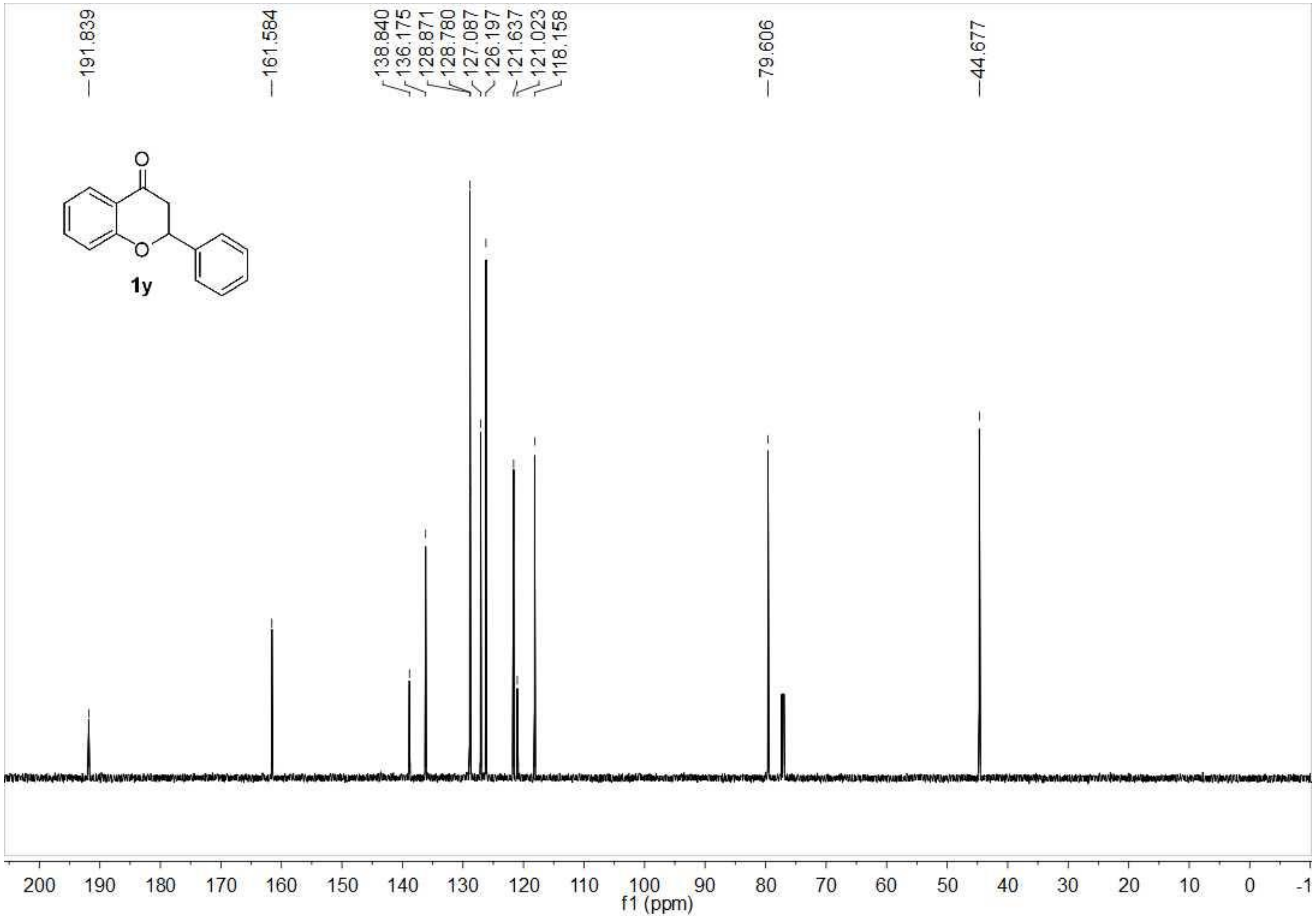
191.839
161.584
138.840
136.175
128.871
128.780
127.087
126.197
121.637
121.023
118.158
79.606
44.677
1y
200 190 180 170 160 150 140 130 120 110 100 90 80 70 60 50 40 30 20 10 0 -1
f1 (ppm)

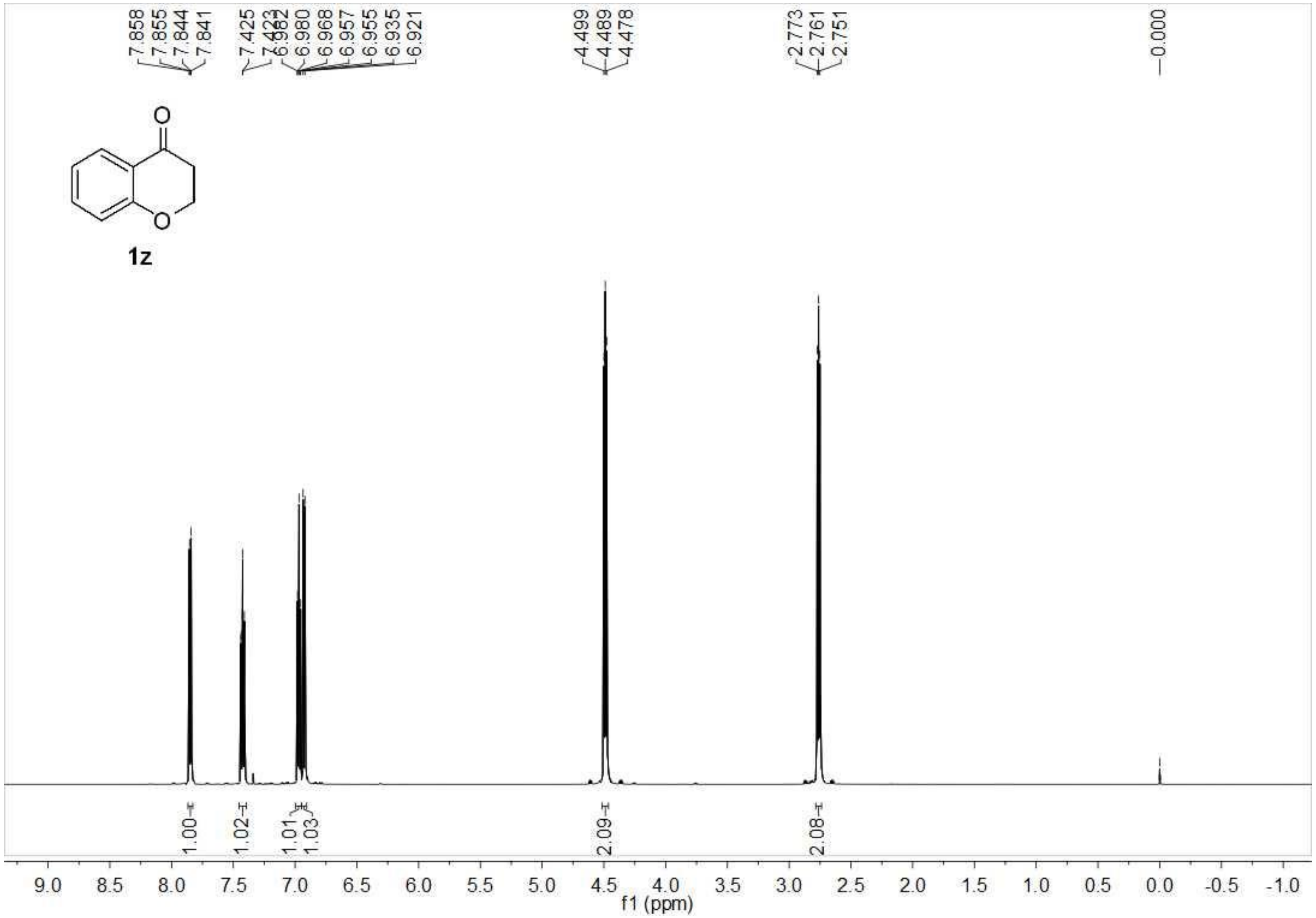
7.858
7.855
7.844
7.841
7.425
7.423
6.982
6.980
6.968
6.957
6.955
6.935
6.921
4.499
4.489
4.478
2.773
2.761
2.751
0.000
1z
1.00
1.02
1.01
1.03
2.09
2.08
9.0
8.5
8.0
7.5
7.0
6.5
6.0
5.5
5.0
4.5
4.0
3.5
3.0
2.5
2.0
1.5
1.0
0.5
0.0
-0.5
-1.0
f1 (ppm)

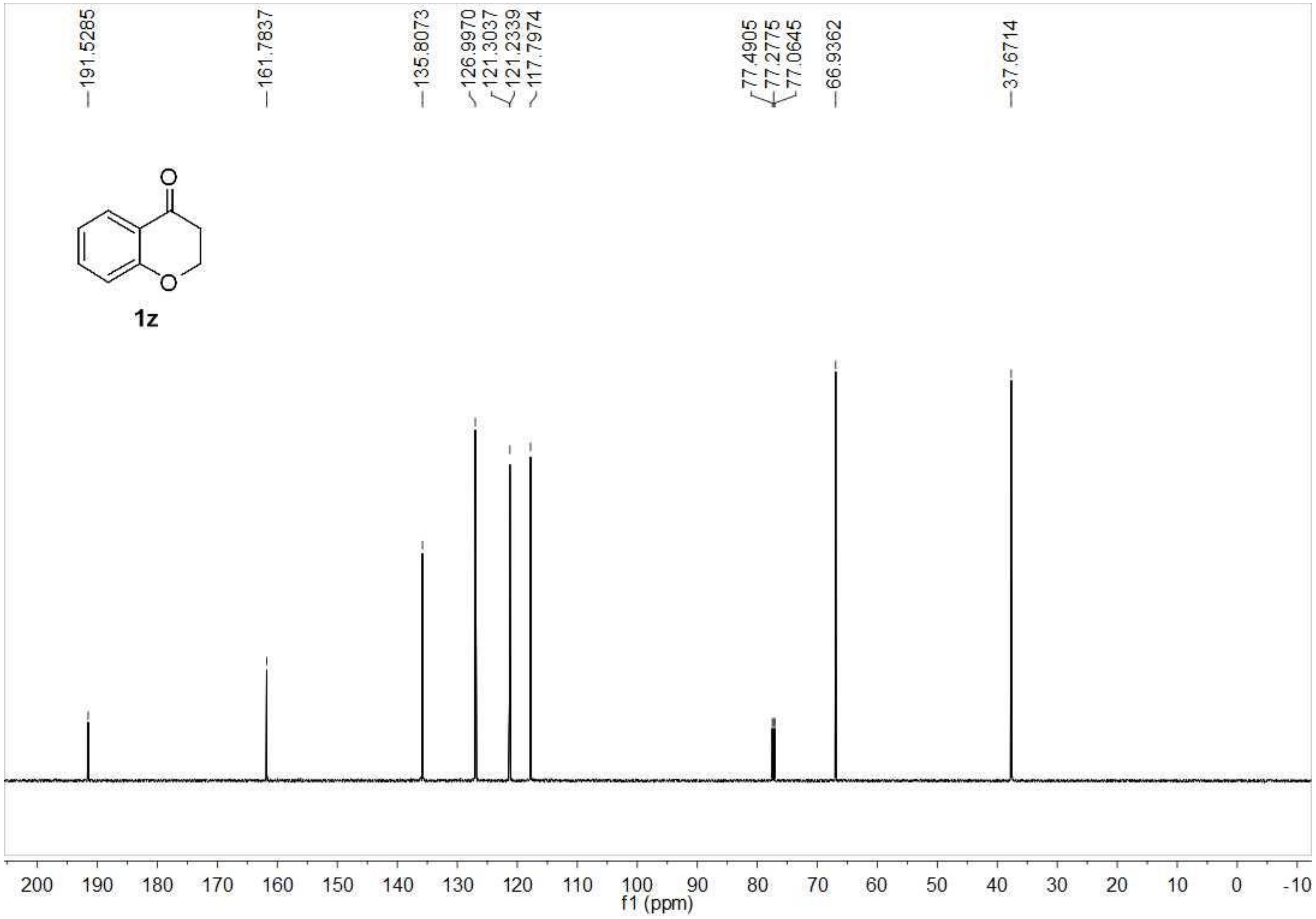
191.5285
161.7837
135.8073
126.9970
121.3037
121.2339
117.7974
77.4905
77.2775
77.0645
66.9362
37.6714
1z
200 190 180 170 160 150 140 130 120 110 100 90 80 70 60 50 40 30 20 10 0 -10
f1 (ppm)

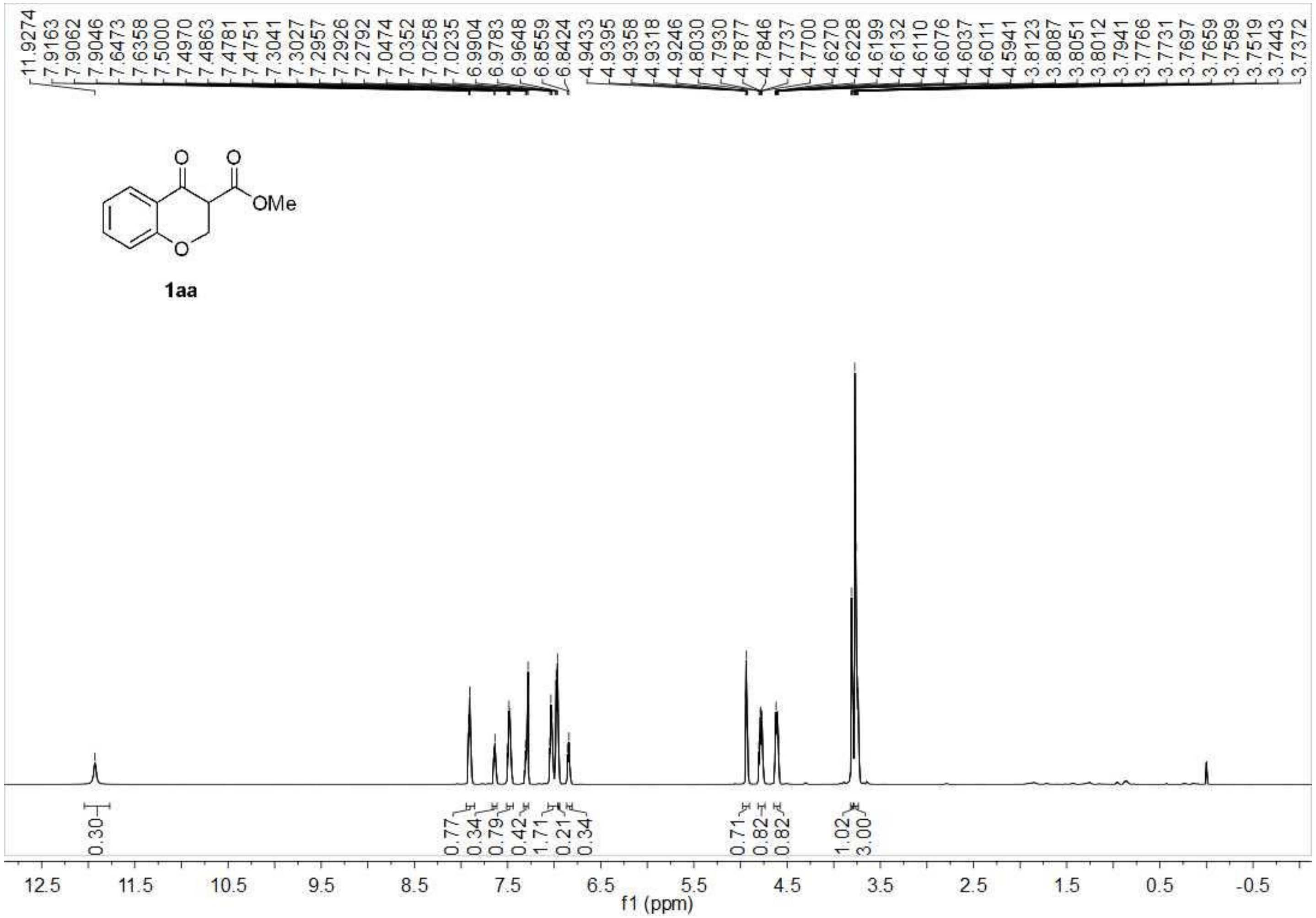

1aa

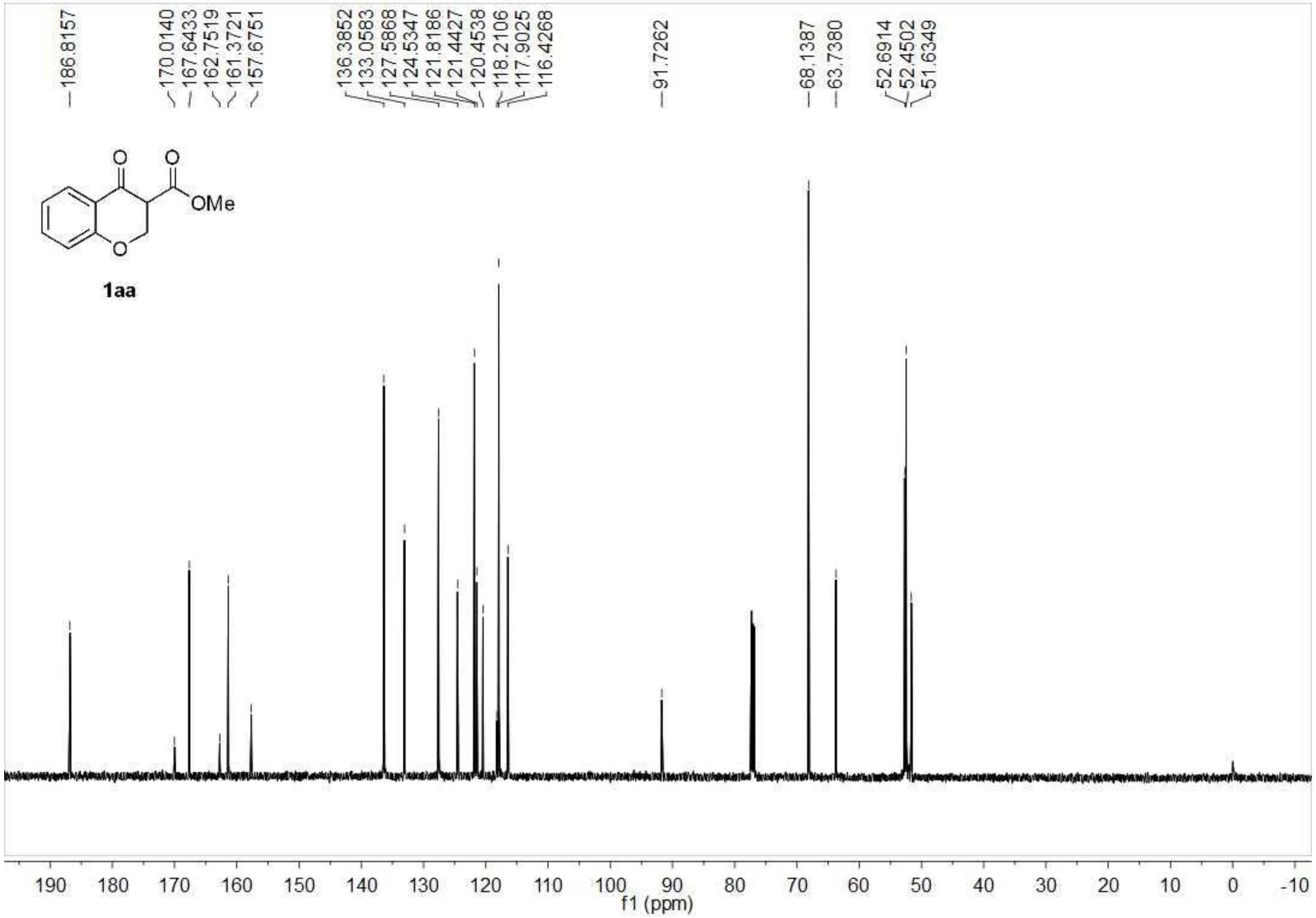
186.8157
170.0140
167.6433
162.7519
161.3721
157.6751
136.3852
133.0583
127.5868
124.5347
121.8186
121.4427
120.4538
118.2106
117.9025
116.4268
91.7262
68.1387
63.7380
52.6914
52.4502
51.6349
OMe
O
1aa
190 180 170 160 150 140 130 120 110 100 90 80 70 60 50 40 30 20 10 0 -10
f1 (ppm)

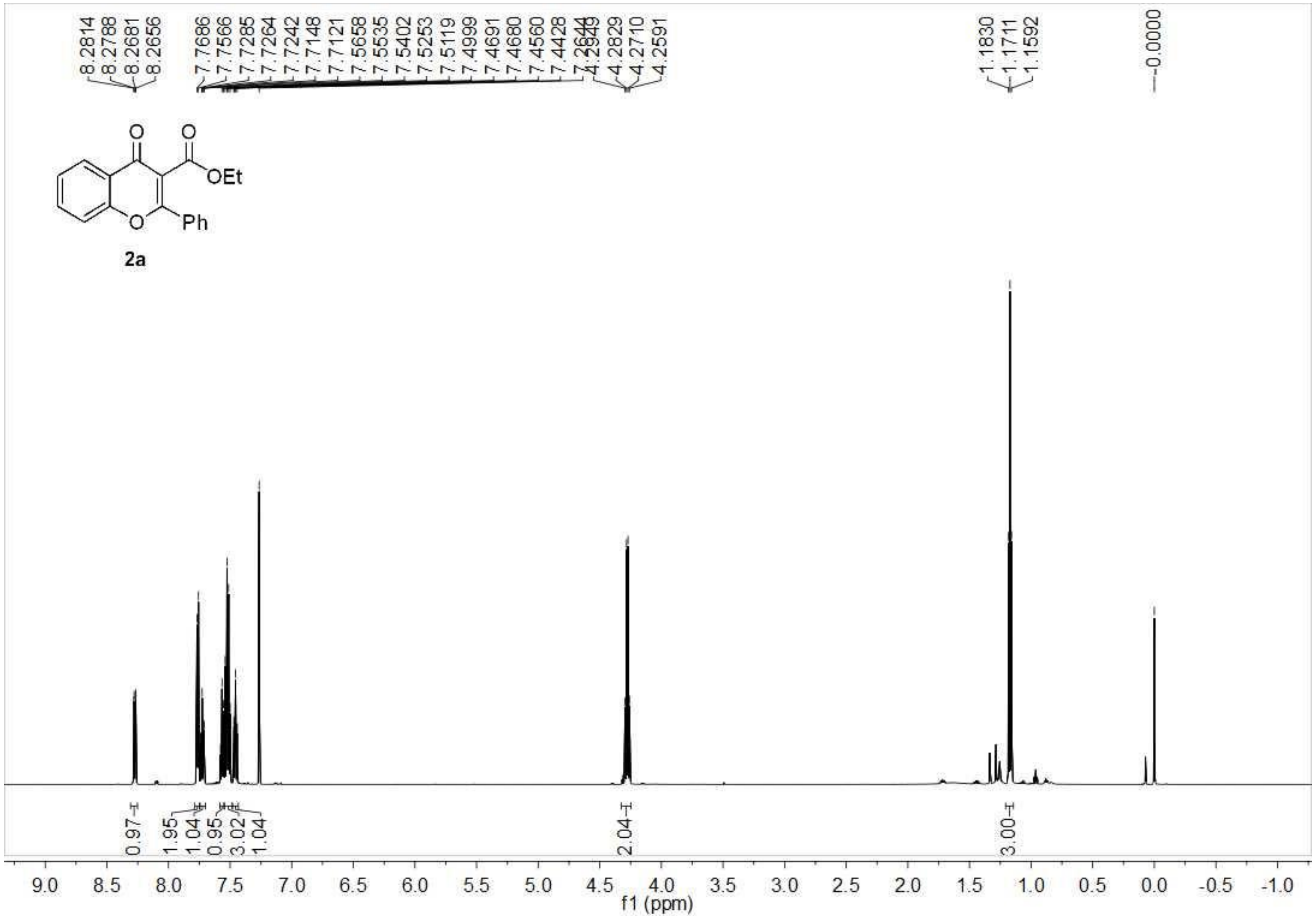

8.2814
8.2788
8.2681
8.2656
7.7686
7.7566
7.7285
7.7264
7.7242
7.7148
7.7121
7.5658
7.5535
7.5402
7.5253
7.5119
7.4999
7.4691
7.4680
7.4560
7.4428
7.2644
4.2949
4.2829
4.2710
4.2591
1.1830
1.1711
1.1592
0.0000
OEt
Ph
2a
0.97
1.95
1.04
0.95
3.02
1.04
2.04
3.00
9.0 8.5 8.0 7.5 7.0 6.5 6.0 5.5 5.0 4.5 4.0 3.5 3.0 2.5 2.0 1.5 1.0 0.5 0.0 -0.5 -1.0
f1 (ppm)

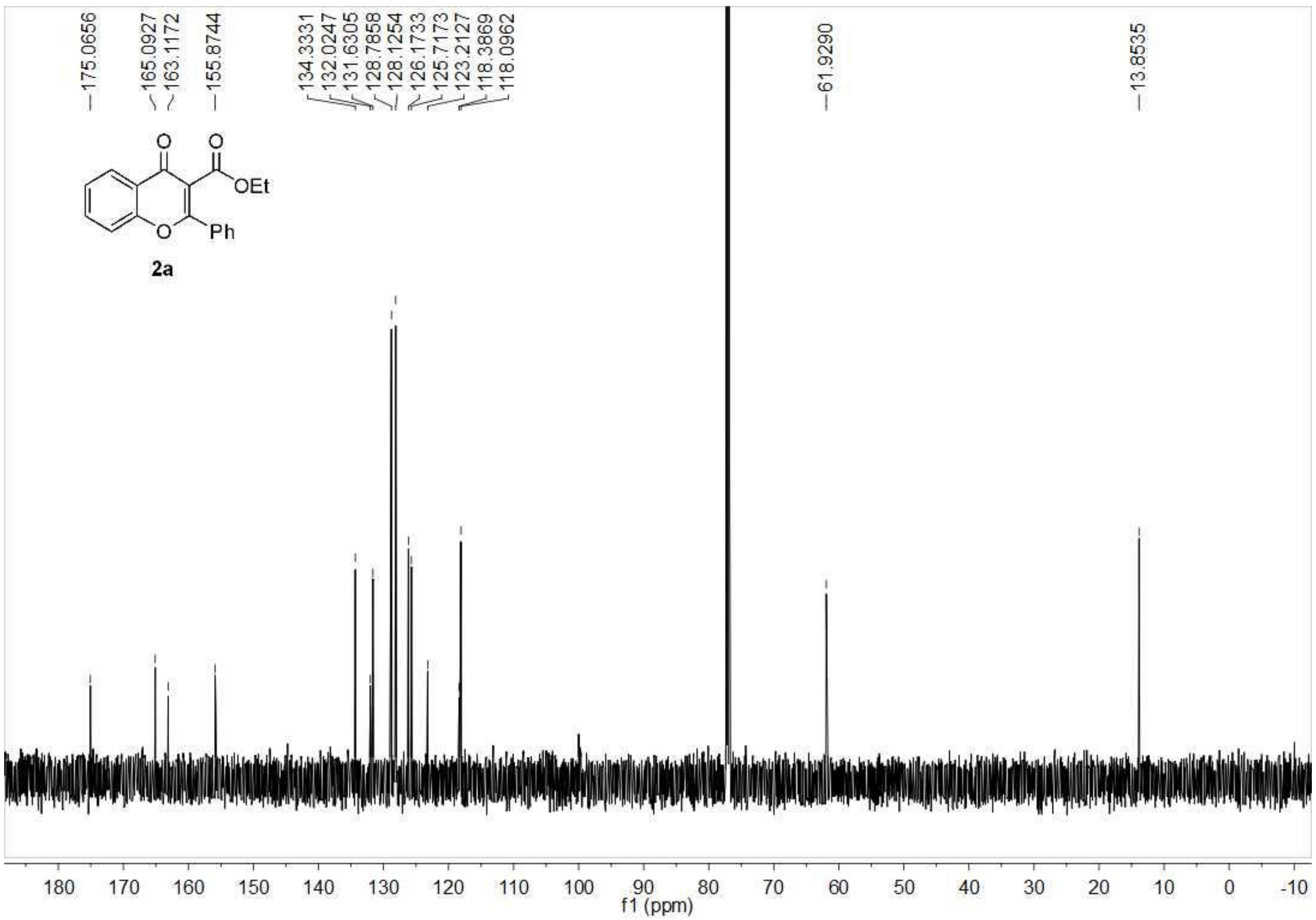
175.0656
165.0927
163.1172
155.8744
134.3331
132.0247
131.6305
128.7858
128.1254
126.1733
125.7173
123.2127
118.3869
118.0962
61.9290
13.8535
OEt
Ph
2a
180 170 160 150 140 130 120 110 100 90 80 70 60 50 40 30 20 10 0 -10
f1 (ppm)

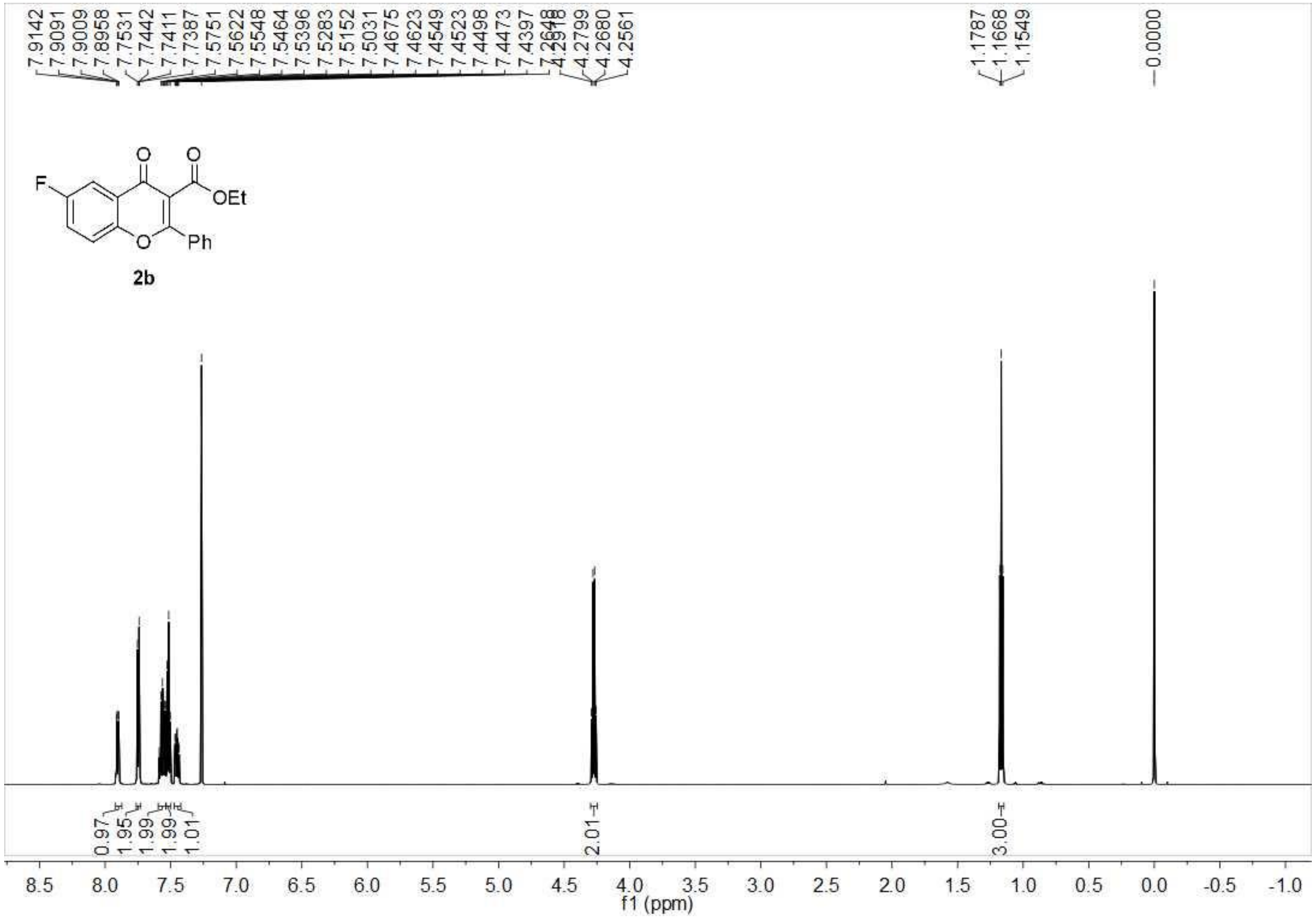
7.9142
7.9091
7.9009
7.8958
7.7531
7.7442
7.7411
7.7387
7.5751
7.5622
7.5548
7.5464
7.5396
7.5283
7.5152
7.5031
7.4675
7.4623
7.4549
7.4523
7.4498
7.4473
7.4397
7.2648
4.2918
4.2799
4.2680
4.2561
1.1787
1.1668
1.1549
0.0000
F
O
O
OEt
O
Ph
2b
0.97
1.95
1.99
1.99
1.01
2.01
3.00
8.5 8.0 7.5 7.0 6.5 6.0 5.5 5.0 4.5 4.0 3.5 3.0 2.5 2.0 1.5 1.0 0.5 0.0 -0.5 -1.0
f1 (ppm)

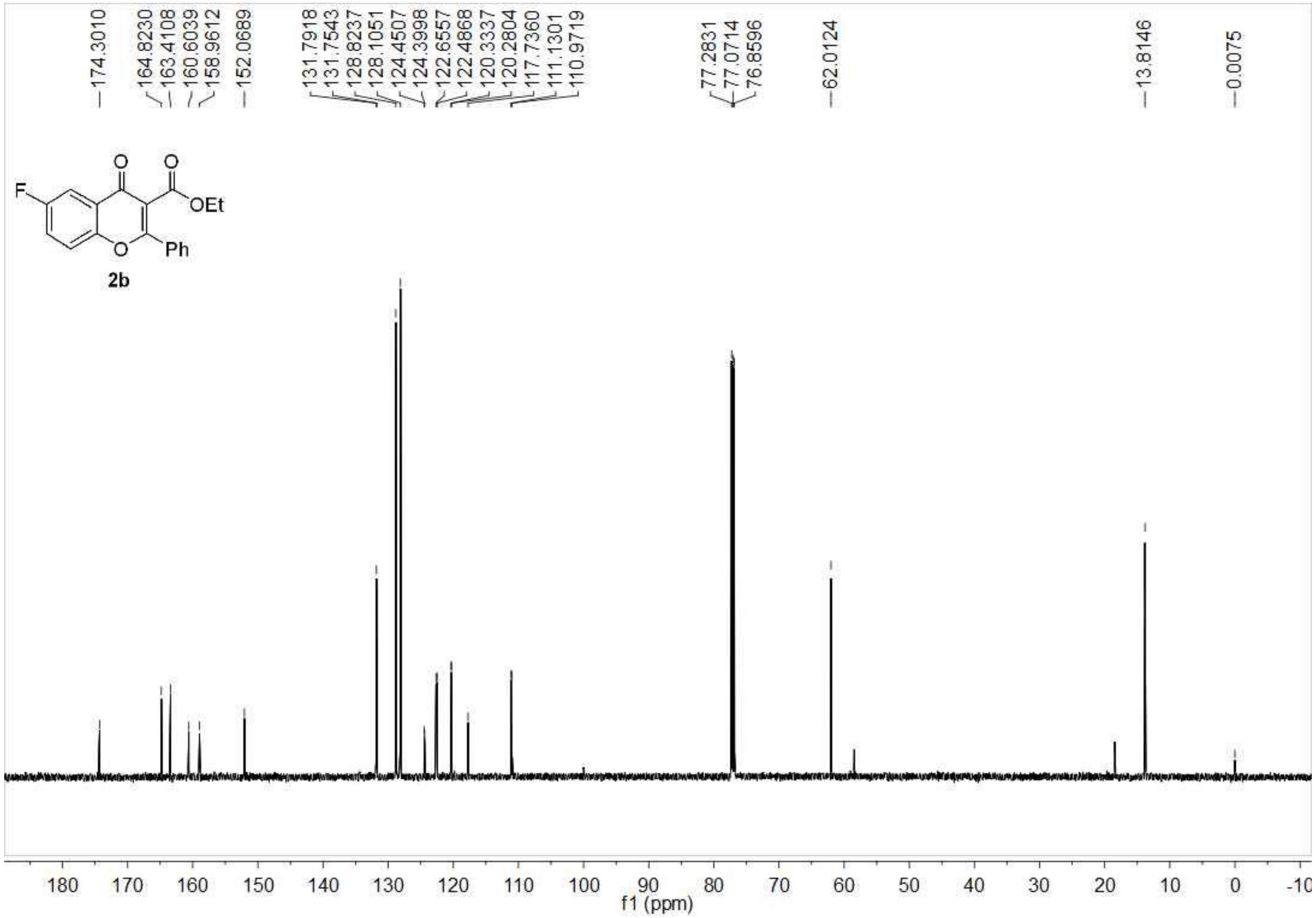

174.3010
164.8230
163.4108
160.6039
158.9612
152.0689
131.7918
131.7543
128.8237
128.1051
124.4507
124.3998
122.6557
122.4868
120.3337
120.2804
117.7360
111.1301
110.9719
77.2831
77.0714
76.8596
62.0124
13.8146
-0.0075
F
O
O
OEt
O
Ph
2b
180 170 160 150 140 130 120 110 100 90 80 70 60 50 40 30 20 10 0 -10
f1 (ppm)

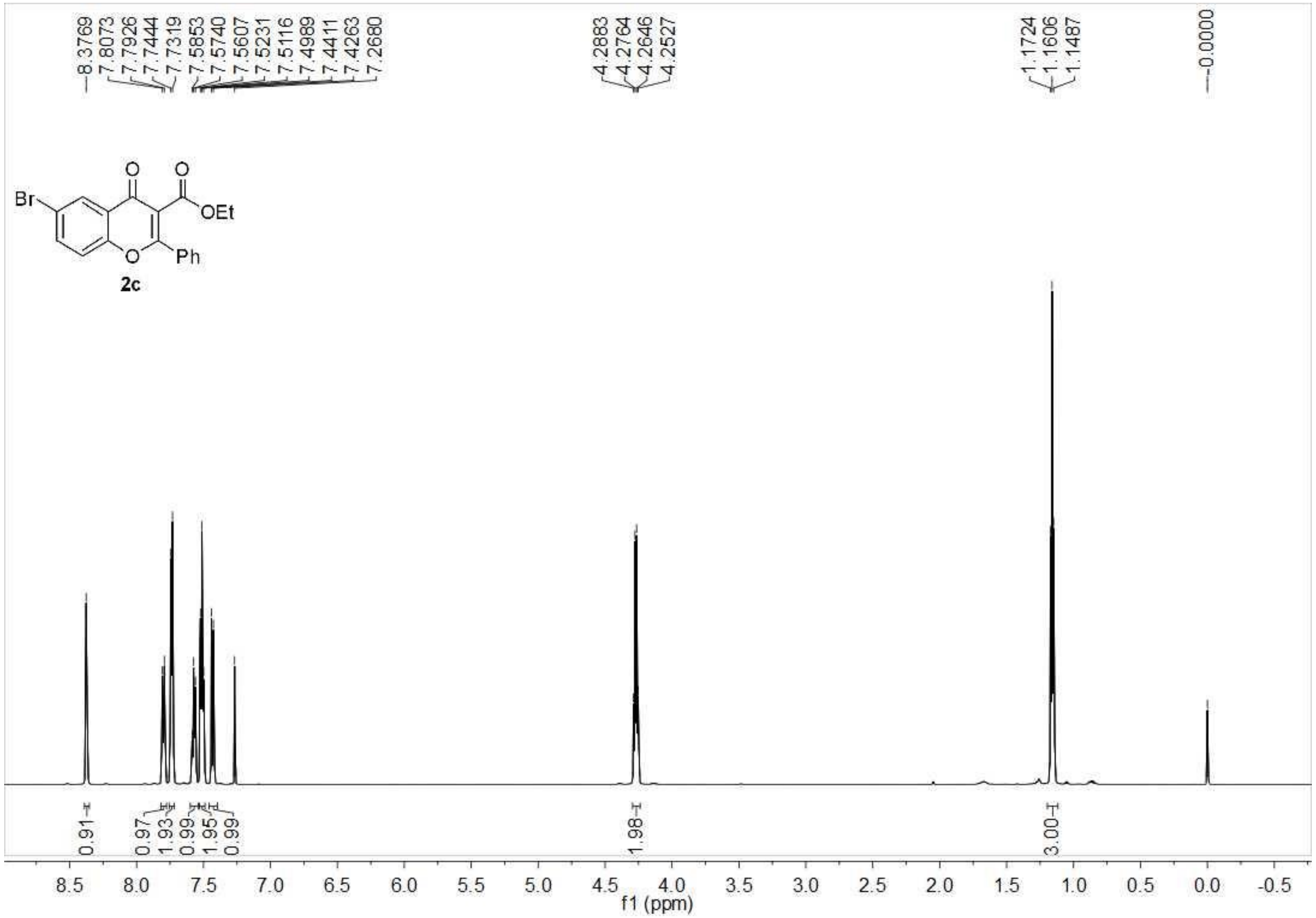
8.3769
7.8073
7.7926
7.7444
7.7319
7.5853
7.5740
7.5607
7.5231
7.5116
7.4989
7.4411
7.4263
7.2680
4.2883
4.2764
4.2646
4.2527
1.1724
1.1606
1.1487
0.0000
Br
O
O
OEt
O
Ph
2c
0.91
0.97
1.93
0.99
1.95
0.99
1.98
3.00
8.5
8.0
7.5
7.0
6.5
6.0
5.5
5.0
4.5
4.0
3.5
3.0
2.5
2.0
1.5
1.0
0.5
0.0
-0.5
f1 (ppm)

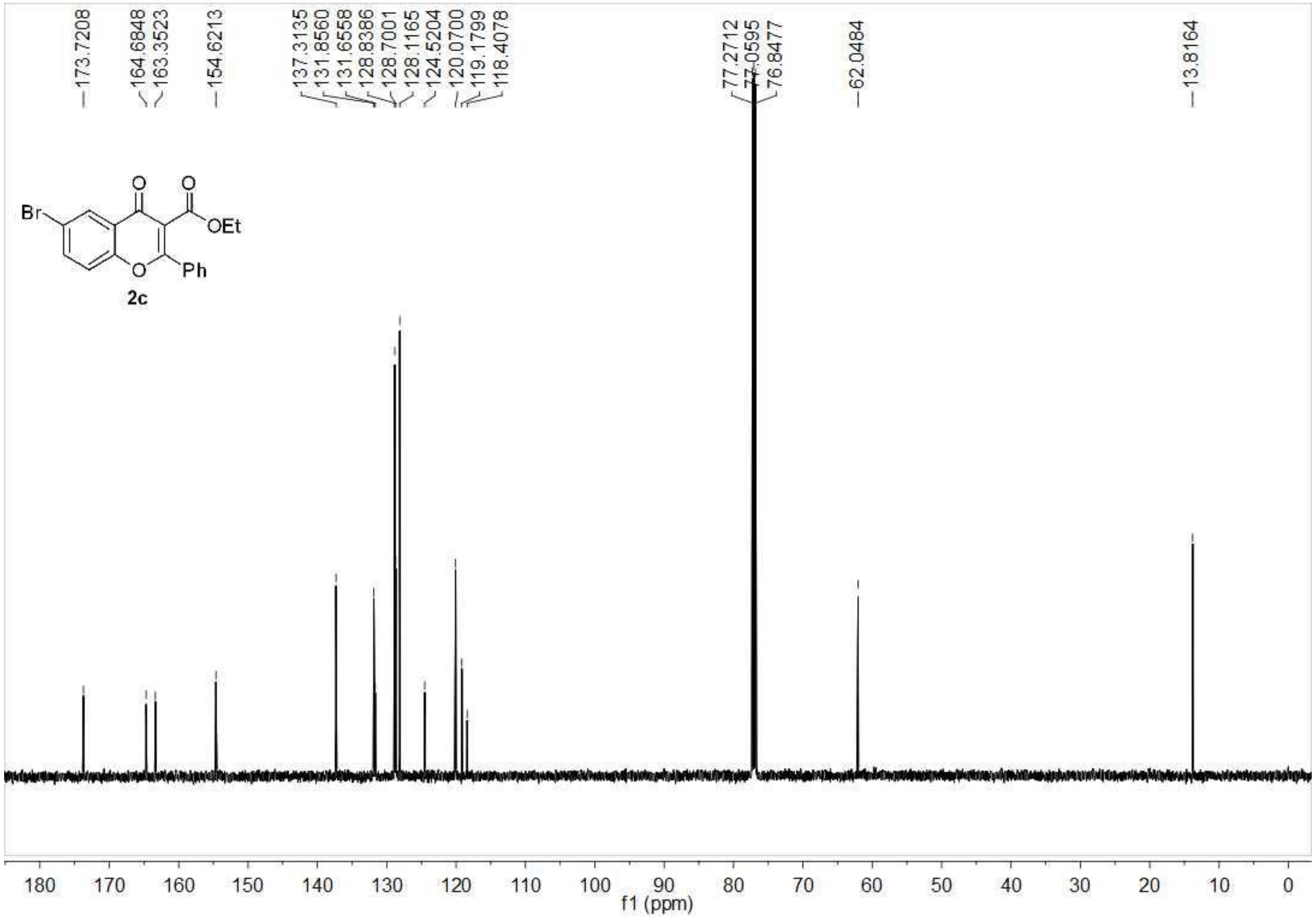

173.7208
164.6848
163.3523
154.6213
137.3135
131.8560
131.6558
128.8386
128.7001
128.1165
124.5204
120.0700
119.1799
118.4078
77.2712
77.0595
76.8477
62.0484
13.8164
Br
O
O
OEt
O
Ph
2c
180 170 160 150 140 130 120 110 100 90 80 70 60 50 40 30 20 10 0
f1 (ppm)

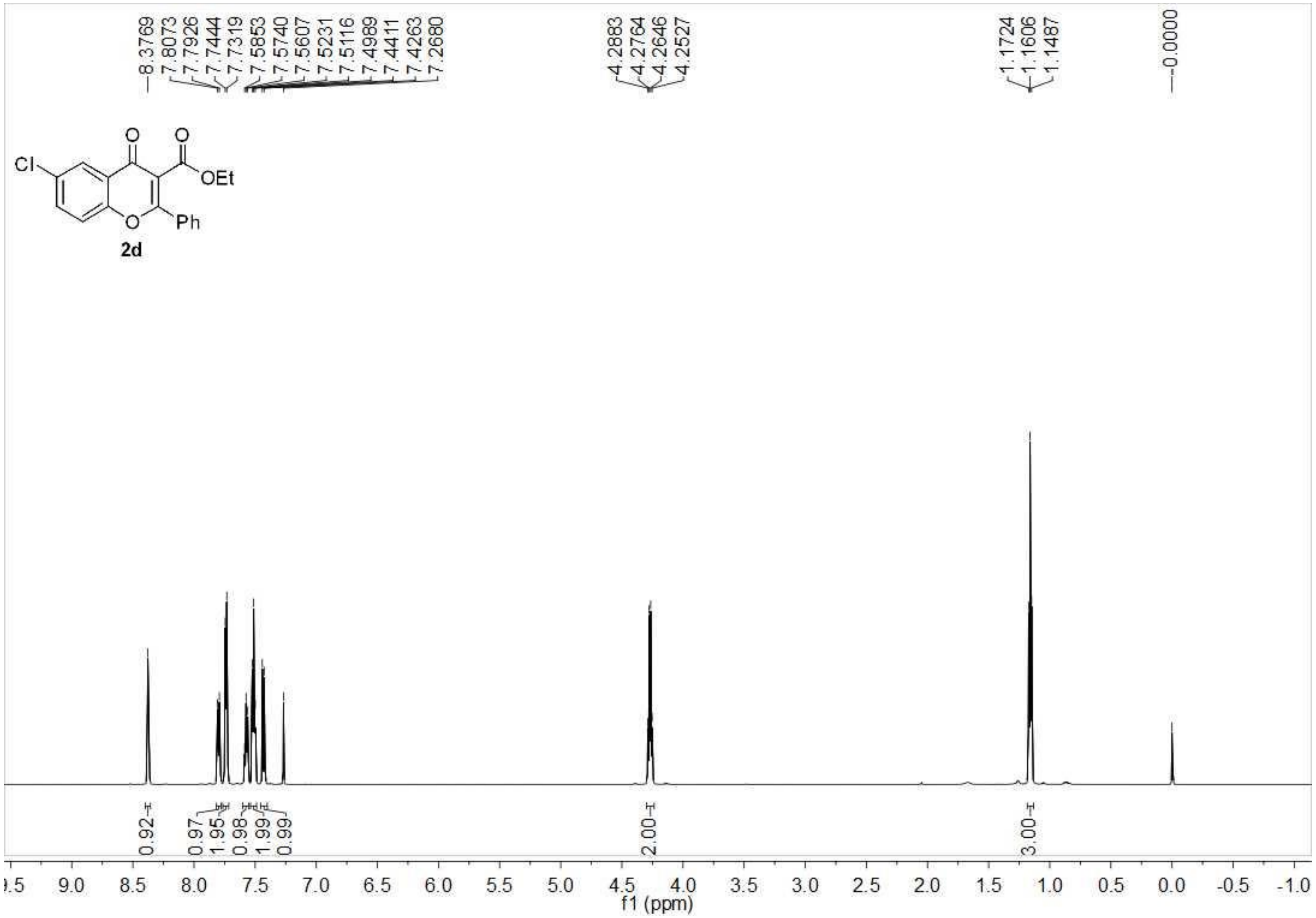
8.3769
7.8073
7.7926
7.7444
7.7319
7.5853
7.5740
7.5607
7.5231
7.5116
7.4989
7.4411
7.4263
7.2680
4.2883
4.2764
4.2646
4.2527
1.1724
1.1606
1.1487
0.0000
Cl
O
O
OEt
O
Ph
2d
0.92
0.97
1.95
0.98
1.99
0.99
2.00
3.00
9.0
8.5
8.0
7.5
7.0
6.5
6.0
5.5
5.0
4.5
4.0
3.5
3.0
2.5
2.0
1.5
1.0
0.5
0.0
-0.5
-1.0
f1 (ppm)

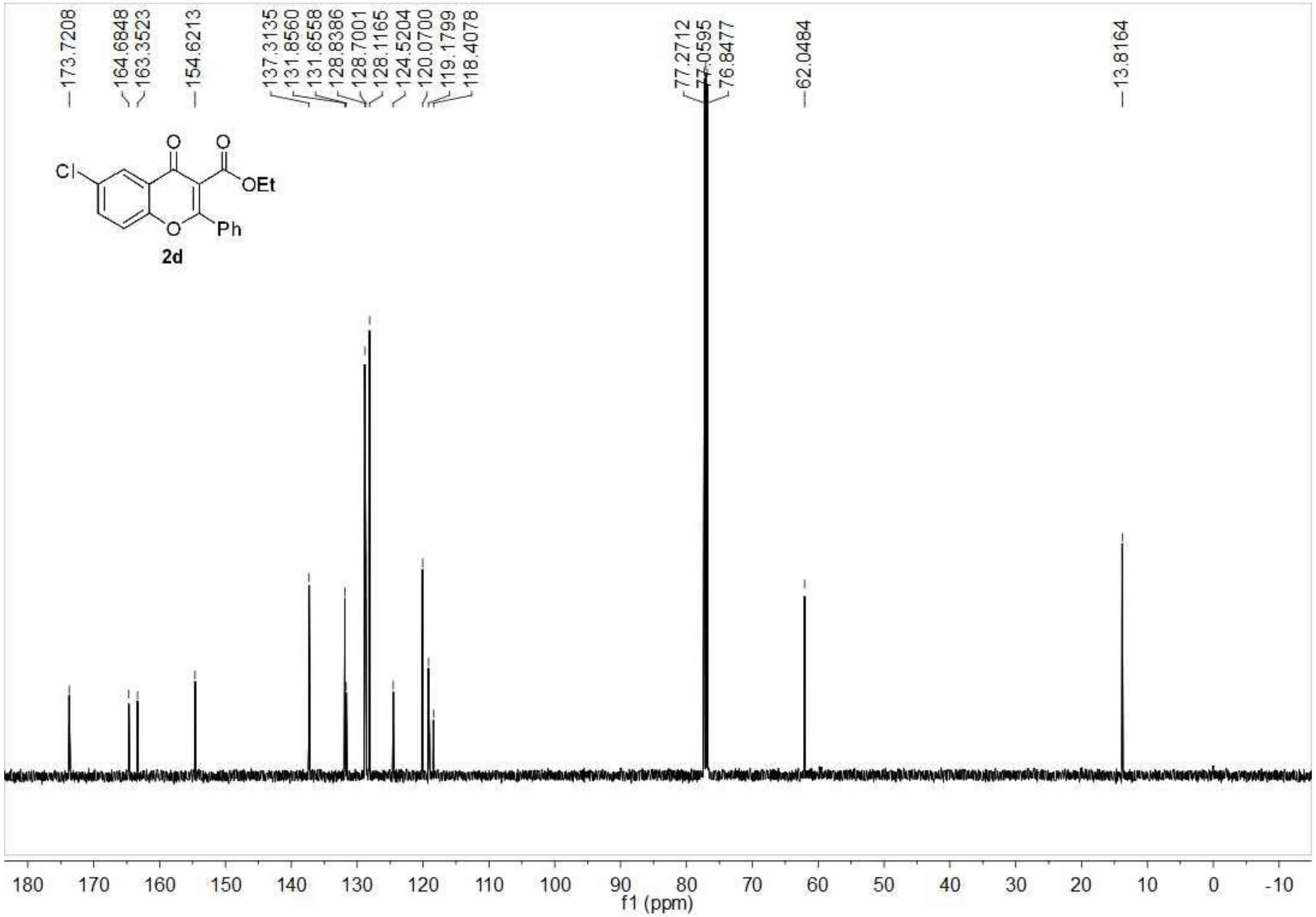
173.7208
164.6848
163.3523
154.6213
137.3135
131.8560
131.6558
128.8386
128.7001
128.1165
124.5204
120.0700
119.1799
118.4078
77.2712
77.0595
76.8477
62.0484
13.8164
Cl
O
O
OEt
O
Ph
2d
180 170 160 150 140 130 120 110 100 90 80 70 60 50 40 30 20 10 0 -10
f1 (ppm)

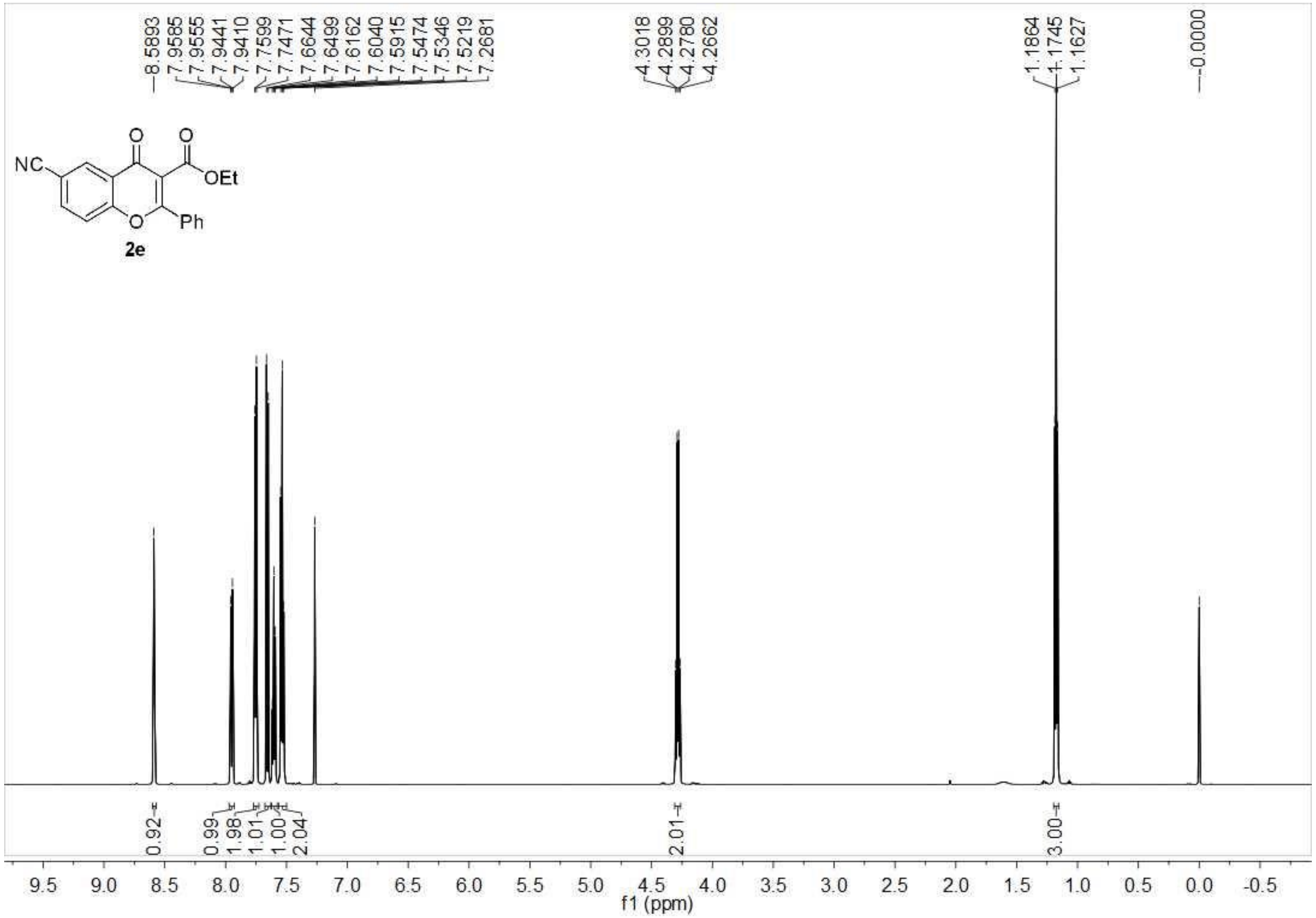
8.5893
7.9585
7.9555
7.9441
7.9410
7.7599
7.7471
7.6644
7.6499
7.6162
7.6040
7.5915
7.5474
7.5346
7.5219
7.2681
4.3018
4.2899
4.2780
4.2662
1.1864
1.1745
1.1627
-0.0000
NC
O
O
OEt
O
Ph
2e
0.92
0.99
1.98
1.01
1.00
2.04
2.01
3.00
9.5 9.0 8.5 8.0 7.5 7.0 6.5 6.0 5.5 5.0 4.5 4.0 3.5 3.0 2.5 2.0 1.5 1.0 0.5 0.0 -0.5
f1 (ppm)

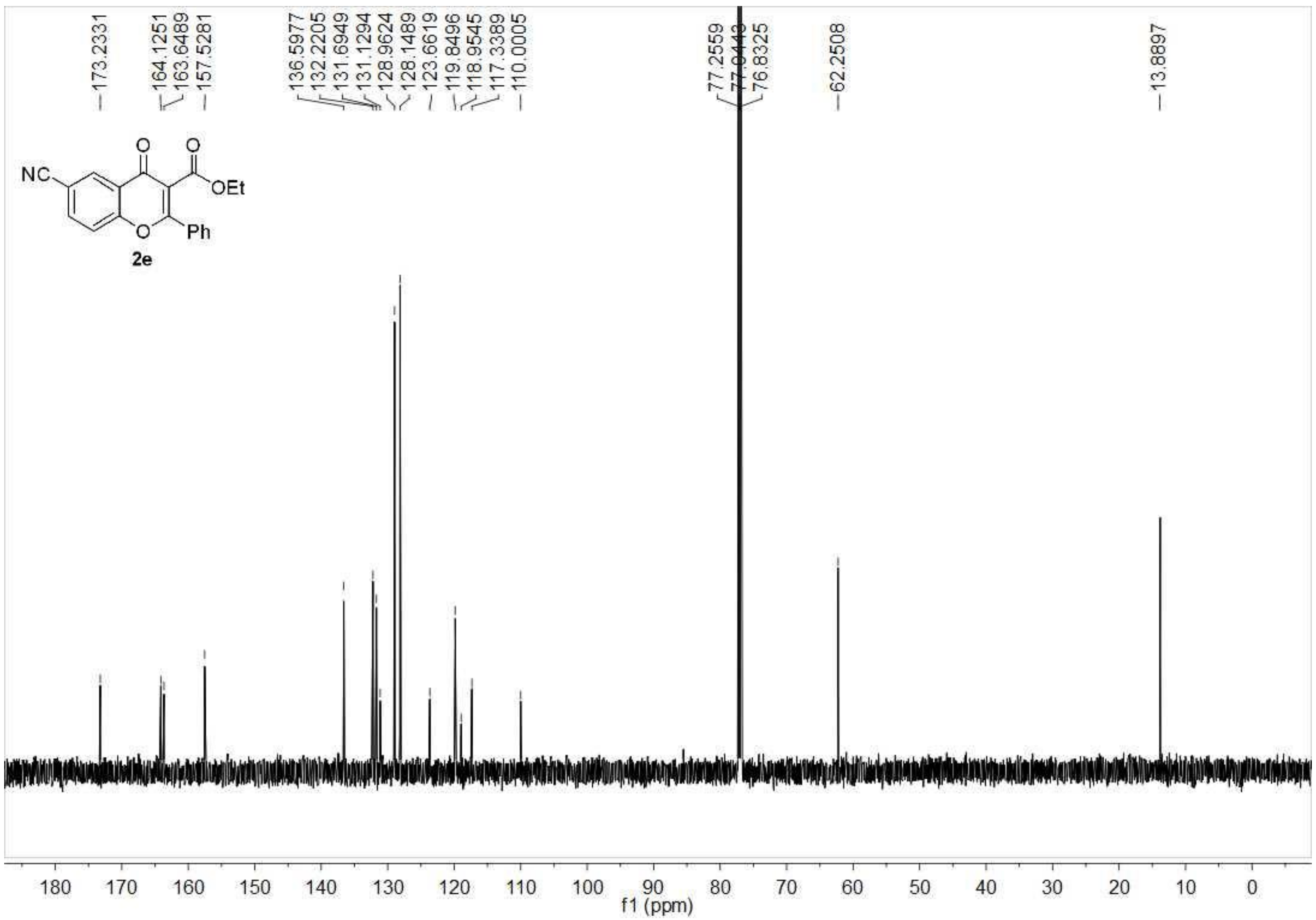

173.2331
164.1251
163.6489
157.5281
136.5977
132.2205
131.6949
131.1294
128.9624
128.1489
123.6619
119.8496
118.9545
117.3389
110.0005
77.2559
76.8325
62.2508
13.8897
NC
O
O
OEt
O
Ph
2e
180
170
160
150
140
130
120
110
100
90
80
70
60
50
40
30
20
10
0
f1 (ppm)

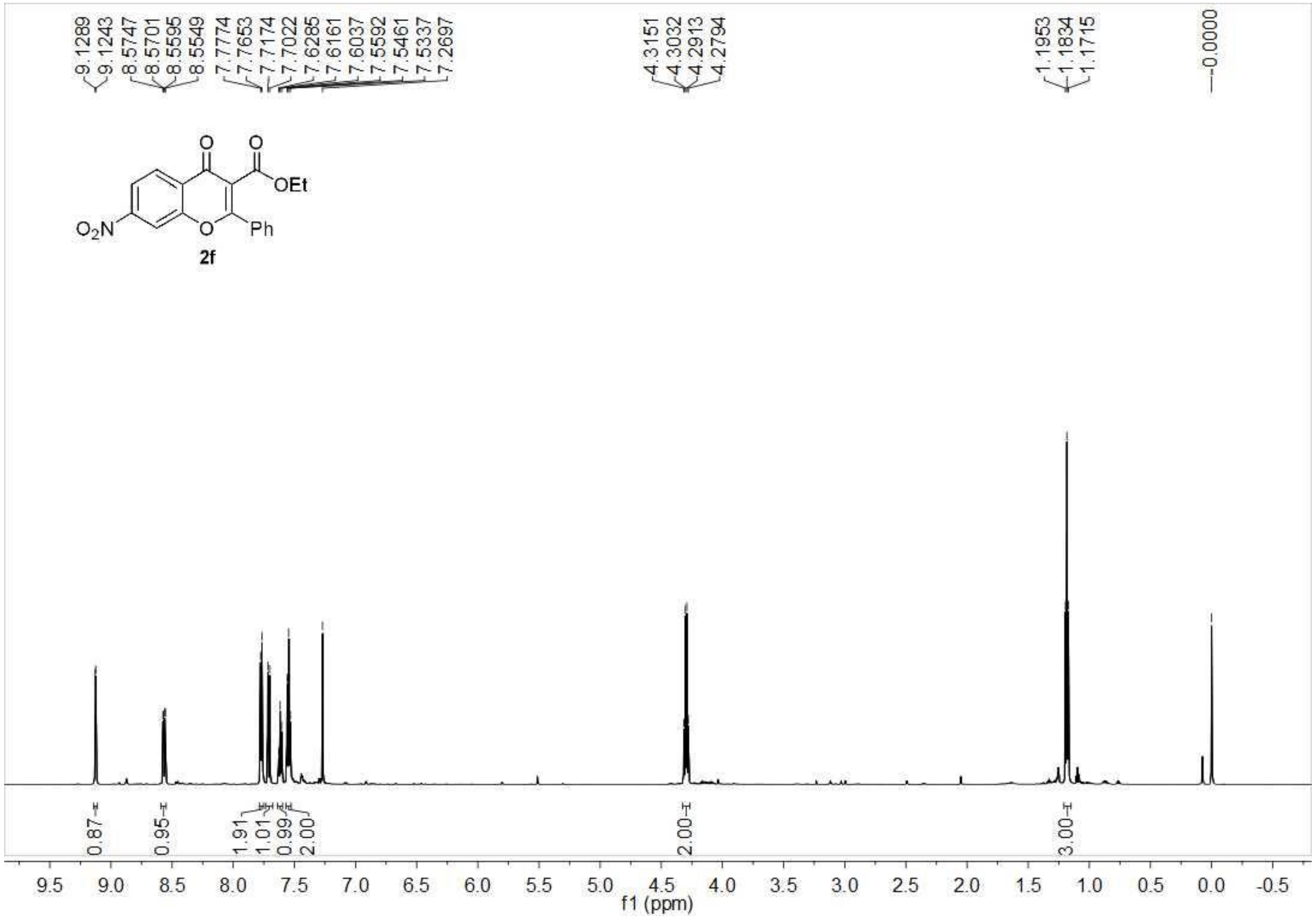

9.1289
9.1243
8.5747
8.5701
8.5595
8.5549
7.7774
7.7653
7.7174
7.7022
7.6285
7.6161
7.6037
7.5592
7.5461
7.5337
7.2697
4.3151
4.3032
4.2913
4.2794
1.1953
1.1834
1.1715
0.0000
OEt
O_2N
Ph
2f
0.87
0.95
1.91
1.01
0.99
2.00
2.00
3.00
9.5 9.0 8.5 8.0 7.5 7.0 6.5 6.0 5.5 5.0 4.5 4.0 3.5 3.0 2.5 2.0 1.5 1.0 0.5 0.0 -0.5
f1 (ppm)

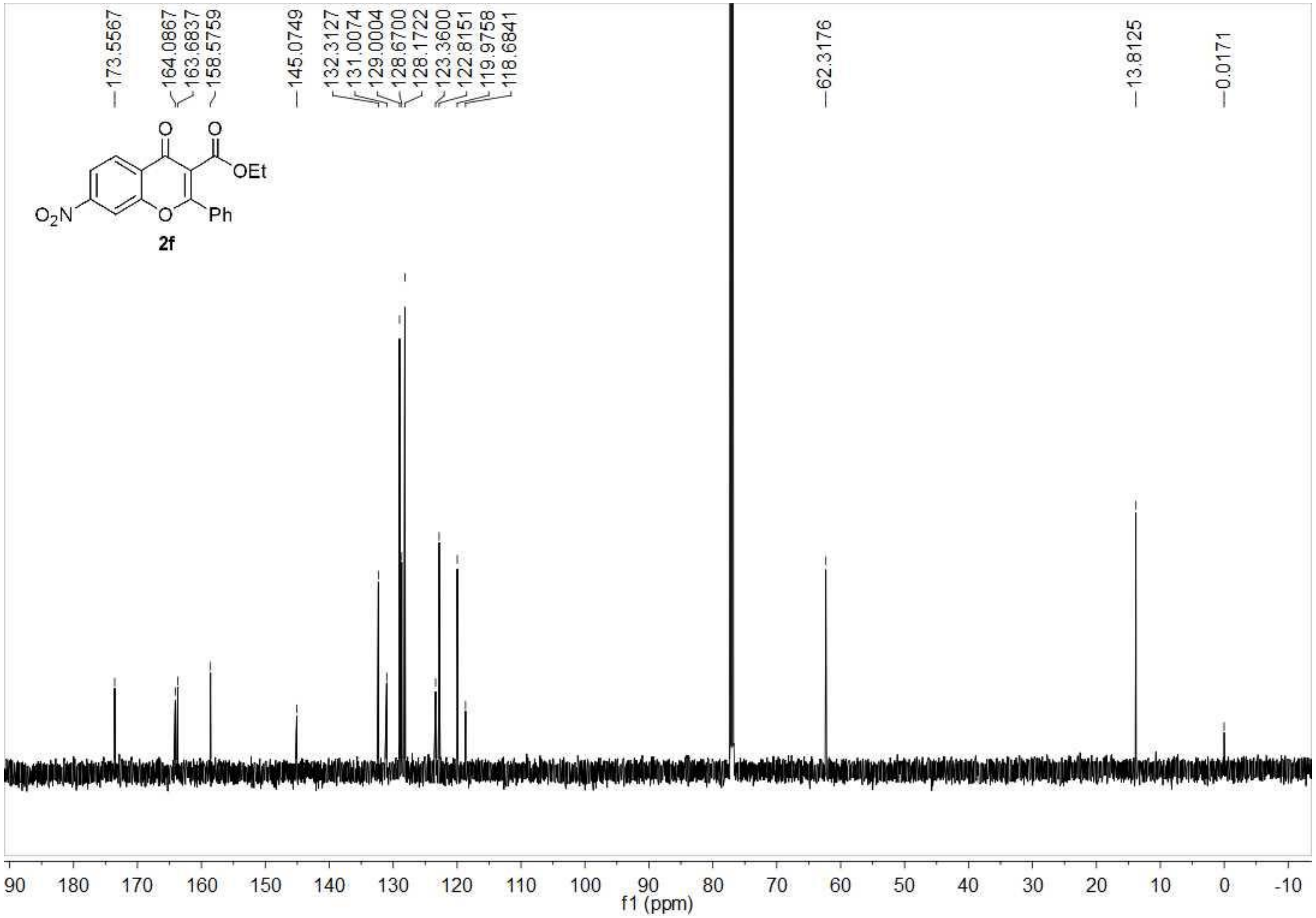

173.5567
164.0867
163.6837
158.5759
145.0749
132.3127
131.0074
129.0004
128.6700
128.1722
123.3600
122.8151
119.9758
118.6841
62.3176
13.8125
-0.0171
O
O
OEt
O_2N
O
Ph
2f
90
180
170
160
150
140
130
120
110
100
90
80
70
60
50
40
30
20
10
0
-10
f1 (ppm)

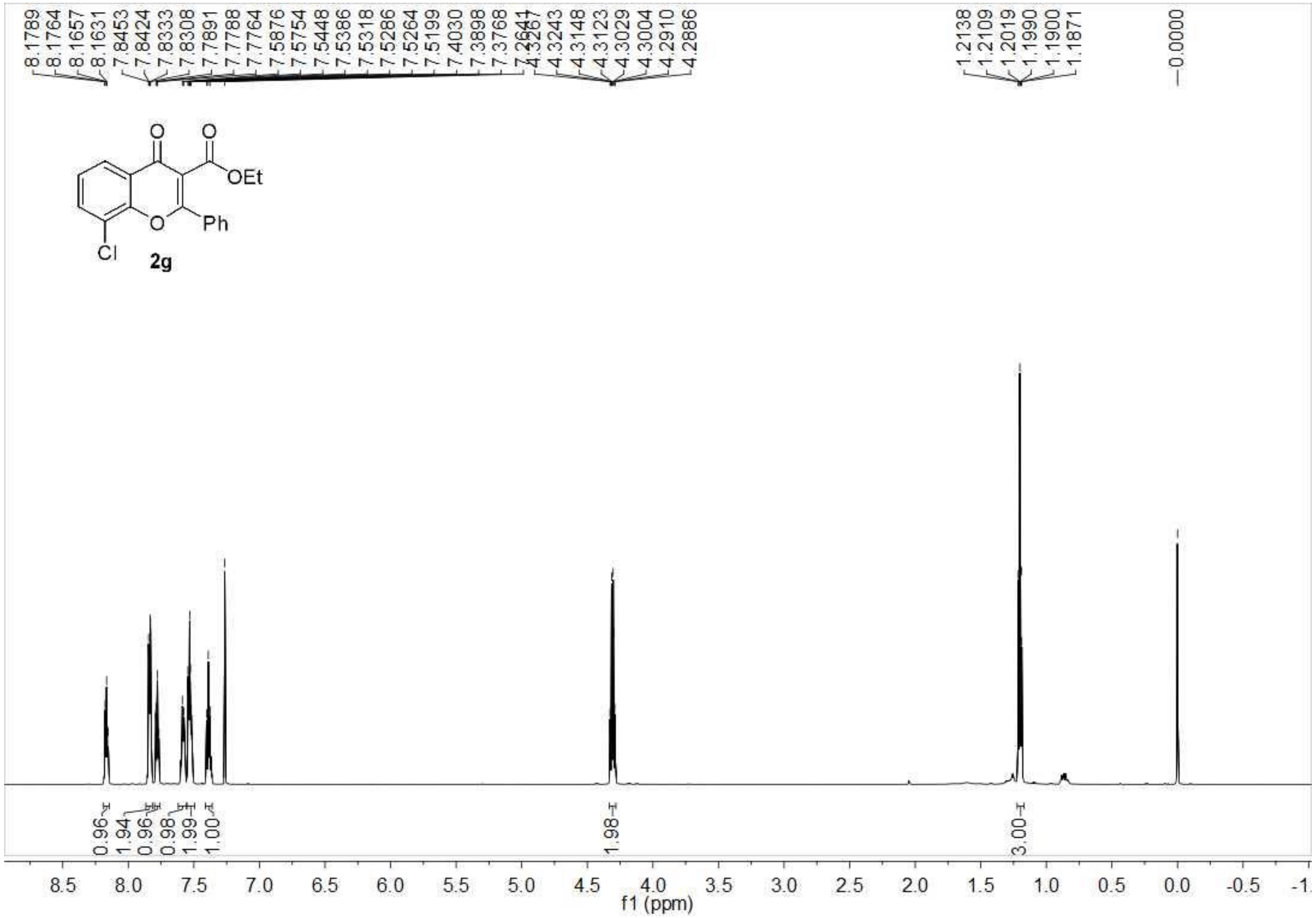
OEt
Ph
Cl
2g
f1 (ppm)

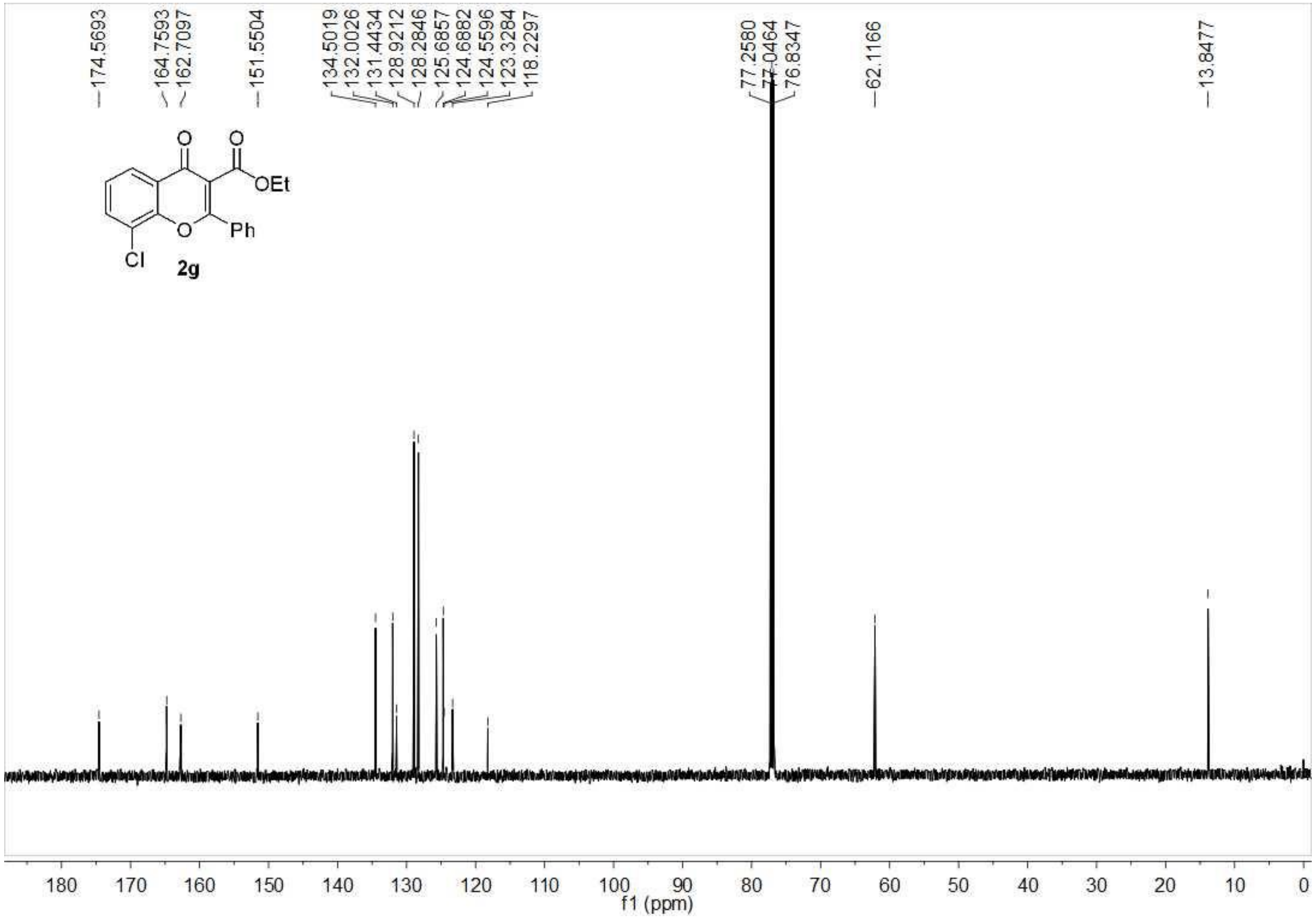
174.5693
164.7593
162.7097
151.5504
134.5019
132.0026
131.4434
128.9212
128.2846
125.6857
124.6882
124.5596
123.3284
118.2297
77.2580
77.0464
76.8347
62.1166
13.8477
OEt
Ph
Cl
2g
180 170 160 150 140 130 120 110 100 90 80 70 60 50 40 30 20 10 0
f1 (ppm)

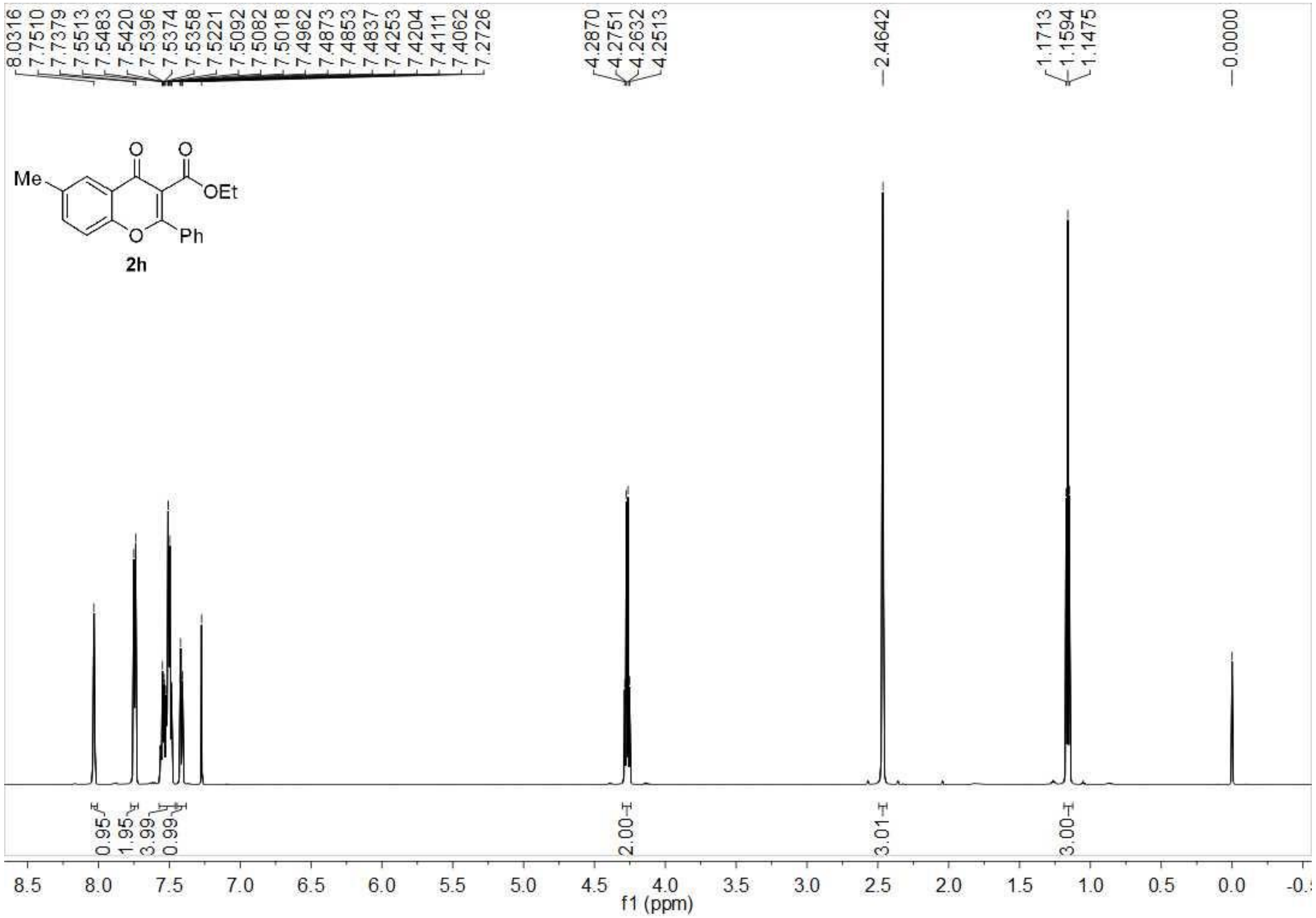
8.0316
7.7510
7.7379
7.5513
7.5483
7.5420
7.5396
7.5374
7.5358
7.5221
7.5092
7.5082
7.5018
7.4962
7.4873
7.4853
7.4837
7.4253
7.4204
7.4111
7.4062
7.2726
4.2870
4.2751
4.2632
4.2513
2.4642
1.1713
1.1594
1.1475
0.0000
Me
O
O
OEt
O
Ph
2h
0.95
1.95
3.99
0.99
2.00
3.01
3.00
f1 (ppm)

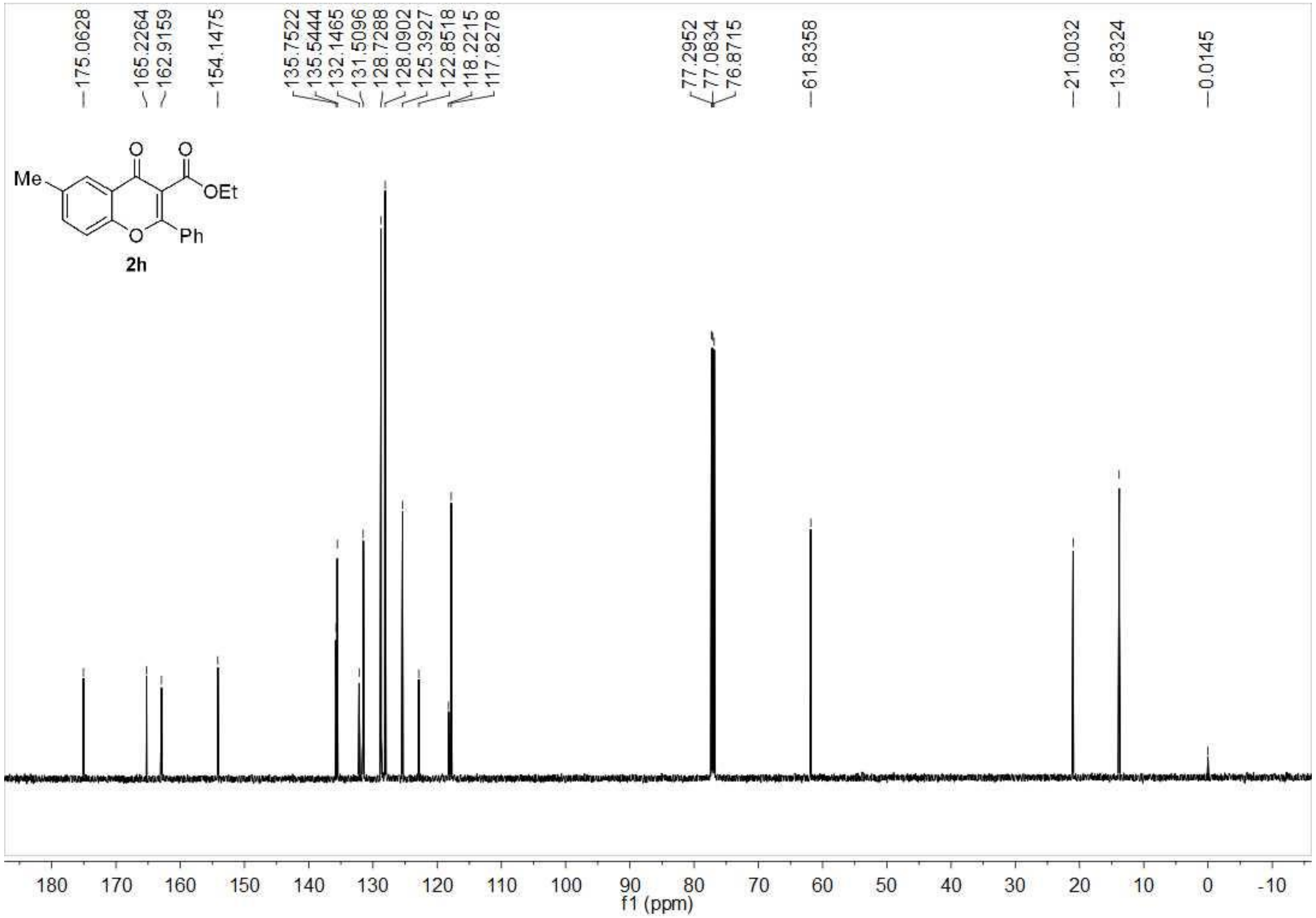
175.0628
165.2264
162.9159
154.1475
135.7522
135.5444
132.1465
131.5096
128.7288
128.0902
125.3927
122.8518
118.2215
117.8278
77.2952
77.0834
76.8715
61.8358
21.0032
13.8324
0.0145
Me
O
O
OEt
O
Ph
2h
180
170
160
150
140
130
120
110
100
90
80
70
60
50
40
30
20
10
0
-10
f1 (ppm)

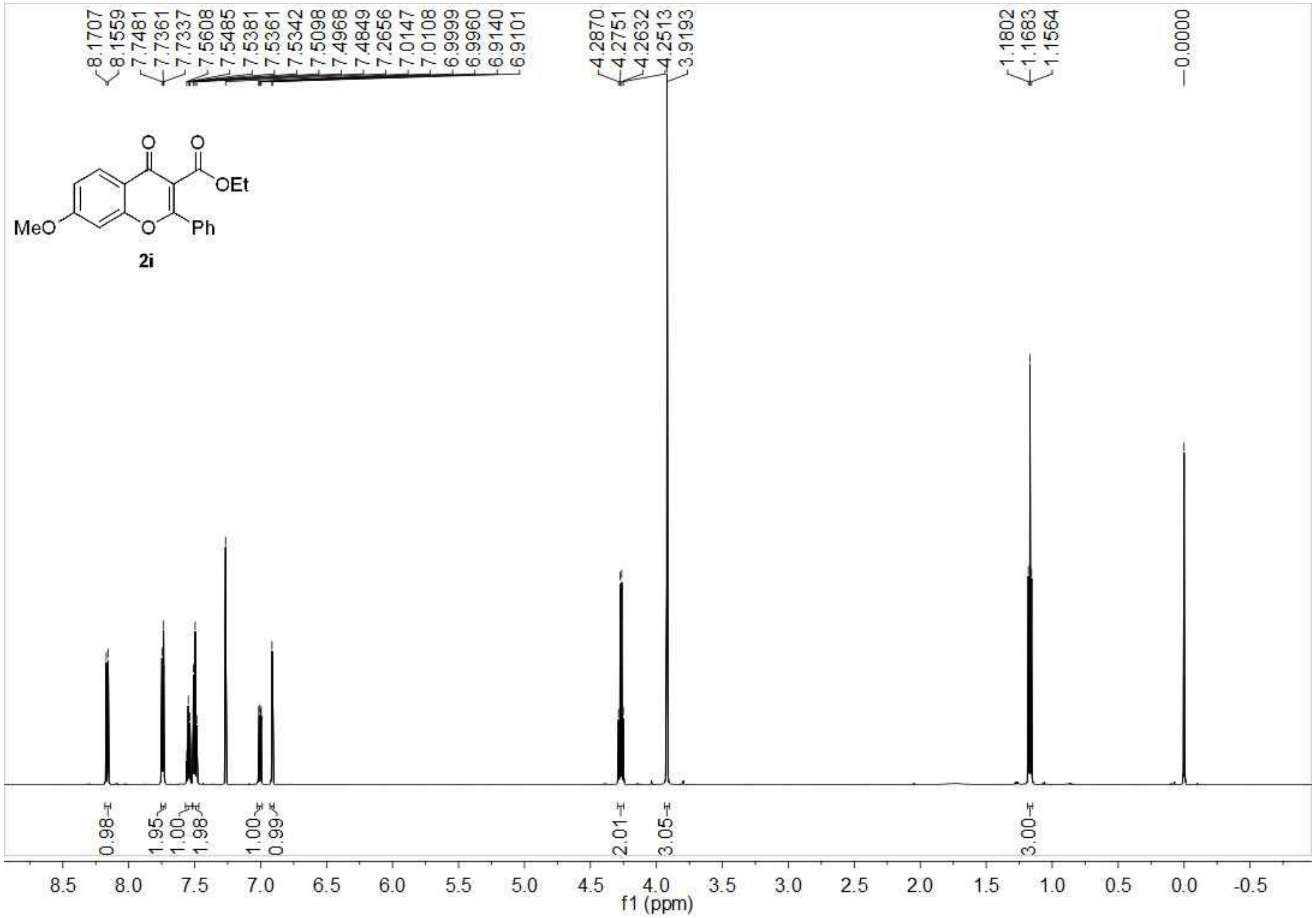
8.1707
8.1559
7.7481
7.7361
7.7337
7.5608
7.5485
7.5381
7.5361
7.5342
7.5098
7.4968
7.4849
7.2656
7.0147
7.0108
6.9999
6.9960
6.9140
6.9101
4.2870
4.2751
4.2632
4.2513
3.9193
1.1802
1.1683
1.1564
0.0000
OEt
MeO
Ph
2i
0.98
1.95
1.00
1.98
1.00
0.99
2.01
3.05
3.00
8.5
8.0
7.5
7.0
6.5
6.0
5.5
5.0
4.5
4.0
3.5
3.0
2.5
2.0
1.5
1.0
0.5
0.0
-0.5
f1 (ppm)

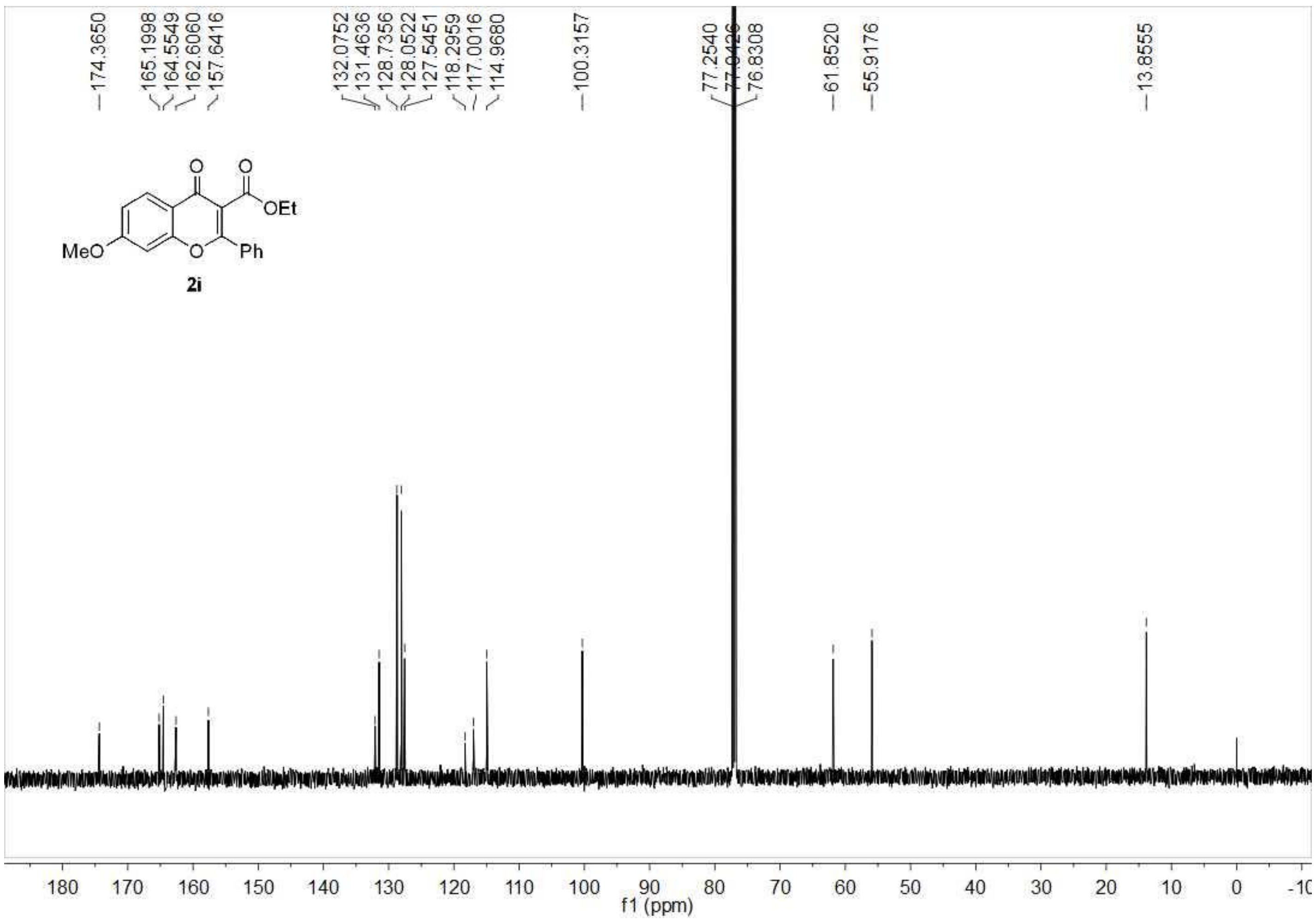

174.3650
165.1998
164.5549
162.6060
157.6416
132.0752
131.4636
128.7356
128.0522
127.5451
118.2959
117.0016
114.9680
100.3157
77.2540
76.8308
61.8520
55.9176
13.8555
O
O
OEt
MeO
O
Ph
2i
180
170
160
150
140
130
120
110
100
90
80
70
60
50
40
30
20
10
0
f1 (ppm)

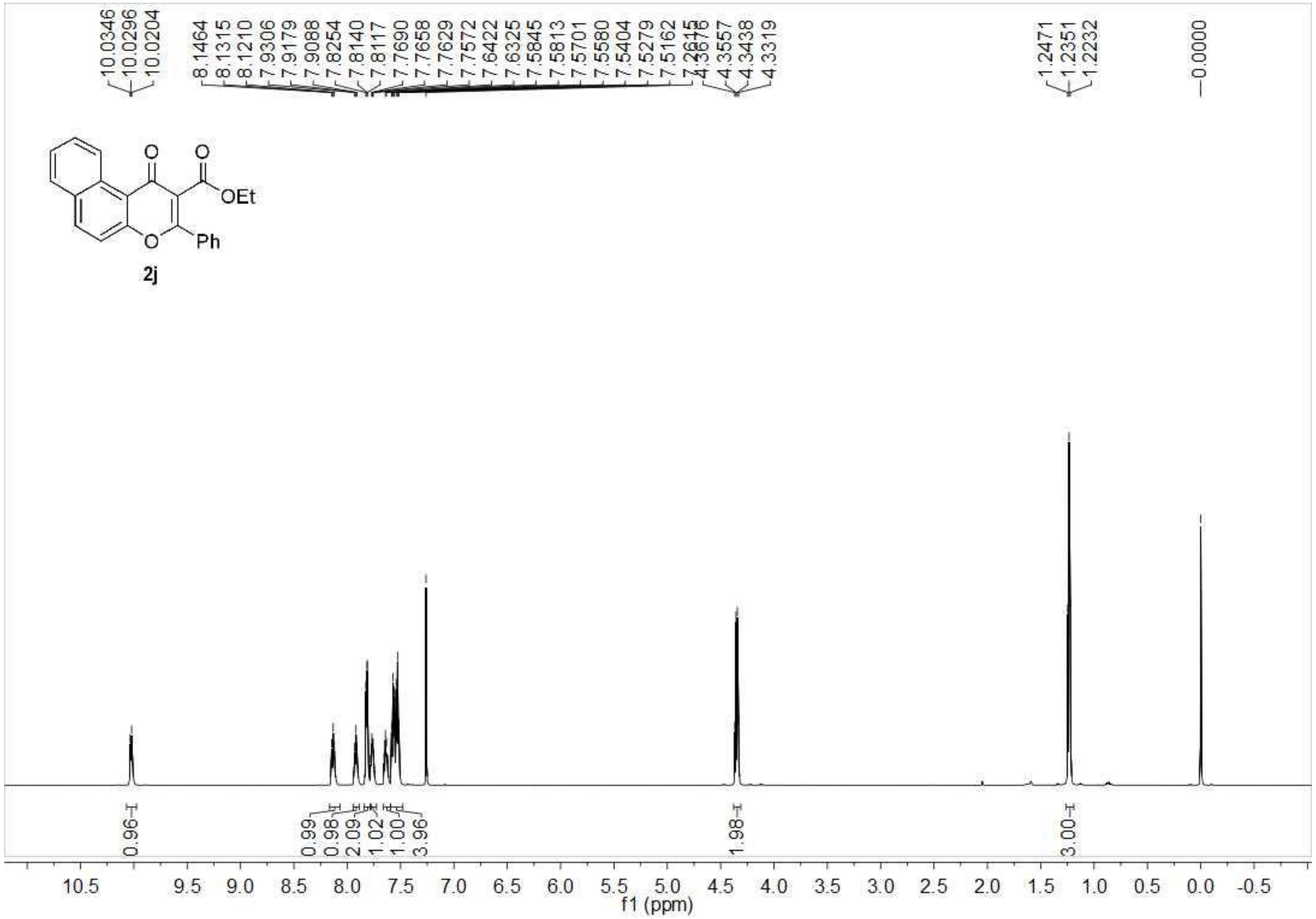
10.0346
10.0296
10.0204
8.1464
8.1315
8.1210
7.9306
7.9179
7.9088
7.8254
7.8140
7.8117
7.7690
7.7658
7.7629
7.7572
7.6422
7.6325
7.5845
7.5813
7.5701
7.5580
7.5404
7.5279
7.5162
7.2615
4.3676
4.3557
4.3438
4.3319
1.2471
1.2351
1.2232
0.0000
O
O
OEt
O
Ph
2j
0.96
0.99
0.98
2.09
1.02
1.00
3.96
1.98
3.00
10.5 9.5 9.0 8.5 8.0 7.5 7.0 6.5 6.0 5.5 5.0 4.5 4.0 3.5 3.0 2.5 2.0 1.5 1.0 0.5 0.0 -0.5
f1 (ppm)

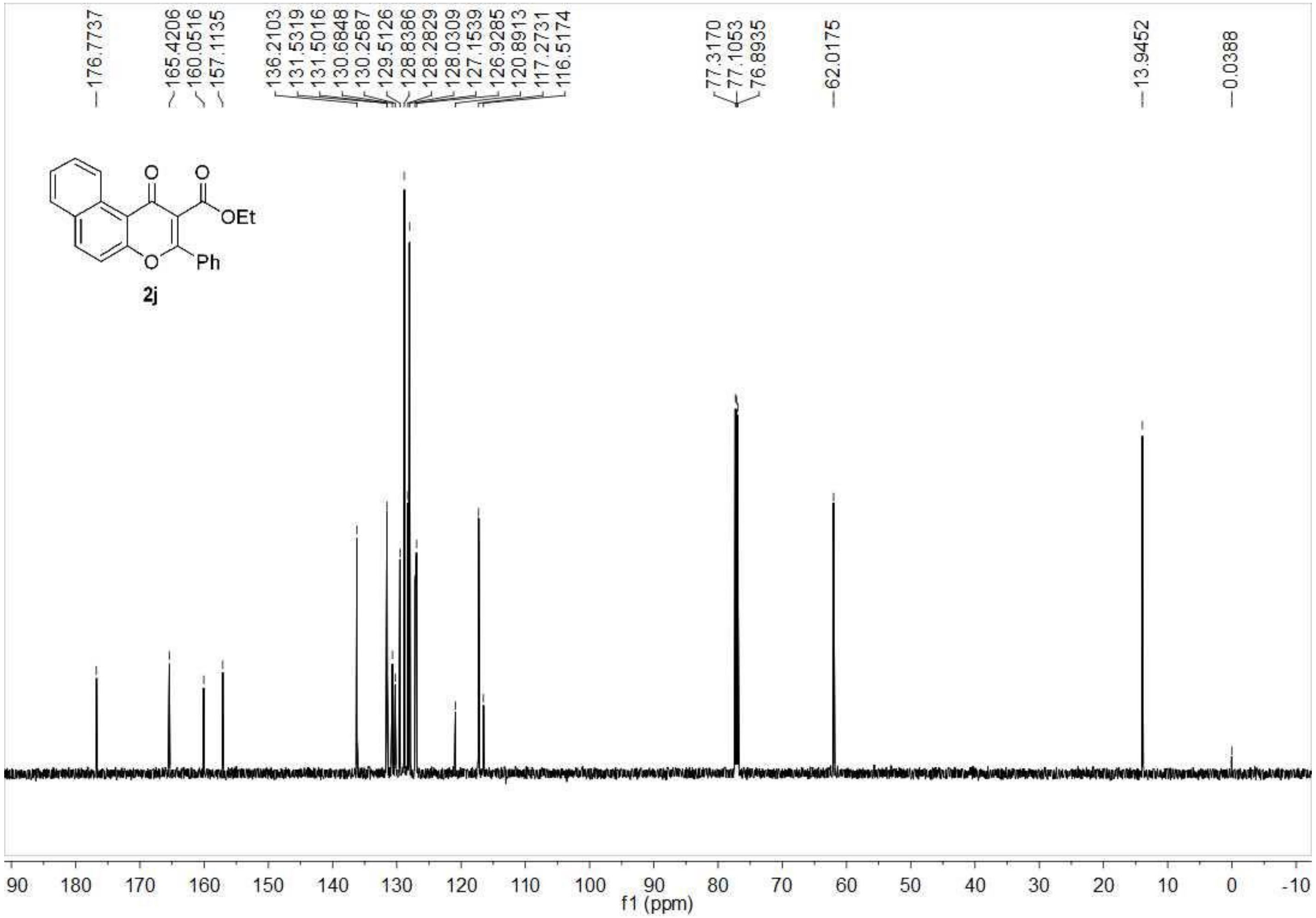

176.7737
165.4206
160.0516
157.1135
136.2103
131.5319
131.5016
130.6848
130.2587
129.5126
128.8386
128.2829
128.0309
127.1539
126.9285
120.8913
117.2731
116.5174
77.3170
77.1053
76.8935
62.0175
13.9452
0.0388
O
O
OEt
O
Ph
2j
f1 (ppm)

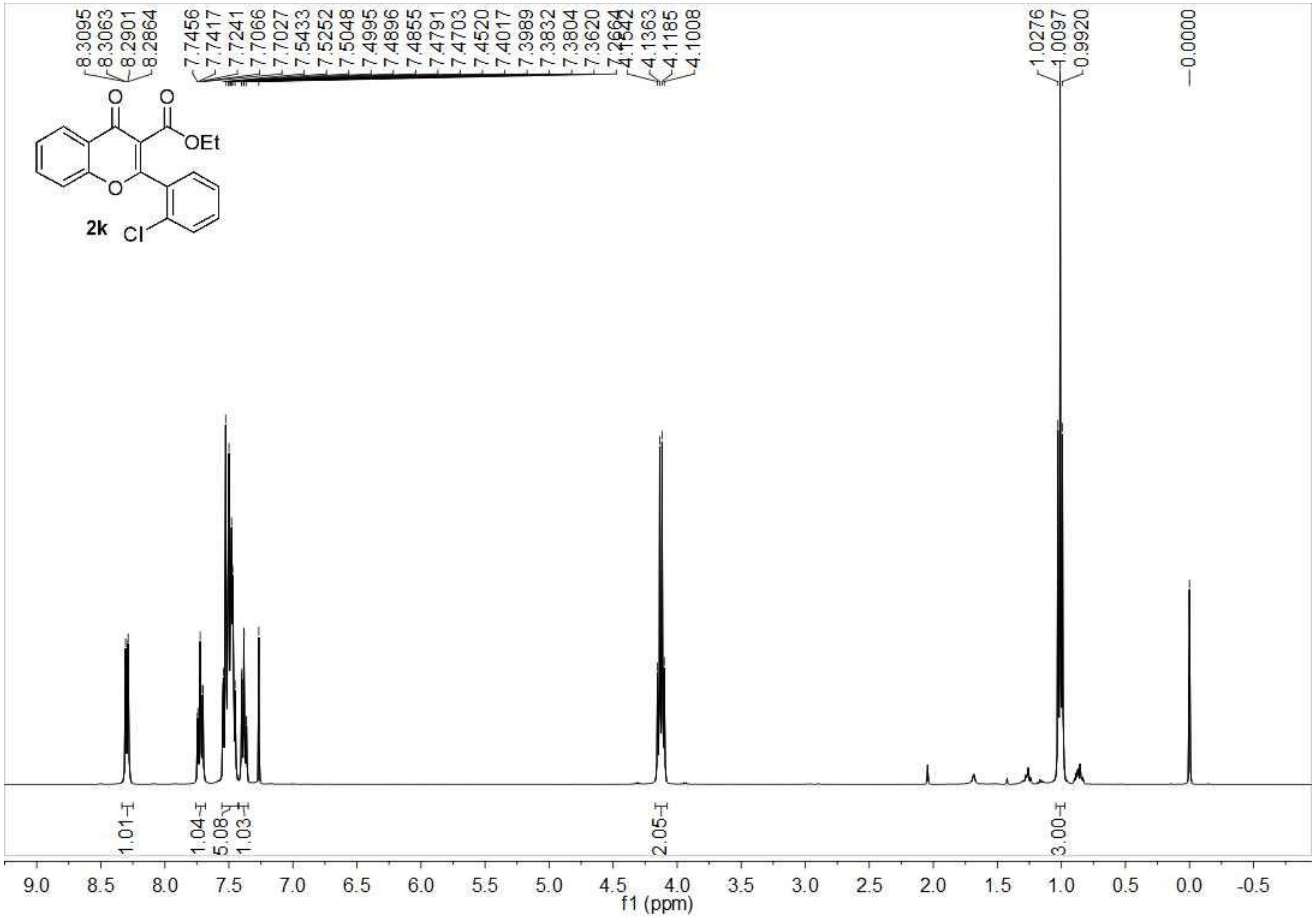

8.3095
8.3063
8.2901
8.2864
7.7456
7.7417
7.7241
7.7066
7.7027
7.5433
7.5252
7.5048
7.4995
7.4896
7.4855
7.4791
7.4703
7.4520
7.4017
7.3989
7.3832
7.3804
7.3620
7.2664
4.1542
4.1363
4.1185
4.1008
1.0276
1.0097
0.9920
0.0000
OEt
2k
Cl
1.01
1.04
5.08
1.03
2.05
3.00
9.0
8.5
8.0
7.5
7.0
6.5
6.0
5.5
5.0
4.5
4.0
3.5
3.0
2.5
2.0
1.5
1.0
0.5
0.0
-0.5
f1 (ppm)

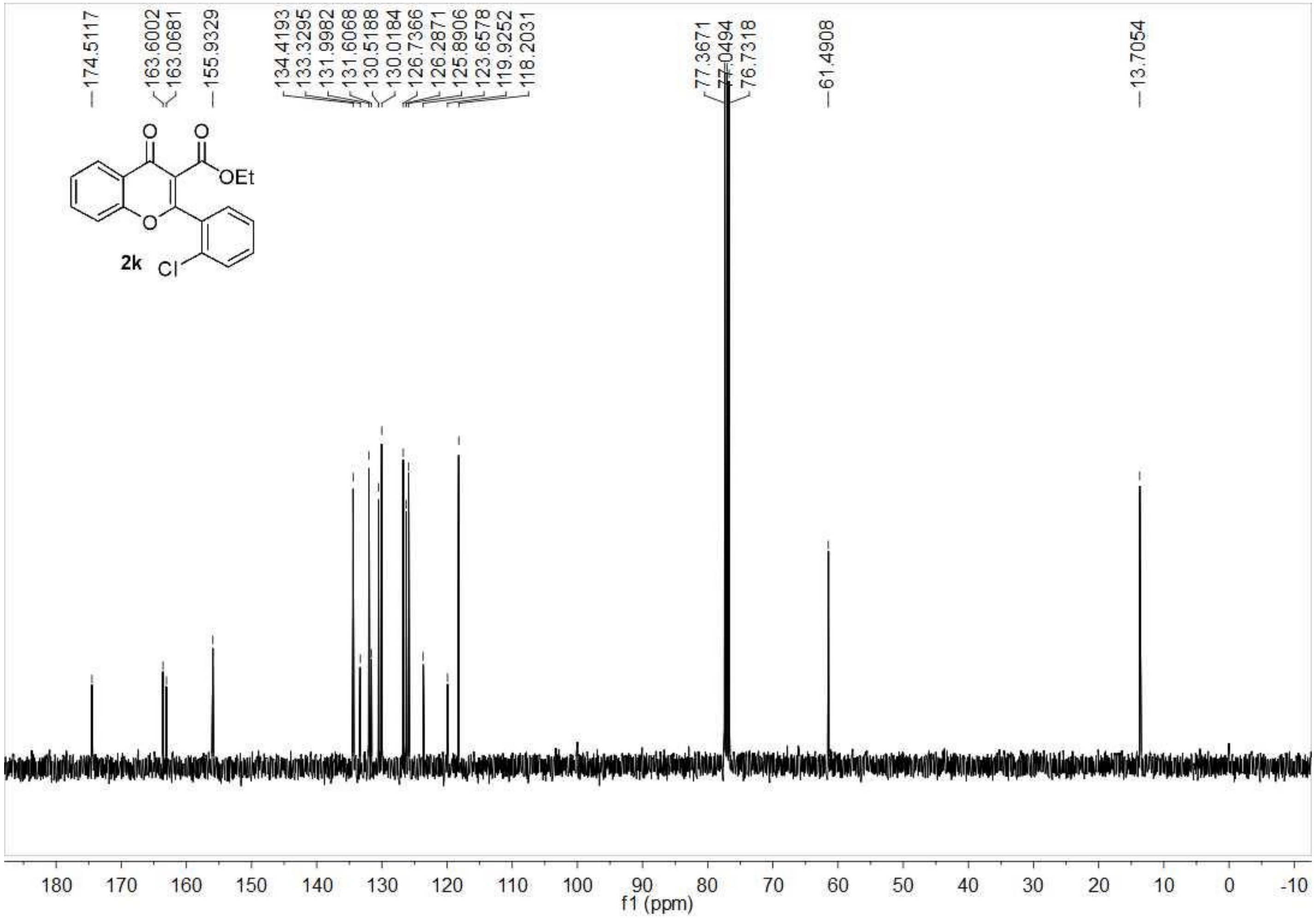
174.5117
163.6002
163.0681
155.9329
134.4193
133.3295
131.9982
131.6068
130.5188
130.0184
126.7366
126.2871
125.8906
123.6578
119.9252
118.2031
77.3671
77.0494
76.7318
61.4908
13.7054
OEt
2k
Cl
180 170 160 150 140 130 120 110 100 90 80 70 60 50 40 30 20 10 0 -10
f1 (ppm)

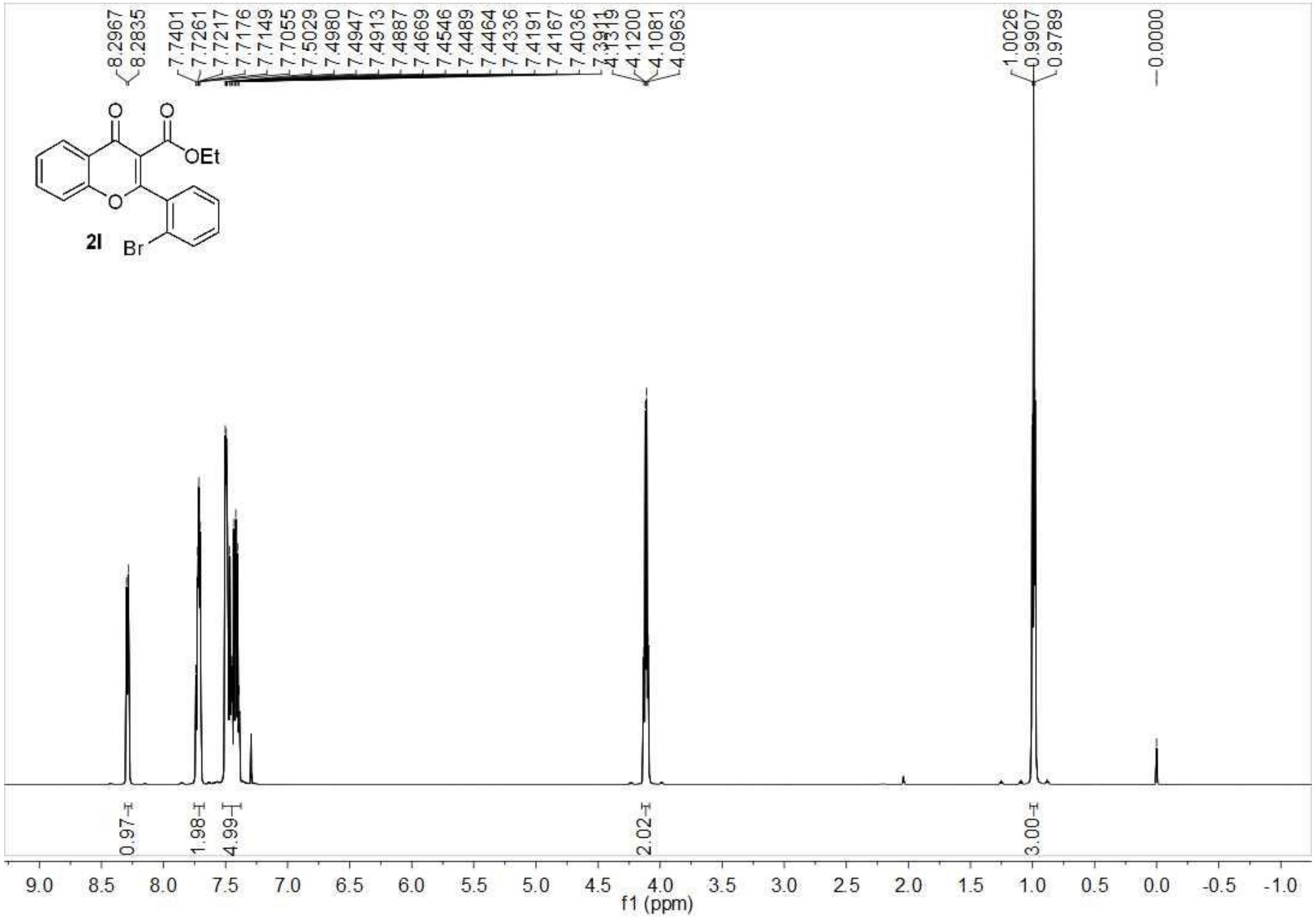
8.2967
8.2835
7.7401
7.7261
7.7217
7.7176
7.7149
7.7055
7.5029
7.4980
7.4947
7.4913
7.4887
7.4669
7.4546
7.4489
7.4464
7.4336
7.4191
7.4167
7.4036
7.3911
4.1319
4.1200
4.1081
4.0963
1.0026
0.9907
0.9789
0.0000
O
O
OEt
O
Br
2l
0.97
1.98
4.99
2.02
3.00
9.0 8.5 8.0 7.5 7.0 6.5 6.0 5.5 5.0 4.5 4.0 3.5 3.0 2.5 2.0 1.5 1.0 0.5 0.0 -0.5 -1.0
f1 (ppm)

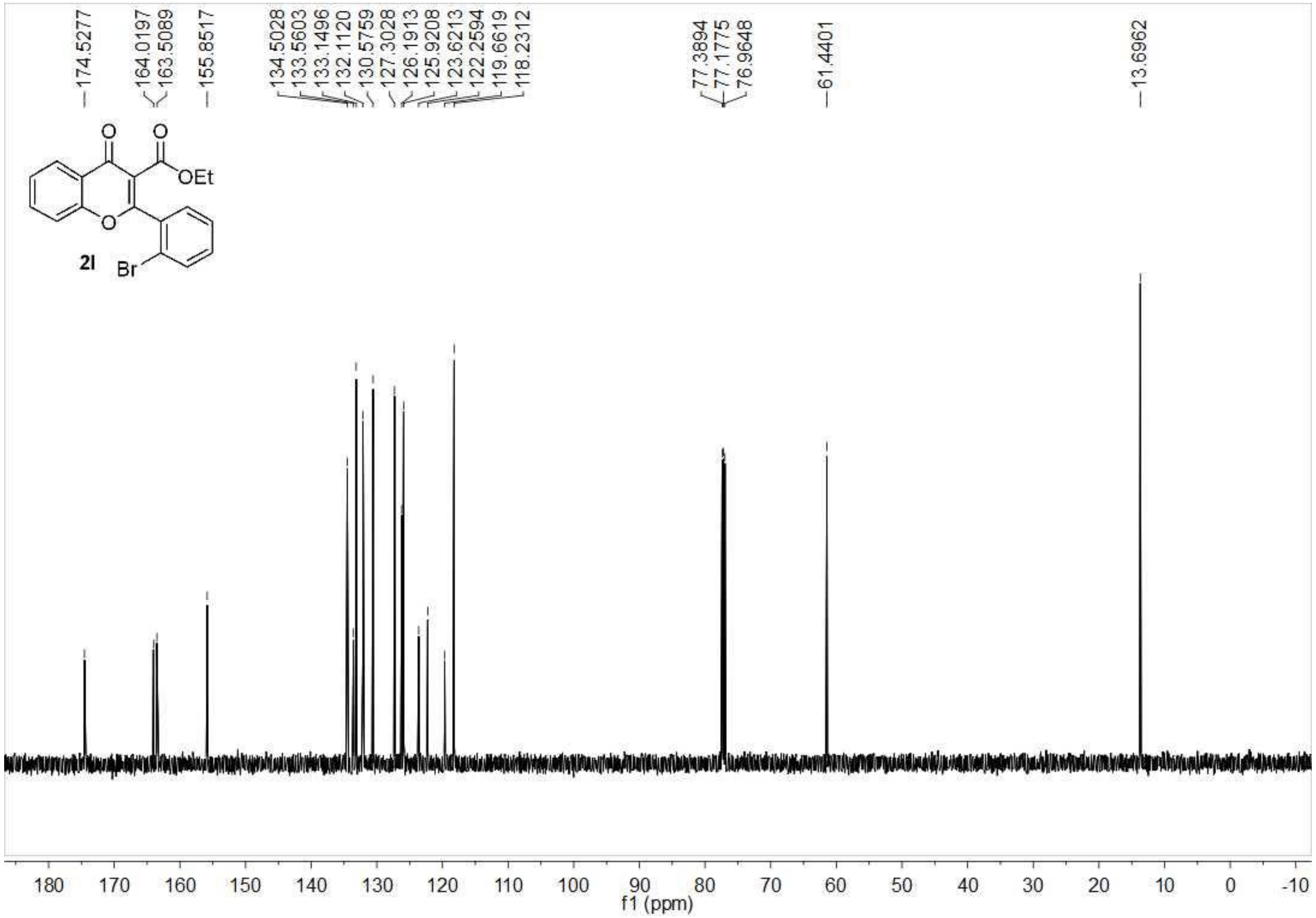
174.5277
164.0197
163.5089
155.8517
134.5028
133.5603
133.1496
132.1120
130.5759
127.3028
126.1913
125.9208
123.6213
122.2594
119.6619
118.2312
77.3894
77.1775
76.9648
61.4401
13.6962
OEt
2l
Br
180 170 160 150 140 130 120 110 100 90 80 70 60 50 40 30 20 10 0 -10
f1 (ppm)

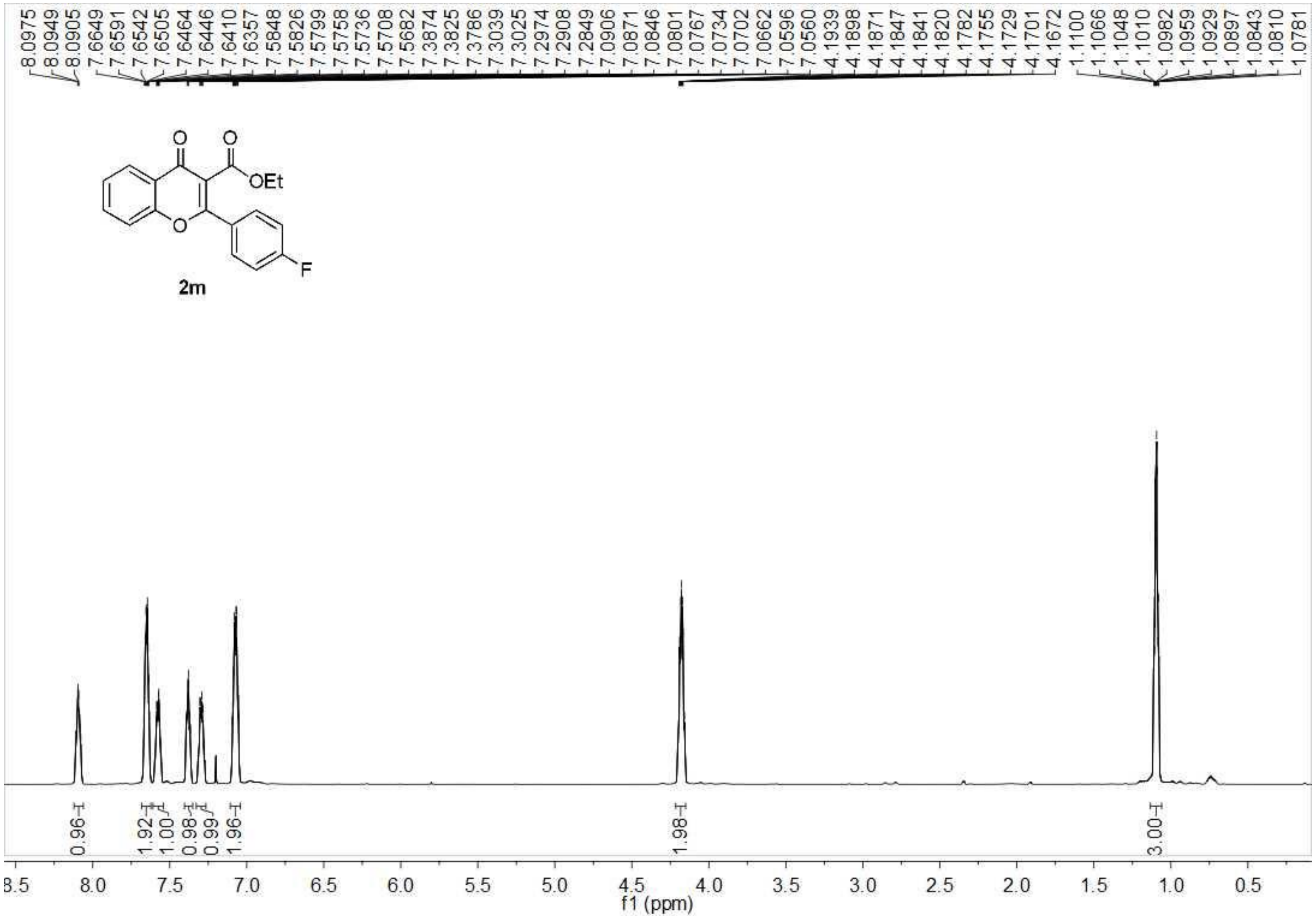

OEt
F
2m
8.0975
8.0949
8.0905
7.6649
7.6591
7.6542
7.6505
7.6464
7.6446
7.6410
7.6357
7.5848
7.5826
7.5799
7.5758
7.5736
7.5708
7.5682
7.3874
7.3825
7.3786
7.3039
7.3025
7.2974
7.2908
7.2849
7.0906
7.0871
7.0846
7.0801
7.0767
7.0734
7.0702
7.0662
7.0596
7.0560
4.1939
4.1898
4.1871
4.1847
4.1841
4.1820
4.1782
4.1755
4.1729
4.1701
4.1672
1.1100
1.1066
1.1048
1.1010
1.0982
1.0959
1.0929
1.0897
1.0843
1.0810
1.0781
0.96
1.92
1.00
0.98
0.99
1.96
1.98
3.00
f1 (ppm)

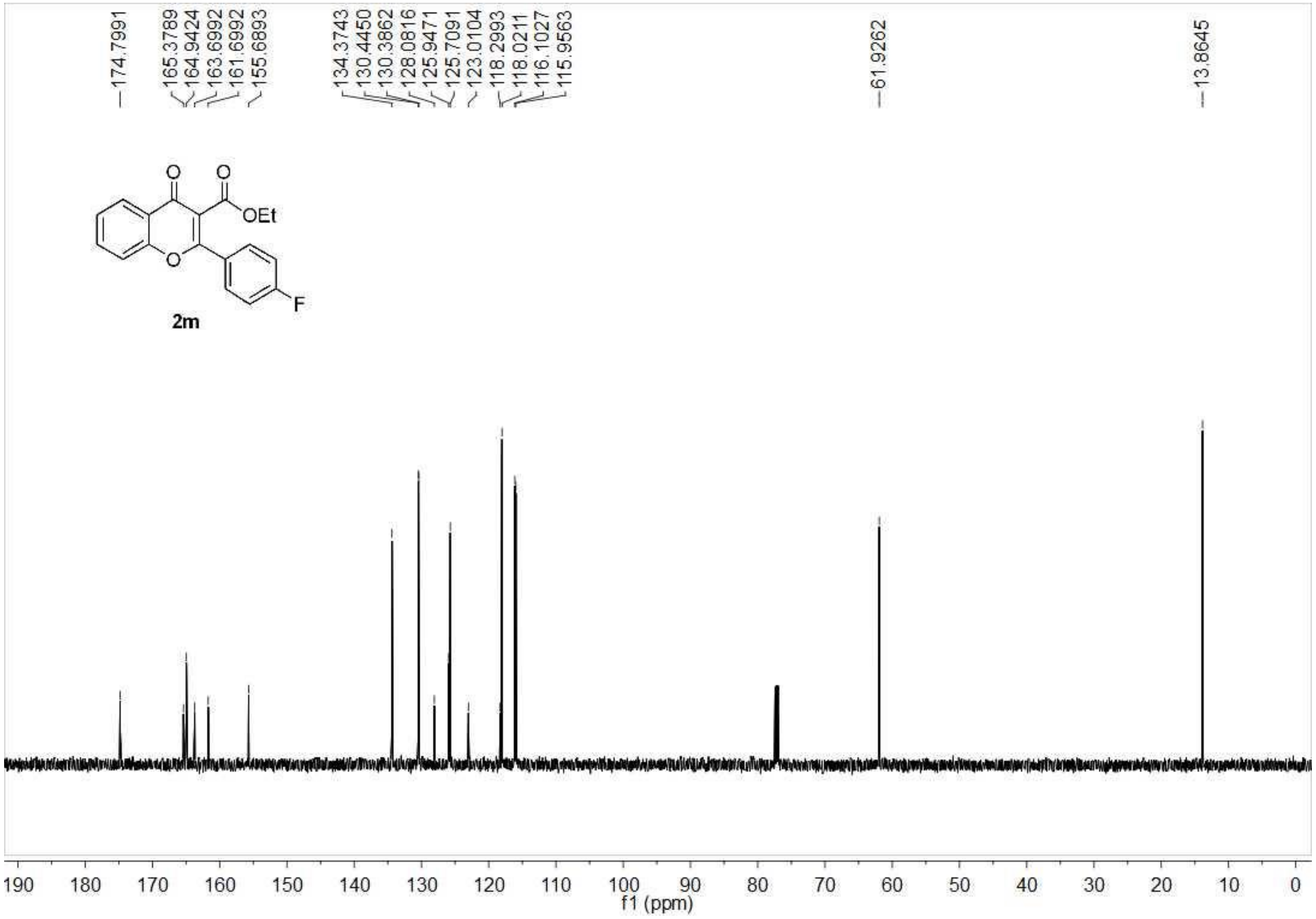
174.7991
165.3789
164.9424
163.6992
161.6992
155.6893
134.3743
130.4450
130.3862
128.0816
125.9471
125.7091
123.0104
118.2993
118.0211
116.1027
115.9563
61.9262
13.8645
O
O
OEt
O
F
2m
190 180 170 160 150 140 130 120 110 100 90 80 70 60 50 40 30 20 10 0
f1 (ppm)

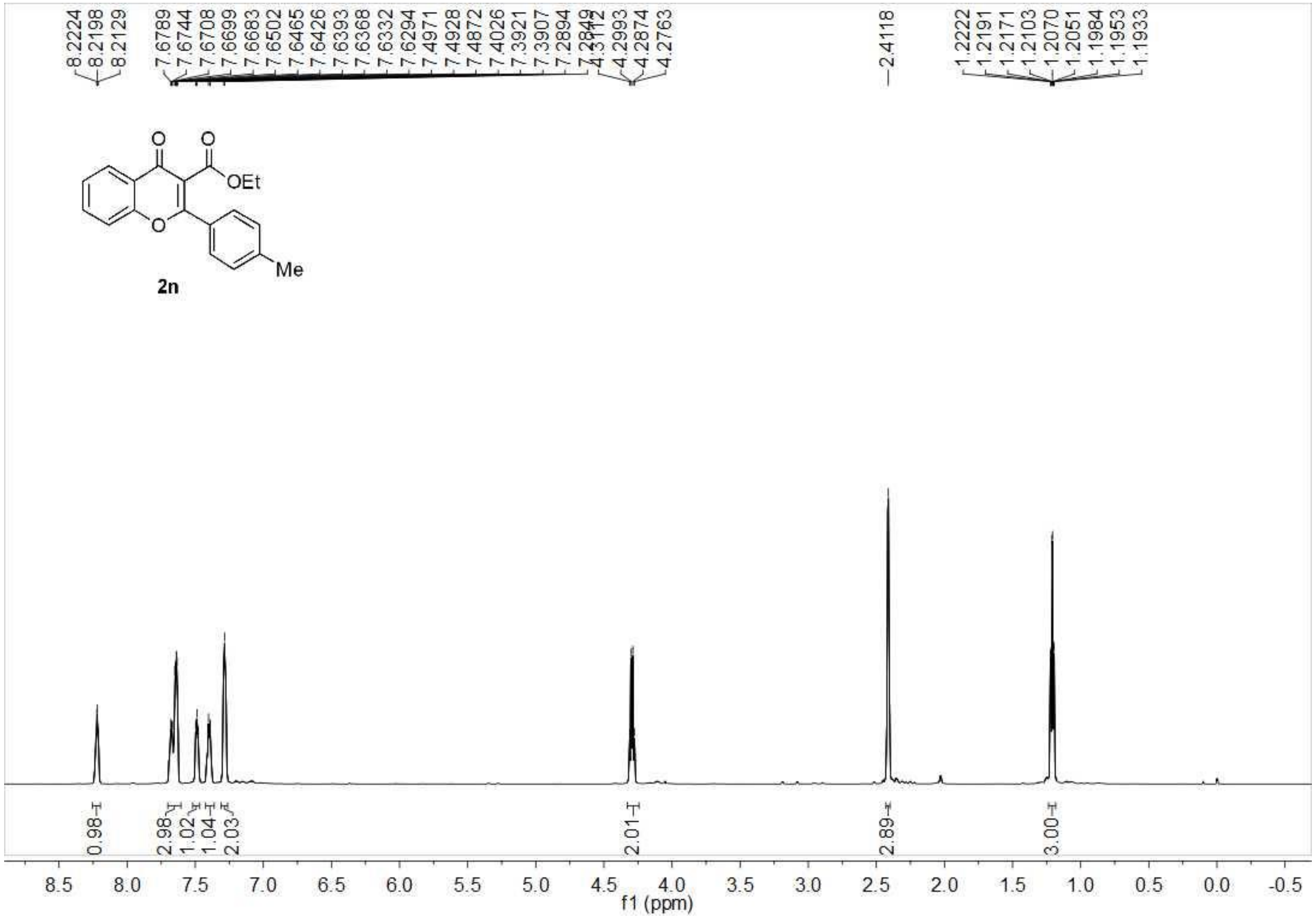

8.2224
8.2198
8.2129
7.6789
7.6744
7.6708
7.6699
7.6683
7.6502
7.6465
7.6426
7.6393
7.6368
7.6332
7.6294
7.4971
7.4928
7.4872
7.4026
7.3921
7.3907
7.2894
7.2849
4.3112
4.2993
4.2874
4.2763
2.4118
1.2222
1.2191
1.2171
1.2103
1.2070
1.2051
1.1984
1.1953
1.1933
O
O
OEt
O
Me
2n
0.98
2.98
1.02
1.04
2.03
2.01
2.89
3.00
8.5 8.0 7.5 7.0 6.5 6.0 5.5 5.0 4.5 4.0 3.5 3.0 2.5 2.0 1.5 1.0 0.5 0.0 -0.5
f1 (ppm)

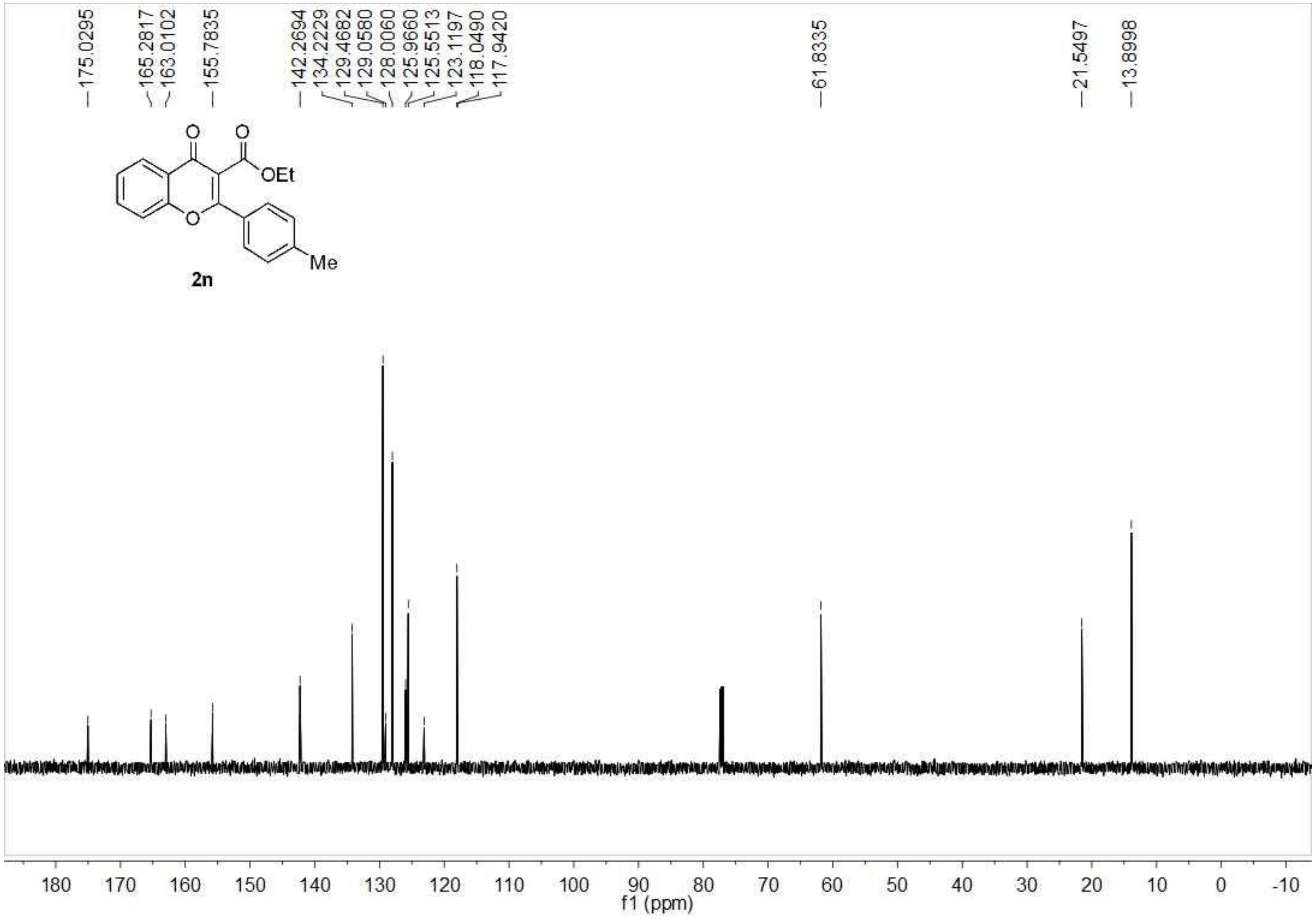
175.0295
165.2817
163.0102
155.7835
142.2694
134.2229
129.4682
129.0580
128.0060
125.9660
125.5513
123.1197
118.0490
117.9420
61.8335
21.5497
13.8998
O
O
OEt
O
Me
2n
180
170
160
150
140
130
120
110
100
90
80
70
60
50
40
30
20
10
0
-10
f1 (ppm)

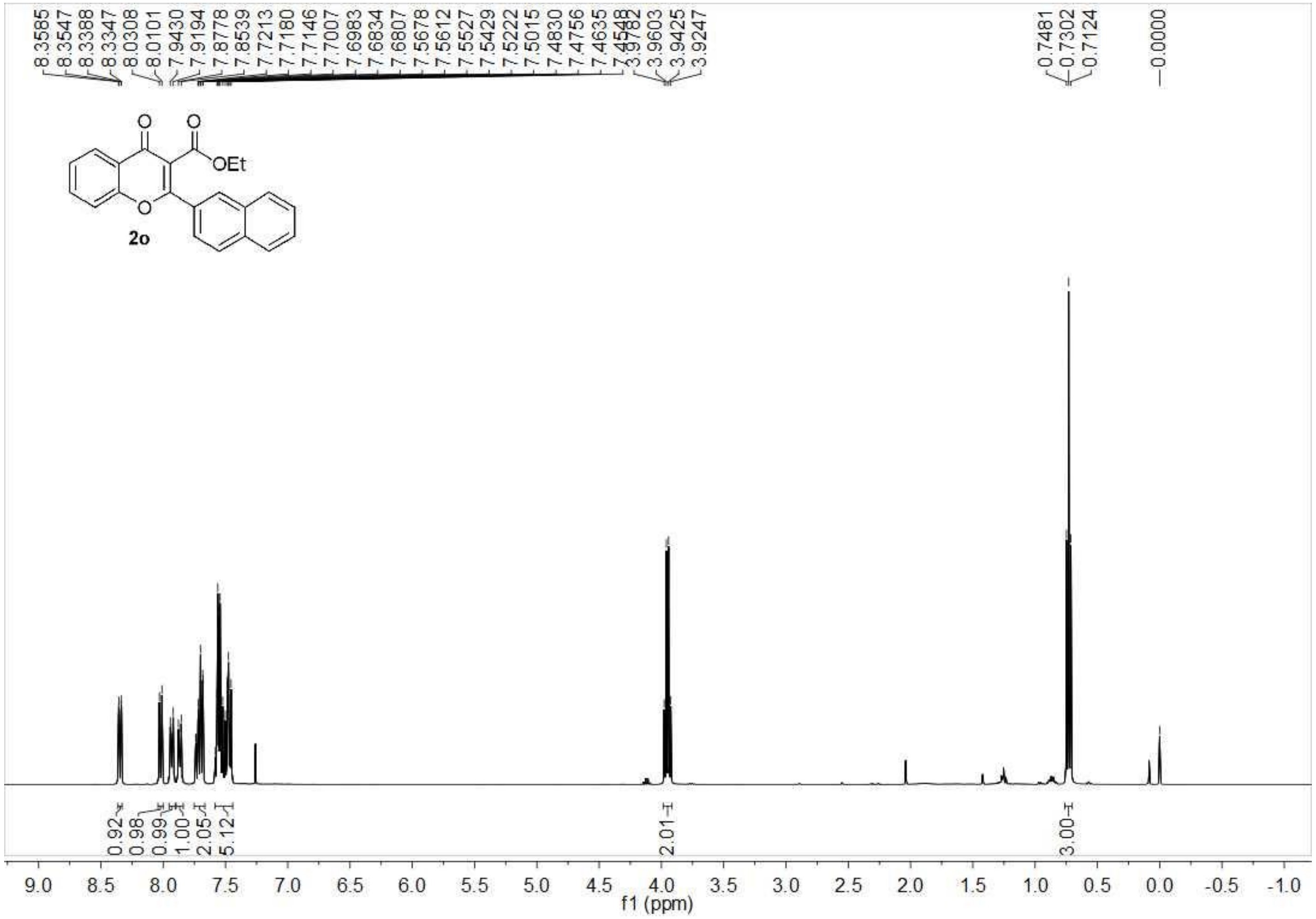

8.3585
8.3547
8.3388
8.3347
8.0308
8.0101
7.9430
7.9194
7.8778
7.8539
7.7213
7.7180
7.7146
7.7007
7.6983
7.6834
7.6807
7.5678
7.5612
7.5527
7.5429
7.5222
7.5015
7.4830
7.4756
7.4635
7.4548
3.9782
3.9603
3.9425
3.9247
0.7481
0.7302
0.7124
0.0000
OEt
2o
0.92
0.98
0.99
1.00
2.05
5.12
2.01
3.00
9.0 8.5 8.0 7.5 7.0 6.5 6.0 5.5 5.0 4.5 4.0 3.5 3.0 2.5 2.0 1.5 1.0 0.5 0.0 -0.5 -1.0
f1 (ppm)

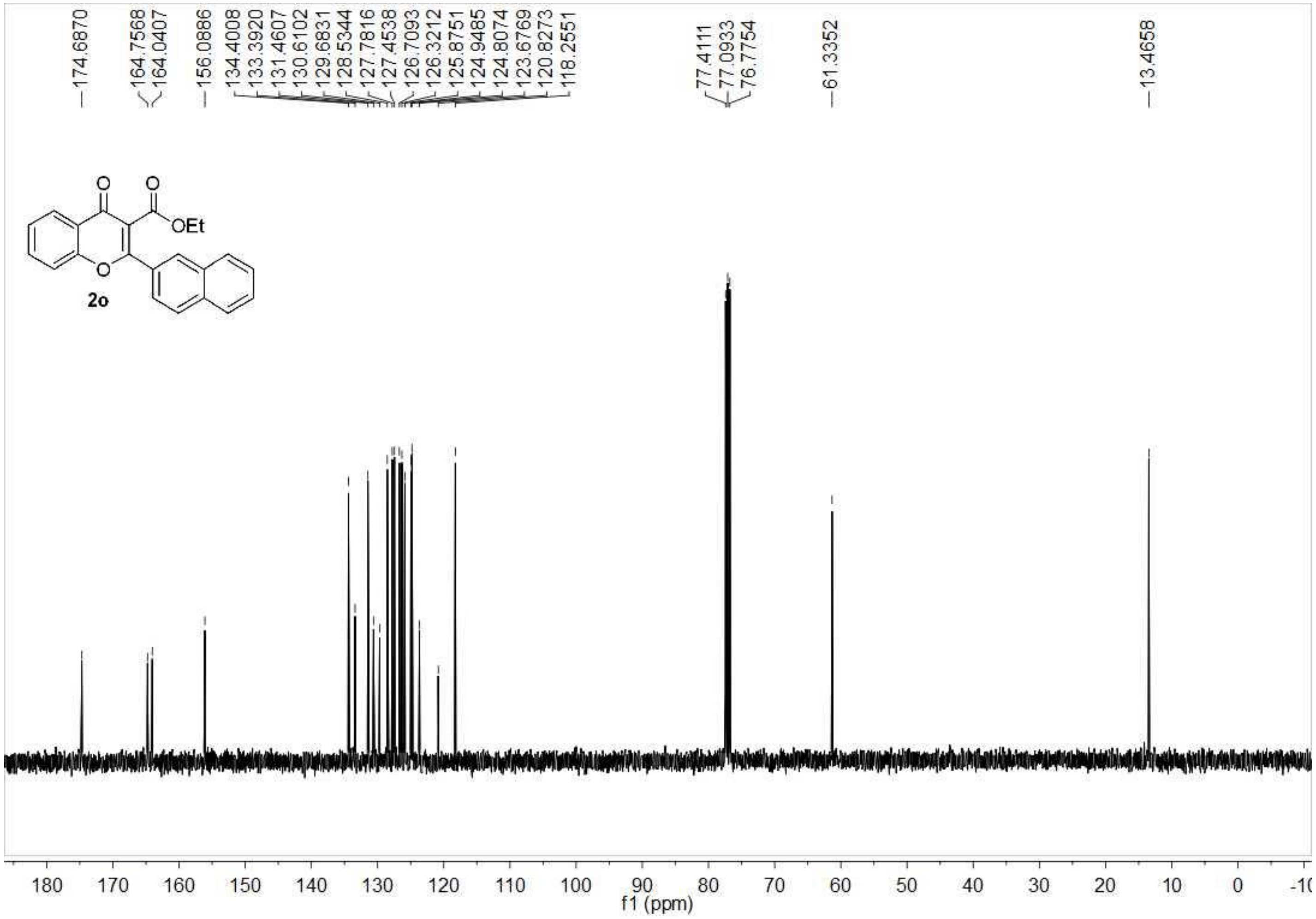
174.6870
164.7568
164.0407
156.0886
134.4008
133.3920
131.4607
130.6102
129.6831
128.5344
127.7816
127.4538
126.7093
126.3212
125.8751
124.9485
124.8074
123.6769
120.8273
118.2551
77.4111
77.0933
76.7754
61.3352
13.4658
OEt
2o
f1 (ppm)

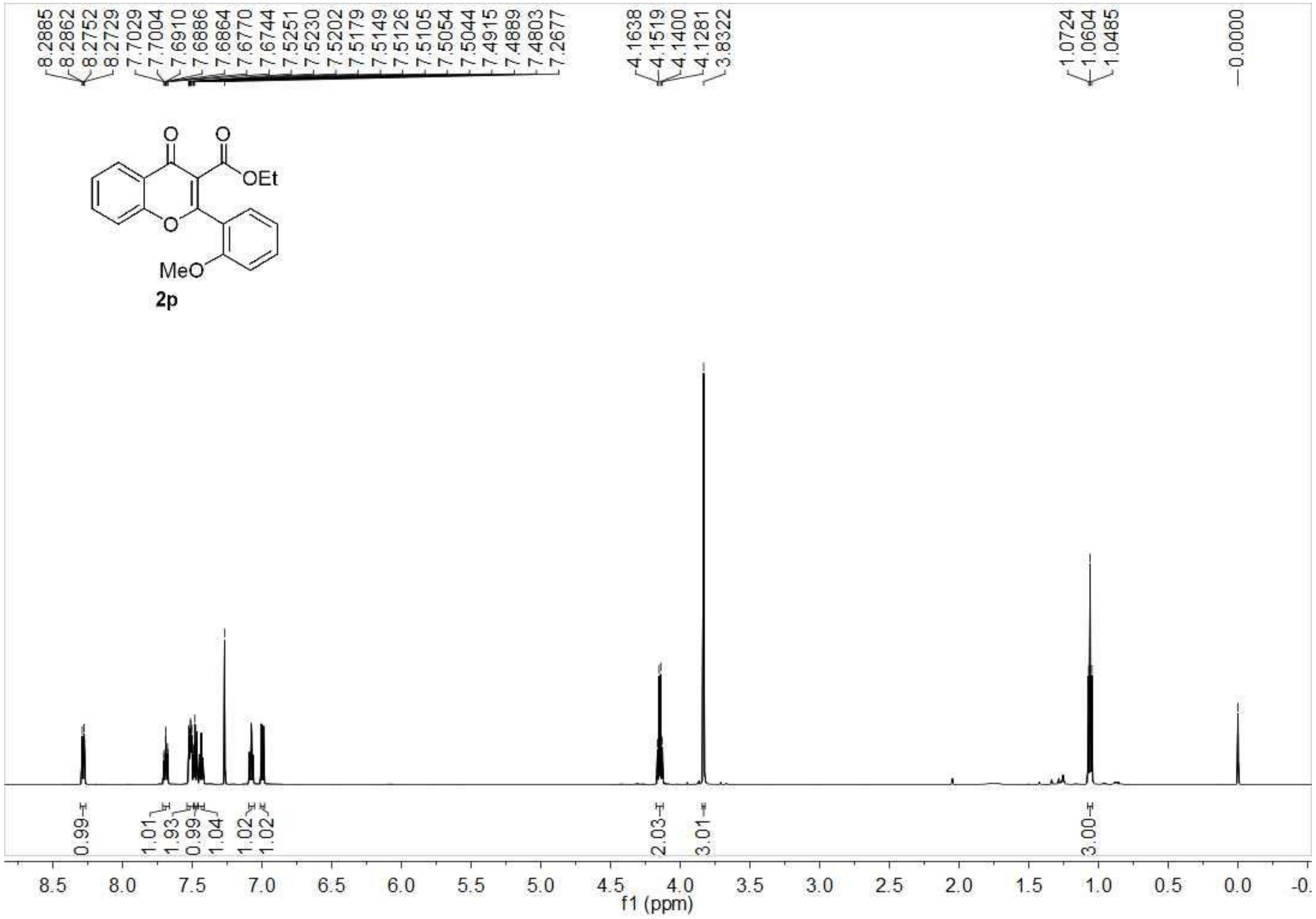
8.2885
8.2862
8.2752
8.2729
7.7029
7.7004
7.6910
7.6886
7.6864
7.6770
7.6744
7.5251
7.5230
7.5202
7.5179
7.5149
7.5126
7.5105
7.5054
7.5044
7.4915
7.4889
7.4803
7.2677
4.1638
4.1519
4.1400
4.1281
3.8322
1.0724
1.0604
1.0485
0.0000
OEt
MeO
2p
0.99
1.01
1.93
0.99
1.04
1.02
1.02
2.03
3.01
3.00
f1 (ppm)

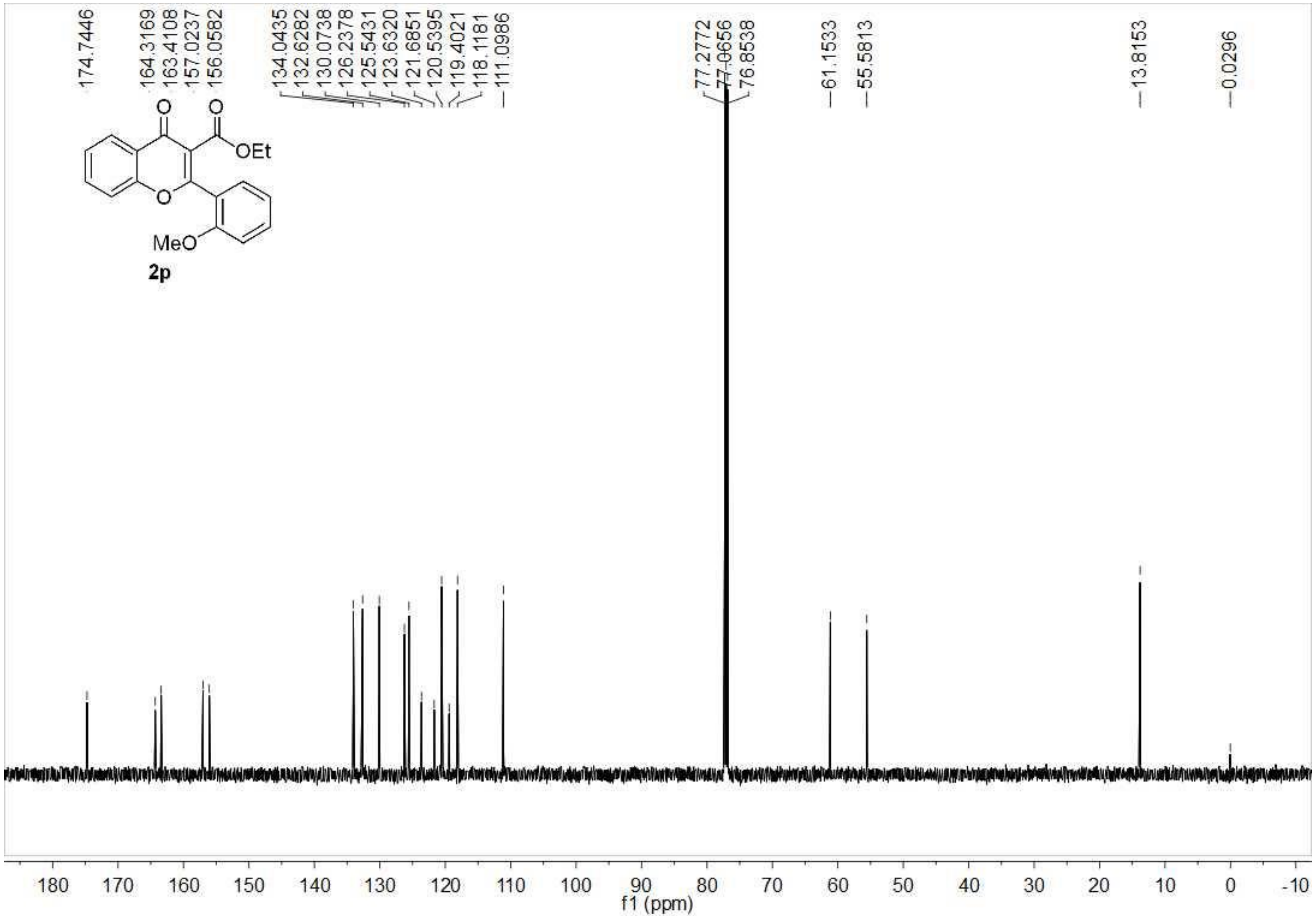
174.7446
164.3169
163.4108
157.0237
156.0582
134.0435
132.6282
130.0738
126.2378
125.5431
123.6320
121.6851
120.5395
119.4021
118.1181
111.0986
77.2772
77.0656
76.8538
61.1533
55.5813
13.8153
0.0296
OEt
MeO
2p
f1 (ppm)

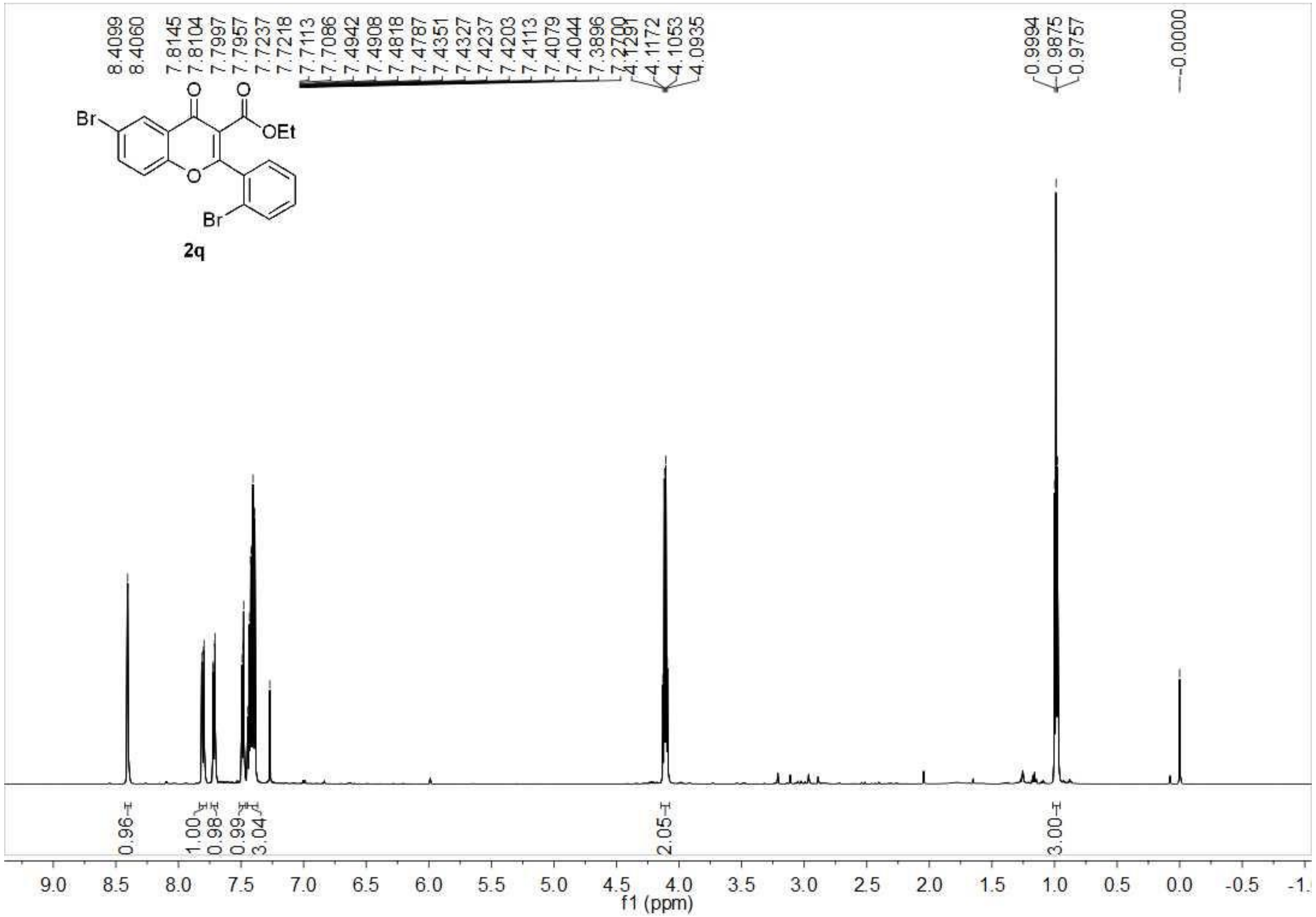

8.4099
8.4060
7.8145
7.8104
7.7997
7.7957
7.7237
7.7218
7.7113
7.7086
7.4942
7.4908
7.4818
7.4787
7.4351
7.4327
7.4237
7.4203
7.4113
7.4079
7.4044
7.3896
7.2700
4.1291
4.1172
4.1053
4.0935
0.9994
0.9875
0.9757
0.0000
Br
O
O
OEt
O
Br
2q
0.96
1.00
0.98
0.99
3.04
2.05
3.00
f1 (ppm)

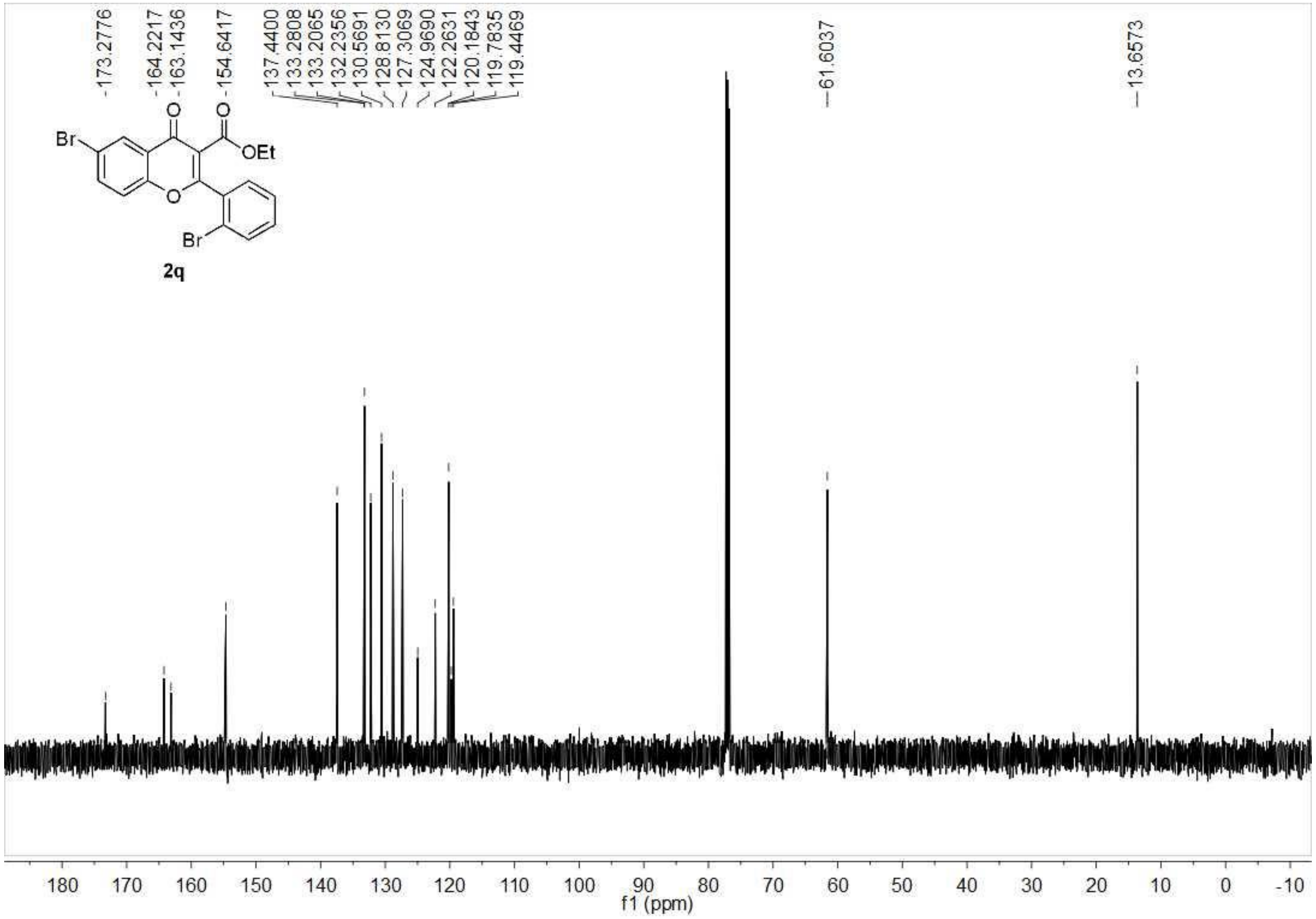
173.2776
164.2217
163.1436
154.6417
137.4400
133.2808
133.2065
132.2356
130.5691
128.8130
127.3069
124.9690
122.2631
120.1843
119.7835
119.4469
61.6037
13.6573
Br
O
O
OEt
O
Br
2q
180
170
160
150
140
130
120
110
100
90
80
70
60
50
40
30
20
10
0
-10
f1 (ppm)

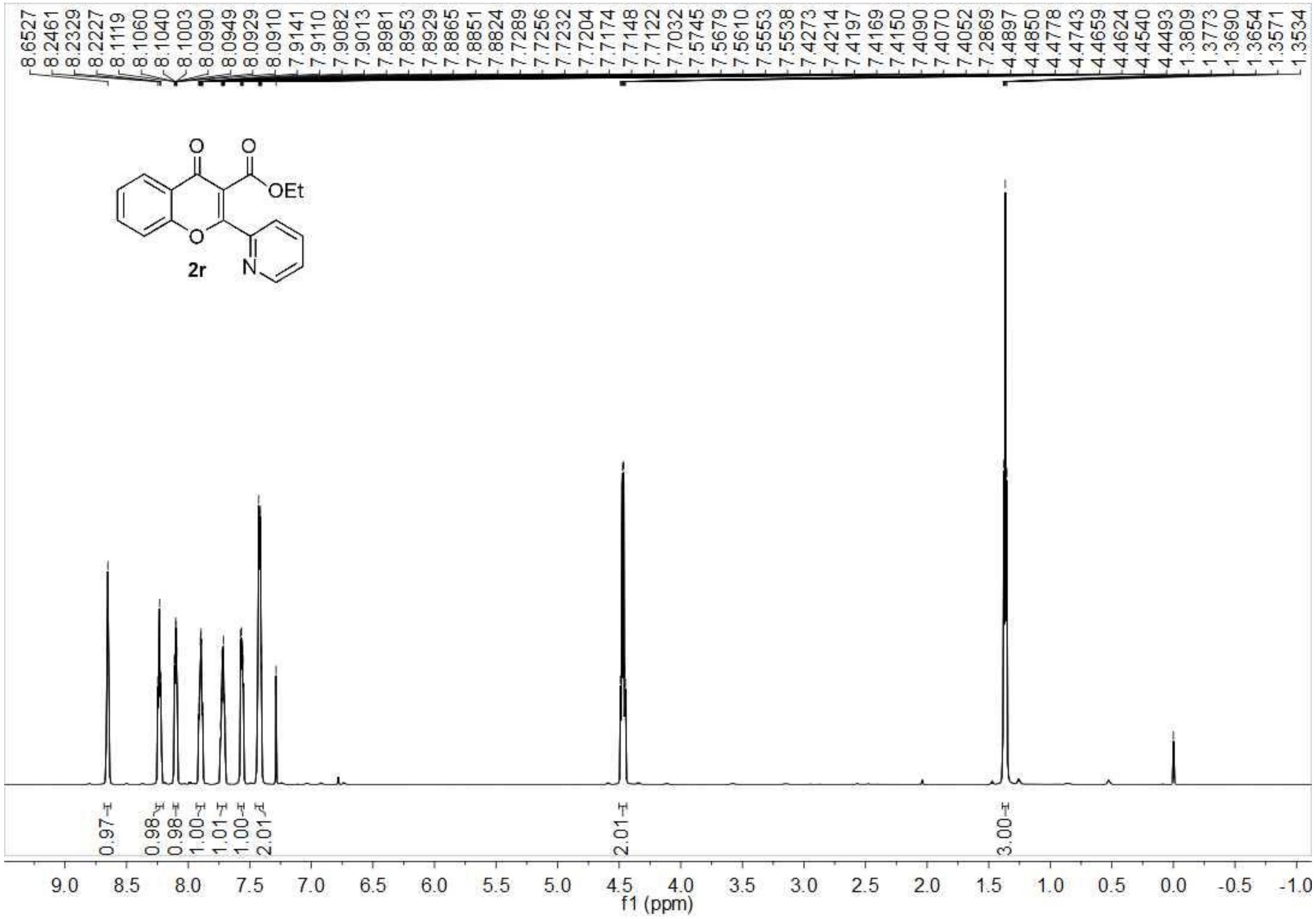
8.6527
8.2461
8.2329
8.2227
8.1119
8.1060
8.1040
8.1003
8.0990
8.0949
8.0929
8.0910
7.9141
7.9110
7.9082
7.9013
7.8981
7.8953
7.8929
7.8865
7.8851
7.8824
7.7289
7.7256
7.7232
7.7204
7.7174
7.7148
7.7122
7.7032
7.5745
7.5679
7.5610
7.5553
7.5538
7.4273
7.4214
7.4197
7.4169
7.4150
7.4090
7.4070
7.4052
7.2869
4.4897
4.4850
4.4778
4.4743
4.4659
4.4624
4.4540
4.4493
1.3809
1.3773
1.3690
1.3654
1.3571
1.3534
OEt
O
N
2r
0.97
0.98
0.98
1.00
1.01
1.00
2.01
2.01
3.00
f1 (ppm)

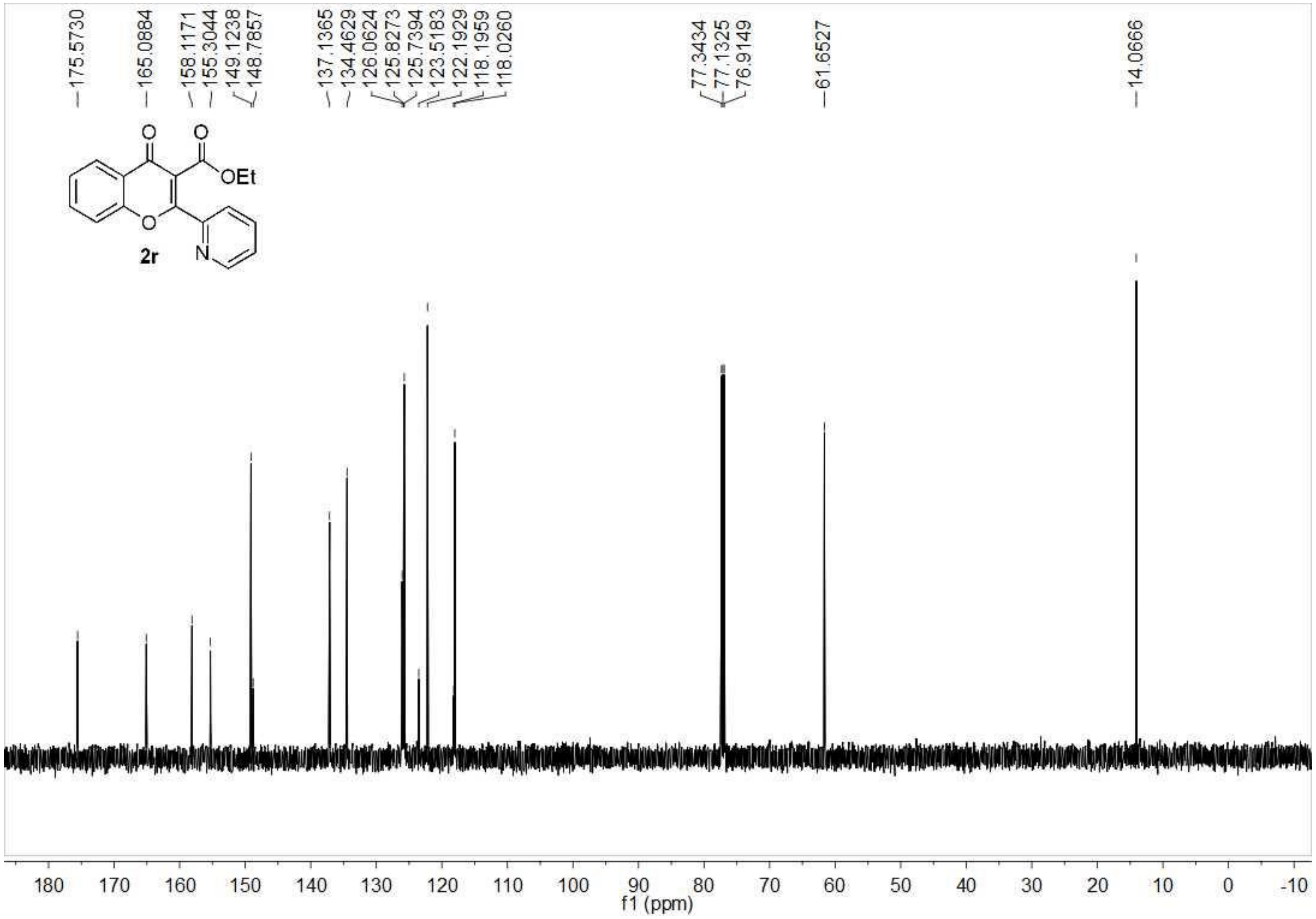

175.5730
165.0884
158.1171
155.3044
149.1238
148.7857
137.1365
134.4629
126.0624
125.8273
125.7394
123.5183
122.1929
118.1959
118.0260
77.3434
77.1325
76.9149
61.6527
14.0666
OEt
O
N
2r
180 170 160 150 140 130 120 110 100 90 80 70 60 50 40 30 20 10 0 -10
f1 (ppm)

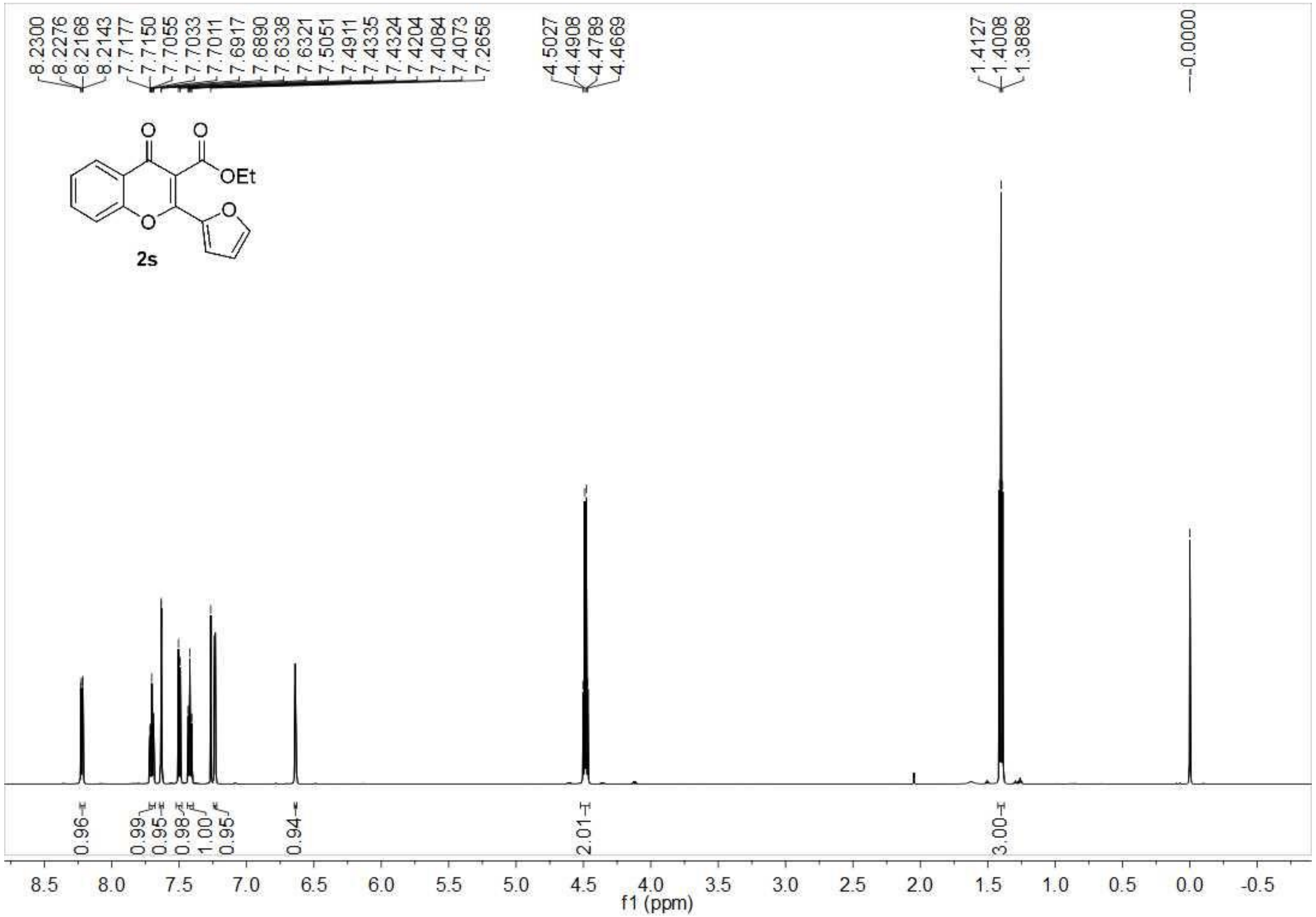
8.2300
8.2276
8.2168
8.2143
7.7177
7.7150
7.7055
7.7033
7.7011
7.6917
7.6890
7.6338
7.6321
7.5051
7.4911
7.4335
7.4324
7.4204
7.4084
7.4073
7.2658
4.5027
4.4908
4.4789
4.4669
1.4127
1.4008
1.3889
0.0000
OEt
2s
0.96
0.99
0.95
0.98
1.00
0.95
0.94
2.01
3.00
f1 (ppm)

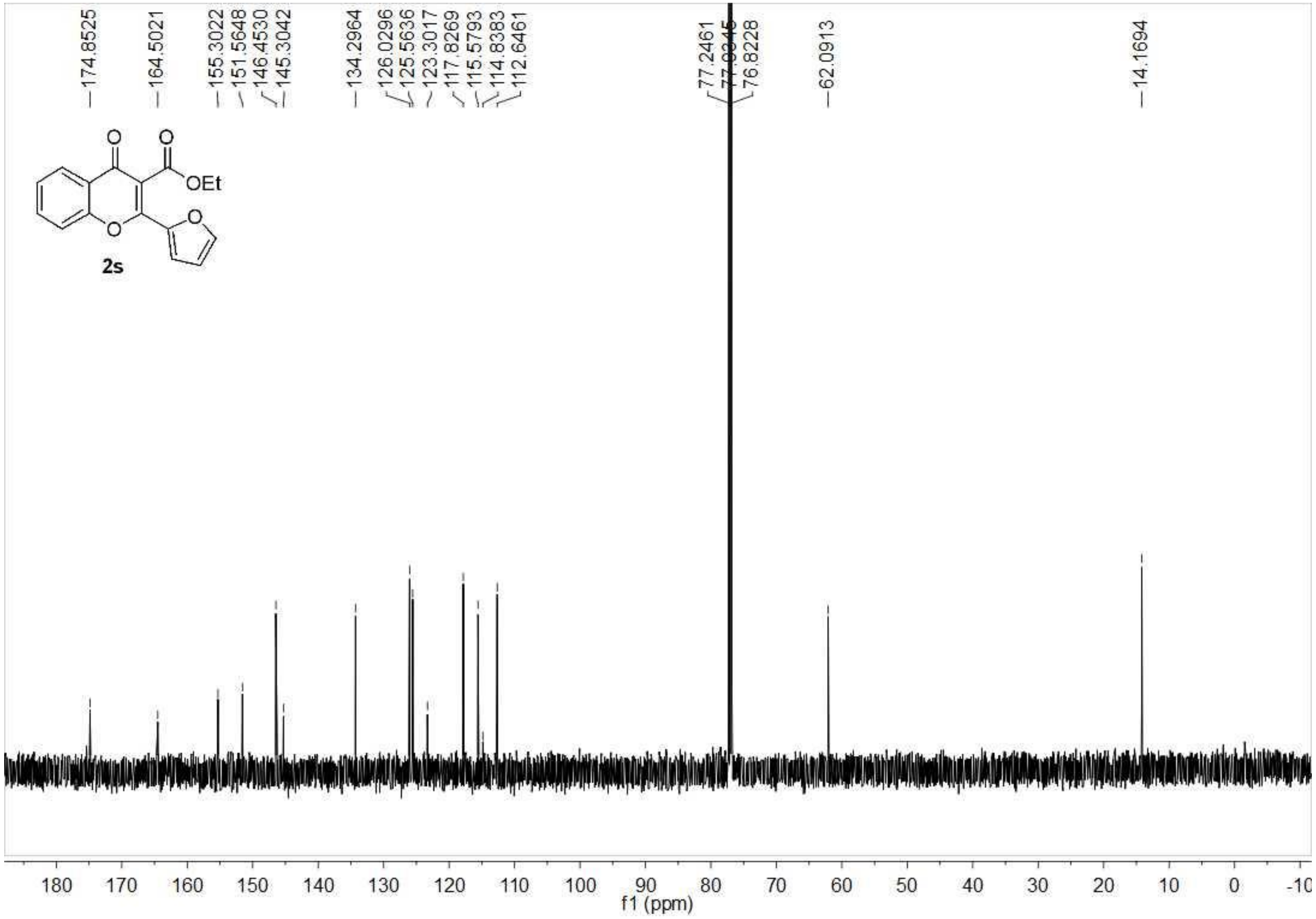

174.8525
164.5021
155.3022
151.5648
146.4530
145.3042
134.2964
126.0296
125.5636
123.3017
117.8269
115.5793
114.8383
112.6461
77.2461
76.8228
62.0913
14.1694
O
O
OEt
O
O
2s
180 170 160 150 140 130 120 110 100 90 80 70 60 50 40 30 20 10 0 -10
f1 (ppm)

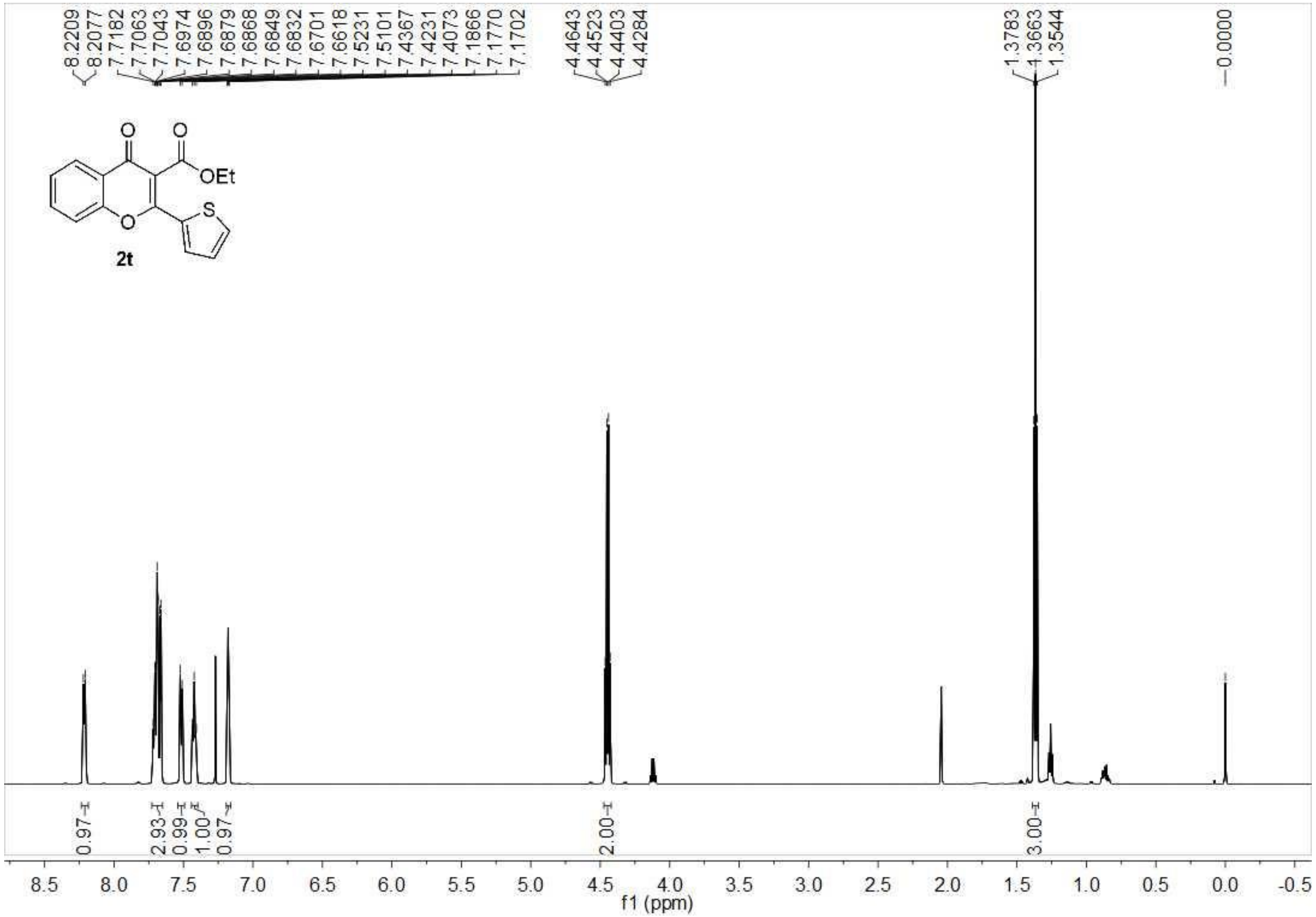
8.2209
8.2077
7.7182
7.7063
7.7043
7.6974
7.6896
7.6879
7.6868
7.6849
7.6832
7.6701
7.6618
7.5231
7.5101
7.4367
7.4231
7.4073
7.1866
7.1770
7.1702
4.4643
4.4523
4.4403
4.4284
1.3783
1.3663
1.3544
0.0000
OEt
2t
0.97
2.93
0.99
1.00
0.97
2.00
3.00
f1 (ppm)

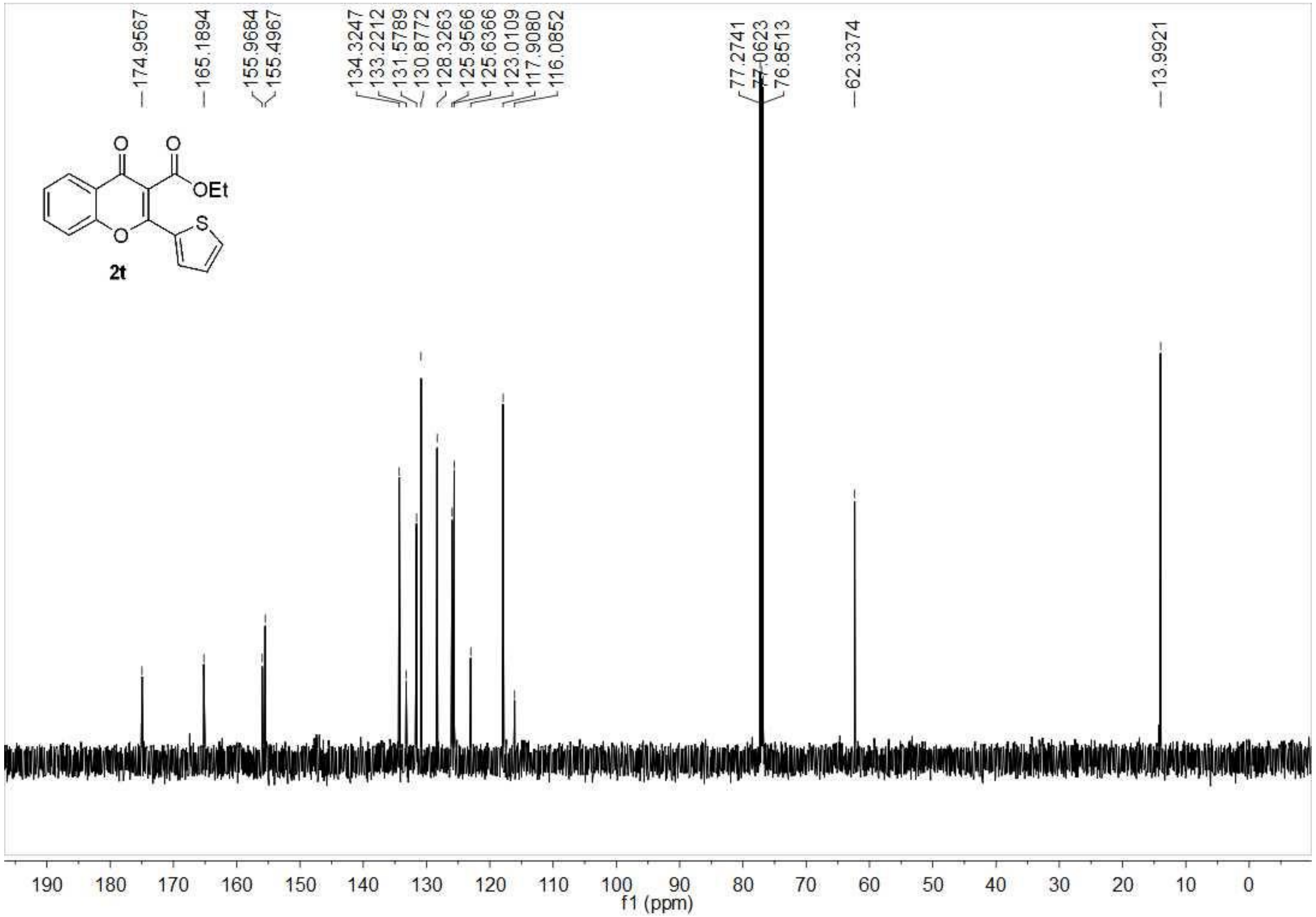

174.9567
165.1894
155.9684
155.4967
134.3247
133.2212
131.5789
130.8772
128.3263
125.9566
125.6366
123.0109
117.9080
116.0852
77.2741
77.0623
76.8513
62.3374
13.9921
OEt
S
O
2t
190
180
170
160
150
140
130
120
110
100
90
80
70
60
50
40
30
20
10
0
f1 (ppm)

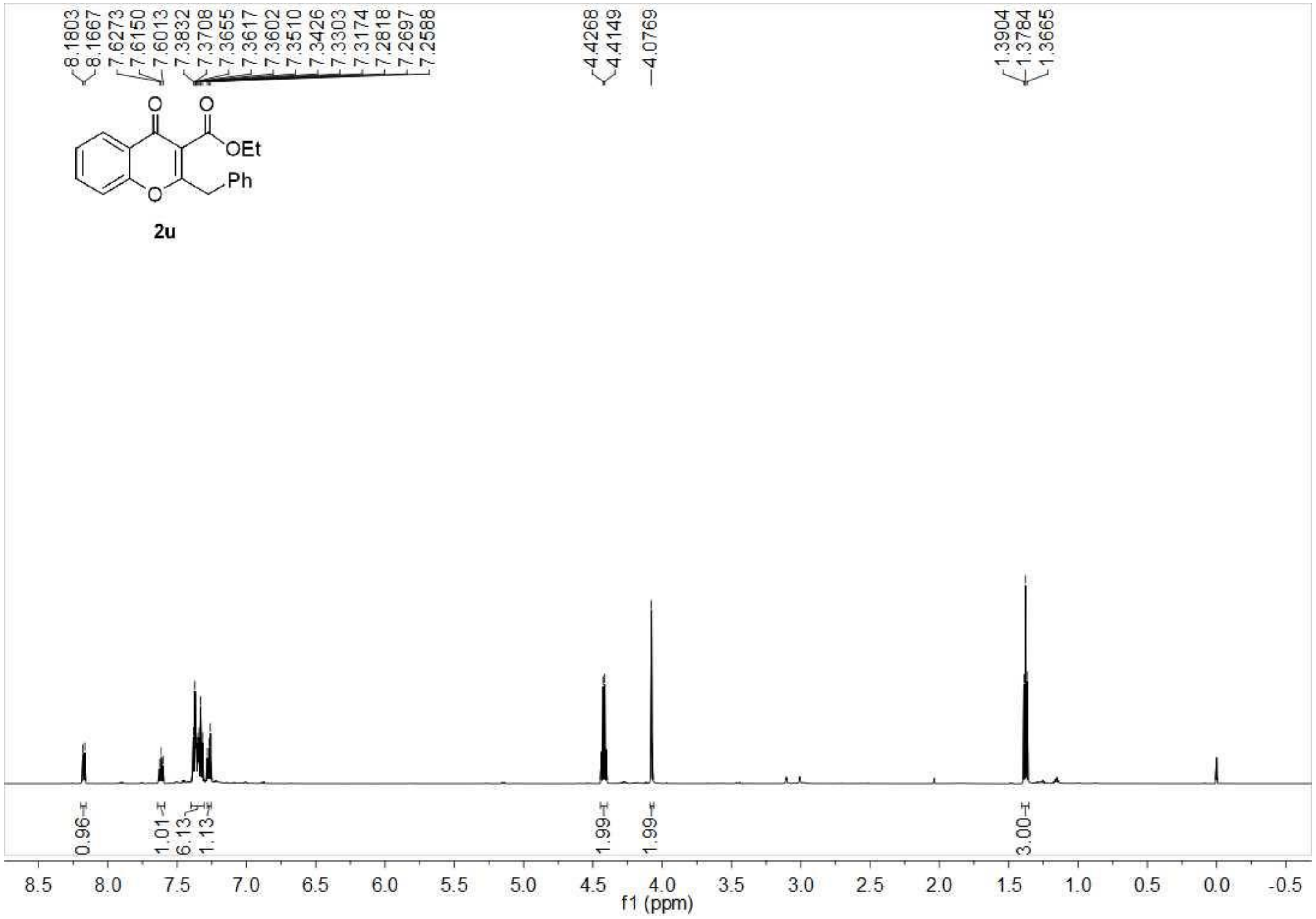
8.1803
8.1667
7.6273
7.6150
7.6013
7.3832
7.3708
7.3655
7.3617
7.3602
7.3510
7.3426
7.3303
7.3174
7.2818
7.2697
7.2588
4.4268
4.4149
4.0769
1.3904
1.3784
1.3665
OEt
Ph
2u
0.96
1.01
6.13
1.13
1.99
1.99
3.00
f1 (ppm)

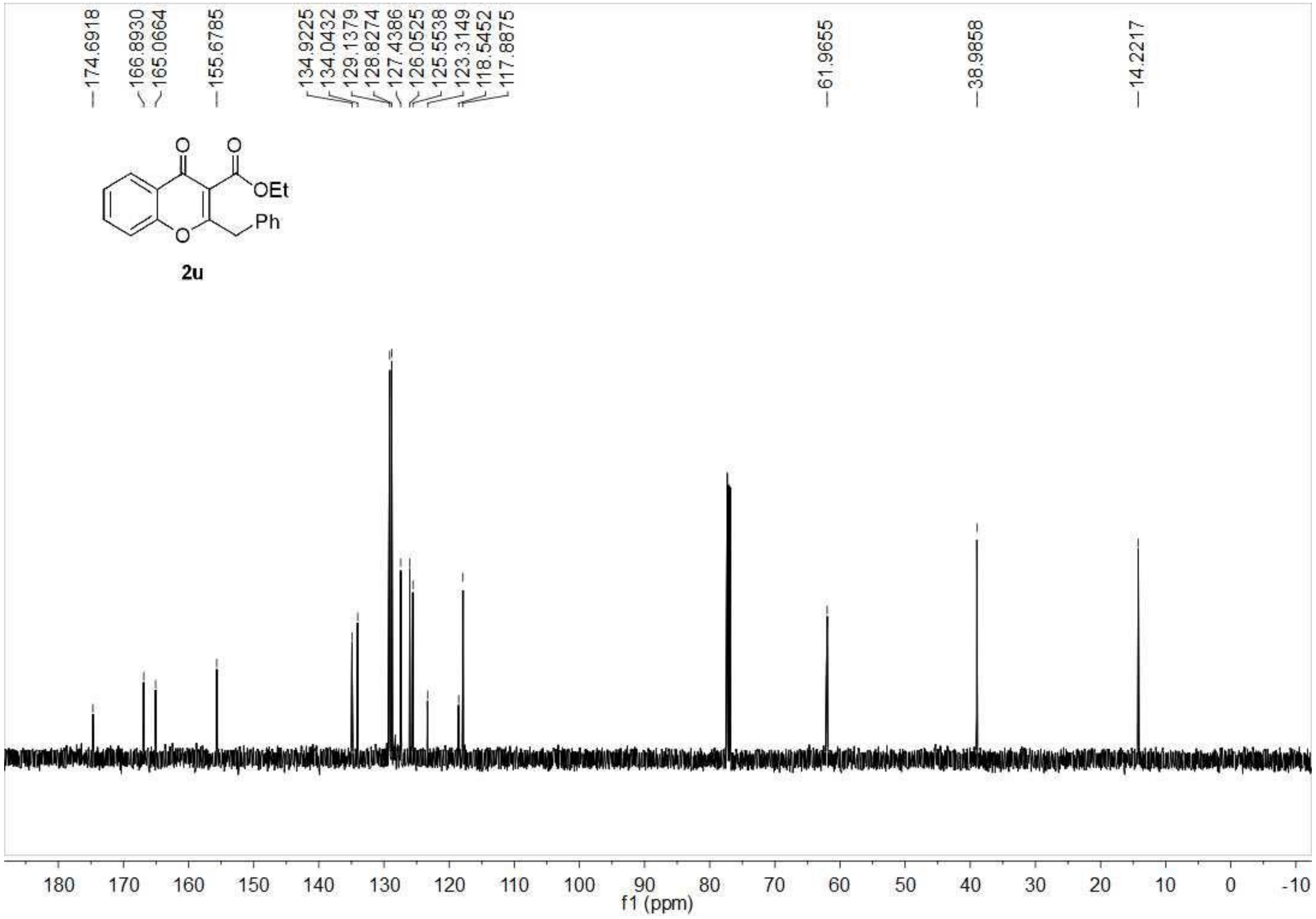
174.6918
166.8930
165.0664
155.6785
134.9225
134.0432
129.1379
128.8274
127.4386
126.0525
125.5538
123.3149
118.5452
117.8875
61.9655
38.9858
14.2217
O
O
OEt
O
Ph
2u
180 170 160 150 140 130 120 110 100 90 80 70 60 50 40 30 20 10 0 -10
f1 (ppm)

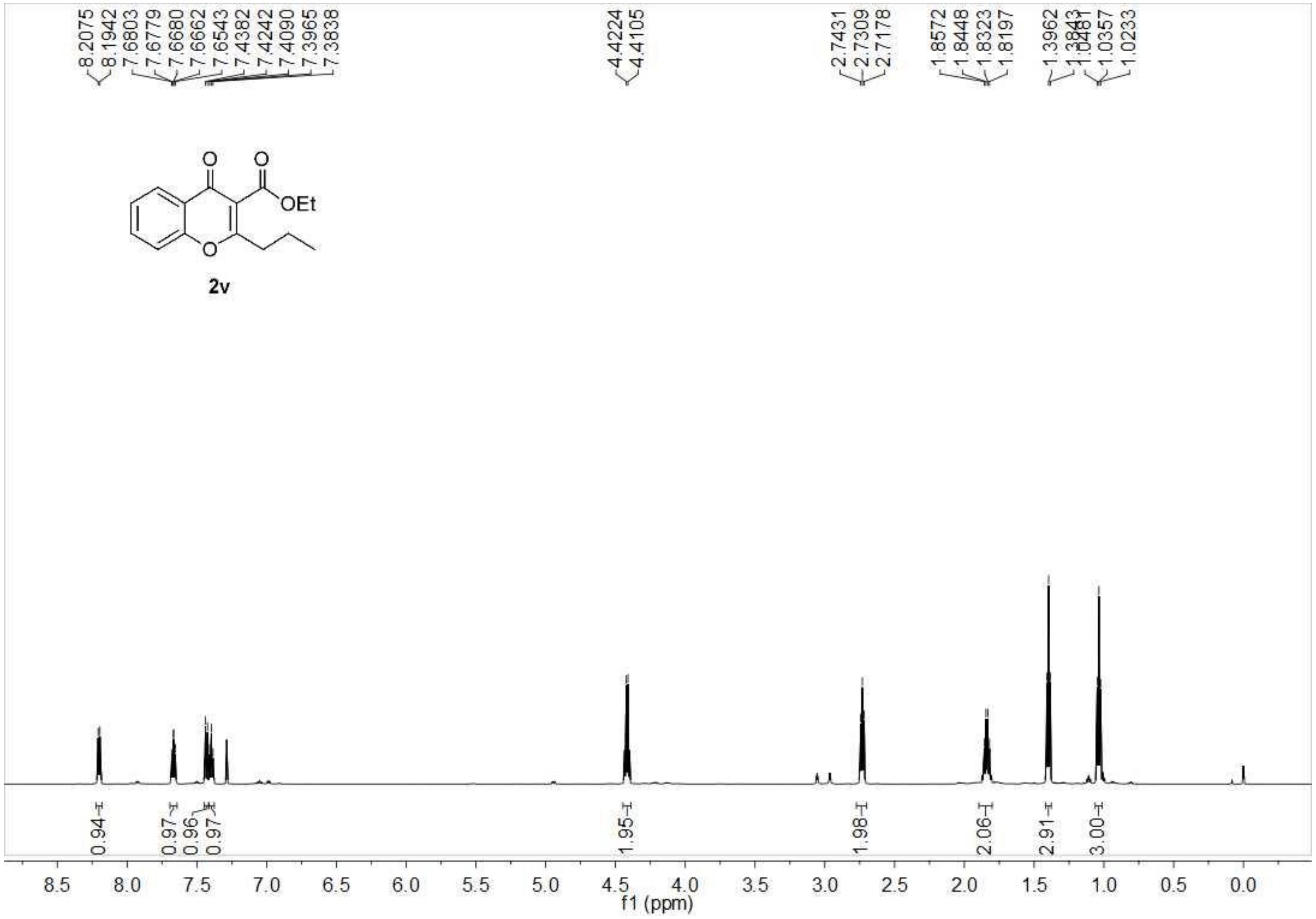

8.2075
8.1942
7.6803
7.6779
7.6680
7.6662
7.6543
7.4382
7.4242
7.4090
7.3965
7.3838
4.4224
4.4105
2.7431
2.7309
2.7178
1.8572
1.8448
1.8323
1.8197
1.3962
1.0357
1.0233
O
O
OEt
O
2v
0.94
0.97
0.96
0.97
1.95
1.98
2.06
2.91
3.00
8.5
8.0
7.5
7.0
6.5
6.0
5.5
5.0
4.5
4.0
3.5
3.0
2.5
2.0
1.5
1.0
0.5
0.0
f1 (ppm)

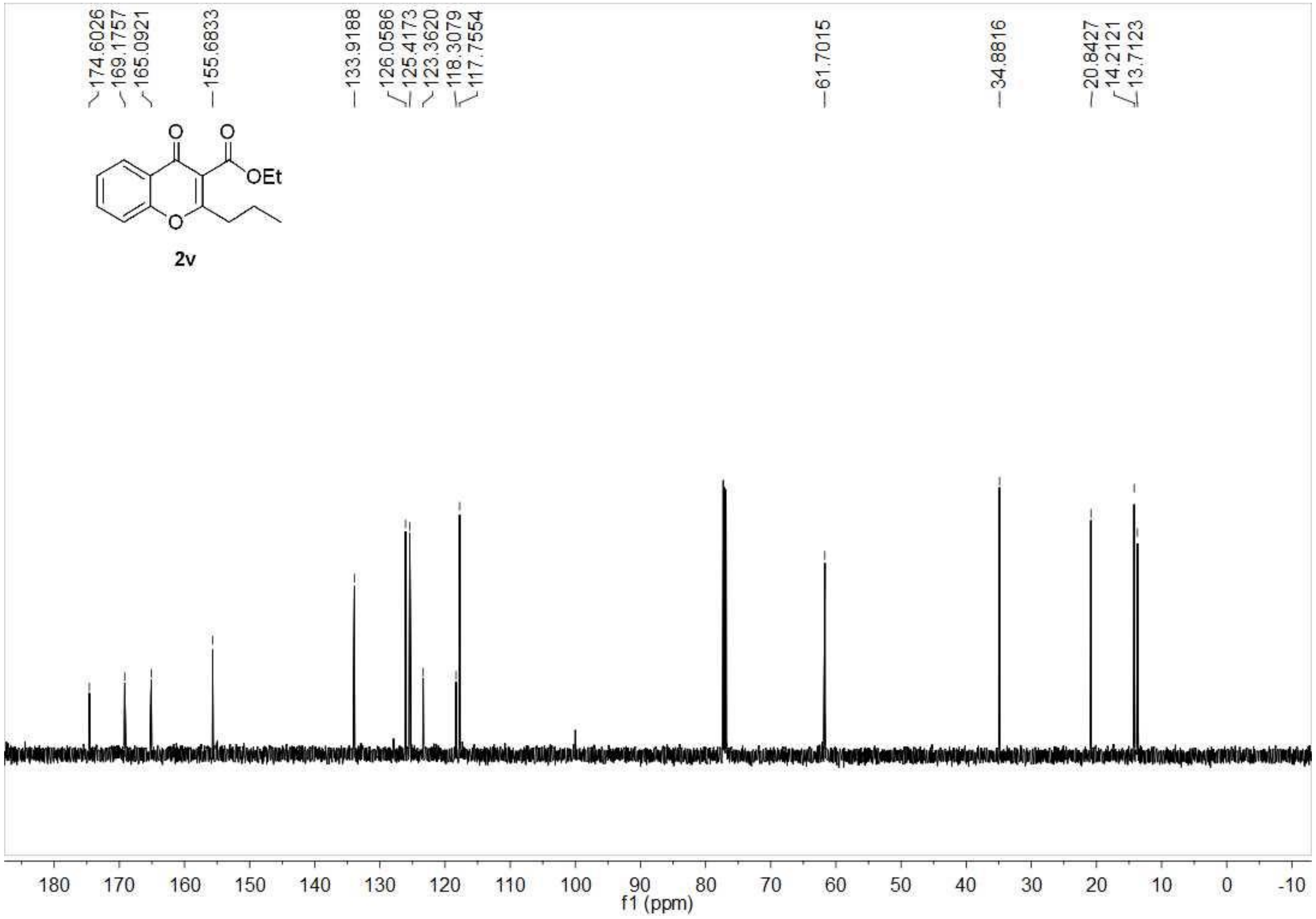
174.6026
169.1757
165.0921
155.6833
133.9188
126.0586
125.4173
123.3620
118.3079
117.7554
61.7015
34.8816
20.8427
14.2121
13.7123
O
O
OEt
O
2v
180
170
160
150
140
130
120
110
100
90
80
70
60
50
40
30
20
10
0
-10
f1 (ppm)

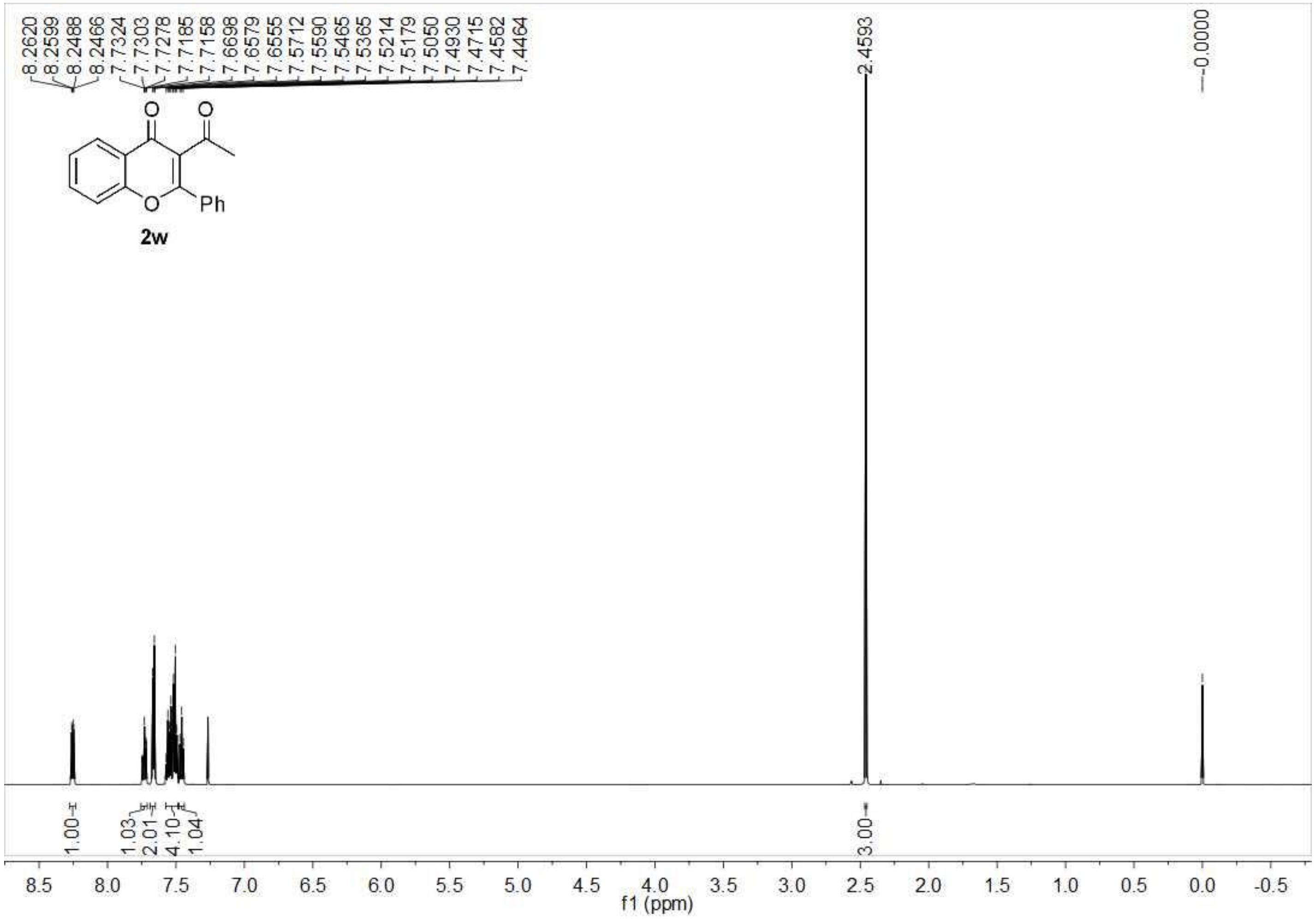
8.2620
8.2599
8.2488
8.2466
7.7324
7.7303
7.7278
7.7185
7.7158
7.6698
7.6579
7.6555
7.5712
7.5590
7.5465
7.5365
7.5214
7.5179
7.5050
7.4930
7.4715
7.4582
7.4464
2.4593
0.0000
O
O
O
Ph
2w
1.00
1.03
2.01
4.10
1.04
3.00
8.5
8.0
7.5
7.0
6.5
6.0
5.5
5.0
4.5
4.0
3.5
3.0
2.5
2.0
1.5
1.0
0.5
0.0
-0.5
f1 (ppm)

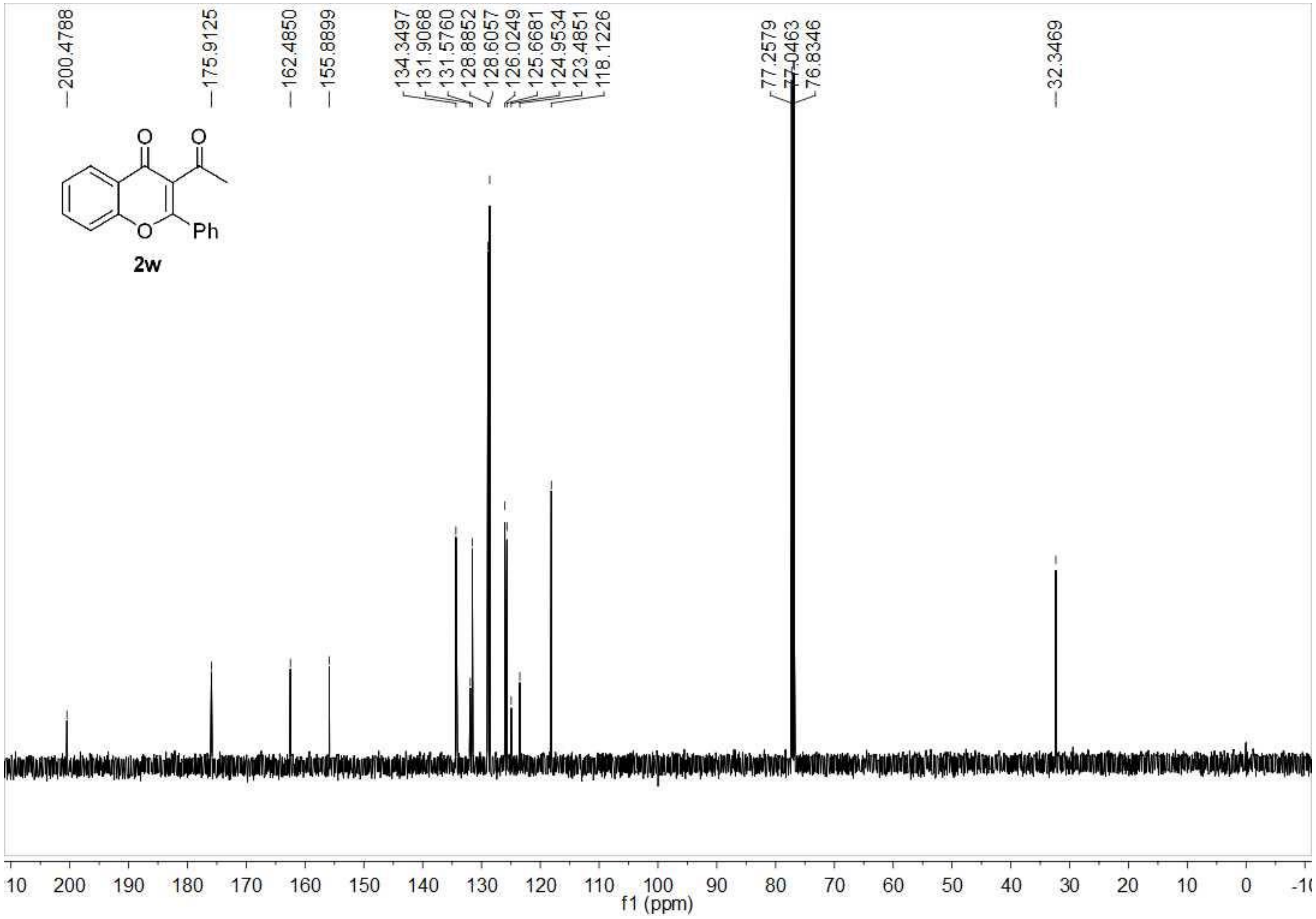
200.4788
175.9125
162.4850
155.8899
134.3497
131.9068
131.5760
128.8852
128.6057
126.0249
125.6681
124.9534
123.4851
118.1226
77.2579
77.0463
76.8346
32.3469
O
O
O
Ph
2w
10 200 190 180 170 160 150 140 130 120 110 100 90 80 70 60 50 40 30 20 10 0 -10
f1 (ppm)

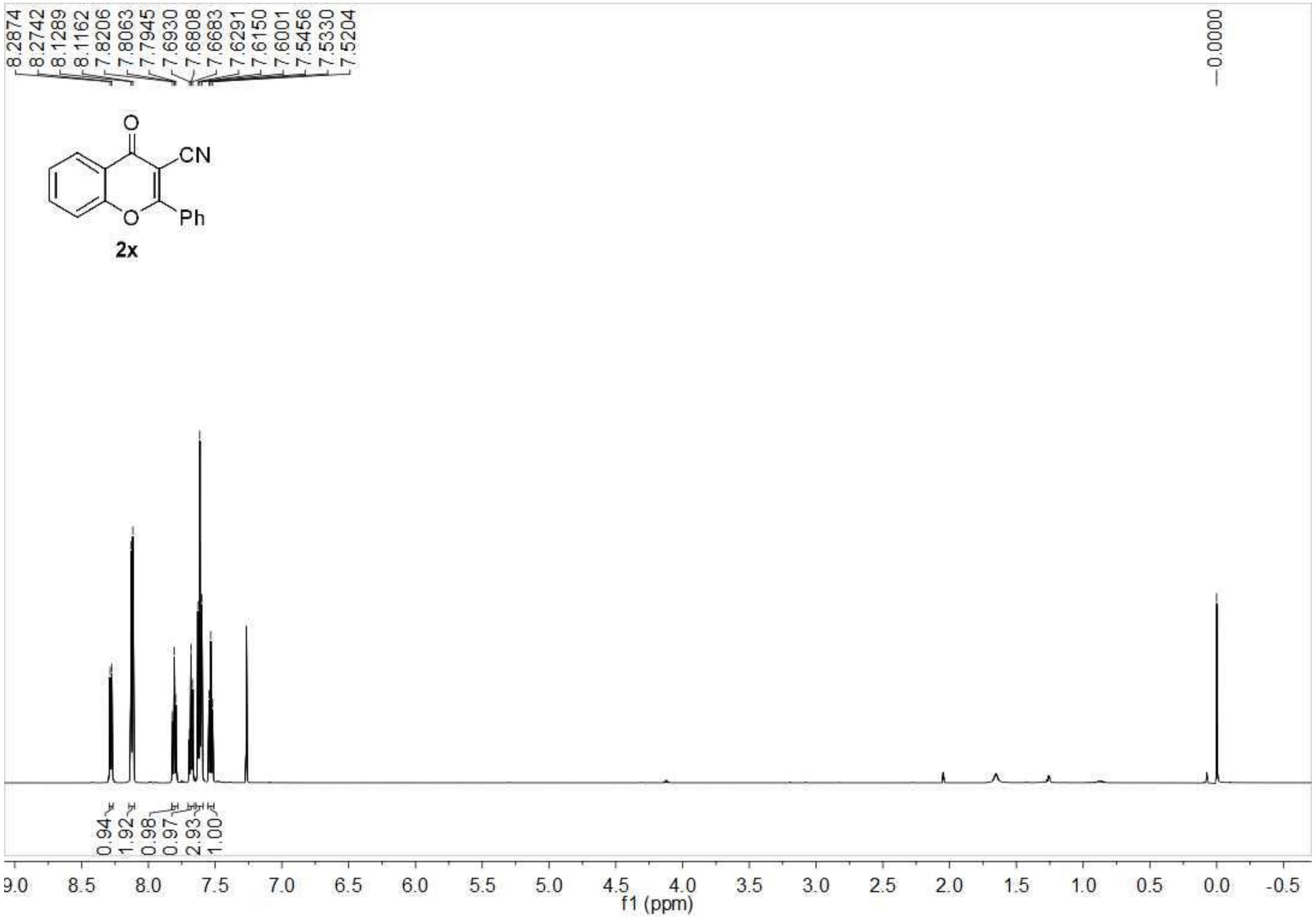

8.2874
8.2742
8.1289
8.1162
7.8206
7.8063
7.7945
7.6930
7.6808
7.6683
7.6291
7.6150
7.6001
7.5456
7.5330
7.5204
0.0000
O
CN
O
Ph
2x
0.94
1.92
0.98
0.97
2.93
1.00
9.0 8.5 8.0 7.5 7.0 6.5 6.0 5.5 5.0 4.5 4.0 3.5 3.0 2.5 2.0 1.5 1.0 0.5 0.0 -0.5
f1 (ppm)

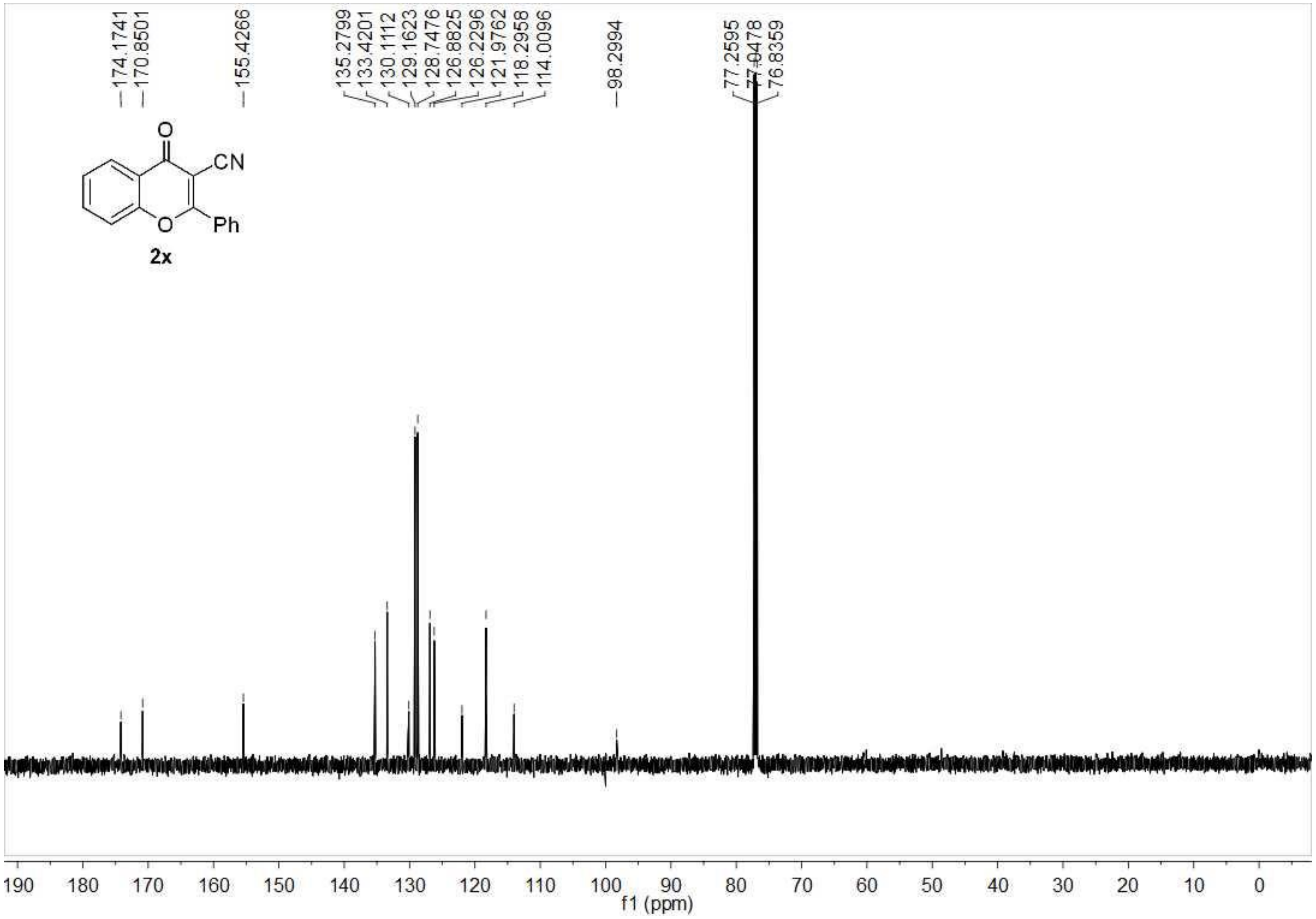
174.1741
170.8501
155.4266
135.2799
133.4201
130.1112
129.1623
128.7476
126.8825
126.2296
121.9762
118.2958
114.0096
98.2994
77.2595
77.0478
76.8359
O
CN
O
Ph
2x
190 180 170 160 150 140 130 120 110 100 90 80 70 60 50 40 30 20 10 0
f1 (ppm)

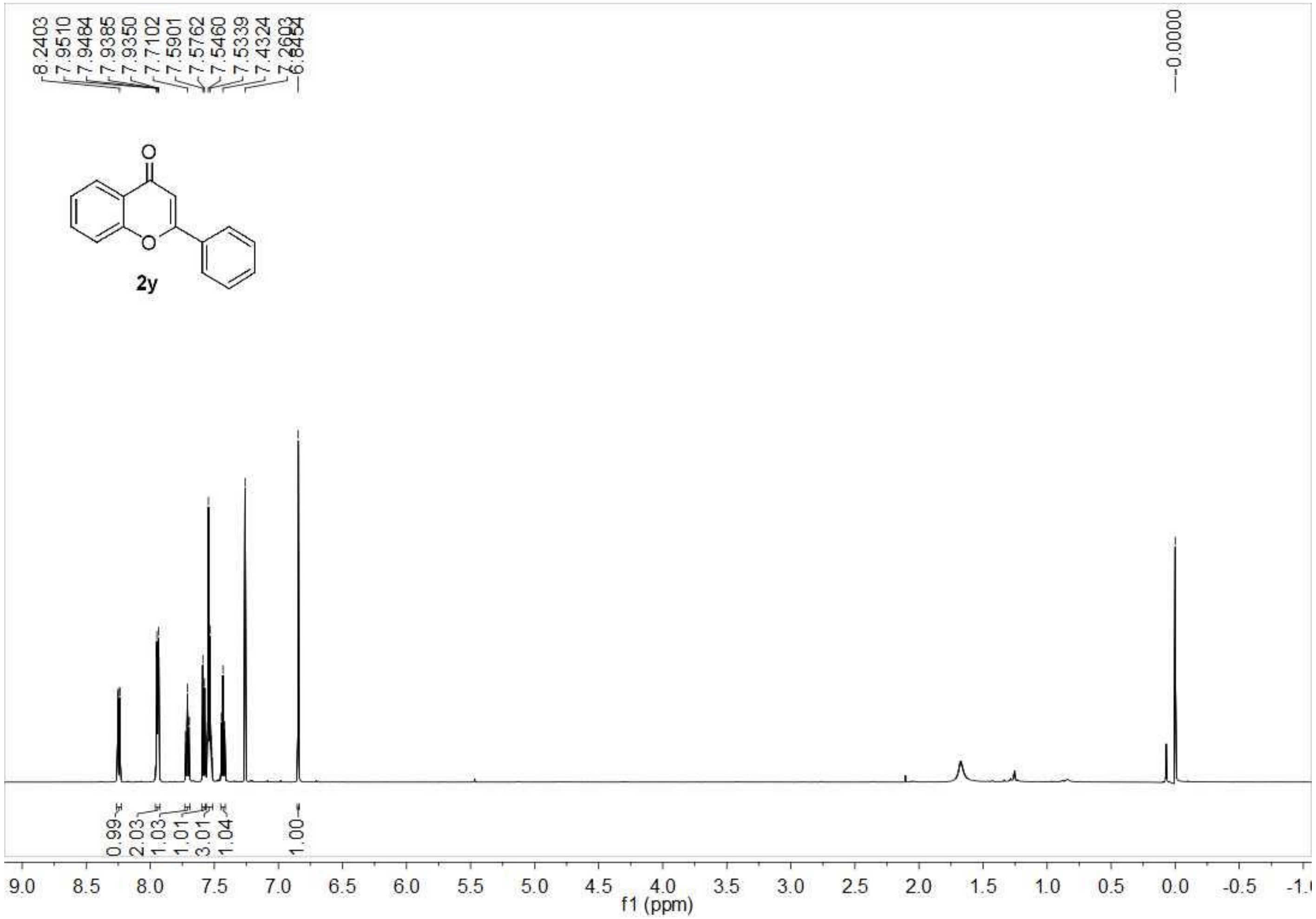
8.2403
7.9510
7.9484
7.9385
7.9350
7.7102
7.5901
7.5762
7.5460
7.5339
7.4324
-0.0000
2y
0.99
2.03
1.03
1.01
3.01
1.04
1.00
9.0 8.5 8.0 7.5 7.0 6.5 6.0 5.5 5.0 4.5 4.0 3.5 3.0 2.5 2.0 1.5 1.0 0.5 0.0 -0.5
f1 (ppm)

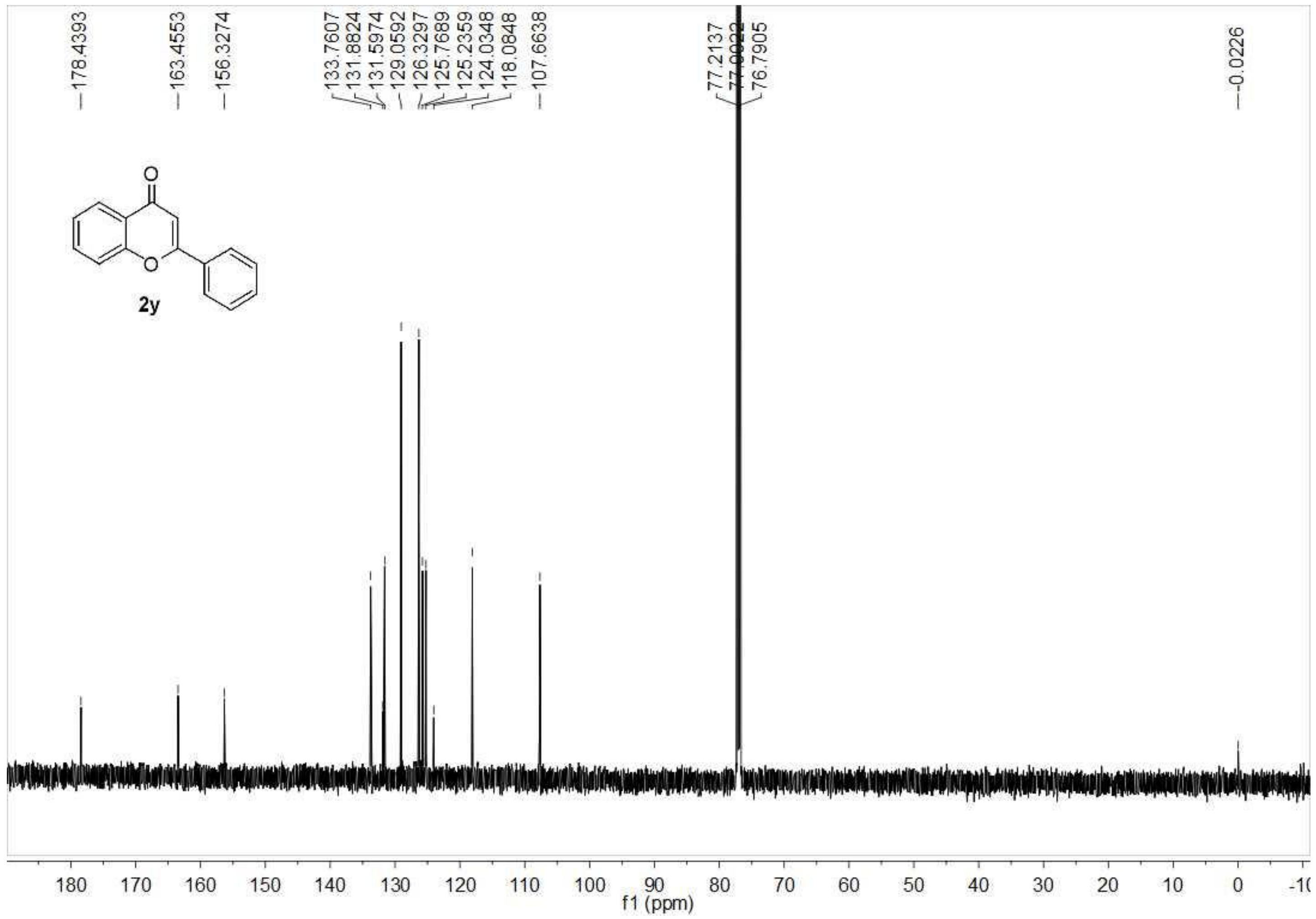
178.4393
163.4553
156.3274
133.7607
131.8824
131.5974
129.0592
126.3297
125.7689
125.2359
124.0348
118.0848
107.6638
77.2137
77.0022
76.7905
-0.0226
2y
f1 (ppm)

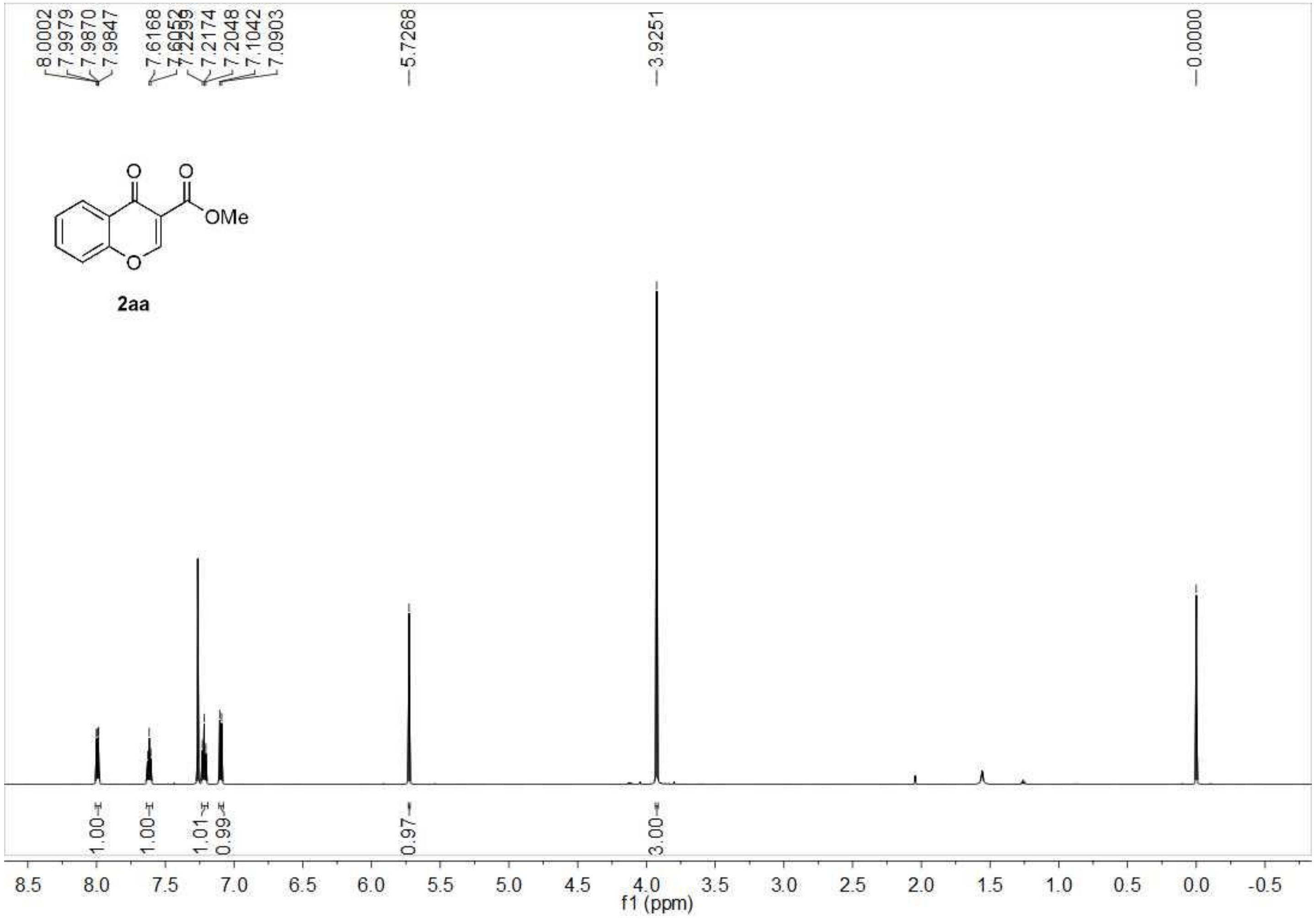
8.0002
7.9979
7.9870
7.9847
7.6168
7.2174
7.2048
7.1042
7.0903
5.7268
3.9251
0.0000
O
O
OMe
O
2aa
1.00
1.00
1.01
0.99
0.97
3.00
8.5 8.0 7.5 7.0 6.5 6.0 5.5 5.0 4.5 4.0 3.5 3.0 2.5 2.0 1.5 1.0 0.5 0.0 -0.5
f1 (ppm)

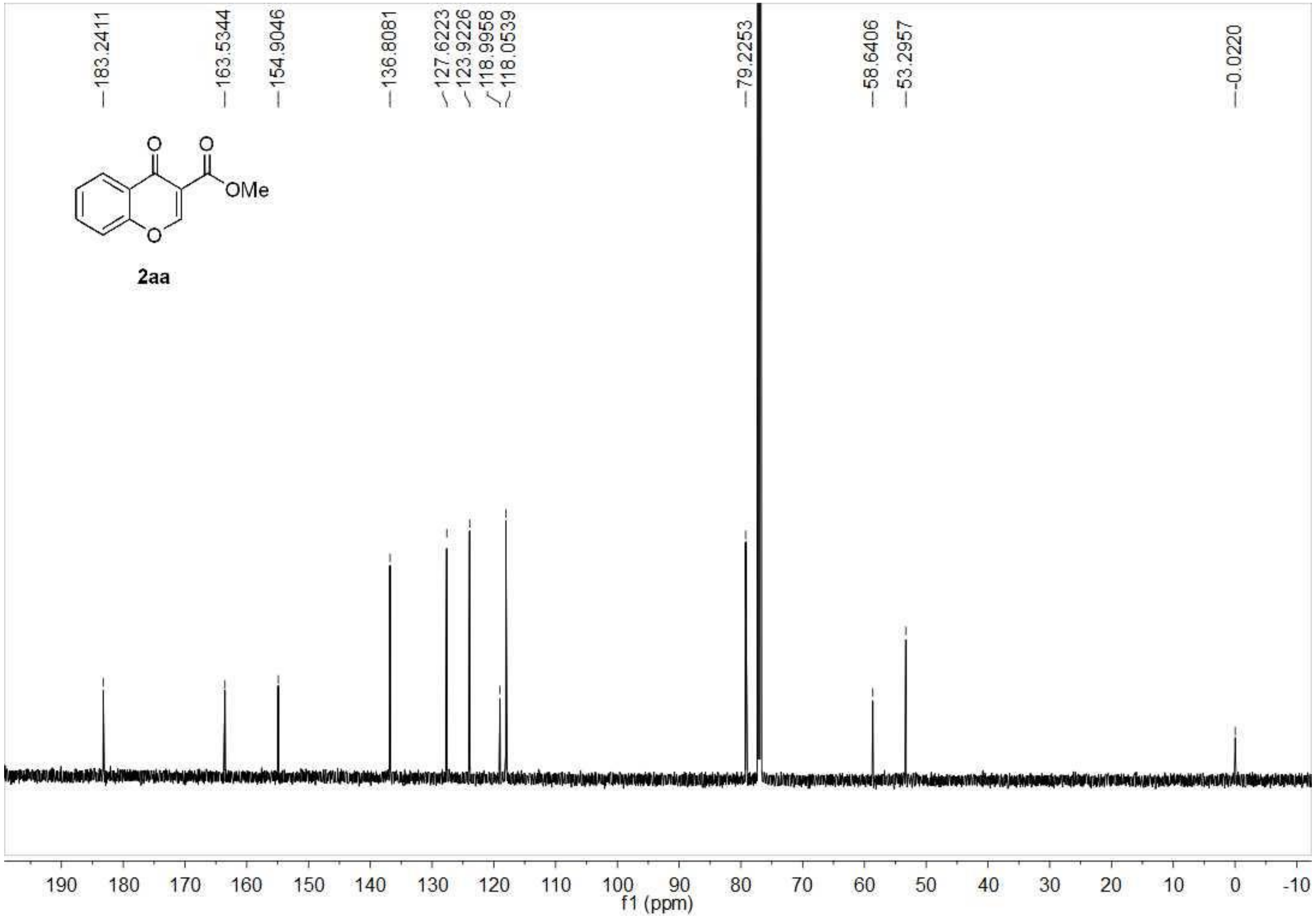
183.2411
163.5344
154.9046
136.8081
127.6223
123.9226
118.9958
118.0539
79.2253
58.6406
53.2957
-0.0220
O
O
OMe
O
2aa
190 180 170 160 150 140 130 120 110 100 90 80 70 60 50 40 30 20 10 0 -10
f1 (ppm)

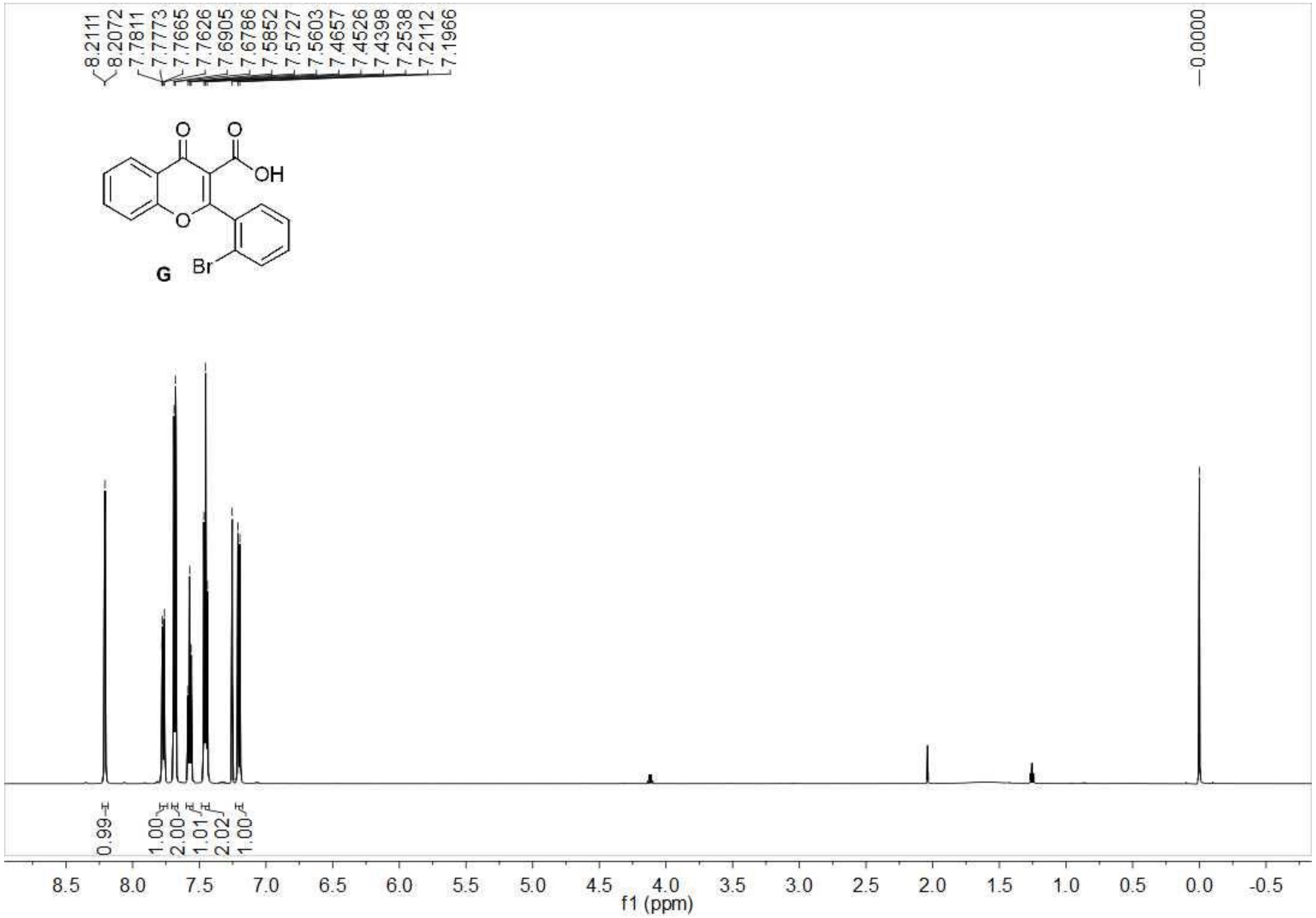

8.2111
8.2072
7.7811
7.7773
7.7665
7.7626
7.6905
7.6786
7.5852
7.5727
7.5603
7.4657
7.4526
7.4398
7.2538
7.2112
7.1966
0.0000
OH
O
Br
G
0.99
1.00
2.00
1.01
2.02
1.00
8.5
8.0
7.5
7.0
6.5
6.0
5.5
5.0
4.5
4.0
3.5
3.0
2.5
2.0
1.5
1.0
0.5
0.0
-0.5
f1 (ppm)

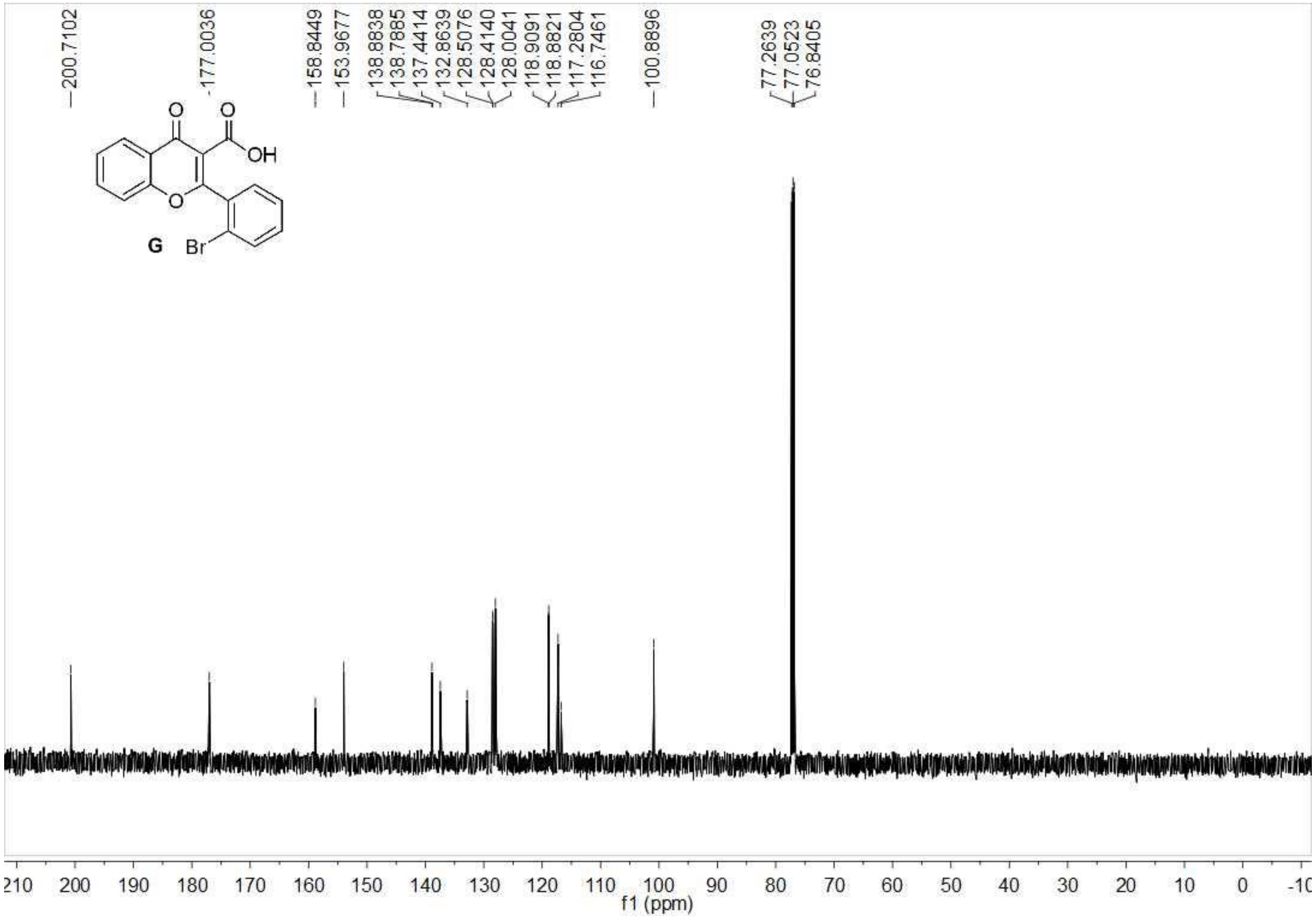

200.7102
177.0036
158.8449
153.9677
138.8838
138.7885
137.4414
132.8639
128.5076
128.4140
128.0041
118.9091
118.8821
117.2804
116.7461
100.8896
77.2639
77.0523
76.8405
O
O
OH
O
G
Br
210 200 190 180 170 160 150 140 130 120 110 100 90 80 70 60 50 40 30 20 10 0 -10
f1 (ppm)

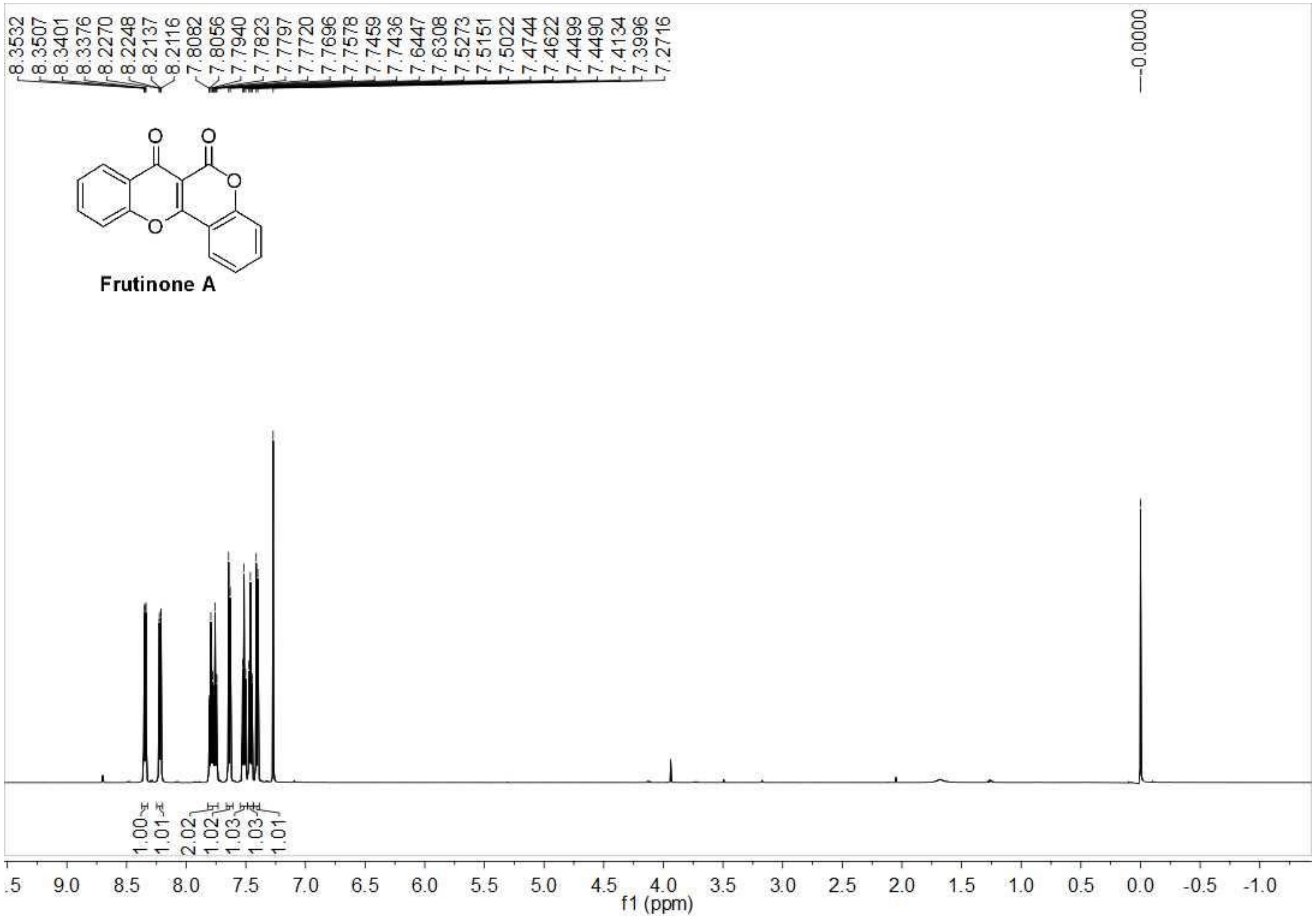
8.3532
8.3507
8.3401
8.3376
8.2270
8.2248
8.2137
8.2116
7.8082
7.8056
7.7940
7.7823
7.7797
7.7720
7.7696
7.7578
7.7459
7.7436
7.6447
7.6308
7.5273
7.5151
7.5022
7.4744
7.4622
7.4499
7.4490
7.4134
7.3996
7.2716
0.0000
Frutinone A
1.00
1.01
2.02
1.02
1.03
1.03
1.01
f1 (ppm)

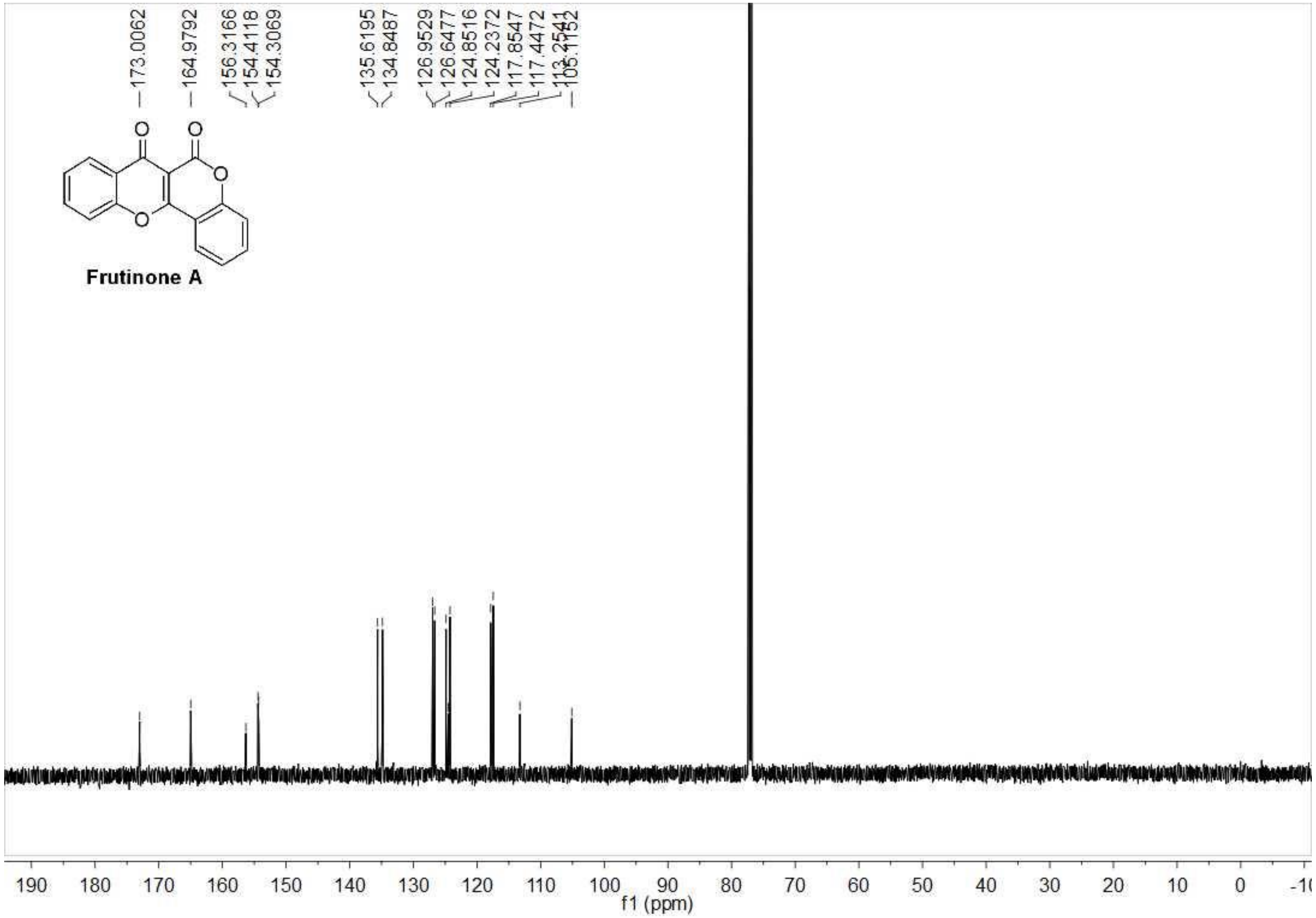
173.0062
164.9792
156.3166
154.4118
154.3069
135.6195
134.8487
126.9529
126.6477
124.8516
124.2372
117.8547
117.4472
113.2541
105.1152
Frutinone A
190 180 170 160 150 140 130 120 110 100 90 80 70 60 50 40 30 20 10 0
f1 (ppm)
